# Chemical synthesis of linear ADP-ribose oligomers up to pentamer and their binding to the oncogenic helicase ALC1†

**DOI:** 10.1039/d1sc02340c

**Published:** 2021-08-18

**Authors:** Qiang Liu, Gunnar Knobloch, Jim Voorneveld, Nico J. Meeuwenoord, Herman S. Overkleeft, Gijsbert A. van der Marel, Andreas G. Ladurner, Dmitri V. Filippov

**Affiliations:** Leiden Institute of Chemistry, Leiden University P.O. Box 9502 2300 RA Leiden The Netherlands filippov@chem.leidenuniv.nl; Biomedical Center (BMC), Physiological Chemistry, Faculty of Medicine LMU Munich 82152 Planegg-Martinsried Germany andreas.ladurner@bmc.med.lmu.de; Eisbach Bio GmbH Am Klopferspitz 19, Planegg-Martinsried 82152 Germany

## Abstract

ADP-ribosylation is a pivotal post-translational modification that mediates various important cellular processes producing negatively charged biopolymer, poly (ADP-ribose), the functions of which need further elucidation. Toward this end, the availability of well-defined ADP-ribose (ADPr) oligomers in sufficient quantities is a necessity. In this work, we demonstrate the chemical synthesis of linear ADPr oligomers of defined, increasing length using a modified solid phase synthesis method. An advanced phosphoramidite building block temporarily protected with the base sensitive Fm-group was designed and implemented in the repeating pyrophosphate formation *via* a P(v)–P(iii) coupling procedure on Tentagel solid support. Linear ADPr oligomers up to a pentamer were successfully synthesized and their affinity for the poly-(ADP-ribose)-binding macrodomain of the human oncogenic helicase and chromatin remodeling enzyme ALC1 was determined. Our data reveal a length-dependent binding manner of the nucleic acid, with larger ADPr oligomers exhibiting higher binding enthalpies for ALC1, illustrating how the activity of this molecular machine is gated by PAR.

## Abstract 2

We report the synthesis of linear ADPr oligomers of defined length up to a pentamer using an improved solid phase method. Binding study with human oncogenic helicase ALC1 shows that ADPr oligomers bind to ALC1 in a length-dependent manner.

## Graphical abstract

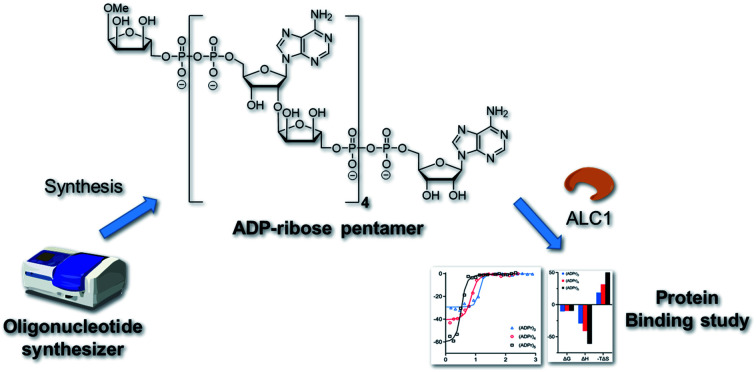

## Introduction

**Fig. 1:**
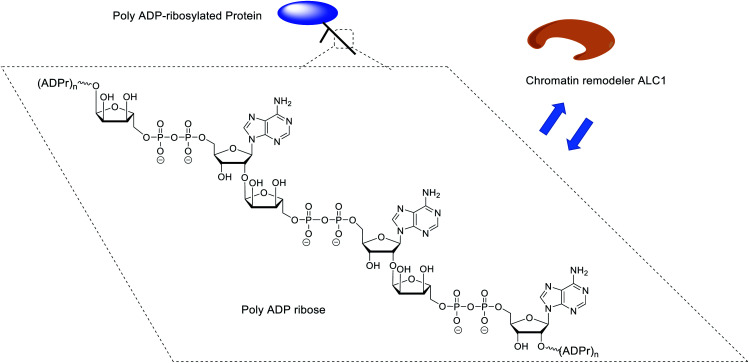
The structure of poly ADP ribose and its binding with ALC1.

ADP-ribosylation is a post-translational modification that plays a crucial role in various important cellular events.^1^ ADP-ribosylation is catalyzed by a family of ADP-ribosyl-transferases (ARTs) that mediate the transfer of a single ADP-ribose (ADPr) moiety from NAD^+^ to nucleophilic side chains of specific amino acids in target proteins.^2^ Some of these enzymes, such as the cancer-relevant PARP1 and PARP2 enzymes, can also add additional ADPr units, generating poly-ADPr (PAR) chains up to a length of hundreds of ADPr units.^3^ PAR exists not only as linear, but also as branched polymers.^4^ Although the nature of the PAR polymers and the amino acids that can be modified by PAR is known, insights into their interactions at the molecular level with associated proteins is far from complete. Some proteins recognize mono-ADP-ribosylated proteins, whilst others require oligomers of ADPr for binding and function. A prime example of the latter and subject of the study presented here is the human chromatin remodeling enzyme ALC1 (CHD1L), which binds PAR polymers with high affinity and is allosterically activated by a minimum of three ADPr units (a trimer of ADPr), but does not bind, nor is catalytically activated by monomeric ADPr (Fig. 1).^5^

To dissect these protein–PAR interactions at the molecular level, the availability of well-defined, chemically pure ADPr oligomers would be highly desirable since PAR is a heterogenous molecule. Although enzymatically prepared PAR fragments have been applied for biological experiments,^6–8^ enzymatic synthesis is not always sufficient in terms of the homogeneousness and the quantity of the produced samples. Chemical synthesis is a relevant alternative when multi-milligram quantities of relatively short well-defined ADPr oligomers are desirable,^9^ as illustrated by the synthesis of a ADPr dimer^10^ and a trimer.^11^ Ensuing studies revealed the crystallographic structures of human NudT16, a hydrolase that is involved in maintaining chromosome stability and cell growth, in complex with monomeric and dimeric ADP-ribose.^12^ We have previously shown that a synthetic ADPr trimer, in sharp contrast to the corresponding dimer and monomer, potently regulates the ATPase activity of the chromatin remodeler ALC1 *in vitro*, a helicase involved in oncogenesis and DNA damage signaling.^5,13–16^ Further, although up to now most reported PAR-binding proteins/domains recognize only mono- or di-ADPr,^3,17,18^ we surmise that proteins/domains might exist that specifically show a preference for longer PAR oligomers. The availability of sufficient quantities of linear PAR oligomers of predetermined length would facilitate such investigations, including at the structural level, and contribute to a better understanding of the molecular functions of PAR,^10,19^ thereby contributing to future insights in oncology,^5,20^ neurodegeneration^21^ and inflammation,^22^ a prerequisite to the establishment of new therapeutic opportunities.

**Fig. 2:**
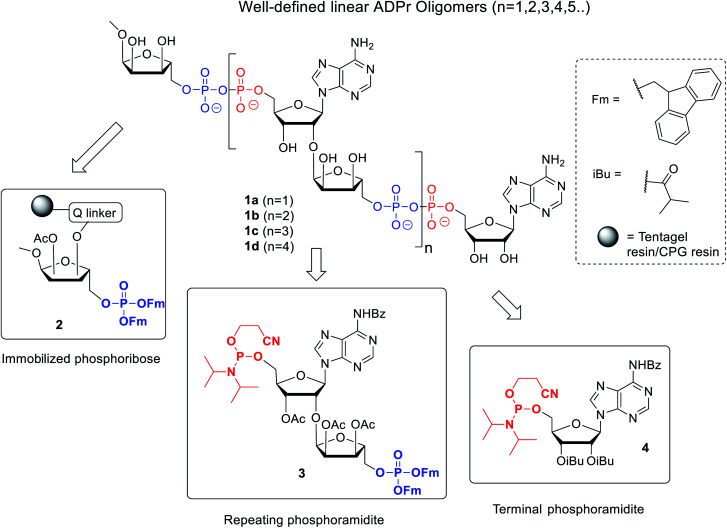
Retrosynthetic analysis of the synthesis of linear ADPr oligomers **1**.

Here we describe an improved method towards the solid phase synthesis of linear ADPr oligomers (**1**, Fig. 2). Our method to prepare pyrophosphate diesters is based on a combination of P(v)–P(iii) chemistry^23^ and adaptation of this method to the solid phase synthesis of linear ADPr oligomers resulted in the assembly of an ADPr trimer.^11^ We reasoned that the observed limitation in length of the oligomer possibly originated from the protective group pattern of the applied building blocks, resulting in repetitive exposure of the growing ADPr-chain to acidic conditions.^11^ It is known that the pyrophosphates and *O*-glycosidic linkages in PAR oligomers can be cleaved under acidic conditions, therefor acid sensitive temporary protections may exert an adverse effect on the maximal length of the projected oligomers that can be achieved. For this reason, we replace the acid-labile *tert*-butyl groups^24^ applied in the first procedure by the base-labile fluorenylmethyl (Fm) groups as temporary phosphate protective groups. In order to enable a repetitive assembly of ADPr-chains on solid phase *via* such Fm-based strategy, advanced linear phosphoramidite building block (**3**) was designed and prepared, while a protected phosphoribose moiety immobilized *via* alkali labile Q-linker (**2**) was selected as the starting point of the projected solid phase syntheses. The resulting tetra-ADPr and penta-ADPr molecules were tested for their binding to the macrodomain module of the PAR-regulated chromatin remodeller ALC1 in order to establish their affinity and interaction enthalpies.

## Results and discussion

The presence of difficult to protect anionic pyrophosphates functionalities in ADPr oligomers during the synthesis (**1**, *n* ≥ 1, Fig. 2) makes them poorly soluble in organic solvents and mandate the use of a solid-phase rather than a solution-phase approach. Retro-synthetically, linear ADPr oligomers should be accessible by a solid phase synthesis methodology with the aid of three components: (a) resin **2**, functionalized with an immobilized ribose-5′-phosphate derivative, as start of the synthesis;^11,25^ (b) sufficient quantities of the orthogonally protected phosphoramidite building block **3**, used in all elongation cycles; and (c) known adenosine phosphoramidite **4**, used in the termination cycle.^11,26–28^ As said, because of the inherent acid sensitivity of the sugar-pyrophosphate chain of ADPr, Fm groups were selected to protect the phosphomonoester in the key phosphoramidite **3** of the elongation cycle because of the mild basic conditions for their efficient and repeated removal.

**Scheme 1:**
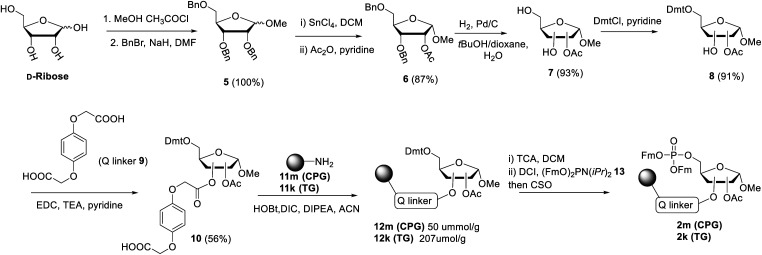
Synthesis of resins **2m** and **2k**, functionalized *via* the Q-linker with a ribose phosphotriester, protected with Fm groups.

The projected solid phase procedure of the linear ADPr target oligomers starts with the preparation of functionalized resins **2m** and **2k**, provided with the alkali sensitive Q-linker (Scheme 1). On the basis of the favorable performance in oligonucleotide synthesis both the usual controlled pore glass support long chain alkylamine (LCAA-CPG) and TentaGel N (TG), consisting of a polystyrene matrix with polyethylene glycol (PEG) linker grafted on it were investigated as solid support. Tentagel N is equally suitable for phosphoramidite chemistry as CPG but due to its swelling properties TG has a higher loading capacity and potentially allows for the synthesis of ADPr oligomers on a bigger scale. The functionalization of these resins is shown in Scheme 1.^11,25^ First, d-ribose was converted *via* two standard reactions into 1-*O*-methyl-2,3,5-tri-*O*-benzyl-ribofuranoside (**5**) as an anomeric mixture. The 2-*O*-benzyl in **5** was selectively removed with SnCl_4_/DCM^29^ and the released hydroxyl was acetylated, after which the pure α-anomer **6** could be isolated. The isomerization of the β- to the desired α-anomer can be explained by the opening and closing of the furanose ring under the influence of Lewis acid (SnCl_4_). Of note is that compared to the previously reported β-linked OMe group at the anomeric position of the terminal ribose of di- and tri-ADPr, we prepare here α-riboside **6** to introduce an α-configured OMe group to the terminus of oligo-ADPr that mimics the natural 1,2-α cis glycosidic bonds of PAR. Pd/C catalyzed hydrogenolysis of the benzyl groups in **6** was accompanied by acetyl migration to give **7**, as a mixture of regioisomers. After protection of the primary OH in **7** with the 4,4′-dimethoxytrityl (Dmt) group to give **8**, the Q-linker (**9**) was appended^11^ with EDC/TEA in pyridine. The obtained acid **10** was used to functionalize both LCAA-CPG **11m** and TentaGel N (TG) **11k** solid supports to yield resins **12m** (50 μmol g^−1^) and **12k** (207 μmol g^−1^), respectively, with desired loadings. Finally, the Fm-protected phosphotriester was installed by the following sequence of reactions. At first, the Dmt group was removed with trichloroacetic acid (TCA) in DCM, followed by phosphitylation of the released primary OH with known bis-(*9H*-fluoren-9-ylmethyl)-diisopropylamidophosphite **13** and, finally, oxidation of the intermediate phosphite triester by (1*S*)-(+)-(camphorsulfonyl)oxaziridine (CSO) to give either CPG **2m** and TG **2k**, respectively. The successful phosphorylation was indicated qualitatively by ^31^P-NMR analysis of the mixture obtained by NH_4_OH treatment of the solid supports **2m** and **2k**.

**Scheme 2:**
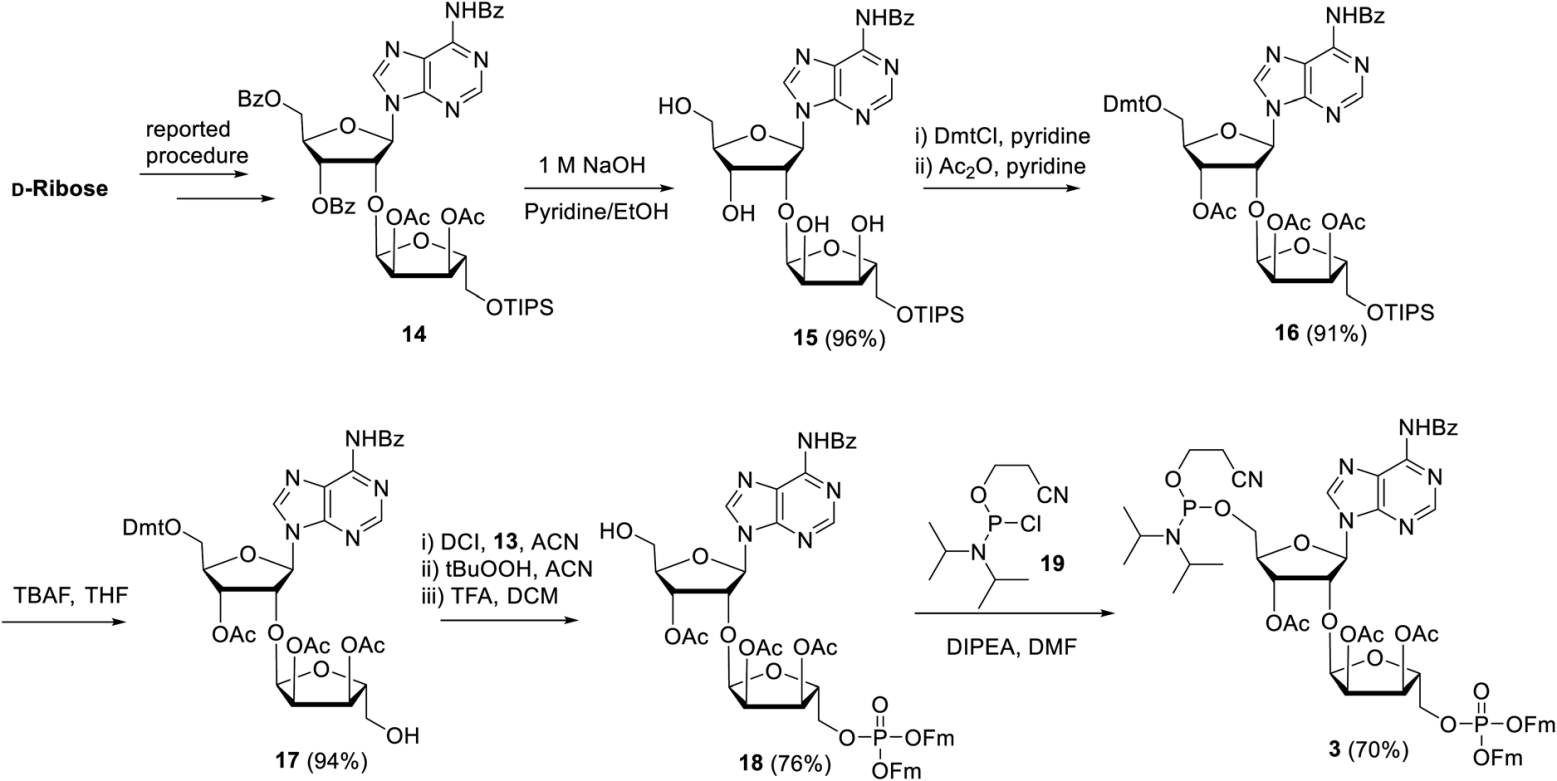
Synthesis of key repeating phosphoramidite **3**.

Next, attention is directed to the preparation of advanced building block **3**, which is repeatedly needed in the elongation cycle of the solid phase synthesis towards the target ADPr oligomers (Scheme 2). Known α-ribosylated adenosine **14** (ref. 11) was converted in tetraol **15** by saponification of the benzoyl and acetyl esters with aqueous NaOH (1 M) to give after column chromatography **15** in excellent yield (96%). Subsequent selective dimethoxytritylation of the free primary hydroxyl followed by acetylation of the remaining secondary hydroxyls led to the isolation of **16** in high yield and in sufficient amount (2.3 mmol). Careful removal of TIPS group in **16** by TBAF liberated terminal 5′′-OH (**17**), allowing the sequential introduction a phosphate triester and phosphoramidite function by single-operation cascade. The first step consisted of 4,5-dicyanoimidazole (DCI)-catalyzed phosphitylation of the primary OH-group in **17** with Fm amidite **13** ^30,31^ followed by oxidation of the resulting phosphite to the phosphate triester by *t*BuOOH. In the second step TFA was used to rapidly remove the Dmt leading to compound **18**, having a free 5-OH′. Finally, key phosphoramidite **3** was obtained by treatment of alcohol **18** with standard 2-cyanoethyl *N*,*N*-diisopropylchlorophosphoramidite (**19**) and DIPEA in DMF. It is important to note that the latter phosphitylation needs a careful work-up procedure, in which DIPEA is completely removed, as DIPEA is capable to cleave one of the base labile Fm groups of the phosphate triester in **3**. We found that H-phosphonate byproduct formed by hydrolysis of **19** is difficult to completely remove from **3** using column chromatography, but since it does not interfere with the pyrophosphate formation we used phosphoramidite **3** with traces of the H-phosphonate in the solid-phase synthesis. In addition, the simultaneous presence of an acid labile (phosphoramidite) and the base labile (Fm) groups in building block **3** requires column chromatography with high-quality IRR silica gel instead of the traditional TEA-neutralized one.

**Scheme 3:**
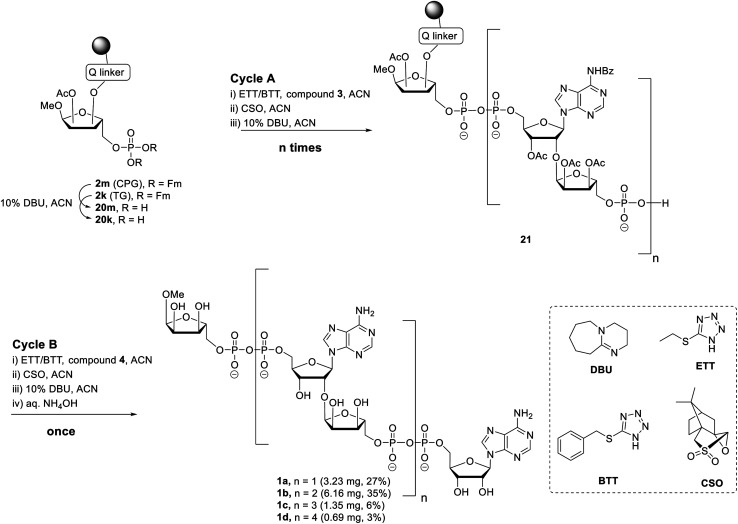
Synthesis of linear ADPr oligomers **1a–d**.

Having all the components (*i.e.***2m**, **2k** and **3**) at our disposal, the projected solid-phase synthesis of ADPr oligomers can be performed. In the first instance, the feasibility and efficiency of the pyrophosphate formation was evaluated by the synthesis of a linear ADPr dimer in a fritted syringe *via* manual couplings (Scheme 3).

The influence of solid support was determined by the application of both CPG **2m** and TG **2k** supports. Removal of the Fm groups of **2m**/**k** by DBU treatment (10% in ACN, 15 min × 2) gave phosphomonoester (**20m**/**k**) suitable for pyrophosphate coupling using our P(v)–P(iii) coupling procedure. To this end the following 3-step elongation cycle A was executed: (i) treatment of immobilized phosphate monoester **20m**/**k** with phosphoramidite building block **3** using 5-(ethylthio)-1*H*-tetrazole (ETT) as activator (10 min × 2); (ii) CSO mediated oxidation of the formed phosphate–phosphite (P^V^–P^III^) to a phosphate–phosphate (P^V^–P^V^) intermediate (5 min × 2); (iii) treatment of the immobilized and partly protected pyrophosphate with DBU (10% in ACN, 15 min × 2) to simultaneously remove both the cyanoethyl (CE) group on the pyrophosphate and the Fm groups on the terminal phosphate. Completion of elongation cycle A gave the immobilized phosphomonoester **21** (*n* = 1), ready for the introduction of the second pyrophosphate. To obtain the ADPr dimer **1a** the synthesis was continued with a similar three step termination cycle B in which phosphoramidite **4** was used in “step i” to form the phosphate–phosphite (P^V^–P^III^) intermediate. After completion of cycle B, the resin was treated with NH_4_OH to remove all the protecting groups and to release the target ADPr dimer from the resin. Subsequent anion exchange chromatography purification yielded 0.32 mg (3%) and 3.23 mg (27%) of dimer **1a** from CPG and TG resin, respectively. This result suggests that for this manual oligo-ADPr synthesis the TG resin is more preferable than the CPG resin.^11,32^ In both syntheses, we found 1-*O*-methyl mono-ADPr side product which may be attributed to an inefficient pyrophosphate coupling of the cycle A. The surprisingly low yield in the CPG supported synthesis could be attributed to the degradation of the support under the repetitive DBU treatment.^33^

**Fig. 3:**
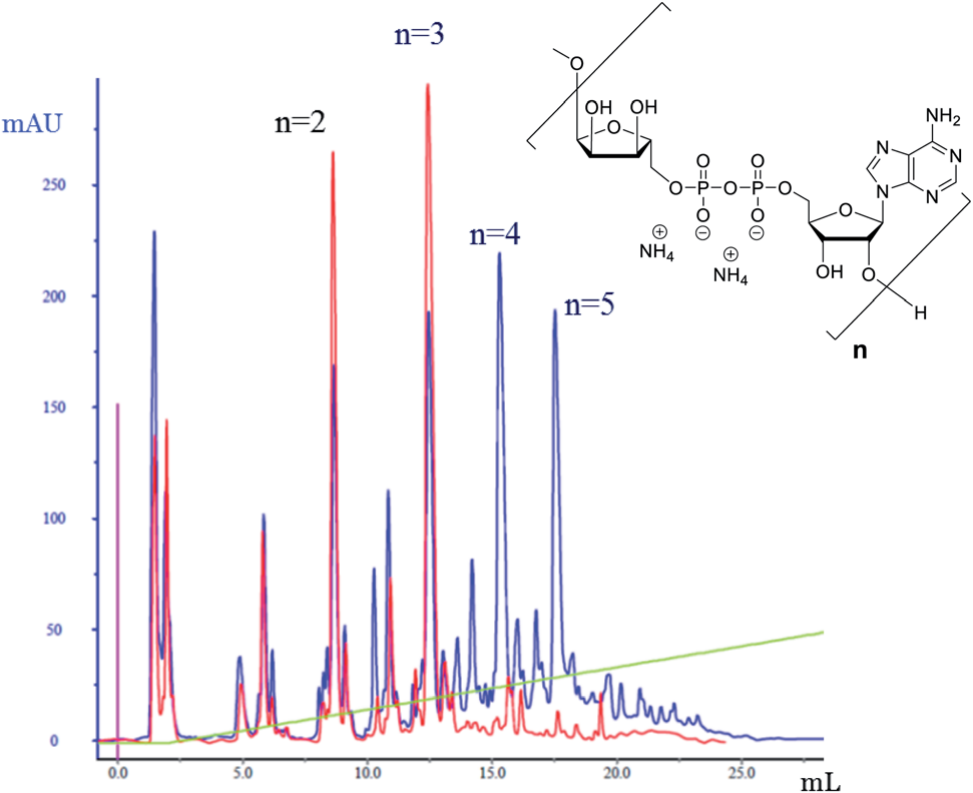
Analytical anion exchange chromatography traces (UV detection) for the crude sample of the synthesis of ADPr trimer (red) and pentamer (blue). Column used: DNAPac PA-100, 4 × 250 mm. Gradient applied: aqueous NH_4_OAc, 0–20% over 10 column volumes.

The successful manual synthesis of dimer **1a** was an incentive to explore the synthesis of ADPr oligomers using an automated DNA synthesizer. On the basis of the above experiment, TG resin was chosen for synthesis while other studies showed that 5-(benzylthio)-1*H*-tetrazole (BTT)^34,35^ is a better activator than ETT and an elongated DBU treatment time (10 min *×* 4) is necessary to completely cleave the Fm groups (Scheme 3). With these adaptations, an ADPr trimer was successfully synthesized on a DNA synthesizer (Fig. 3, red line) with 35% yield, which is higher than the procedure in which the acid labile *tBu* groups were used for phosphate monoester protection.^11^ Next, a more ambitious pentamer synthesis was performed using the same method. After cleavage from resin, the crude sample was analyzed by anion exchange chromatography showing the target pentamer peak together with tetramer and trimer peaks (Fig. 3, blue line). The formation of side-products and the shorter oligomers that are prominent in the crude reaction mixture may have originated from the incomplete couplings in cycle A. A partial cleavage of the already present immobilized pyrophosphates during the next coupling cycle cannot be excluded as well. Purification by anion exchange chromatography and gel filtration furnished the pure ADPr trimer **1b** (1.18 mg), tetramer **1c** (1.35 mg) and pentamer **1d** (0.69 mg), respectively. The structure of obtained ADPr oligomers (**1a–d**) was carefully characterized by NMR and high resolution mass spectrometry and the high purity of each compound was ascertained by RP-HPLC analysis (see ESI† for details). Next, the biophysical evaluation of synthetic oligo-ADPr could be undertaken.

**Fig. 4:**
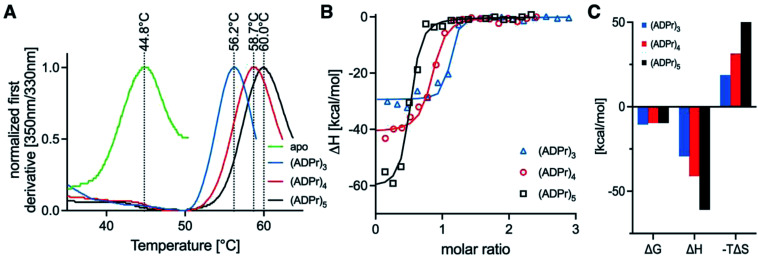
Binding profiles for tri-, tetra- and penta-ADPr to the recombinant, purified human ALC1 macrodomain module. (A) Tycho-thermal shift analysis of unliganded ALC1 macrodomain module (green line), as well as in the presence of tri-ADPr (blue line), tetra-ADPr (red line) and penta-ADPr (black line). (B) ITC assays for tri-ADPr (blue triangles), tetra-ADPr (red circles) and penta-ADPr (black squares). (C) Thermodynamic fitting parameters for the binding of tri-, tetra- and penta-ADPr to the ALC1 macrodomain module as determined by ITC.

To test whether the homogeneous tetra- and penta-ADPr fragments acted as biochemical ligands for the PAR-regulated human chromatin remodeller ALC1, we conducted thermal shift analyses and isothermal titration calorimetry (ITC) assays for the binding of tri-, tetra- and penta-ADPr with the recombinant, purified macrodomain module of the human ALC1 chromatin remodeler. The thermal shift assays (TSA) revealed a strong stabilization of the ALC1 macrodomain module by the three oligomeric ligands, increasing the denaturation temperature of the domain from 45 °C by 11 °C for tri-ADPr, 14 °C for tetra-ADPr and 15 °C for penta-ADPr (Fig. 4A). Thermodynamic analysis of the binding by ITC revealed a high affinity interaction between the protein and the oligomers, with equilibrium dissociation constants in the 20–100 nM range for tri-, tetra- and penta-ADPr (Fig. 4B and Table 1). Interestingly, both tetra- and penta-ADPr bound the macrodomain of ALC1 with very large enthalpies of binding of 41 and 61 kcal mol^−1^, respectively, compared to 29 kcal mol^−1^ for tri-ADPr (Fig. 4C). The *K*_D_ and Δ*H* we determined for tri-ADPr in the ITC assays is very similar to the previously reported values. What the new TSA and ITC data with tetra- and penta-ADPr show is that the ALC1 macrodomain module engages more extensively with the ADPr oligomers, as they are extended from three to five units in length. However, it is also clear that the biggest gain in binding enthalpy and affinity occurs when extending a monomer of ADPr to dimer and then (ADP-ribose)_3_, with the *K*_D_ changing from above detection limit, to 4 μM and then low nanomolar affinity. Our biophysical binding assays reveal a protein surface engineered to selectively recognize longer ADPr oligomers of ideally three or four ADPr-units in length. This is in sharp contrast to other ADPr-binding macrodomains, such as that of the human histone variant macroH2A.1.1, which bound monomeric and dimeric ADPr with the same affinity and enthalpy.^5^

**Table 1:** ITC thermodynamic parameters for the interaction of tri-, tetra- and penta-ADPr with the purified recombinant macrodomain module of the human ALC1 chromatin remodeler

Ligand	Temperature (°C)	[Syr] (M)	[Cell] (M)	N (sites)	*K*_D_ (M)	Δ*H* (kcal mol^−1^)	Δ*G* (kcal mol^−1^)	−*T*Δ*S* (kcal mol^−1^)
(ADPr)_3_	25.1	9.1 × 10^−5^	7.5 × 10^−6^	1.050	1.9 × 10^−8^	−29.3	−10.5	18.7
(ADPr)_4_	25.1	9.4 × 10^−5^	7.5 × 10^−6^	0.805	9.3 × 10^−8^	−41.0	−9.59	31.4
(ADPr)_5_	25.1	11.3 × 10^−5^	7.5 × 10^−6^	0.478	9.6 × 10^−8^	−60.9	−9.57	51.4

Moreover, as we increased ADPr length from three to five units in our ITC assays, we observed that the apparent stoichiometry of fitting for the tetra- and penta-ADPr oligomers in the ITC binding assay to the recombinant ALC1 module decreased from an observed ∼1.0 for tri-ADP-ribose, to ∼0.81 for tetra-ADPr and ∼0.5 for penta-ADPr (Fig. 4B). The stoichiometries were close to 1.0 for both di-ADPr and tri-ADPr also in our previous ITC analysis of the ALC1 macrodomain.^5^ These parameters suggest that a tetramer or pentamer of ADPr may be recognized by more than one ALC1 macrodomain module at the same time, at least *in vitro*. This could also occur *in vivo* upon PARP1/PARP2 activation during DNA damage, for example, since PAR is a branched nucleic acid and the ALC1 macrodomain is expected to recognize multiple 2′-OH ends of the branched PAR ‘tree’. However, testing *in vitro* such a multivalent mode of interaction is experimentally challenging, would require orthogonal methods for validation and might ultimately not fully recapitulate how ALC1 is regulated by PARP1-/PARP2-mediated PARylation at or near DNA damage sites and transcriptionally active promoters.

## Conclusion

An improved solid phase procedure towards linear ADPr oligomers is described. An advanced Fm protected phosphoramidite building block **3** was designed and synthesized in a good overall yield and sufficient quantity to attain the synthetic ADPr oligomers of unprecedented length. The resins LCAA-CPG **2m** and TentaGel N **2k** were evaluated, to which the phosphoribosyl residue was attached with the ester-based Q-linker in line with the alkali sensitive semi-permanent protective groups in the building blocks **3** and **4**. Due to its relatively high loading capacity and the ease of handling the TentaGel resin allowed a convenient preparation of well-defined oligo-ADPr fragments up to a pentamer in quantities ranging from submilligram to milligram. The presented solid-phase approach, based on the introduction of pyrophosphate linkages with P(v)–P(iii) chemistry allows, in principle, for the future assembly of ADPr oligomers functionalized with fluorescent labels or ligation handles such as terminal alkynes and strained alkenes.

We used the obtained oligomers of ADPr for a binding study with the ALC1 macrodomain module and found that longer ADPr oligomers exhibited tighter binding. Further, the magnitude of the binding enthalpy increased considerably when the length of the ADPr oligomer was enlarged from tri-, tetra- to penta-ADPr (29, 41 and 61 kcal mol^−1^, respectively). In comparison, we showed earlier that di-ADP-ribose bound with an enthalpy of approximately 20 kcal mol^−1^, while mono-ADPr failed to bind the ALC1 macrodomain module with measurable affinity (*K*_D_ > 50 μM).^5^ Our new experiments thus showed that the chromatin remodeler ALC1 has a high affinity for oligomers of ADPr and that it interacted with oligomers of at least 3 ADPr units more tightly and extensively. Further analysis using ultracentrifugation, orthogonal biochemical techniques, high-resolution structural and cellular approaches will be necessary to validate whether the apparent decrease in binding stoichiometry observed for both tetra- and penta-ADPr (Fig. 4B) represents the binding of more than one ALC1 macrodomain module with these longer oligomers of ADPr. Clearly, our results showed that tri-, tetra- and penta-ADPr are recognized by the PAR-regulated human chromatin remodeller ALC1 with high affinity and enthalpies for binding. ALC1's ability to engage with longer ADPr oligomers potently make it an ideal, sensitive responder to PARP1/2 activation. Its distinct macrodomain engenders a tightly controlled enzymatic gating function for PAR. Moreover, exciting recent work indicates that factors such as HPF1 can switch the *in vitro* activity of PARP1 from a polymerase to hydrolase.^36^ One impact of this is that the average length of the ADPr oligomer is severely reduced, potentially down to mono-ADP-ribosylation in combination with PARG activity.^37^ Based on our current and previous data,^5^ we can propose that ALC1 activity would be exquisitely sensitive to all cellular events that result in dynamic changes to the balance of mono-, di-, oligo- and poly-ADPr on chromatin. This might allow ALC1 to be activated only in specific biological contexts, where PARylation prevails over the MARylation activities of the poly-ART enzymes PARP1 and PARP2.

## Data availability

The ESI† include the detailed experimental procedures, LC-MS data and NMR data.

## Author contributions

Q. L., J. V., N. J. M. developed and performed the synthesis of oligo-ADP-ribose. D. V. F. and G. A. v. d. M. jointly designed the synthesis, supervised and coordinated the synthetic work. G. K. purified the protein and performed the biophysical experiments with ALC1 and oligo-ADP-ribose. A. G. L. designed and supervised the biophysics experiments. D. V. F., A. G. L., G. A. v. d. M. and H. S. O. conceived and conceptualised the study. Q. L., D. V. F., A. G. L., G. A. v. d. M. and H. S. O. wrote the article with the contributions from all other authors. All authors read and approved the final manuscript.

## Conflicts of interest

A. G. L. is a founder, CSO and shareholder of Eisbach Bio GmbH.

## Supplementary Material

SC-012-D1SC02340C-s001

SC-012-D1SC02340C-s002
