# Supplementary material for: Chemical synthesis of linear ADP-ribose oligomers up to pentamer and their binding to the oncogenic helicase ALC1
Source: Chem Sci. 2021 Aug 18;12(37):12468–75. doi: 10.1039/d1sc02340c (PMC8480336; doi:10.1039/d1sc02340c)

Electronic Supplementary Material (ESI) for Chemical Science.
This journal is © The Royal Society of Chemistry 2021

Chemical Synthesis of Linear ADP-ribose Oligomers up to Pentamer and their Binding to the Oncogenic Helicase ALC1

Qiang Liu,[a#] Gunnar Knobloch,[b§] Jim Voorneveld,[a] Nico J. Meeuwenoord,[a] Herman S. Overkleeft,[a] Gijsbert A. van der Marel,[a] Andreas G. Ladurner*[bc] and Dmitri V. Filippov*[a]

*Corresponding authors
[a]Leiden Institute of Chemistry, Leiden University, P.O. Box 9502, 2300 RA Leiden, The Netherlands, filippov@chem.leidenuniv.nl
[b]Biomedical Center (BMC), Physiological Chemistry, Faculty of Medicine, LMU Munich, 82152 Planegg-Martinsried, Germany, andreas.ladurner@bmc.med.lmu.de
[c]Eisbach Bio GmbH, Am Klopferspitz 19, 82152 Planegg-Martinsried, Germany
[#]Current address: Complex Carbohydrate Research Center, University of Georgia, 315 Riverbend Road, Athens, GA, 30602-4712, United States
[§]Current address: Eisbach Bio GmbH, Am Klopferspitz 19, 82152 Planegg-Martinsried, Germany

Supplementary Information

Table of Contents

Page

Experimental procedures chemistry--02

References---15

UV spectra of LC-MS analysis of compound **1a-d**--16

HRMS analysis of compound **1a-d**--18

Materials and methods biology--20

^{1}H, ^{13}C and ^{31}P-NMR spectra of all compounds---22

Experimental procedures chemistry

All solvents used were stored over molecular sieves and all reactions were carried out in oven or flame-dried glassware. Unless stated otherwise, all solvents were removed by rotary evaporation under reduced pressure at 40°C. Reactions were monitored by TLC-analysis using Merk 25 DC plastikfolien 60 F_{254} with detection by spraying with 20% H_2SO_4 in MeOH or $(NH_4)_6Mo_7O_{24}\cdot 4H_2O$ (25g/L) and $(NH_4)_4Ce(SO_4)_4\cdot 2H_2O$ in 10% sulfuric acid, followed by charring at approx. 150°C. LC-MS analysis was performed on a Thermo Finnigan LCQ Advantage MAX ion-trap mass spectrometer with an electrospray ion source coupled to Surveyor HPLC system (Thermo Finnegan) using an analytical Gemini C18 column (Phenomex, 50 x 4.60 mm, 3 micron) in combination with eluents A: H_2O; B: MeCN and C: 1% aq. TFA as the solvent system. High resolution mass spectra were recorded by direct injection (2 µL of a 2 µM solution in water/acetonitrile; 50/50; v/v and 0.1% formic acid) on a mass spectrometer (Thermo Finnigan LTQ Orbitrap) equipped with an electrospray ion source in positive mode with resolution R = 60000 at m/z 400 (mass range m/z = 150-2000) and dioctylpthalate (m/z = 391.2842) as a “lock mass”. The high-resolution mass spectrometer was calibrated prior to measurements with a calibration mixture (Thermo Finnigan). ^{1}H-, ^{13}C- and ^{31}P-NMR spectra were measured on Brüker DPX-300, Brüker AV-400/500/600/850 and all individual signal was assigned using 2D-NMR spectroscopy. Chemical shifts were given in ppm (δ) relative to TMS (0 ppm) or indirectly referenced to H_3PO_4 (0.00 ppm) in D_2O via the solvent residual signal and coupling constants were given in Hz. Infrared (IR) spectra were record on a Shimadzu FT-IR 8300. Optical rotation was measured by MCP 100 Modular Circular Polarimeter using methanol as solvent. LCAA-CPG resin were purchased from Sigma-Aldrich. Tentagel N resin was purchased from Rapp Polymere (Product name: TentaGel® N NH_2, product number: N30002).

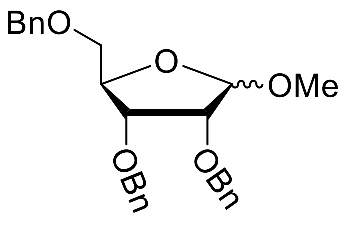

1-*O*-Methyl-2,3,5-tris-*O*-benzyl-αβ-D-ribofuranoside (5)

D-Ribose(5 g, 33.30 mmol), methanol (120 mL) and acyl chloride (0.62 mL, 10.99 mmol) were added into a flask and the reaction was stirred at room temperature for 5 h after which was quenched by $NaHCO_3$ (6 g). The mixture was filtered and concentrated. The residue was then co-evaporated with toluene (3 x) and DMF (160 mL) was added into the flask. The mixture was cooled down to 0°C then NaH (5.4 g, 166.5 mmol, 60% in mineral oil) was added. After gas generation was ceased, BnBr (15 mL, 166.5 mmol) was added in 3 portions over 10 min. The mixture was allowed to warm up to room temperature carefully and stirred for 16 h. MeOH (15 mL) was added to quench the reaction and H_2O and EtOAc were added. The water layer was washed with EtOAc then

all the organic layers were combined and dried ($MgSO_4$). The mixture was filtered, concentrated and purified by silica gel column chromatography (pentane/EtOAc, 19/1 – 2/1) to obtain **5** as a colorless oil (14.5 g, 33.30 mmol, 100%). Spectroscopic data was identical with the reported same compound.[1] ^{1}H NMR (500 MHz, Chloroform-*d*) δ 7.44 – 7.12 (m, 20H, arom. αβ), 4.91 (d, *J* = 1.2 Hz, 1H, H1-β), 4.87 (d, *J* = 4.3 Hz, 0.3H, H1-α), 4.69 – 4.39 (m, 8H, CH_2-Bn-αβ), 4.35 (ddd, *J* = 7.1, 5.8, 3.7 Hz, 1H, H4-β), 4.27 – 4.21 (m, 0.3H, H4-α), 4.02 (dd, *J* = 7.1, 4.7 Hz, 1H, H3-β), 3.86 – 3.80 (m, 1.3H, H2-β, H3-α), 3.77 (dd, *J* = 6.8, 4.3 Hz, 0.3H, H2-α), 3.60 (AB, *J* = 10.6, 3.8 Hz, 1H, H5-β), 3.51 (AB, *J* = 10.6, 5.8 Hz, 1H, H5-β), 3.46 (s, 1H, OMe-α), 3.40 (AB, *J* = 10.4, 4.1 Hz, 0.3H, H5-α), 3.34 (AB, *J* = 10.4, 4.2 Hz, 0.3H, H5-α), 3.30 (s, 3H, OMe-β). ^{13}C NMR (126 MHz, $CDCl_3$) δ 138.37, 138.31, 137.98, 137.89, 137.87 (Cq. arom. αβ), 128.44, 128.41, 128.39, 128.35, 128.34, 128.31, 128.05, 127.99, 127.95, 127.91, 127.84, 127.80, 127.71, 127.69, 127.67, 127.63, 127.61, 127.54 (arom. αβ), 106.40 (C1-β), 102.53 (C1-α), 82.15(C4-α), 80.51 (C4-β), 79.75 (C2-β), 78.44 (C3-β), 77.86 (C2-α), 75.03 (C3-α), 73.48, 73.19, 72.47, 72.45, 72.35, 72.34 (CH_2 Bn-αβ), 71.37 (C5-β), 70.19 (C5-α), 55.57 (OMe-α), 55.09 (OMe-β).

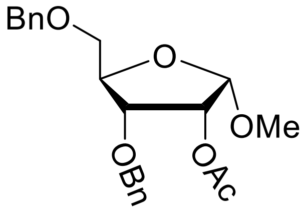

1-*O*-Methyl-2-*O*-acetyl-3,5-di-*O*-benzyl-α-ᴅ-ribofuranoside (6)

Compound **5** (8.3 g, 19.10 mmol) and DCM (95 mL) were added into a flask after which the solution was cooled down to 0°C. $SnCl_4$ (19.1 mL, 19.1 mmol, 1M solution in DCM) was added to the reaction and the mixture was stirred at 4 °C for 16 h. The reaction was quenched by aq. $NaHCO_3$ (sat.) and filtered. The organic filtration was washed by H_2O (1 x), brine (1 x) and dried ($MgSO_4$). The mixture was filtered, concentrated and co-evaporated with toluene (3 x). The residue was re-dissolved in pyridine (95 mL), added DMAP (117 mg, 0.96 mmol) and acetic anhydride (18.0 mL, 191.0 mmol). The reaction was stirred at room temperature for 3 h after which was quenched by aq. $NaHCO_3$ (sat.). EtOAc was added to extract the mixture and the organic layer was further washed by H_2O (1 x) and brine (1 x). The organic layer was dried ($MgSO_4$), filtered and concentrated. The residue was purified by silica gel column chromatography (pentane/EtOAc, 95/5 – 80/20 – 70/30) to obtain **6** as a colorless oil (6.4 g, 16.57 mmol, 87%). ^{1}H NMR (500 MHz, Chloroform-d) δ 7.40 – 7.22 (m, 10H), 5.12 (d, *J* = 4.5 Hz, 1H, H1), 4.91 (dd, *J* = 7.1, 4.5 Hz, 1H, H2), 4.68 (d, *J* = 12.4 Hz, 1H, C*H*H Bn), 4.53 (d, *J* = 12.1 Hz, 1H, C*H*H Bn), 4.47 (dd, *J* = 12.3, 10.4 Hz, 2H, 2xC*H*H Bn), 4.21 (d, J = 4.0 Hz, 1H, H4), 4.04 (dd, *J* = 7.1, 4.2 Hz, 1H, H3), 3.51 – 3.43 (m, 4H, OMe, H5), 3.33 (AB, *J* = 10.5, 4.2 Hz, 1H, H5), 2.20 (s, 3H, Ac). ^{13}C NMR (126 MHz, $CDCl_3$) δ 170.22 (CO Ac), 137.75, 137.74 (Cq. arom.), 128.21, 128.17, 127.97, 127.67, 127.50 (arom.), 101.64 (C1), 81.36 (C4), 75.01 (C3), 73.23, 72.90 (CH_2 Bn), 71.99 (C2), 69.25 (C5), 55.40 (OMe), 20.61 (Me Ac). IR (film): 2928, 1740, 1453, 1372, 1238, 1124, 1096, 1065, 1027, 739, 698 cm^{-1}. HRMS (ESI^+) calcd for $C_{22}H_{26}O_6Na$ (M+Na) 409.1622. Found 409.1622. $[\alpha]_D^{20}$ +95.0 (c = 1, in DCM)

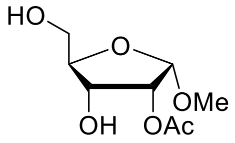

7A

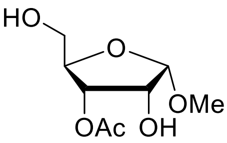

7B

1-*O*-Methyl-2-*O*-acetyl-α-ᴅ-ribofuranoside (7A)/

1-*O*-Methyl-3-*O*-acetyl-α-ᴅ-ribofuranoside (7B)

Compound **6** (1.24 g, 3.21 mmol) was dissolved in *t*BuOH/Dioxane/H_2O (12 mL, 4/4/1; v/v/v). Pd/C (124 mg, 10 wt % Pd) were added and H_2 was bubbled through the mixture for 24 h at room temperature after which the reaction mixture was filtered over celite. The filtration was concentrated under reduced pressure and co-evaporated with toluene (1 x). The residue was purified by silica gel column chromatography (DCM/methanol, 100/2 – 100/5) to obtain **7** as a colorless oil (615 mg, 2.98 mmol, 93%). (80% 2-OAC product and 20% 3-OAc product) ^{1}H NMR (500 MHz, Chloroform-*d*) δ 5.10 (d, *J* = 4.1 Hz, 1H, H1-A), 4.96 (dd, *J* = 7.2, 3.3 Hz, 0.25H, H3-B), 4.92 (d, *J* = 4.6 Hz, 0.25H, H1-B), 4.75 (dd, *J* = 6.3, 4.1 Hz, 1H, H2-A), 4.19 (td, *J* = 7.2, 6.8, 3.5 Hz, 1H, H3-AB), 4.13 (td, *J* = 3.6, 2.4 Hz, 1H, H4-A), 4.05 (q, *J* = 3.4 Hz, 0.25H, H4-B), 3.81 – 3.73 (m, 1.5H, H5-A, H5-B), 3.69 (AB, *J* = 11.5, 3.4 Hz, 1H, H5-A), 3.45 (s, 0.75H, OMe-B), 3.41 (s, 3H, OMe-A), 2.84 (d, *J* = 9.6 Hz, 1H, 3-OH-A), 2.74 (d, *J* = 10.8 Hz, 0.25H, 3-OH-B), 2.40 (bs, 1.25H, 5-OH-AB), 2.15 (s, 3H, Ac-A), 2.11 (s, 0.75H, Ac-B). ^{13}C NMR (126 MHz, $CDCl_3$) δ 170.92, 170.47 (CO, Ac), 102.57 (C1-B), 101.96 (C1-A), 86.03 (C4-A), 83.07 (C4-B), 73.24 (C2-A), 71.49 (C3-B), 71.35 (C2-B), 70.04 (C3-A), 62.65 (C5-A), 62.51 (C5-B), 55.57 (OMe-B), 55.37 (OMe-A), 21.00 (CH_3 Ac-B), 20.77 (CH_3 Ac-A). IR (film): 3444, 2932, 1735, 1374, 1234, 1081, 1028, 964, 899, 5002, 607, 479 cm^{-1}. HRMS (ESI^{+}) calcd for $C_8H_{14}O_6Na$ (M+Na) 229.0683. Found 229.0685. $[\alpha]_D^{20}$ +112.3 (c = 1, in DCM)

1-*O*-Methyl-2-*O*-acetyl-5-O-(4,4'-di-methoxyltrityl)-α-ᴅ-ribofuranoside (8A) /

1-*O*-Methyl-3-*O*-acetyl-5-O-(4,4'-di-methoxyltrityl)-α-ᴅ-ribofuranoside (8B)

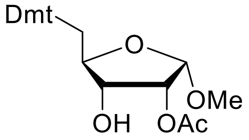

8A

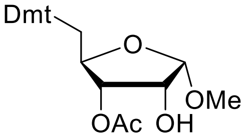

8B

Compound **7** (584 mg, 2.83 mmol), 4,4'-dimethoxyltrityl chloride (DmtCl, 1.01 g, 2.98 mmol), and pyridine were added into a flask and the solution was stirred for 16 h at room temperature after which was concentrated. The residue was dissolved in EtOAc and washed by aq. $NaHCO_3$ (sat.). The organic layer was dried ($MgSO_4$), filtered and concentrated. The residue was purified by silica gel column chromatography (pentane/EtOAc, 100/0 – 90/10 – 80/20 – 60/40) to obtain **8** as a light-yellow foam (1.31 g, 2.58 mmol, 91%). ^{1}H NMR (400 MHz, Chloroform-*d*) δ 7.46 – 7.42 (m, 2H, DMT arom.), 7.38 – 7.16 (m, 7H, DMT arom.), 6.88 – 6.80 (m, 4H, DMT arom.), 5.22 (d, *J* = 4.1 Hz, 0.25H, H1-B), 5.16 (dd, *J* = 7.0, 2.8 Hz, 0.75H, H3-A), 5.06 – 5.04 (m, 1H, H1-A, H2-B), 4.44 (dd, *J* = 7.0, 4.7 Hz, 0.75H, H2-A), 4.27 – 4.24 (m, 0.5H, H3-B, H4-B), 4.16 (q, *J* = 3.3 Hz, 0.75H, H4-A), 3.79 (s, 6H, OMe DMT), 3.51 (s, 2.25H, OMe-A), 3.47 (s, 0.75H, OMe-B), 3.39 – 3.33 (m, 1H, H5), 3.25 – 3.16 (m, 1H, H5), 2.72 (s, 1H, OH), 2.20 (s, 0.75H, Ac-B), 2.10 (s, 2.25H, Ac-A). ^{13}C NMR (101 MHz, $CDCl_3$) δ 170.44(CO Ac-A), 170.30 (CO Ac-B),

158.55, 144.83, 144.69, 136.03, 135.96, 135.87, 135.76 (Cq. arom.), 130.40, 130.13, 128.22, 127.91, 126.87, 113.21 (arom.), 102.59 (C1-A), 101.94(C1-B), 86.27, 86.26 (Cq. DMT), 85.50 (C4-B), 81.97 (C4-A), 73.34 (C2-B), 72.08 (C3-A), 71.42 (C2-A), 70.73 (C3-B), 63.68 (C5-A), 63.67 (C5-B), 55.70 (OMe), 55.34 (OMe DMT-B), 55.25 (OMe DMT-A), 21.01 (CH_3 Ac-A), 20.85 (CH_3 Ac-B). IR (film): 3507, 2933, 2837, 1741, 1608, 1509, 1446, 1300, 1248, 1177, 1077, 1035, 830, 596 cm^{-1}. HRMS (ESI^+) calcd for $C_{29}H_{32}O_8Na$ (M+Na) 531.1989. Found 531.1992. $[\alpha]_D^{20}$ +53.1 (c = 1, in DCM)

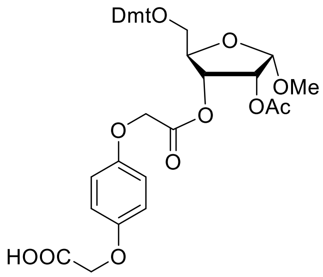

10A

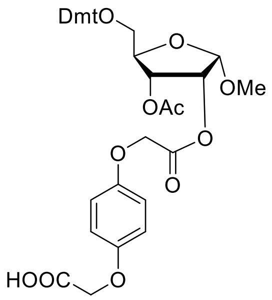

10B

1-*O*-Methyl-2-*O*-acetyl-3-*O*-hydroquinone-*O,O'*-diacetylhemiester-5-O-(4,4'-di-methoxyltrityl)-α-ᴅ-ribofuranoside (10A) / 1-*O*-Methyl-2-*O*-hydroquinone-*O,O'*-diacetylhemiester-5-*O*-(4,4'-di-methoxyltrityl)-3-*O*-acetyl-α-ᴅ-ribofuranoside (10B)

Compound **8** (1.22 g, 2.40 mmol) was dissolved in pyridine (12 mL). DMAP (29 mg, 0.24 mmol), EDC (446 mg, 2.88 mmol), Et_3N (0.24 mL, 1.73 mmol) and hydroquinone-O,O'-diacetic acid (Q-linker, **9**) (650 mg, 2.88 mmol) were added and the reaction was stirred at room temperature for 16 h. The reaction mixture was concentrated, diluted with $CHCl_3$ and washed with H_2O. The water layer was extracted with $CHCl_3$ and the combined organic layers were dried ($MgSO_4$), concentrated and purified by silica gel chromatography neutralized with 1% Et_3N (DCM/methanol, 100/0 – 99/1 – 95/5 – 90/10) to obtain **10** as a white foam (962 mg, 1.34 mmol, 56%). ^1H NMR (500 MHz, Chloroform-*d*) δ 7.38 – 7.32 (m, 2H, arom.), 7.27 – 7.18 (m, 6H, arom.), 7.17 – 7.10 (m, 1H, arom.), 6.85 – 6.73 (m, 8H, arom.), 5.35 (dq, *J* = 7.5, 2.6 Hz, 1/3H, H3-B), 5.28 – 5.22 (m, 2/3H, H3-A), 5.19 (dd, *J* = 5.4, 3.7 Hz, 4/3H, H1-A, H2-A), 5.17 – 5.12 (m, 2/3H, H1-B, H2-B), 4.63 – 4.48 (m, 2H, CO*CH₂*O), 4.39 – 4.37 (m, 2H, *CH₂*COOH), 4.15 (q, *J* = 3.4 Hz, 1H, H4), 3.72 – 3.71 (m, 6H, OMe DMT), 3.38 (s, 3H, OMe), 3.33 – 3.29 (m, 1H, H5), 3.20 – 3.13 (m, 1H, H5), 2.01 (s, 1H, CH_3 Ac-B), 1.97 (s, 2H, CH_3 Ac-A). ^{13}C NMR (126 MHz, $CDCl_3$) δ 174.30(COOH-A), 174.28 (COOH-B), 170.49 (CO Ac-A), 170.03(CO Ac-B), 168.82(CH_2*CO*O-B), 168.45(CH_2*CO*O-A), 158.66, 158.65, 153.83, 152.15, 152.13, 144.75, 136.00, 135.96, 135.85, 135.78 (Cq. arom.), 130.21, 130.19, 128.29, 128.01, 126.98, 126.97, 116.00, 115.91, 115.71, 113.31 (arom.), 101.66 (C1-B), 101.64 (C1-A), 86.47 (Cq. DMT-B), 86.43(Cq. DMT-A), 81.21 (C4-B), 81.03 (C4-A), 71.78 (C2-A), 71.64 (C3-B), 71.42 (C2-B), 70.60 (C3-A), 67.62 (CH_2COOH), 66.25 (CH_2COO-B), 66.05 (CH_2COO-A), 63.45 (C5-B), 63.41 (C5-A), 55.88 (OMe-A), 55.76 (OMe-B), 55.36 (OMe DMT), 20.93 (CH_3 Ac-A), 20.75 (CH_3 Ac-B). IR (film): 2934, 1738, 1607, 1507, 1445, 1246, 1178, 1073, 1032, 828cm^{-1}. HRMS (ESI^+) calcd for $C_{39}H_{40}O_{13}Na$ (M+Na) 739.2361. Found 739.2364. $[\alpha]_D^{20}$ +33.0 (c = 1, in DCM)

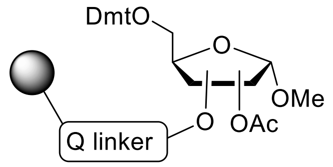

12m (CPG)
12k (TG)

1-*O*-Methyl-2-*O*-acetyl-3-*O*-Q-CPG-5-*O*-(4,4'-di-methoxyltrityl)-α-ribofuranoside/ 1-*O*-Methyl-2-*O*-Q-CPG-3-*O*-acetyl-5-*O*-(4,4'-di-methoxyltrityl)-α-ᴅ-ribofuranoside (12m, CPG)

1-*O*-Methyl-2-*O*-acetyl-3-*O*-Q-TG-5-*O*-(4,4'-di-methoxyltrityl)-α-ribofuranoside/ 1-*O*-Methyl-2-*O*-Q-TG-3-*O*-acetyl-5-*O*-(4,4'-di-methoxyltrityl)-α-ᴅ-ribofuranoside (12k, TG)

To a 20 mL reaction syringe with filter frit was added LCAA-CPG (**11m**, 3 g, 0.20 mmol) or Tentagel N NH_2 (**11k**, 800 mg, 0.20 mmol), MeCN (12 mL), compound **10** (430 mg, 0.6 mmol), HOBT (12 mg, 0.08 mmol), DIC (0.28 mL, 1.8 mmol) and DIPEA (0.52 mL, 3 mmol). The mixture was shaken at room temperature for 16 h. The reaction mixture was drained and the CPG/Tentagel was washed with ACN (2 x), DMF (2 x) and DCM (3 x) under N_2. The remaining unmodified amine groups were capped by adding a mixture of CAP 1 (6 mL) and CAP 2 (6 mL). The mixture was shaken for 2 h, drained and washed with DMF (3 x) and DCM (3 x) under N_2. The CPG/Tentagel was dried under reduced pressure and the loading was determined by trityl analysis at 503 nm. The loadings for **12m** (CPG) and **12k** (TG) were 50 µmol/g and 207 µmmol/g respectively.

1-*O*-Methyl-2-*O*-acetyl-3-*O*-Q-CPG-5-*O*-(di-*O*-fluorenylmethylphosphoryl)-α-ᴅ-ribofuranoside/ 1-*O*-Methyl-2-*O*-Q-CPG-3-*O*-acetyl-5-*O*-(di-*O*-fluorenylmethylphosphoryl)-α-ᴅ-ribofuranoside (2m, CPG)

1-*O*-Methyl-2-*O*-acetyl-3-*O*-Q-TG-5-*O*-(di-*O*-fluorenylmethylphosphoryl)-α-ᴅ-ribofuranoside/ 1-*O*-Methyl-2-*O*-Q-TG-3-*O*-acetyl-5-*O*-(di-*O*-fluorenylmethylphosphoryl)-α-ᴅ-ribofuranoside (2k, TG)

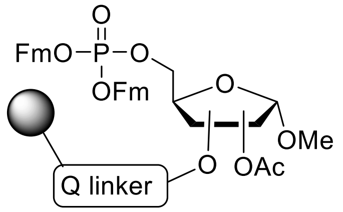

2m (CPG)
2k (TG)

To a 20 mL reaction syringe with filter frit was added **12m** (3 g) or **12k** (900 mg). Dichloroacetic acid (5 %, v/v, in DCM) was added repeatedly until no yellow color was observed. The resin was extensively washed with DCM (3 x), ACN (5 x) under N_2. ETT (0.25M in ACN, 12 eq) and **13** (0.2 M in ACN, 4 eq) were added into the resin and the mixture was shaken under N_2 for 10 min and drained. Repeat this coupling once and the resin was drained and washed with ACN (5 x). 9 mL (1*S*)-(+)-(10-camphorsulfonyl)-oxaziridine (CSO) (0.5 M in ACN) was added and the mixture was shaken for 30 min under N_2. The resin was drained, washed with ACN (5 x) under N_2 and dried under reduced pressure to obtain **2m** or **2k** which was stored at 4°C before use. A test sample of **2m** (60 mg) or **2k** (20 mg) was added into a 2 mL reaction syringe. To this syringe was added 1 mL DBU solution (10%, v/v, in ACN) and was shaken for 10 min to remove Fm groups on 5-phosphate after which was drained and washed with ACN (3 x). The resin was treated with 1 mL NH_4OH (35%) for 1 h to cleave the product and filtered off. The filtrate was concentrated and analyzed by NMR.

^{31}P NMR (162 MHz, D_2O): **2m** δ 4.58 (s); **2k** δ 4.57 (s)

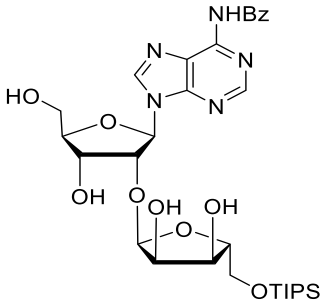

N^6-benzoyl-9-(5-*O*-triisopropylsilyl-β-parobiosyl) adenine (15)

To a round-bottom flask, compound **14** (2.48 g, 2.63 mmol), pyridine (18 mL) and EtOH (9 mL) were added in sequence. The mixture was cooled to 0 °C after which aqueous NaOH (15.78 mL, 1 M solution) was slowly added. The reaction was stirred for 1 h at 0 °C after which Amberlite-H^+ was added until pH = 6. The mixture was filtered, concentrated and purified by silica gel column chromatography (DCM/MeOH, 100/0 – 100/3 – 100/5) to obtain **15** as a white foam (1.66 g, 2.52 mmol, 96%). ^{1}H NMR (500 MHz, Chloroform-*d*) δ 9.69 (s, 1H, NH), 8.69 (s, 1H, H2), 8.32 (s, 1H, H8), 8.00 (d, *J* = 7.1 Hz, 2H, arom), 7.57 (t, *J* = 7.4 Hz, 1H, arom.), 7.47 (t, *J* = 7.7 Hz, 2H, arom.), 6.11 (d, *J* = 7.0 Hz, 1H, H1'), 5.78 (brs, 1H, OH), 5.01 (d, *J* = 4.1 Hz, 1H, H1''), 4.95 (dd, *J* = 7.1, 4.6 Hz, 1H, H2'), 4.76-4.41 (m, 4H, H3', OH x 3), 4.29 (s, 1H, H4'), 4.23-4.19 (m, 3H, H2'', H3'', H4''), 3.93-3.67 (m, 4H, H5', H5''), 0.98-0.97 (m, 21H, TIPS). ^{13}C NMR (126 MHz, $CDCl_3$) δ 165.29 (CO, Bz), 150.84, 150.20, 133.44 (Cq. arom.), 133.04, 128.88, 128.23 (arom.), 124.07 (cq, arom.), 101.73 (C1''), 89.09 (C1'), 87.81 (C4'), 86.67 (C4''), 79.39 (C2'), 73.35 (C2''), 72.69 (C3'), 71.72 (C3''), 63.95 (C5''), 63.06 (C5'), 18.03, 18.01, 11.92 (TIPS). IR (film): 3337, 2942, 2866, 1704, 1614, 1584, 1459, 1252, 1093, 1042, 883, 709, 686 cm^{-1}. HRMS (ESI$^+$) calcd for $C_{31}H_{46}N_5O_9Si$ (M+H) 660.3059. Found 660.3061. $[\alpha]_D^{20}$ +5.2 (c = 1, in DCM)

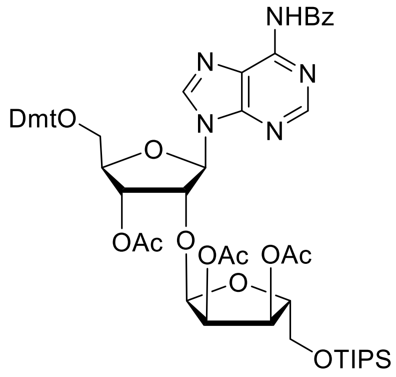

N^6-benzoyl-9-(3',2'',3''-tris-*O*-acetyl-5'-*O*-dimethoxyltrityl-5''-*O*-triisopropylsilyl-β-parobiosyl) adenine (16)

Compound **15** (1.66 g, 2.52 mmol) was co-evaporated with pyridine (1 x), then N_2 was applied. Dry pyridine (12 mL) and 4,4'-dimethoxyltrityl chloride (DmtCl, 1.36 g, 4.01 mmol) was added into the flask. 40 min later, TLC showed complete conversion and the mixture was cool to 0 °C after which Ac_2O (1.42 mL, 15 mmol) was added. The mixture was stirred at 0 °C for 5 h and was quenched by aq. $NaHCO_3$ (sat.). DCM extracted (3 x) the mixture and the organic layers were combined and dried ($MgSO_4$). The mixture was filtered, concentrated and purified by silica gel column chromatography (pentane/acetone, 100/0 – 90/10 – 85/15 – 80/20 – 70/30) to obtain **16** as a white foam (2.50 g, 2.30 mmol, 91%). ^{1}H NMR (500 MHz, Chloroform-*d*) δ 9.37 (s, 1H, NH), 8.70 (s, 1H, H2), 8.17 (s, 1H, H8), 8.01(d, *J* = 7.3 Hz, 2H, arom.), 7.58 – 7.51 (m, 1H, arom. Bz), 7.47 – 7.43 (m, 4H, arom. Bz, DMT), 7.33 – 7.19 (m, 7H, Dmt), 6.81 (d, *J* = 9 Hz, 4H, arom. Dmt), 6.26 (d, *J* = 6.0 Hz, 1H, H1'), 5.62 (dd, *J* = 5.2, 3.6 Hz, 1H, H3'), 5.38 (dd, *J* = 7.0, 2.7 Hz, 1H, H3''), 5.35 (d, *J* = 4.7 Hz, 1H, H1''), 5.26 (t, *J* = 5.6 Hz, 1H, H2'), 4.86 (dd, *J* = 7.0, 4.6 Hz, 1H, H2''), 4.35 (q, *J* = 3.5 Hz, 1H, H4'), 4.15 (q, *J* = 2.8 Hz, 1H,

H4''), 3.86 (AB, ddd, *J* = 11.0, 2.9 Hz, 2H, H5''), 3.76 – 3.75 (m, 6H, CH3, DMT), 3.52 (AB, *J* = 10.6, 3.5 Hz, 2H, H5'), 2.16 (s, 3H, Ac), 2.11 (s, 3H, Ac), 1.84 (s, 3H, Ac), 1.07 – 1.03 (m, 21H, TIPS). ^{13}C NMR (126 MHz, $CDCl_3$) δ 170.13, 169.49, 169.40 (CO, Ac), 164.65 (CO, Bz), 158.57 (Cq. arom.), 152.68 (CH, C2), 151.84 (C4), 149.63 (C6), 144.26 (Cq. arom.), 141.38 (CH, C8), 135.26 (Cq. arom.), 133.59, 132.61, 130.04, 128.68, 128.10, 127.84, 127.82, 126.98 (arom.), 123.41 (C5), 113.15 (Cq. arom.), 101.23 (C1''), 86.83 (Cq, DMT), 86.34 (C1'), 83.24 (C4''), 82.49 (C4'), 77.90 (C2'), 72.20 (C3'), 71.45 (C2''), 70.06 (C3''), 63.10 (C5''), 62.86 (C5'), 55.09 (CH_3, DMT), 20.77, 20.73, 20.10 (CH3, Ac), 17.79, 17.76, 11.76 (TIPS).

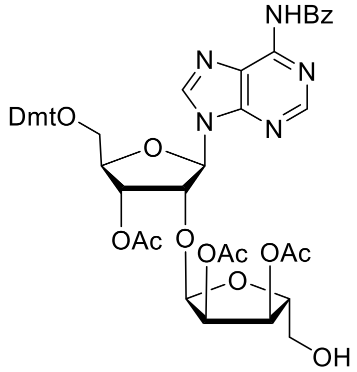

N^6-benzoyl-9-(3',2'',3''-tris-*O*-acetyl-5'-*O*-dimethoxyltrityl-β-parobiosyl) adenine (17)

Compound **16** (2.50 g, 2.30 mmol), dry THF (23 mL) and TBAF (tetrabutylammonium fluoride solution 1.0 M in THF, 4.60 mL, 4.60 mmol) was added into a flask and the mixture was stirred for 24 h. Excessive amount of EtOAc was added and the mixture was washed by H_2O (2 x) and brine (2 x). The organic layer was dried ($MgSO_4$), filtered, concentrated and purified by silica gel column chromatography (DCM/methanol, 100/0 – 99/1 – 99/2) to obtain **17** as a white foam (2.01 g, 2.16 mmol, 94%). ^{1}H NMR (400 MHz, Chloroform-*d*) δ 9.40 (s, 1H, NH), 8.72 (s, 1H, H2), 8.20 (s, 1H, H8), 8.04 (d, *J* = 7.1 Hz, 2H, arom. Bz), 7.63 – 7.54 (m, 1H, arom. Bz), 7.54 – 7.46 (m, 2H, arom. Bz), 7.45 – 7.36 (m, 2H, arom. Dmt), 7.36 – 7.15 (m, 7H, arom. Dmt), 6.81 (d, *J* = 9.0 Hz, 4H, arom. Dmt), 6.24 (d, *J* = 5.9 Hz, 1H, H1'), 5.55 (dd, *J* = 5.2, 3.7 Hz, 1H, H3'), 5.33 (d, *J* = 4.6 Hz, 1H, H1''), 5.25 – 5.16 (m, 2H, H2'), 4.78 (dd, *J* = 7.3, 4.5 Hz, 1H, H3''), 4.33 (q, *J* = 3.6 Hz, 1H, H4'), 4.12 (q, *J* = 3.4 Hz, 1H, H4''), 3.82 – 3.66 (m, 8H, CH_3 DMT, H5''), 3.50 (AB, *J* = 10.7, 3.7 Hz, 2H, H5'), 2.86 (s, 1H, OH), 2.13 (s, 3H, CH_3 Ac), 2.10 (s, 3H, CH_3 Ac), 1.83 (s, 3H, CH_3 Ac). ^{13}C NMR (101 MHz, $CDCl_3$) δ 170.43, 169.86, 169.70(CO, Ac), 158.67 (Cq. arom.), 151.82 (C4), 149.78 (C6), 144.34 (Cq. arom.), 135.43, 133.68 (Cq. arom), 132.85, 130.17, 128.87, 128.21, 128.03, 127.15 (arom.), 123.24 (C5), 113.30 (arom.), 101.32 (C1''), 86.96 (Cq. Dmt), 86.42 (C1'), 82.68 (C4''), 82.50 (C4'), 78.23 (C2'), 72.12 (C3'), 71.24 (C2''), 69.72 (C3''), 62.95 (C5'), 61.93 (C5''), 55.31 (CH_3, Dmt), 20.95, 20.82, 20.23 (CH_3 Ac). IR (film): 2931, 1743, 1734, 1609, 1583, 1508, 1448, 1247, 1227, 1178, 1091, 1030 cm^{-1}. HRMS (ESI$^+$) calcd for $C_{49}H_{50}N_5O_{14}$ (M+H) 932.3349. Found 932.3369. $[\alpha]_D^{20}$ +28.6 (c = 1, in $CHCl_3$)

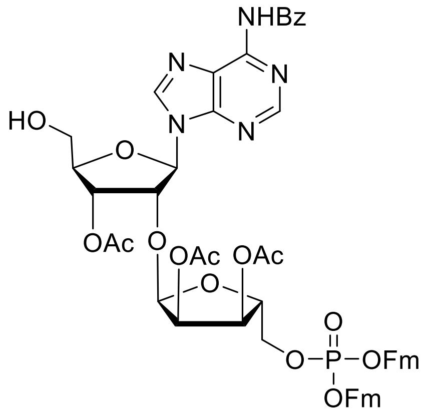

N^6-benzoyl-9-(3',2'',3'''-tris-*O*-acetyl-5''-*O*-(di-flourenylphosphoryl)-β-parobiosyl)adenine (18)

Compound **17** (1.99 g, 2.14 mmol), DCI activator (4,5-dicyanoimidazole solution 0.25 M in ACN, 17 mL, 4.28 mmol) and freshly activated 3Å molecular sieves were added in to a flask. Compound **13** (0.2 M in ACN, 16 mL, 3.21 mmol) were added into the mixture and the reaction was stirred for 10 min at room temperature after which *tBu*OOH (5.5 M in decane, 3.89 mL, 21.40 mmol) was added at 0 °C. The reaction was stirred at same temperature for 30 min and quenched by aq. $NaHCO_3$ (sat.). The mixture was filtered and EtOAc was added to the filtrate. The mixture was washed by H_2O (1 x) and brine (2 x) and the organic layer was dried (Na_2SO_4), filtered, concentrated and co-evaporated with toluene (3 x). To the residue, DCM (28 mL) and TFA (0.41 mL, 5.35 mmol) were added and the reaction was stirred for 10 min at room temperature after which it was quenched by aq. $NaHCO_3$ (sat.). DCM extracted (2 x) the mixture and the organic layers are combined and washed by H_2O (1 x) and brine (1 x). The organic layers were dried (Na_2SO_4), filtered and concentrated. The residue was purified by silica gel column chromatography (DCM/methanol, 100/0 – 100/1 – 100/2 – 100/3) to obtain **18** as a white foam (1.72 g, 1.62 mmol, 76%). ^{1}H NMR (500 MHz, Chloroform-*d*) δ 9.08 (s, 1H, NH), 8.78 (s, 1H, H2), 8.05 (s, 1H, H8), 8.03 – 7.99 (m, 2H, arom. Bz), 7.68 (ddd, *J* = 15.0, 7.6, 3.5 Hz, 4H, arom.), 7.64 – 7.59 (m, 1H, arom.), 7.56 – 7.41 (m, 6H), 7.39 – 7.19 (m, 8H), 6.00 (d, *J* = 11.5 Hz, 1H, 5'-OH), 5.96 (d, *J* = 7.8 Hz, 1H, H1'), 5.64 (d, *J* = 5.2 Hz, 1H, H3'), 5.13 (dd, *J* = 7.8, 5.2 Hz, 1H, H2'), 5.05 (dd, *J* = 7.3, 4.1 Hz, 1H, H3''), 4.95 (d, *J* = 4.6 Hz, 1H, H1''), 4.62 (dd, *J* = 7.3, 4.6 Hz, 1H, H2''), 4.31 (d, *J* = 1.8 Hz, 1H, H4'), 4.28 – 4.14 (m, 4H, CH_2 Fm), 4.12 – 4.03 (m, 3H, H4'', CH Fm), 4.00 – 3.80 (m, 4H, H5', H5''), 2.15 (s, 3H, CH_3 Ac), 2.09 (s, 3H, CH_3 Ac), 1.96 (s, 3H, CH_3 Ac). ^{13}C NMR (126 MHz, $CDCl_3$) δ 170.02, 169.62, 169.35(CO, Ac), 164.48(CO, Bz), 152.47(CH, C2), 150.66, 150.59, 143.16, 143.12, 143.09, 143.00, 141.50, 141.46, 133.54(Cq. arom.), 133.11, 129.07, 128.05, 128.03, 127.99, 127.26, 125.25, 125.24, 125.21(arom.), 124.73 (Cq, arom.), 120.13, 120.12, 120.10, 120.09 (arom.), 101.12 (C1''), 89.59 (C1'), 86.74 (C4'), 80.40 (C4''), 77.77 (C2'), 73.75 (C3'), 70.99 (C2''), 69.51, 69.46 (CH_2 Fm), 69.30 (C3''), 66.26 (C5'), 62.90 (C5'), 47.99, 47.93 (CH Fm), 21.04, 20.78, 20.40 (CH_3 Ac). ^{31}P NMR (202 MHz, $CDCl_3$) δ -1.21. IR (film): 2924, 1743, 1609, 1507, 1452, 1448, 1229, 1219, 1078, 1030, 830, 740 cm^{-1}. HRMS (ESI$^+$) calcd for $C_{56}H_{53}N_5O_{15}P$ (M+H) 1066.3270. Found 1066.3298. $[\alpha]_D^{20}$ +5.8 (c = 1, in $CHCl_3$)

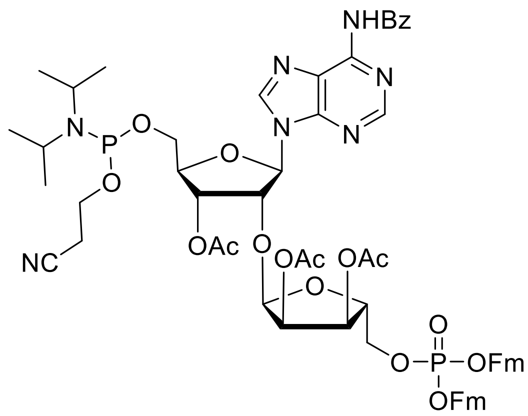

N^6-benzoyl-9-(3',2''3'''-tris-*O*-acetyl-5'-*O*-(*N*,*N*-diisopropylamino-*O*-cyanoethyl)phosphoramidite)-5''-*O*-(di-flourenylphosphoryl)-β-parobiosyl)adenine (3)

Compound **18** (1.38 g, 1.30 mmol), DMF (13 mL), DIPEA (0.56 mL, 3.24 mmol) and 2-cyanoethyl *N*,*N*-diisopropylchlorophosphoramidite **19** (0.32 mL, 1.43 mmol) were added into the flask and stirred at room temperature for 15 min. Methanol (0.2 mL) was added to quench the excessive phosphoramidite after which a liberal amount of EtOAc was added and the mixture was washed by aq. $NaHCO_3$ (sat. 1 x), H_2O (1 x) and brine (2 x). The organic layer was dried (Na_2SO_4) and filtered. The filtration was co-evaporated with toluene (1 x) then purified by automatic column (pentane/EtOAc, 20/80 – 0/100) to furnish **3** as a white foam (1.15 g, 0.91 mmol, 70%). **Note**: Careful wash was needed for the work-up because the DIPEA in the reaction could cleave the Fm group. Automatic column was performed on Biotage Isolera Specktra Four machine using High-quality IRR silica gel column (40-63 µm). ^{1}H NMR (500 MHz, Chloroform-*d*) δ 9.16 (s, 1H, NH), 8.79 (d, *J* = 7.6 Hz, 1H, H2), 8.45 (d, *J* = 8.5 Hz, 1H, H8), 8.10 – 7.93 (m, 2H, arom.), 7.76 – 7.18 (m, 19H, arom.), 6.37 – 6.21 (m, 1H, H1'), 5.48 (ddd, *J* = 20.8, 5.1, 3.2 Hz, 1H, H3'), 5.24 (d, *J* = 4.5 Hz, 1H, H1''), 5.11 (ddd, *J* = 7.5, 6.0, 3.7 Hz, 1H, H3''), 4.94 (dt, *J* = 6.3, 4.6 Hz, 1H, H2'), 4.65 (ddd, *J* = 10.9, 7.3, 4.5 Hz, 1H, H2''), 4.35 (dd, *J* = 3.1, 1.7 Hz, 1H, H4'), 4.29 – 3.72 (m, 12H, Fm, H5', H5'', O$CH_2$$CH_2$CN), 3.67 – 3.44 (m, 2H, *CH*(CH_3)$_2$), 2.71 – 2.59 (m, 2H, *CH_2*CN), 2.15 (d, *J* = 8.8 Hz, 3H, CH_3 Ac), 2.09 (s, 3H, CH_3 Ac), 1.83 (d, *J* = 9.3 Hz, 2H, CH_3 Ac), 1.20-1.14 (m, 12H, CH*(CH_3)$_2$*). ^{13}C NMR (126 MHz, $CDCl_3$) δ 170.09, 170.08, 169.77, 169.73, 169.41, 169.38 (CO Ac), 164.67, 164.64 (CO Bz), 151.99, 151.89 (C4), 149.69 (C6), 143.05, 143.03, 142.96, 142.94, 141.42, 141.40, 133.79 (Cq. arom.), 132.84, 132.82, 128.94, 128.91, 127.94, 127.19, 125.13, 125.10 (arom.), 123.19, 123.07 (C5), 120.06, 120.03 (arom.), 117.98, 117.76 (CN), 101.59 (C1''), 86.19, 85.84 (C1'), 83.13, 83.06, 82.82, 82.75 (C4'), 80.50, 80.43 (C4''), 79.46, 79.43 (C2'), 72.39, 72.17(C3'), 70.80, 70.77 (C2''), 69.41, 69.37 (CH_2 Fm), 69.29, 69.26 (C3''), 66.41, 66.37, 66.32 (C5''), 62.76, 62.66, 62.62, 62.54 (C5'), 58.80, 58.66, 58.63, 58.50 (O*CH2*CH_2CN), 47.92, 47.90, 47.86, 47.83 (CH Fm), 43.29, 43.24, 43.19, 43.15 ((CH_3)$_2$*CH*N), 24.78, 24.72(*(CH_3)$_2$*CHN), 20.95, 20.88, 20.74 (CH_3 Ac), 20.47, 20.41 (OCH_2*CH_2*CN), 20.19, 20.16(CH_3 Ac). ^{31}P NMR (202 MHz, $CDCl_3$) δ 150.02, 149.62, 14.79 (H-phosphate), -1.05, -1.07. IR (film): 2968, 1744, 1609, 1451, 1236, 1074, 1025, 981, 741 cm^{-1}. HRMS (ESI$^+$) calcd for $C_{59}H_{57}N_6O_{17}P_2$ ([H-phosphonate]+H) 1183.3250. Found 1183.3246. $[\alpha]_D^{20}$ +13.9 (c = 1, in DCM)

1-*O*-methyl-α-ADPr dimer (1a, manual synthesis)

To a 5 mL reaction syringe with filter frit, **2a** (200 mg, 10 µmol) or **2b** (50 mg, 10 µmol) was added, washed with ACN (3 x) under N_2. 2 mL DBU solution (10%, v/v, in ACN) and was shaken for 15 min (2 x) to remove Fm groups on 5-O-phosphate after which the syringe was drained and the resin washed with ACN (5 x) to give **20a/b**. Then:

Cycle A: ETT (0.48 mL, 0.25 M in ACN) and **3** (0.4 mL, 0.2 M in ACN) were added to the resin and the mixture was shaken for 10 min, drained and followed by a second addition of ETT and **3**. The syringe was drained and the resin was washed with ACN (5 x) under N_2. The intermediate phosphate-phosphite was oxidized with (1*S*)-(+)-(10-camphorsulfonyl)-oxaziridine (CSO) solution (2 mL, 0.5 M in ACN) for 5 min (2 x) and washed with ACN (5 x). DBU solution (2 mL, 10%, v/v, in ACN) was added into the syringe and was shaken for 15 min (2 x) to remove Fm groups on 5'-phosphate and CE group after which was drained and washed with ACN (5 x) under N_2.

Cycle B: ETT (0.48 mL, 0.25 M in ACN) and **4** (0.4 mL, 0.2 M in ACN) were added to the resin and the mixture was shaken for 10 min, drained and followed by a second addition of ETT and **4**. The syringe was drained and the resin was washed with ACN (5 x) under N_2. The intermediate phosphate-phosphite was oxidized with CSO solution (2 mL, 0.5 M in ACN) for 5 min (2 x) and washed with ACN (5 x). DBU solution (2 mL, 10%, v/v, in ACN) was added into the syringe and was shaken for 10 min to remove CE group after which the syringe was drained and the resin was washed with ACN (5 x) under N_2. The resin was treated with NH_4OH (35%) overnight to cleave the product from the resin and remove all the protecting groups. The mixture was filtered and the filtrate was concentrated.

The crude material was purified by anion exchange column chromatography to obtain ADPr dimer **1a** (0.32 mg, 0.27 µmol, 3%, from CPG; 3.23 mg, 2.73 µmol, 27%, from TG) as a white solid.

Column: Resource Q 6mL.

Gradient: 30% - 70%. (A: 10 mM NH_4OAc, B: 1 M NH_4OAc)

Dimer **1a**:

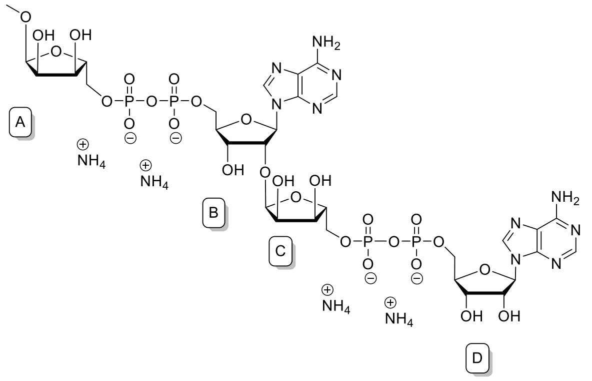

^{1}H NMR (500 MHz, Deuterium Oxide) δ 8.47 (s, 2H, H2), 8.27 (d, *J* = 2.7 Hz, 2H, H8), 6.19 (d, *J* = 3.1 Hz, 1H, H1-B), 5.98 (d, *J* = 5.9 Hz, 1H, H1-D), 5.30 (d, *J* = 4.3 Hz, 1H, H1-C), 4.93 – 4.90 (m, 1H, H1-A), 4.69 – 4.63 (m, 1H, H2-D), 4.55 (dd, *J* = 5.3, 3.1 Hz, 1H, H2-B), 4.53 – 4.43 (m, 2H, H3-B, H3-D), 4.39 – 4.25 (m, 4H, H4-BCD, H5-B), 4.25 – 4.15 (m, 6H, H2-A, H3-A, H4-A, H5-B, H5-D), 4.15 – 4.10 (m, 2H, H2-C, H3-C), 4.03 – 4.00 (m, 4H, H5-A, H5-C), 3.36 (s, 3H, OMe). ^{13}C NMR (126 MHz, D_2O) δ 152.43, 152.30 (C4), 148.37, 147.92 (C6), 118.40, 118.19 (C5), 103.20 (C1-A), 101.24 (C1-C), 87.27 (C1-D), 87.09 (C1-B), 84.19, 84.15, 84.13 (C4-C, C4-D), 83.17, 83.10, 83.02 (C4-B, C4-A), 78.87 (C2-B), 74.57 (C2-D), 71.33 (C2-A), 70.78 (C2-C), 70.49 (C3-D), 69.78 (C3-A), 69.61 (C3-C), 68.72 (C3-B), 65.59, 65.55 (C5-A, C5-C), 65.23, 65.21 (C5-D), 64.29, 64.25 (C5-B), 55.40 (OMe). ^{31}P NMR (202 MHz, D_2O) δ -11.08, -11.18, -11.26, -11.32, -11.37, -11.44, -11.54. LC-MS: Rt = 2.98 min. 0-50% NH_4OAc. ESI MS+ calc. 1115.2 found 1115.2 $[M+1]^+$. HRMS (ESI^+) calcd for $C_{31}H_{47}N_{10}O_{27}P_4$ (M+H) 1115.1557. Found 1115.1558.

1-*O*-methyl-α-ADPr trimer 1b (by oligonucleotide synthesizer)

200 mg **2b** was added into a 5 mL reaction syringe with filter frit and the resin was washed with ACN (5 x) under N_2. 3 mL DBU solution (10%, v/v, in ACN) was added into the syringe and was shaken for 20 min to remove Fm groups on 5-phosphate after which was drained. The DBU treatment was repeated for another 20 min. The resin was washed with ACN (5 x) and dried under reduced pressure to remove traceless water before use. 50 mg resin from above was transferred into a reaction column of a Mermade 6 oligonucleotide synthesizer and the complete synthesis was performed under an argon atmosphere. For trimer synthesis, Cycle A was performed twice and Cycle B was performed once.

Cycle A: The resin was rinsed with ACN (3 x) and drained. BTT (600 µL, 0.25 M in ACN) and **3** (300 µL, 0.1 M in ACN) were added into the resin and the mixture was left to stand for 10 min, drained. Repeat this coupling for two more times. The resin was rinsed by with ACN (3 x). The intermediate phosphate-phosphite was oxidized with CSO solution (2 mL, 0.5 M in MeCN) for 5 min (2 x). The resin was drained and washed with ACN (3 x). DBU solution (2 mL, 10%, v/v, in ACN) was added to the resin and was left to stand for 10 min (4 x) to remove Fm groups on 5'-phosphate and CE group after which was drained and washed with ACN (3 x).

Cycle B: The resin was rinsed with ACN (3 x) and drained. BTT (600 µL, 0.25 M in ACN) and **4** (300 µL, 0.1 M in ACN) were added into the resin and the mixture was left to stand for 10 min, drained. Repeat this coupling for two more times. The resin was rinsed by with ACN (3 x). The intermediate phosphate-phosphite was oxidized with CSO solution (2 mL, 0.5 M in MeCN) for 5 min (2 x). The resin was drained and washed with ACN (3 x). DBU solution (2 mL, 10%, v/v, in ACN) was added into the resin and was left to stand for 10 min to remove CE group after which was drained and washed with ACN (3 x).

The resin was transferred to a tube and treated with 10 mL NH_4OH (35%). The mixture was stirred overnight in a sealed flask, filtered and concentrated. The crude was purified by anion exchange to

obtain ADPr trimer **1b** (6.16 mg, 3.51 µmol, 35%) and ADPr dimer **1a** (4.5 mg, 3.81 µmol, 38%) as white solid.

Column: Resource Q 6mL.

Gradient: 30% - 70%. (A: 10 mM NH_4OAc, B: 1 M NH_4OAc)

Trimer **1b**:

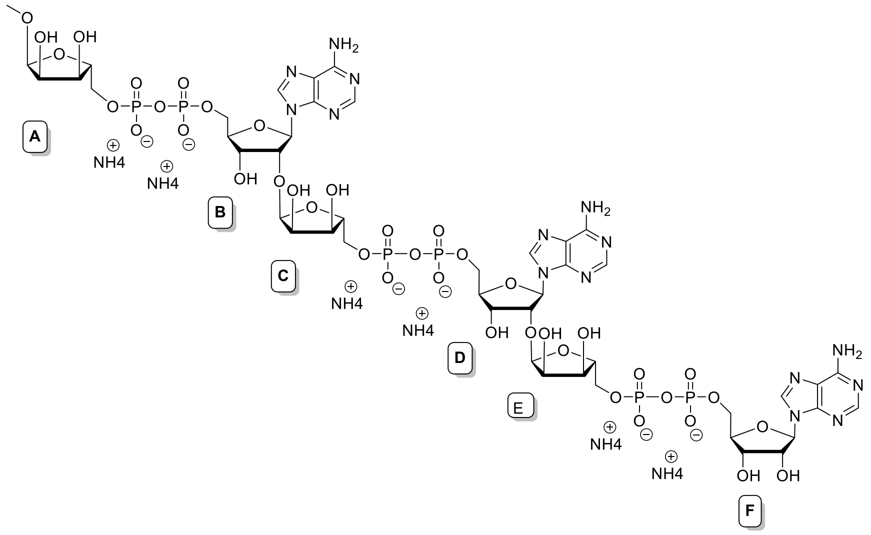

^{1}H NMR (500 MHz, Deuterium Oxide) δ 8.36 – 8.24 (m, 3H, H2), 8.09 – 7.97 (m, 3H, H8), 6.13 (d, *J* = 3.2 Hz, 1H, H1-B), 5.97 (d, *J* = 3.1 Hz, 1H, H1-D), 5.94 (d, *J* = 5.9 Hz, 1H, H1-F), 5.30 (d, *J* = 4.2 Hz, 1H, H1-C), 5.19 (d, *J* = 4.1 Hz, 1H, H1-E), 4.85 – 4.82 (m, 1H, H1-A), 4.63 (t, *J* = 5.5 Hz, 1H, H2-F), 4.57 (dd, *J* = 5.3, 3.3 Hz, 1H, H2-B), 4.54 (t, *J* = 5.7 Hz, 1H, H3-B), 4.48 – 4.40 (m, 3H, H2-D, H3-F, H3-D), 4.34 – 3.94 (m, 24H), 3.33 (s, 3H, OMe). ^{13}C NMR (214 MHz, D_2O) δ 155.66, 155.55, 155.45 (C4), 149.44, 149.14, 148.84 (C6), 119.28, 119.11, 119.00 (C5), 104.13 (C1-A), 102.35 (C1-E), 102.24 (C1-C), 87.87 (C1-F), 87.71 (C1-D), 87.40 (C1-B), 85.18, 85.14, 85.10, 84.82, 84.78, 84.09, 84.05, 84.01, 83.96, 83.81, 83.78 (C4-ABCDEF), 80.26 (C2-D), 79.69 (C2-B), 75.38 (C2-F), 72.33, 72.30, 71.70, 71.41, 70.82, 70.81, 70.80, 70.55, 70.05, 69.87 (the rest C2, C3), 66.50, 66.19, 65.51 (C5-ABCDEF), 56.33, 56.32 (OMe). ^{31}P NMR (202 MHz, D_2O) δ -10.45, -10.51, -10.55, -10.61, -10.67, -10.69, -10.71, -10.78, -10.80, -10.82. LC-MS: Rt = 2.96 min. 0-50% NH_4OAc. ESI MS+ calc. 1656.2 found 1656.2 $[M+1]^+$. HRMS (ESI^+) calcd for $C_{46}H_{68}N_{15}O_{40}P_6$ (M+H) 1656.2168. Found 1656.2196.

1-*O*-methyl-α-ADPr pentamer/tetramer (**1c/1d**, from pentamer synthesis)

1d was synthesized according to the similar procedure as that of **1b**. Cycle A was performed 4 times and cycle B was performed once after which the resin was transferred into a tube and treated with NH_4OH (35%) overnight in a sealed flask. The mixture was filtered, concentrated under reduced pressure and purified by anion exchange and gel filtration to obtain ADPr trimer **1b** (1.18 mg, 0.71

µmol, 7%), tetramer **1c** (1.35 mg, 0.61 µmol, 6%) and pentamer **1d** (0.69 mg, 0.25 µmol, 3%) as white solid.
Anion exchange column: Resource Q 6mL. Gradient: 50% - 80%. (A: 10 mM NH_4OAc, B: 1 M NH_4OAc)
Gel filtration gradient: 20% ACN in 0.15 M aq. NH_4HCO_3.

Trimer **1b**: Identical data with manual synthesis.
Tetramer **1c**:

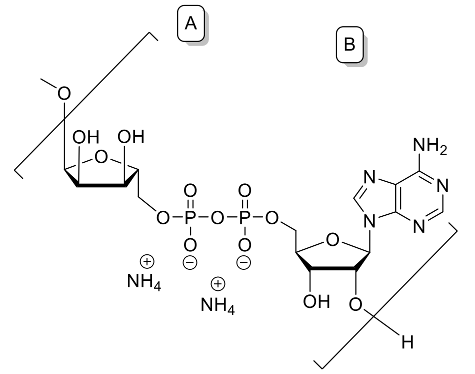

4

^{1}H NMR (850 MHz, Deuterium Oxide) δ 8.39 (s, 1H), 8.38 (s, 1H), 8.32 (s, 1H), 8.31 (s, 1H, H2-ade), 8.10 (d, *J* = 1.2 Hz, 1H), 8.08 (d, *J* = 1.2 Hz, 1H), 8.06 (d, *J* = 1.1 Hz, 1H), 8.02 (d, *J* = 1.1 Hz, 1H, H8-ade), 6.17 (d, *J* = 3.5 Hz, 1H, H1-B), 6.03 (d, *J* = 3.0 Hz, 2H, H1-B), 5.99 (d, *J* = 5.8 Hz, 1H, H1-B), 5.34 (d, *J* = 4.3 Hz, 1H, H1-A), 5.27 (t, *J* = 4.5 Hz, 2H, H1-A), 4.92 – 4.89 (m, 1H, H1-A), 4.66 – 4.65 (m, 2H), 4.58 (t, *J* = 5.7 Hz, 1H), 4.55 (dd, *J* = 5.3, 3.8 Hz, 1H), 4.52 (t, *J* = 5.5 Hz, 1H), 4.50 – 4.45 (m, 3H), 4.39 – 4.01 (m, 32H), 3.38 (d, *J* = 1.0 Hz, 3H, OMe). ^{31}P NMR (202 MHz, D_2O) δ -11.10, -11.21, -11.24, -11.31, -11.34, -11.43, -11.47. LC-MS: Rt = 2.83 min. 0-50% NH_4OAc. ESI MS+ calc. 1099.1 found 1099.8 $[M+2]^+$. HRMS (ESI^+) calcd for $C_{61}H_{90}N_{20}O_{53}P_8$ (M+2H)/2 1099.1426. Found 1099.1446.

Pentamer **1d**:

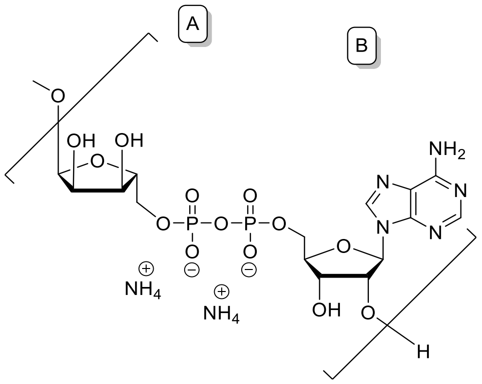

5

^{1}H NMR (850 MHz, Deuterium Oxide) δ 8.38 (s, 1H), 8.33 (s, 1H), 8.31 – 8.26 (m, 3H, H2-ade), 8.12 (s, 1H), 8.02 (d, *J* = 3.7 Hz, 2H), 7.98 (d, *J* = 5.0 Hz, 2H, H8-ade), 6.19 (d, *J* = 3.8 Hz, 1H, H1-B), 6.08 – 6.05

(m, 2H, H1-B), 6.04 (t, *J* = 1.8 Hz, 1H, H1-B), 5.98 (d, *J* = 5.9 Hz, 1H, H1-B), 5.31 (d, *J* = 4.2 Hz, 1H, H1-A), 5.26 (d, *J* = 3.6 Hz, 1H, H1-A), 5.24 (s, 1H, H1-A), 5.22 (d, *J* = 4.2 Hz, 1H, H1-A), 4.87 (d, *J* = 2.5 Hz, 1H, H1-A), 4.65 (dt, *J* = 10.4, 4.9 Hz, 2H), 4.59 (t, *J* = 5.5 Hz, 1H), 4.52 (d, *J* = 3.3 Hz, 5H), 4.49 – 4.46 (m, 1H), 4.39 – 3.98 (m, 41H), 3.37 (s, 3H, OMe). ^{31}P NMR (202 MHz, D_2O) δ -11.10, -11.10, -11.12, -11.21, -11.25, -11.31, -11.36, -11.45. LC-MS: Rt = 2.84 min. 0-50% NH_4OAc. ESI MS+ calc. 913.4 found 913.1 $[M+3]^+$. HRMS (ESI$^+$) calcd for $C_{61}H_{90}N_{20}O_{53}P_8$ (M+2H)/2 1369.6732. Found 1369.6742.

References

1. P. Finch, G. M. Iskander and A. H. Siriwardena, *Carbohydr. Res.*, 1991, **210**, 319-325.
2. N. Minakawa, Y. Kato, K. Uetake, D. Kaga and A. Matsuda, *Tetrahedron*, 2003, **59**, 1699-1702.
3. Q. Liu, H. A. V. Kistemaker, H. S. Overkleeft, G. A. van der Marel and D. V. Filippov, *Chem. Commun.*, 2017, **53**, 10255-10258.

RP-HPLC analysis of compound **1a-d** with UV detection

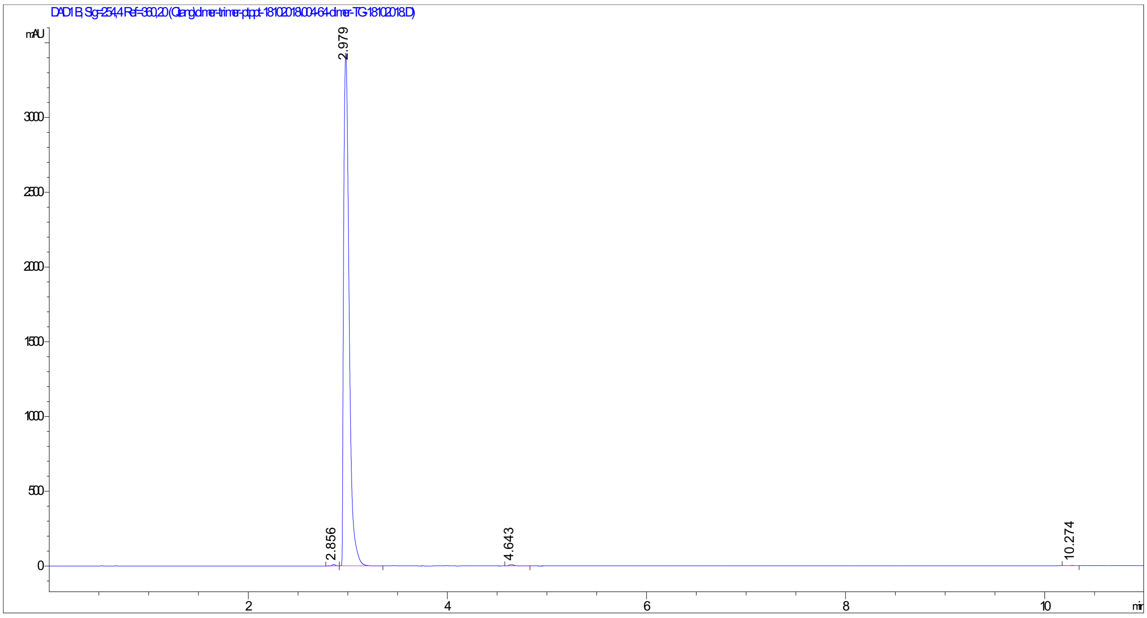

Figure 1S. Chromatogram of compound **1a**

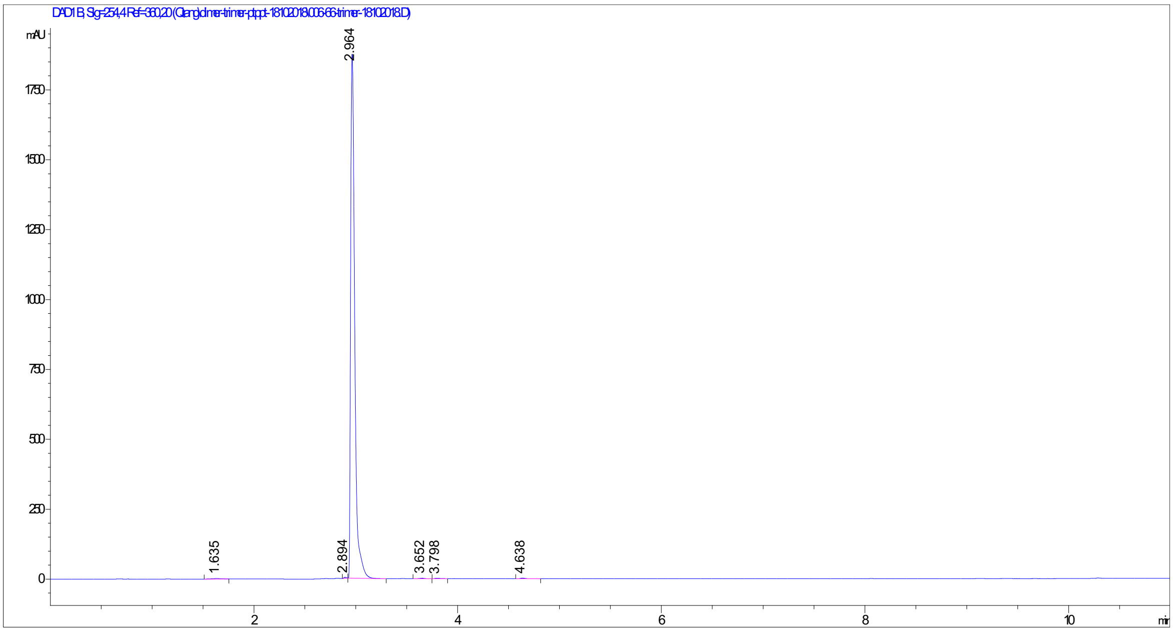

Figure 2S. Chromatogram of compound **1b**

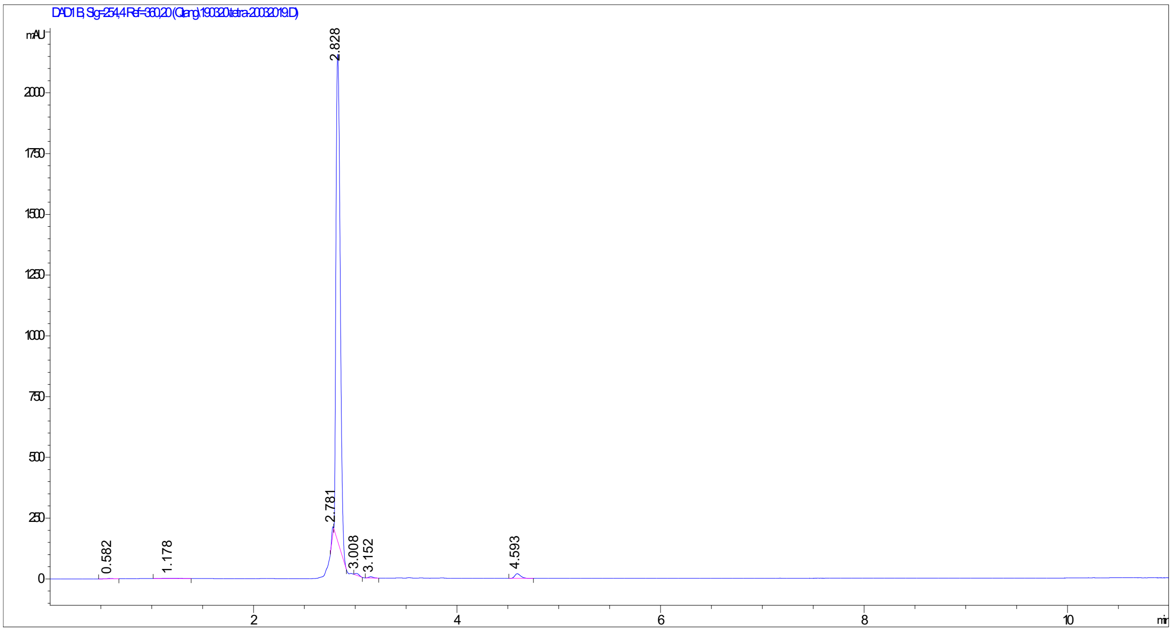

Figure 3S. Chromatogram of compound **1c**

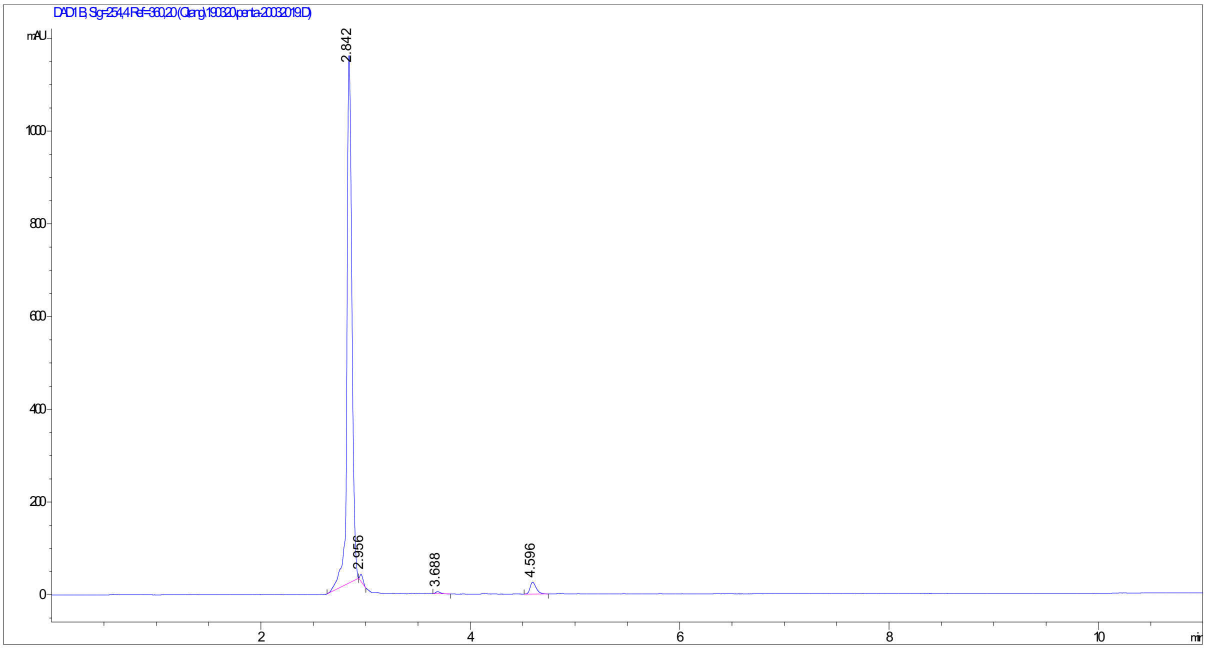

Figure 4S. Chromatogram of compound **1d**

HRMS analysis of compound **1a-d**

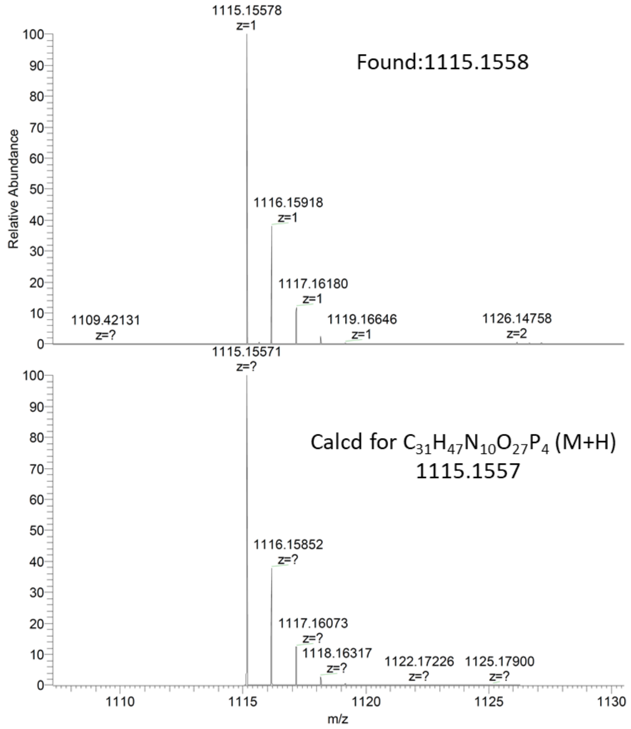

Figure 5S. HRMS analysis of compound **1a**

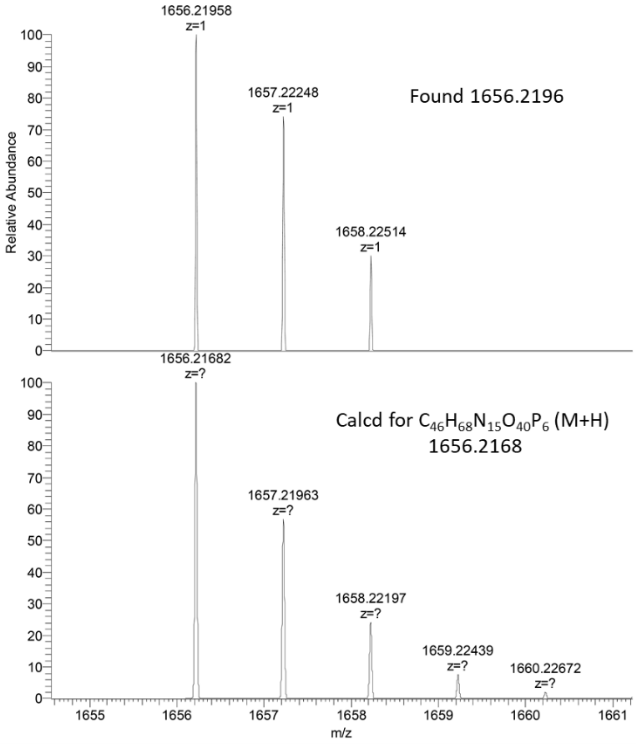

Figure 6S. HRMS analysis of compound **1b**

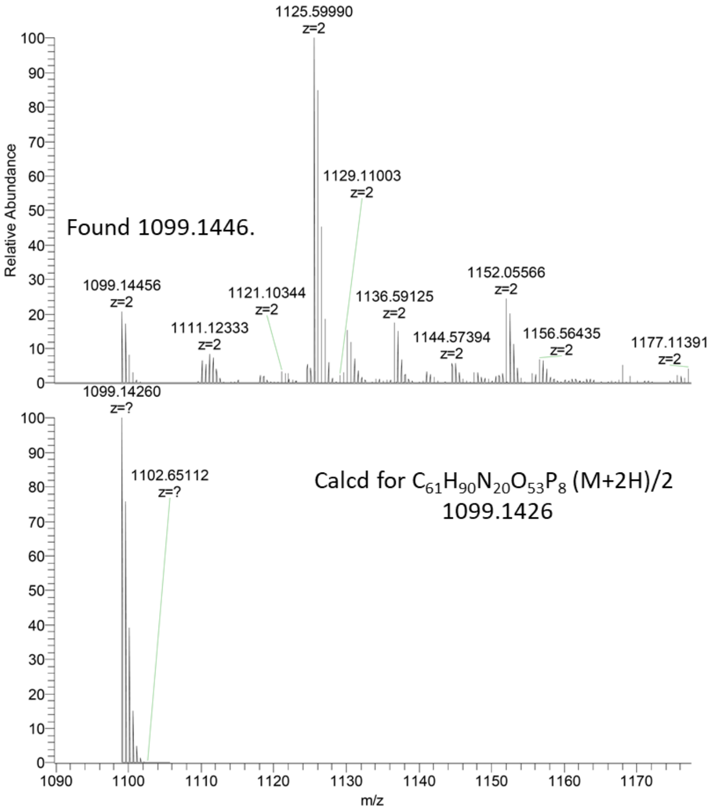

Figure 7S. HRMS analysis of compound **1c**

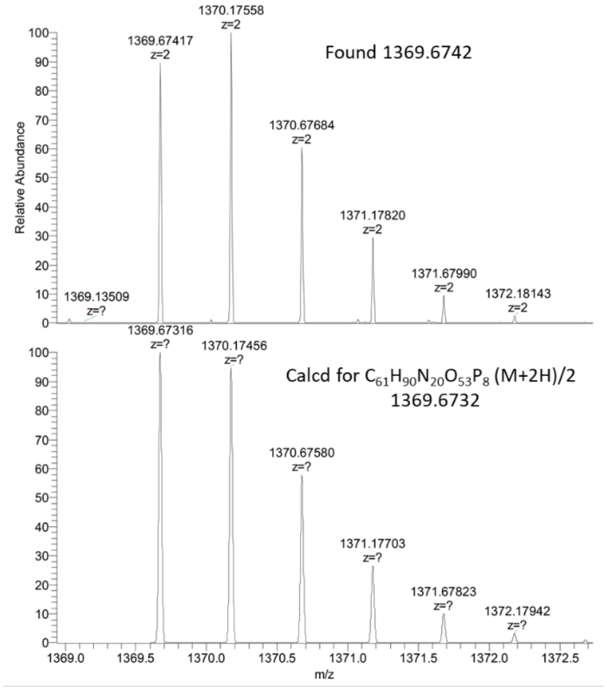

Figure 8S. HRMS analysis of compound **1d**

Materials and methods biology

Protein expression and purification

The recombinant human ALC1 (CHD1L) macrodomain (residues 636-878) was expressed in *E. coli* Rosetta (DE3) from the pET-MCN plasmid as an N-terminally His_6-tagged fusion protein. Starting cultures were grown in LB-medium supplemented with Ampicillin and Chloramphenicol overnight at 30°C and used at a 1:50 dilution to inoculate the expression cultures. Expression cultures were grown at 37°C until reaching an OD_{600} of 0.4. The temperature was then reduced to 18°C and the cultures were grown until reaching an OD_{600} of 0.6 – 0.8 before protein expression was induced with 0.2 mM isopropyl β-D-1 thiogalactopyranoside (IPTG). After induction, protein expression was allowed to proceed for 18 hr. The cells were harvested by centrifugation and the pellets were either directly used for protein purification or stored at -80 °C. The bacteria were lysed by sonication at 4 °C in lysis buffer (50 mM HEPES (pH 7.4), 500 mM NaCl, 20 mM imidazole, 5 mM dithiothreitol (DTT)) supplemented with Complete EDTA free protease inhibitor tablets (Roche). The lysate was cleared by centrifugation at 35,000 × g and loaded onto a HisTrap HP column operated on a ÄKTA pure FPLC system (GE Healthcare). After washing with 15 column volumes (CV) lysis buffer, the protein was eluted in a 10 CV gradient of elution buffer (lysis buffer supplemented with 500 mM imidazole). The fractions containing the protein of interest were pooled and diluted with 25 mM HEPES (pH 7.4), 10 mM NaCl, 5% (w/v) glycerol, 1 mM DTT to a final salt concentration of ~50 mM NaCl and loaded onto a Resource S 6mL cation exchange column (GE Healthcare). The protein was eluted using a linear gradient up to 50% 25 mM HEPES (pH 7.4), 1M NaCl, 1mM DTT. Peak fractions containing the ALC1 macrodomain were pooled and concentrated by ultrafiltration (Amicon ultra-15, 10kDa MWCO) and loaded onto a HiLoad 16/600 Superdex 200 column (GE Healthcare) equilibrated in 25 mM HEPES (pH 7.4), 250 mM NaCl, 1 mM DTT. The pure protein fractions were pooled, concentrated, supplemented with 20% (v/v) glycerol and snap frozen in liquid nitrogen for storage at -80 °C.

Isothermal titration calorimetry (ITC)

All experiments were conducted on a Malvern PEAQ-ITC instrument. For this, the ALC1 macrodomain was dialyzed overnight against 25 mM HEPES (pH 7.4), 100 mM NaCl, 0.25 mM tris(2-carboxyethyl)phosphine (TCEP), at 4 °C. Prior to the experiments, the protein was centrifuged for 20 min at 25,000 × g at 4 °C, and the protein concentration was determined by absorbance measurements at 280 nm wavelength using a calculated molar extinction coefficient of 35,410 M^{-1} cm^{-1} (ProtParam). ADP-ribose oligomers were solubilized in the dialysis buffer and the concentration was determined by absorbance measurements at 260 nm wavelength using a molar extinction coefficient of 40.5 mM^{-1}

cm^{-1} for the ADP-ribose trimers, 54 mM^{-1} cm^{-1} for ADP-ribose tetramers and 67.5 mM^{-1} cm^{-1} for the pentamers. ITC experiments were conducted at 25 °C at a reference power of 10 µcal/sec, a stirring speed of 750 rpm and with 150 sec spacing between the 19 injections. The binding reactions were performed by injecting 90-110 µM ligand into 7.5 µM protein. PEAQ-ITC Analysis Software (Malvern) was used for the analysis of the binding isotherms and GraphPad Prism was used to plot the data.

Thermal shift assay

Label-free thermal shift assays were conducted on a NanoTemper Tycho NT.6 instrument. The samples were prepared as described for the ITC experiments. The thermal unfolding of the ALC1 macrodomain (7.5 µM) was monitored in the absence or presence of a ~7-fold molar excess of tri-, tetra- or penta-ADPr by measuring intensity changes of the protein's intrinsic tryptophane and tyrosine fluorescence at 350 nm and 330 nm. The first derivative of the melting curves measured as a 350 nm/330 nm ratio was normalized to the lowest value before unfolding and to the peak maximum and plotted using GraphPad Prism.

Compound **5**, ^{1}H--NMR, $CDCl_3$, 500 MHz

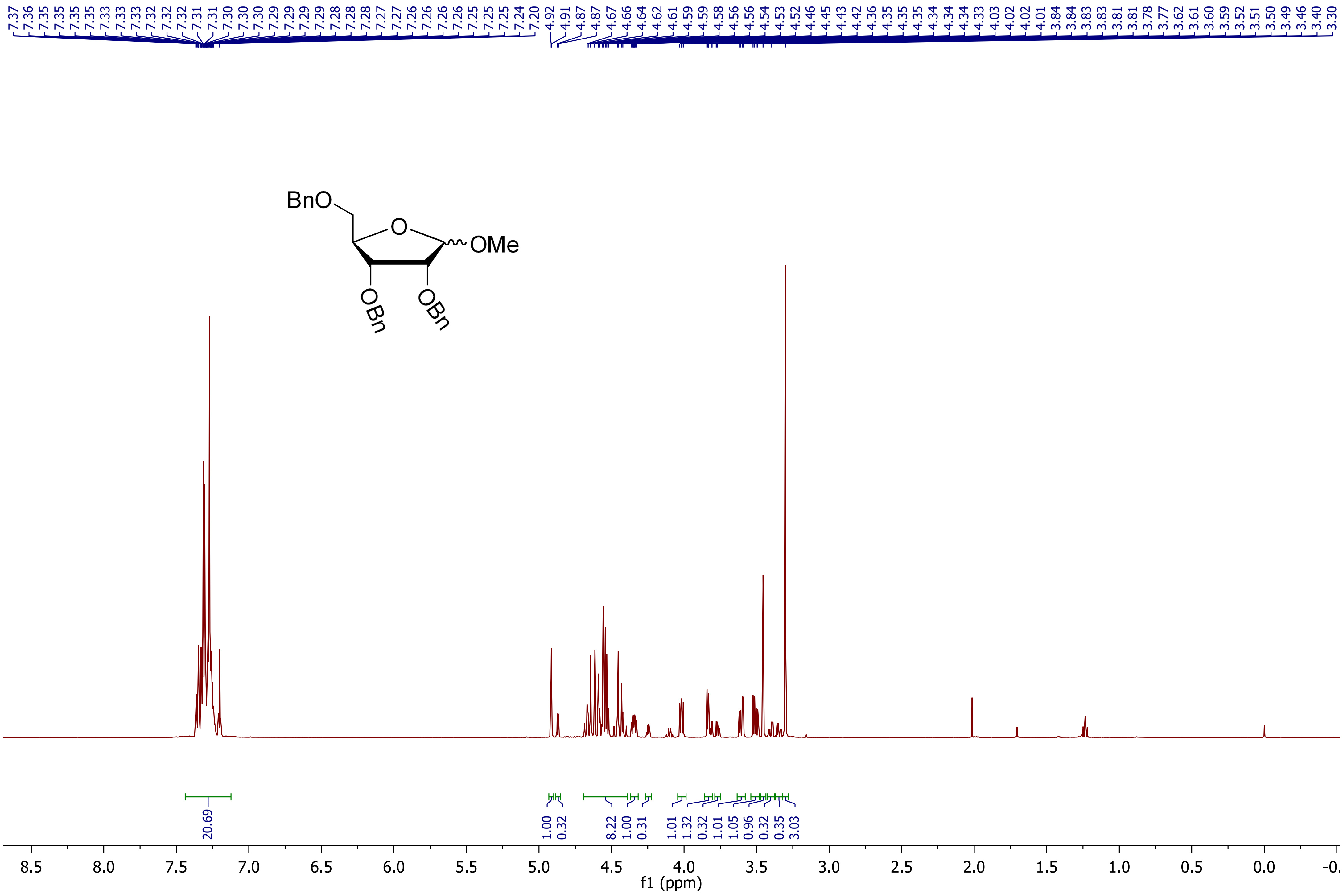

Compound **5**, ^{13}C--NMR, $CDCl_3$, 126 MHz

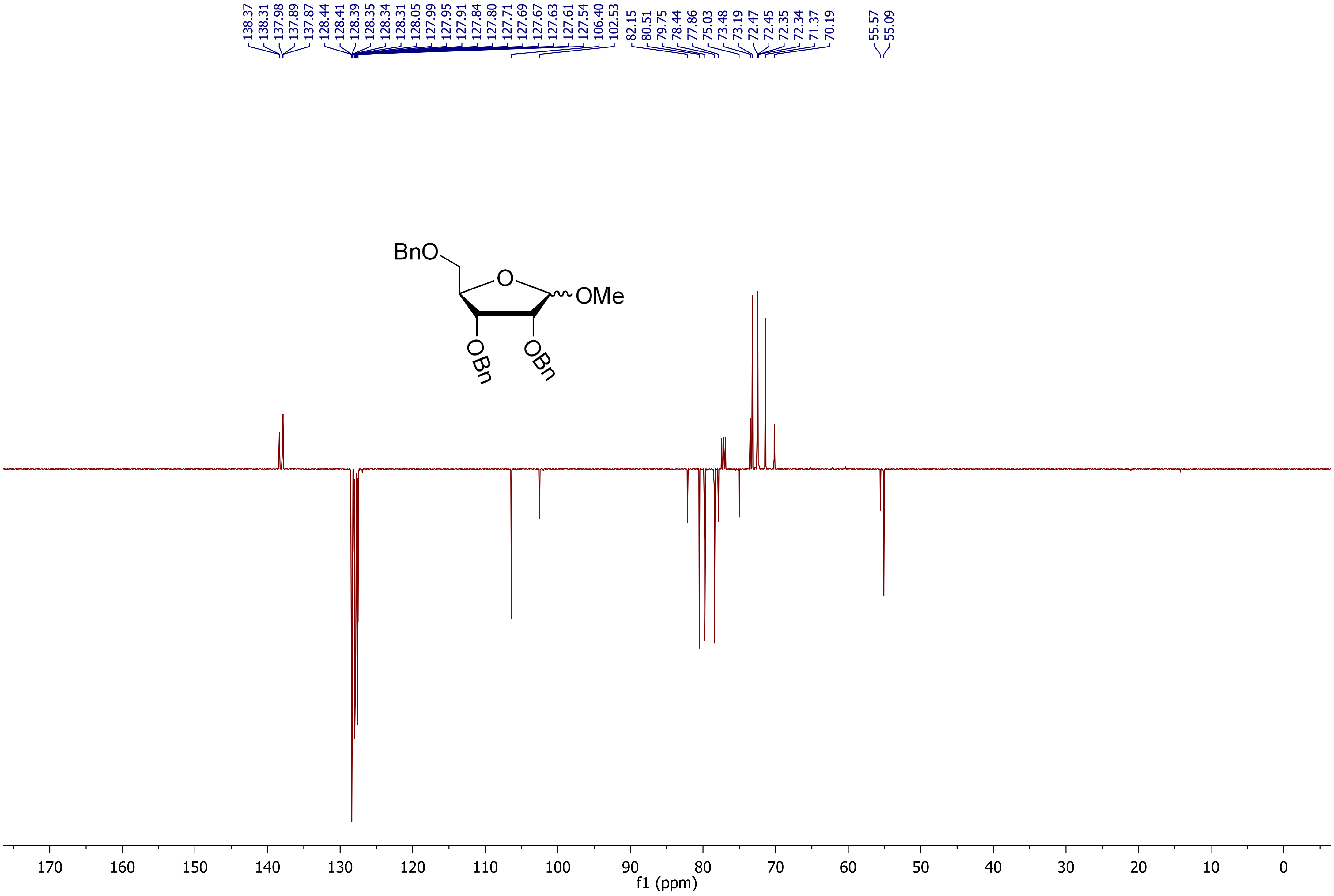

Compound **6**, ^{1}H--NMR, $CDCl_3$, 500 MHz

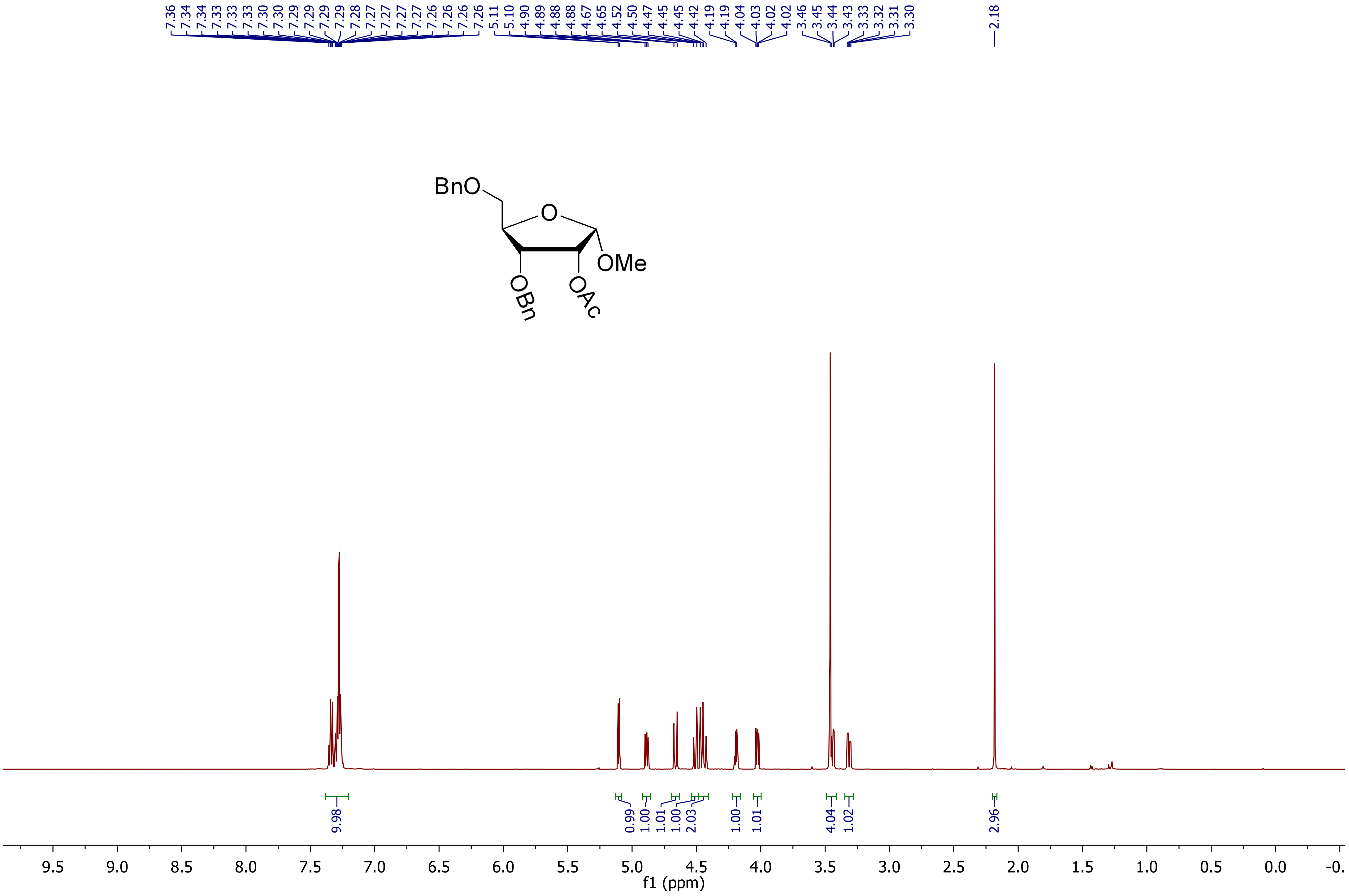

Compound **6,** ^{13}C--NMR, $CDCl_3$, 126 MHz

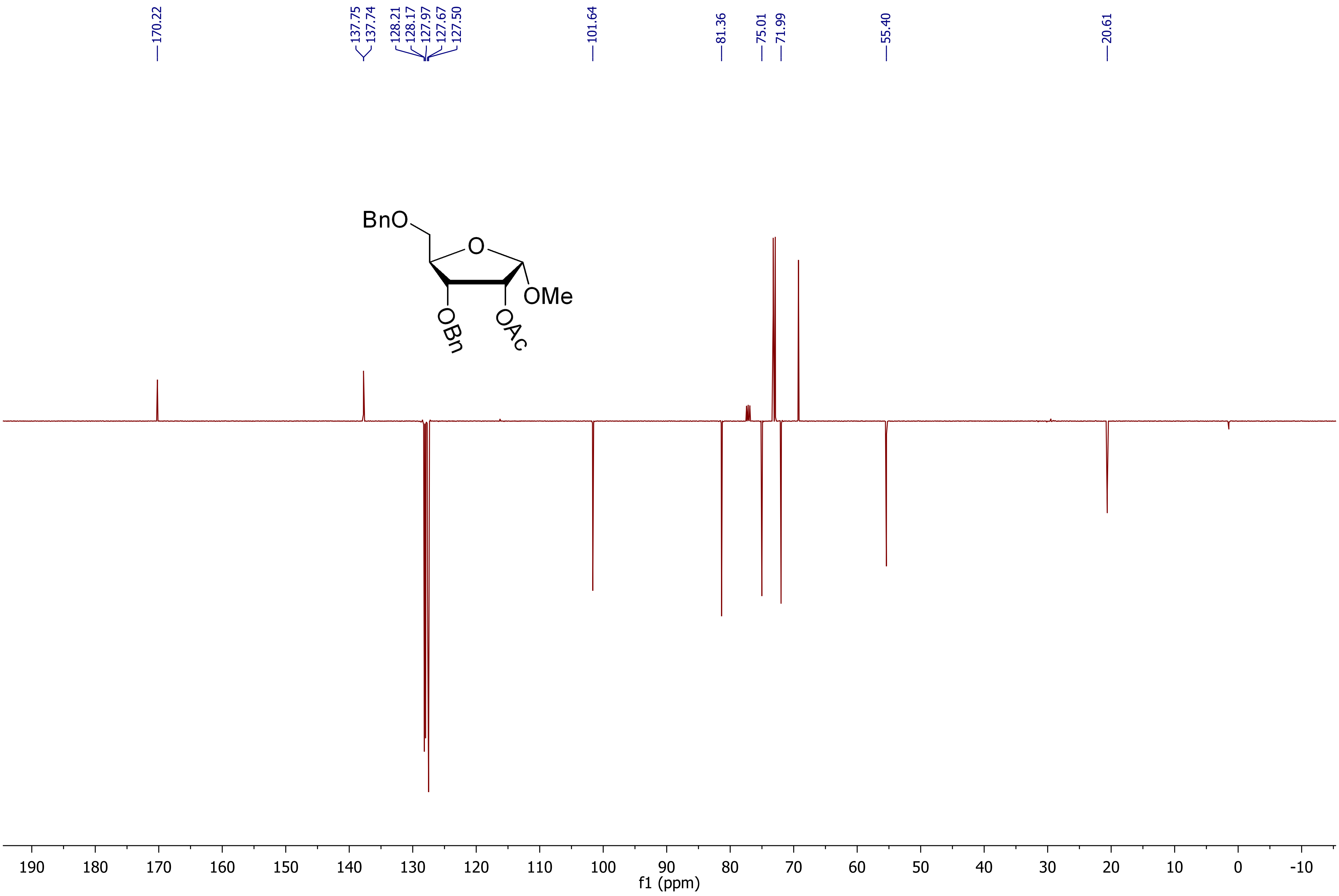

Compound **7**, ^{1}H--NMR, $CDCl_3$, 500 MHz

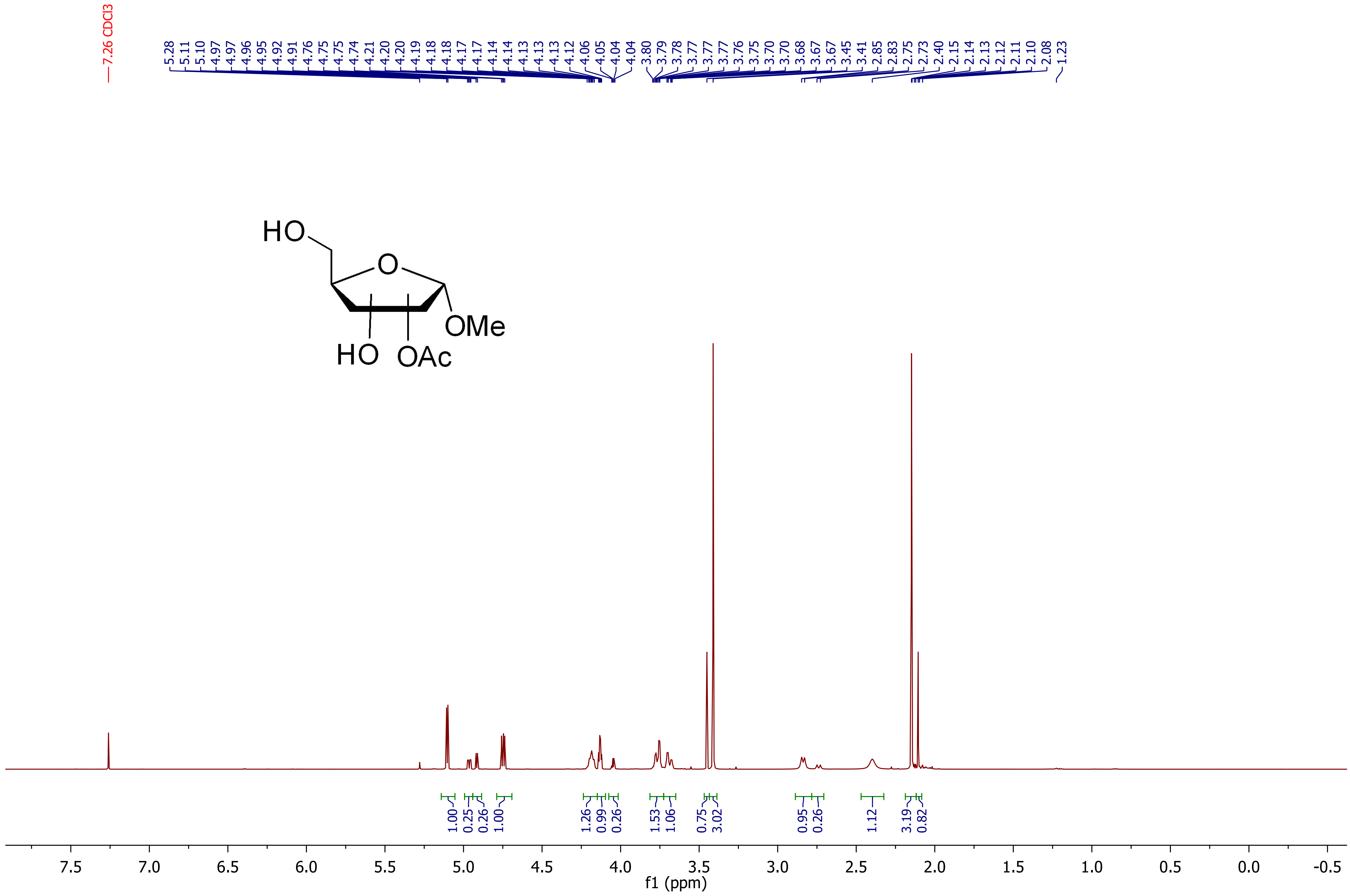

Compound **7**, ^{13}C--NMR, $CDCl_3$, 126 MHz

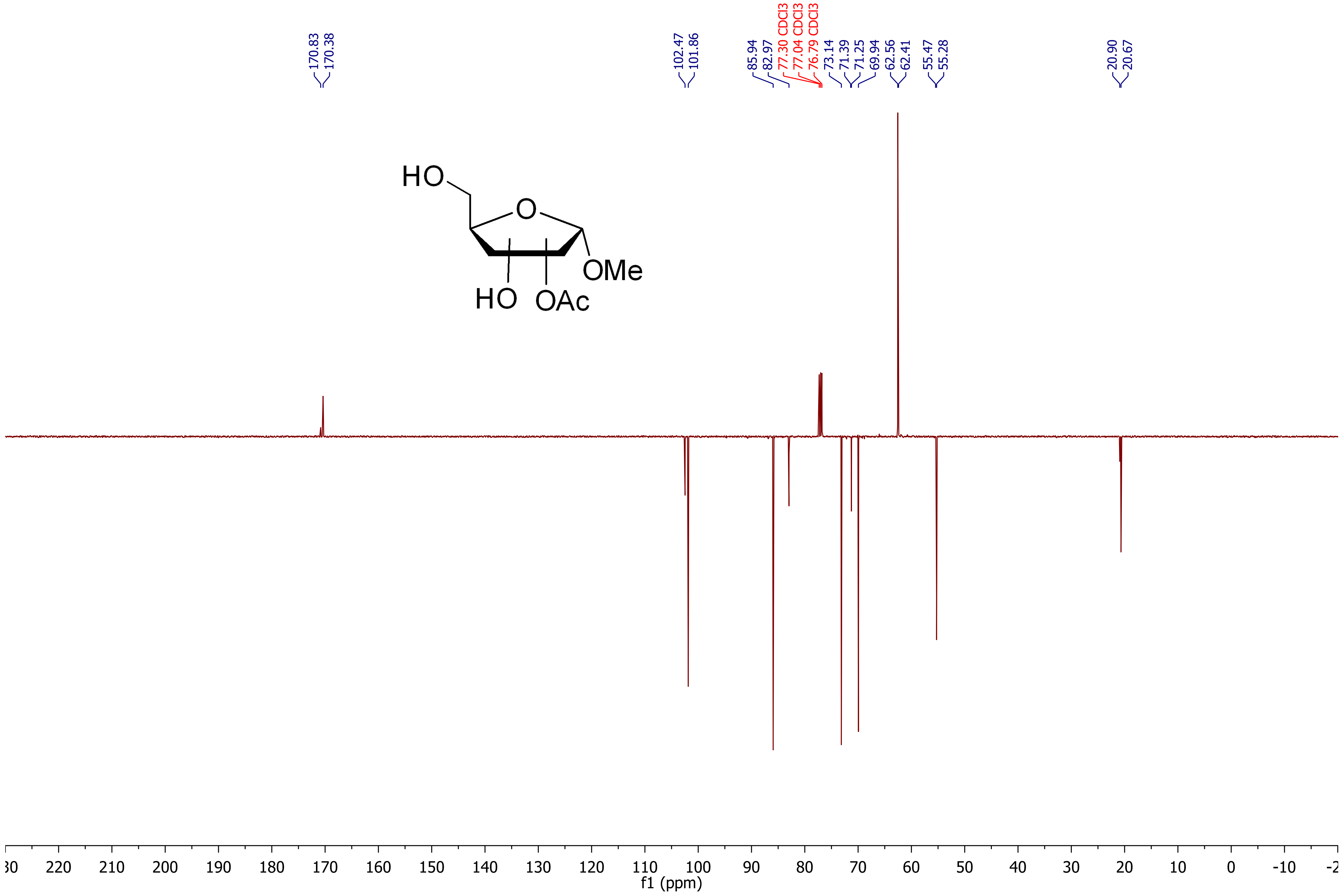

Compound **8**, ^{1}H--NMR, $CDCl_3$, 400 MHz

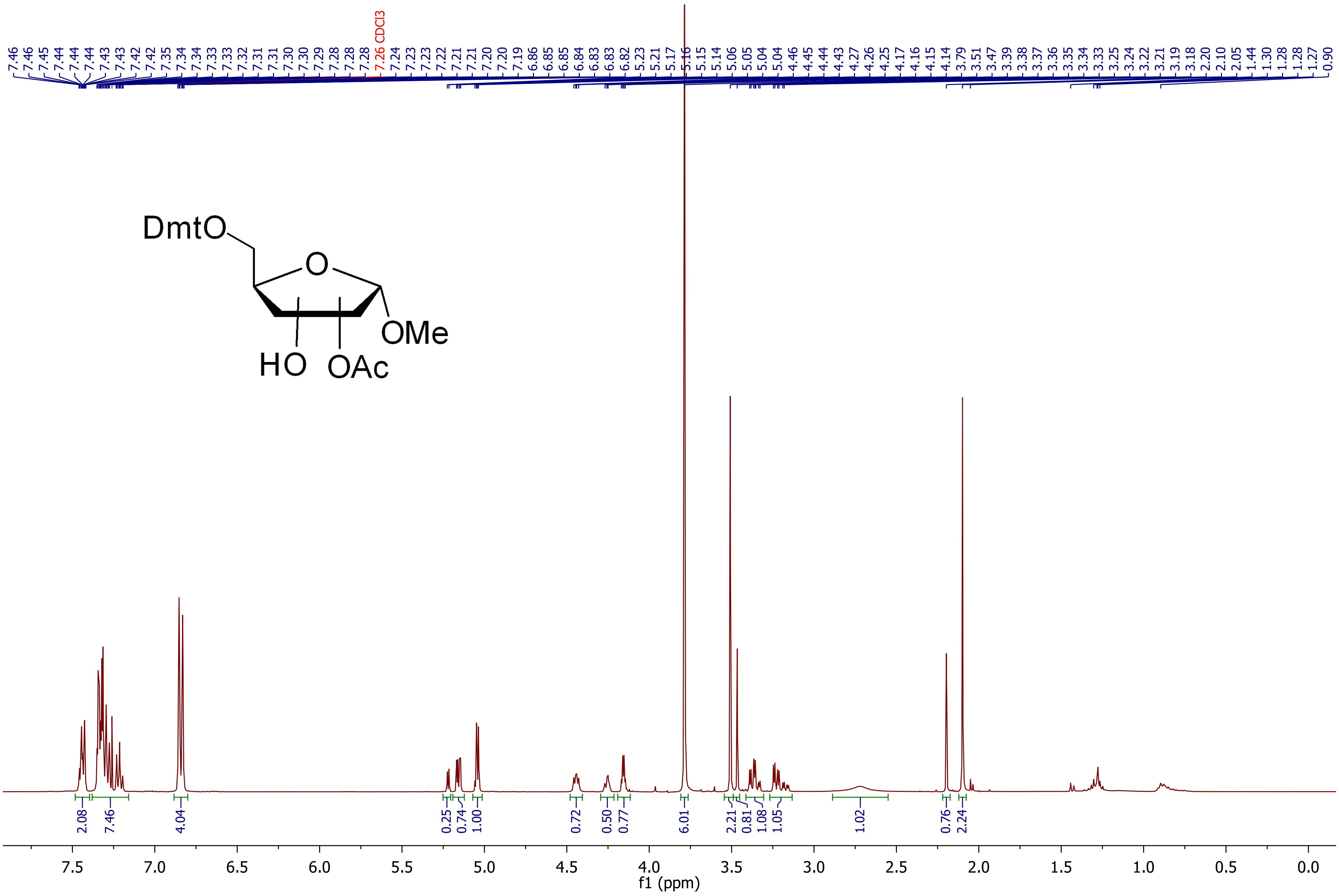

Compound **8**, ^{13}C--NMR, $CDCl_3$, 101 MHz

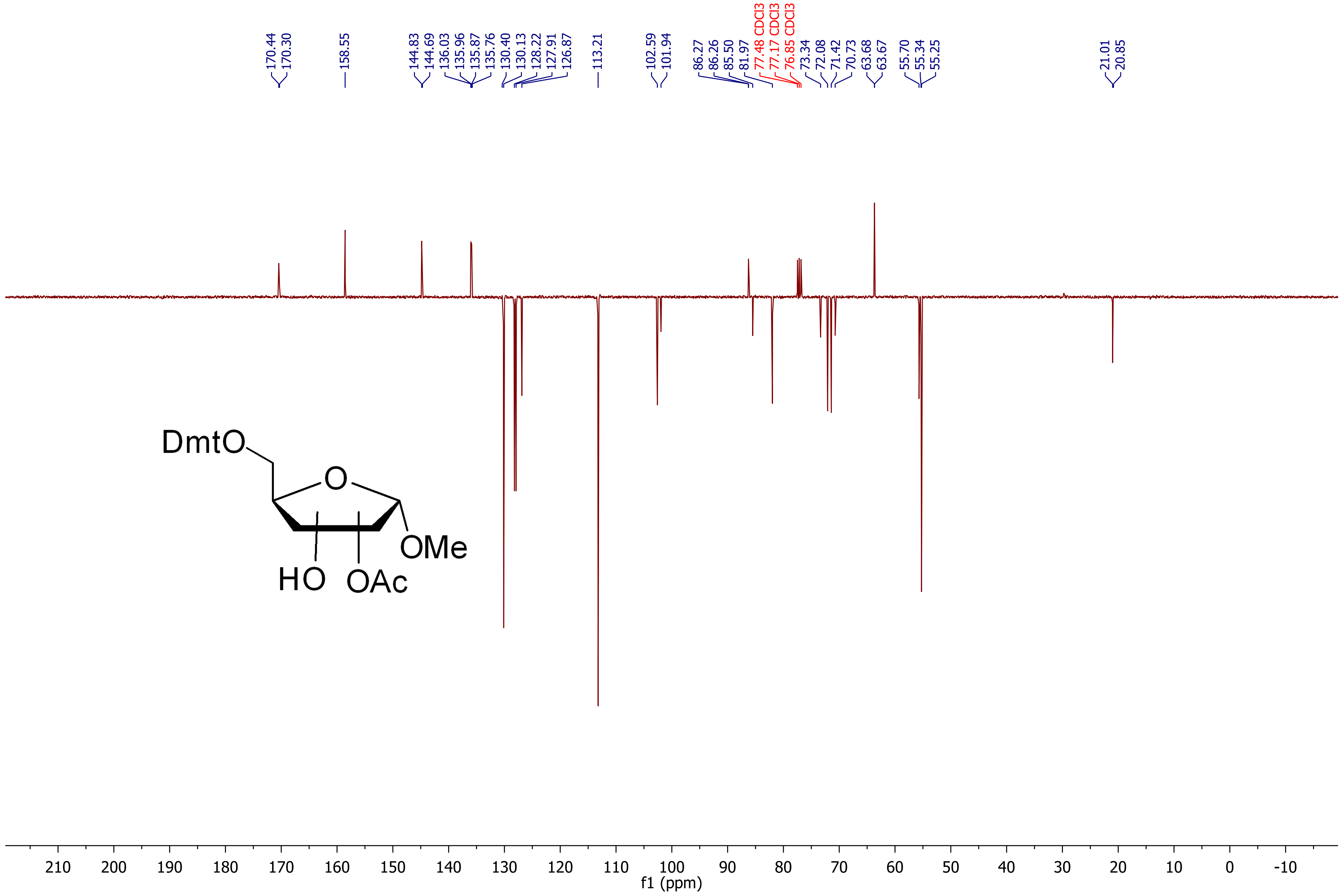

Compound **10**, ^{1}H--NMR, $CDCl_3$, 500 MHz

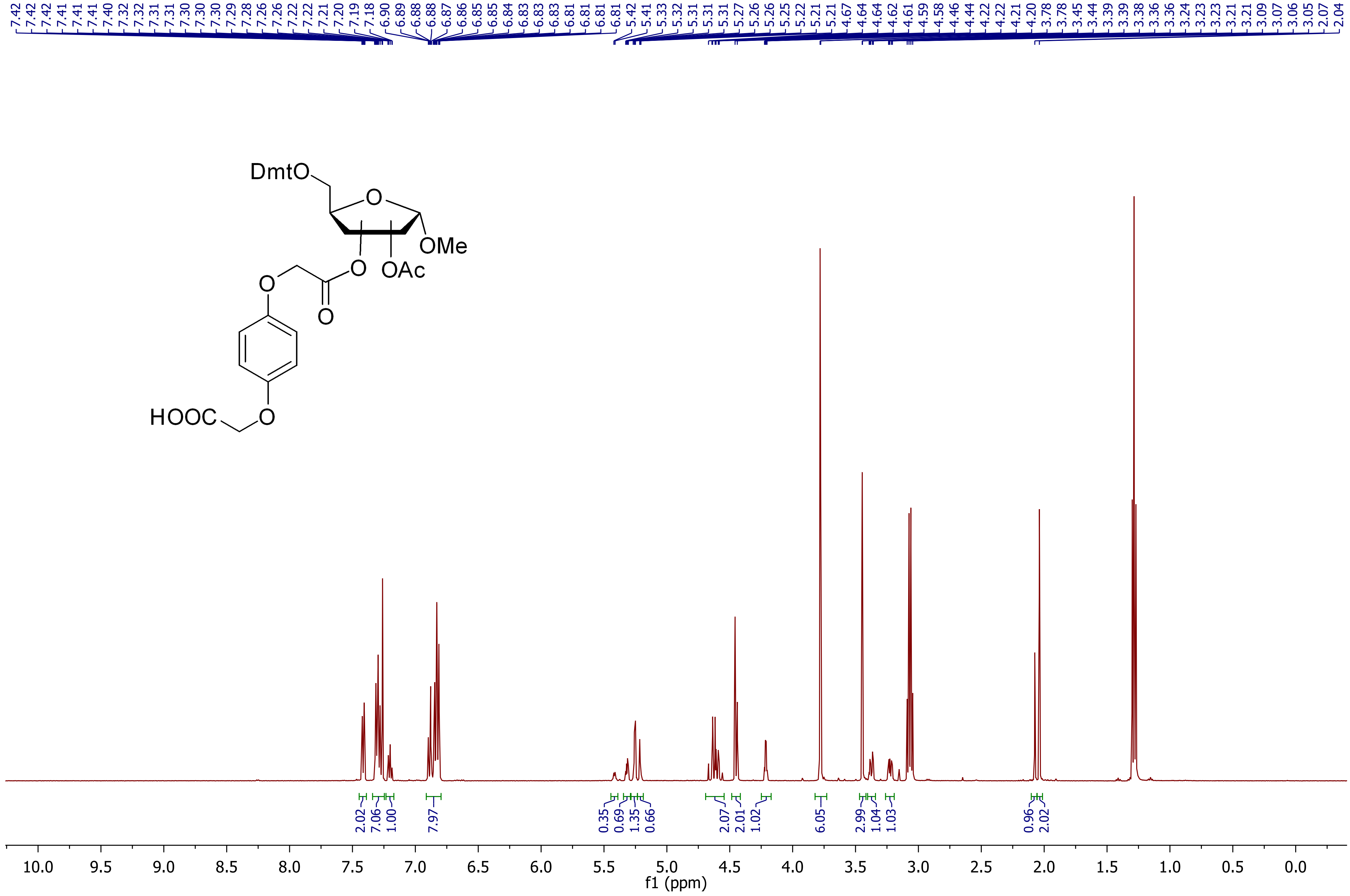

Compound **10**, ^{13}C--NMR, $CDCl_3$, 126 MHz

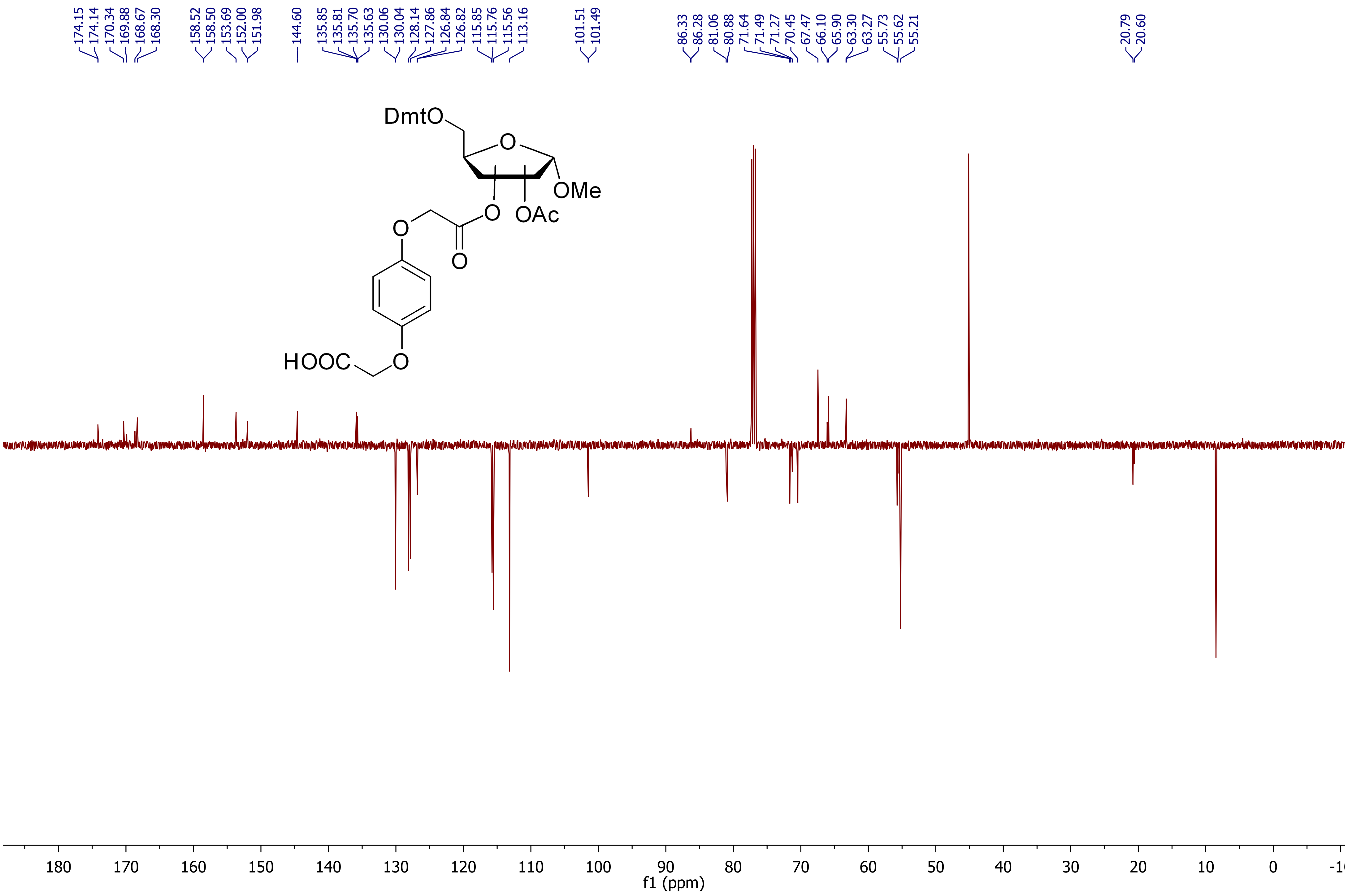

Compound **2m** (deprotected), ^{31}P--NMR, D_2O, 162 MHz

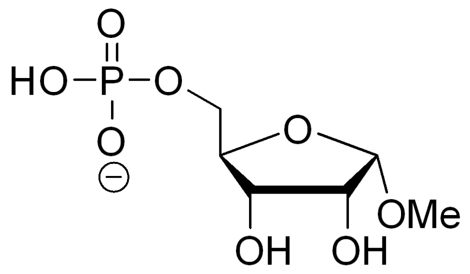

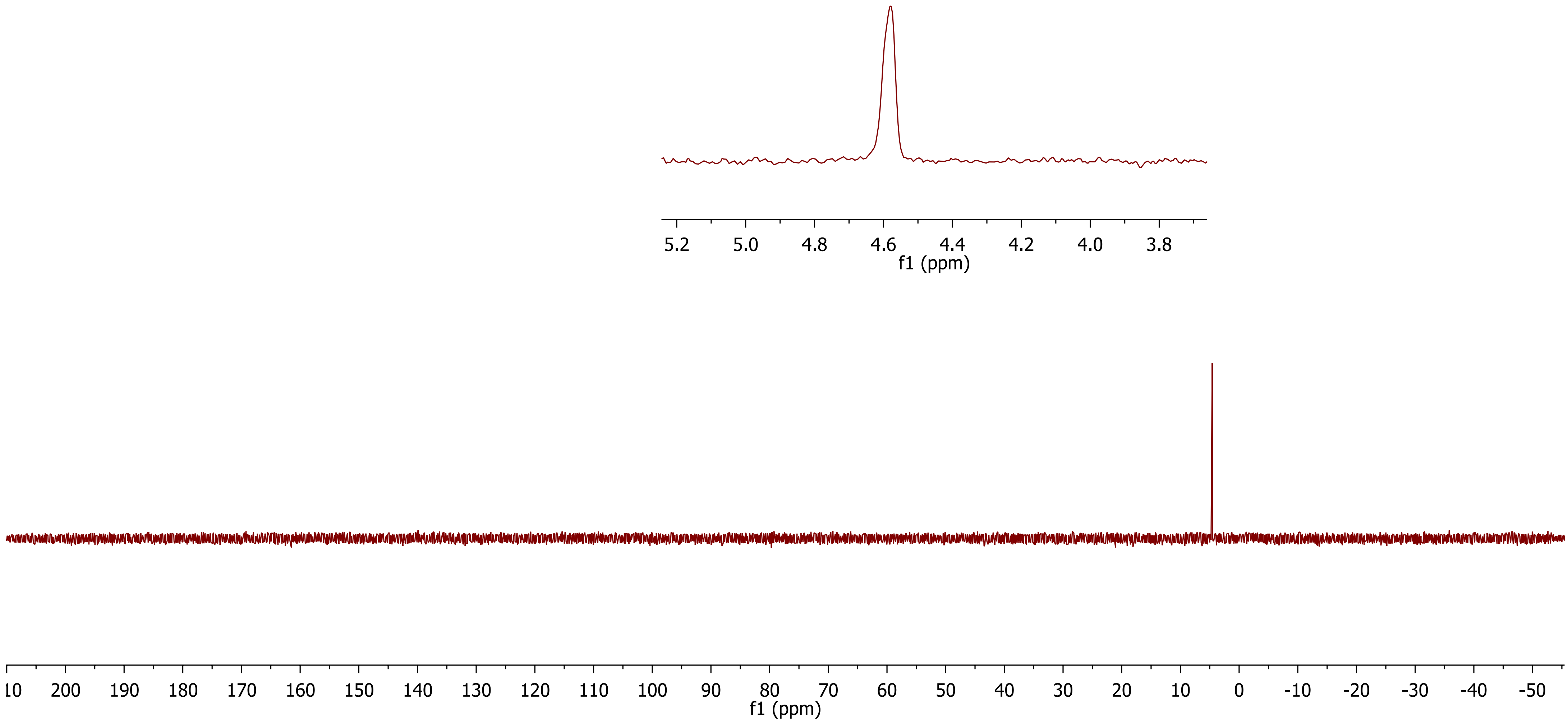

Compound **2k** (deprotected), ^{31}P--NMR, D_2O, 162 MHz

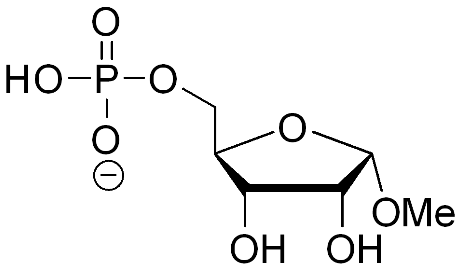

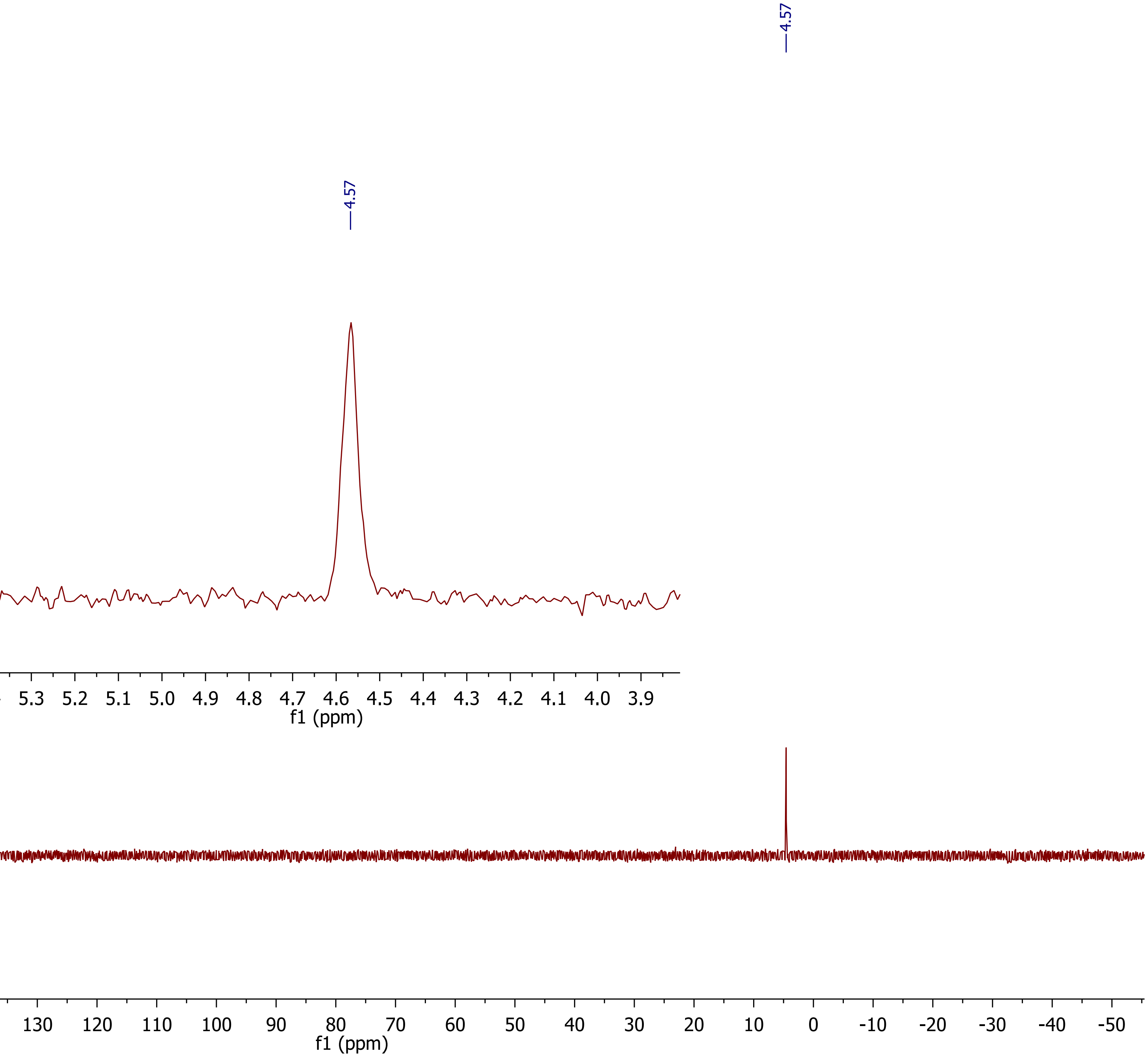

Compound **15**, ^{1}H--NMR, $CDCl_3$, 500 MHz

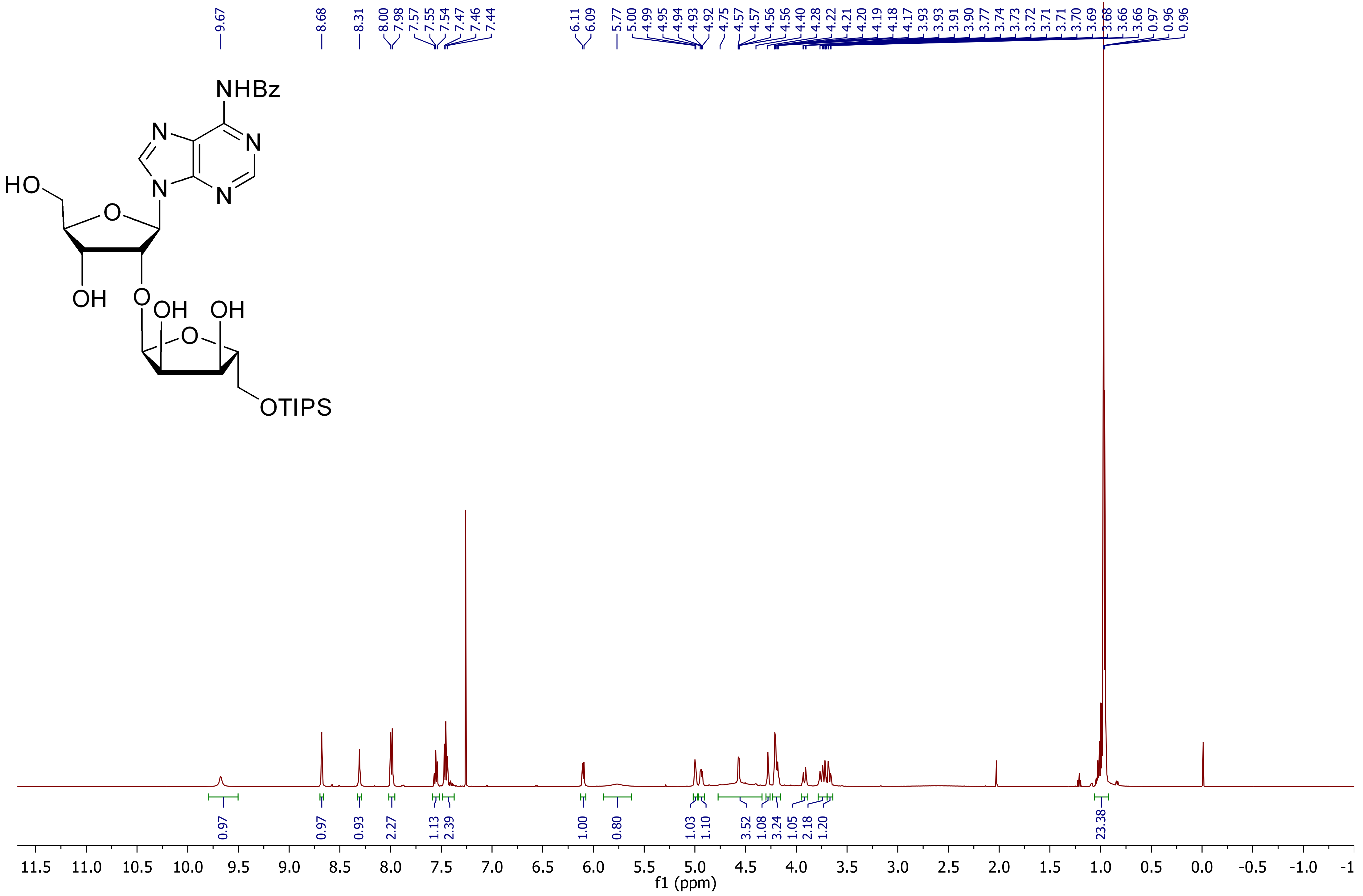

Compound **15**, ^{13}C--NMR, $CDCl_3$, 126 MHz

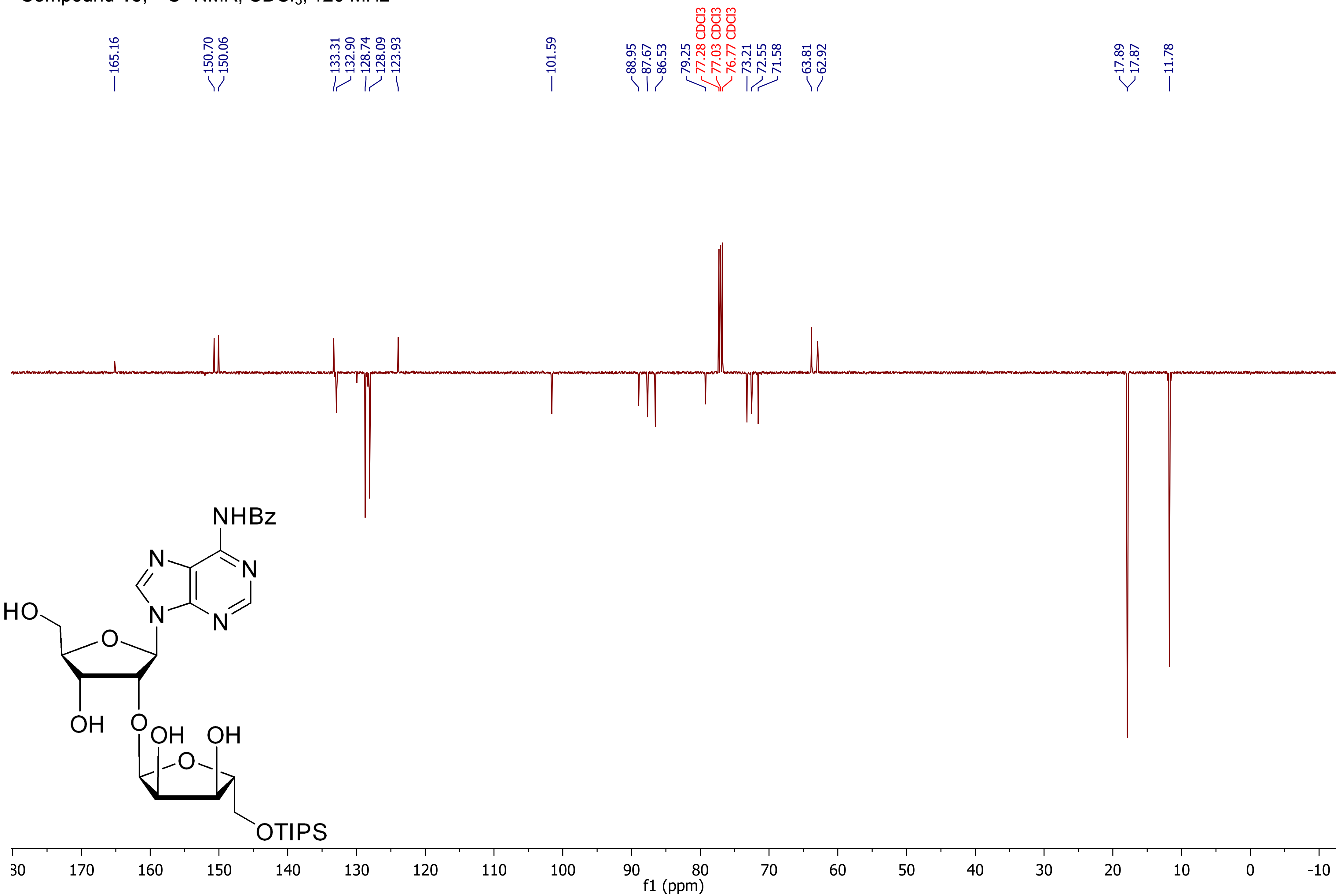

Compound **16**, ^{1}H--NMR, $CDCl_3$, 500 MHz

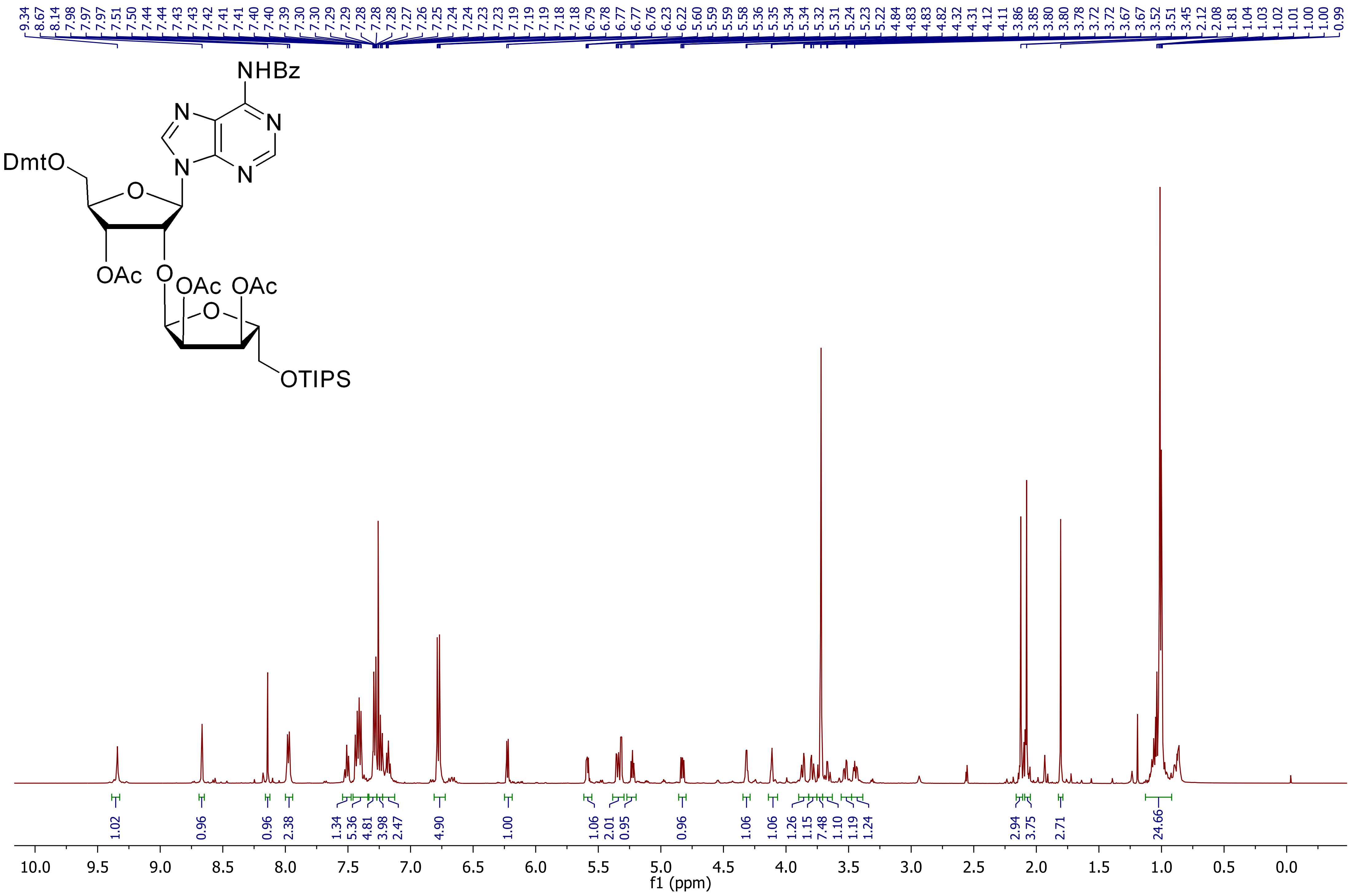

Compound **16**, ^{13}C--NMR, $CDCl_3$, 126 MHz

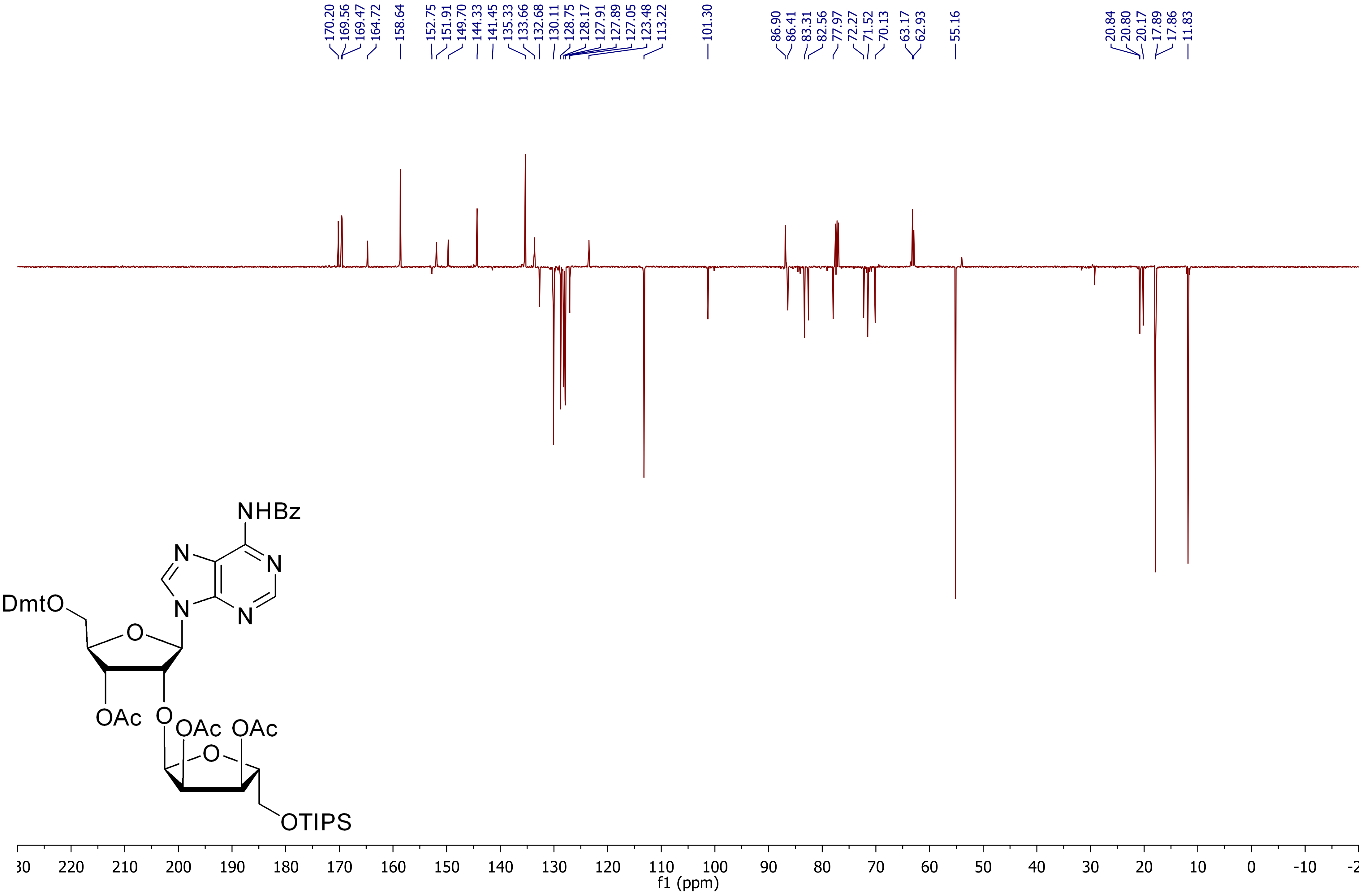

Compound **17**, ^{1}H--NMR, $CDCl_3$, 400 MHz

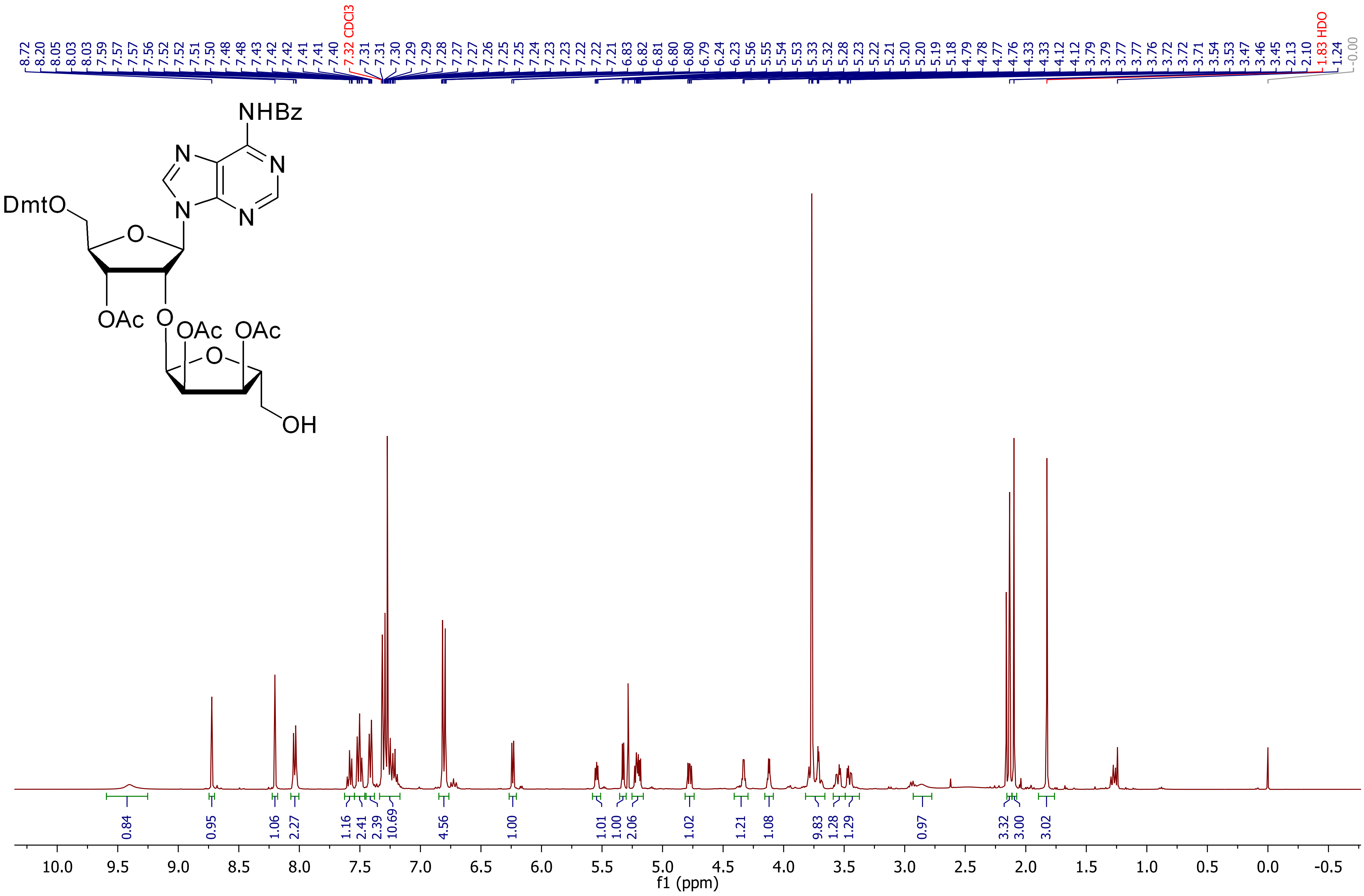

Compound **17**, ^{13}C--NMR, $CDCl_3$, 101 MHz

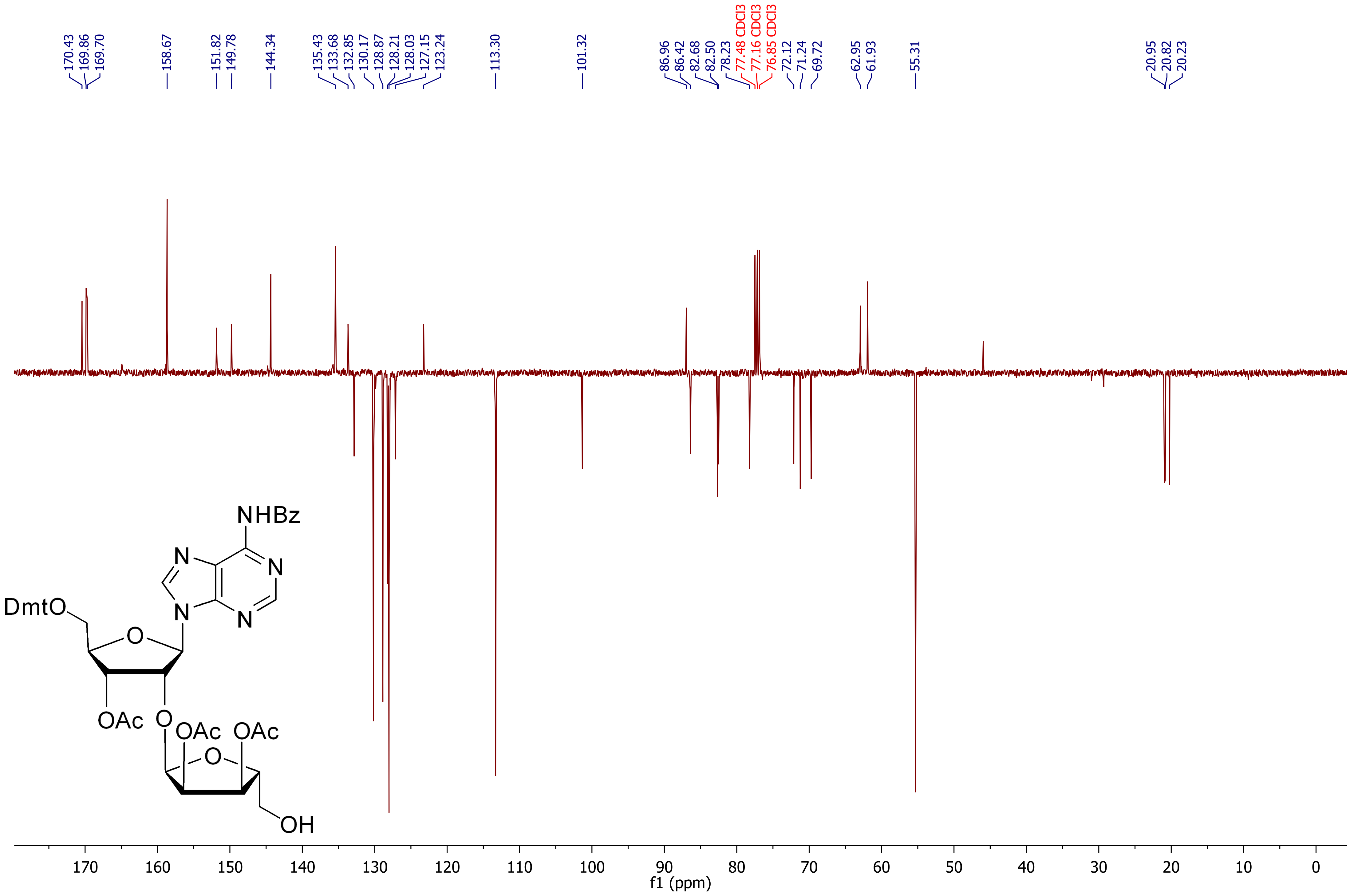

Compound **18**, ^{1}H--NMR, $CDCl_3$, 500 MHz

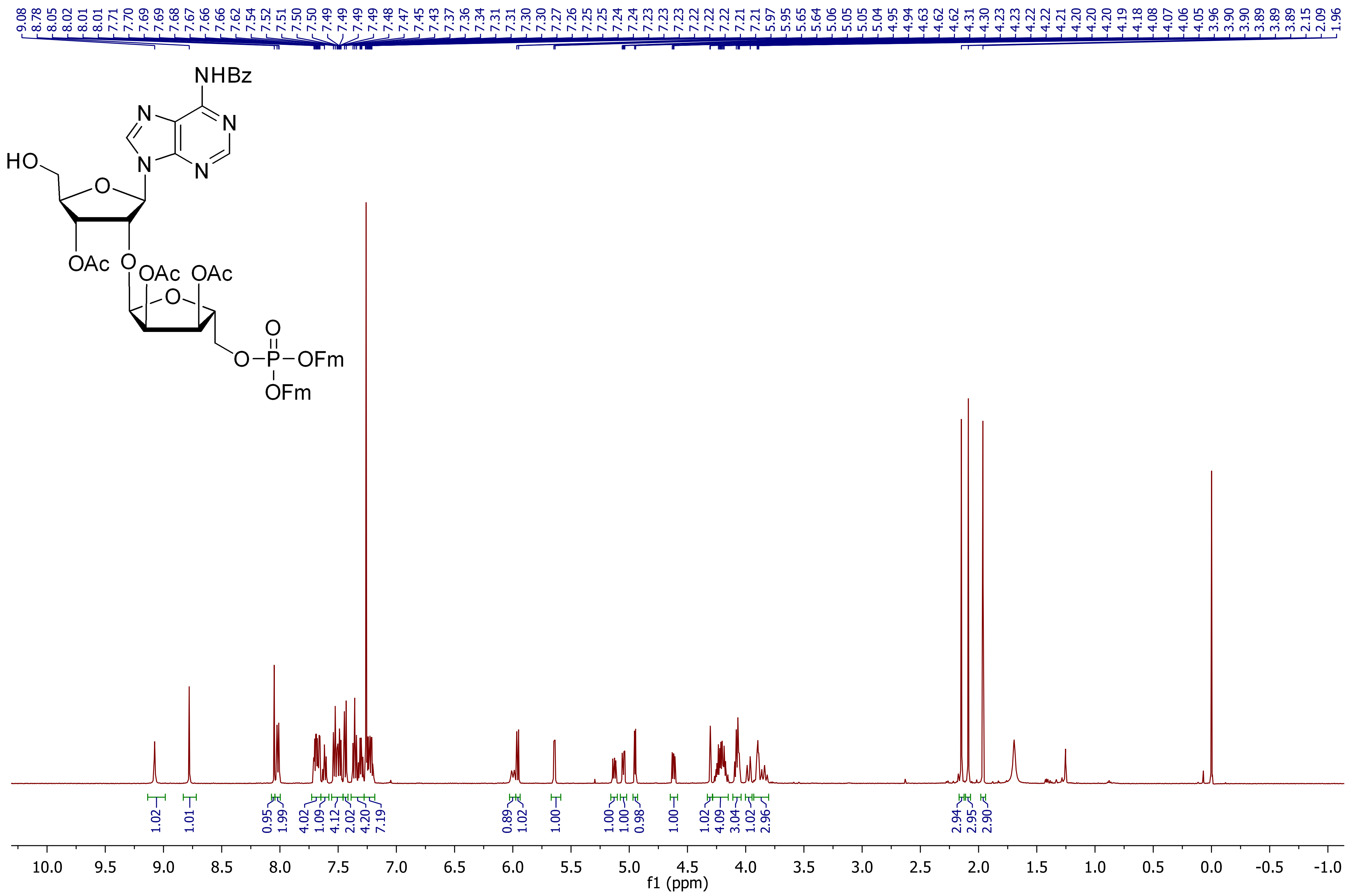

Compound **18**, ^{13}C--NMR, $CDCl_3$, 126 MHz

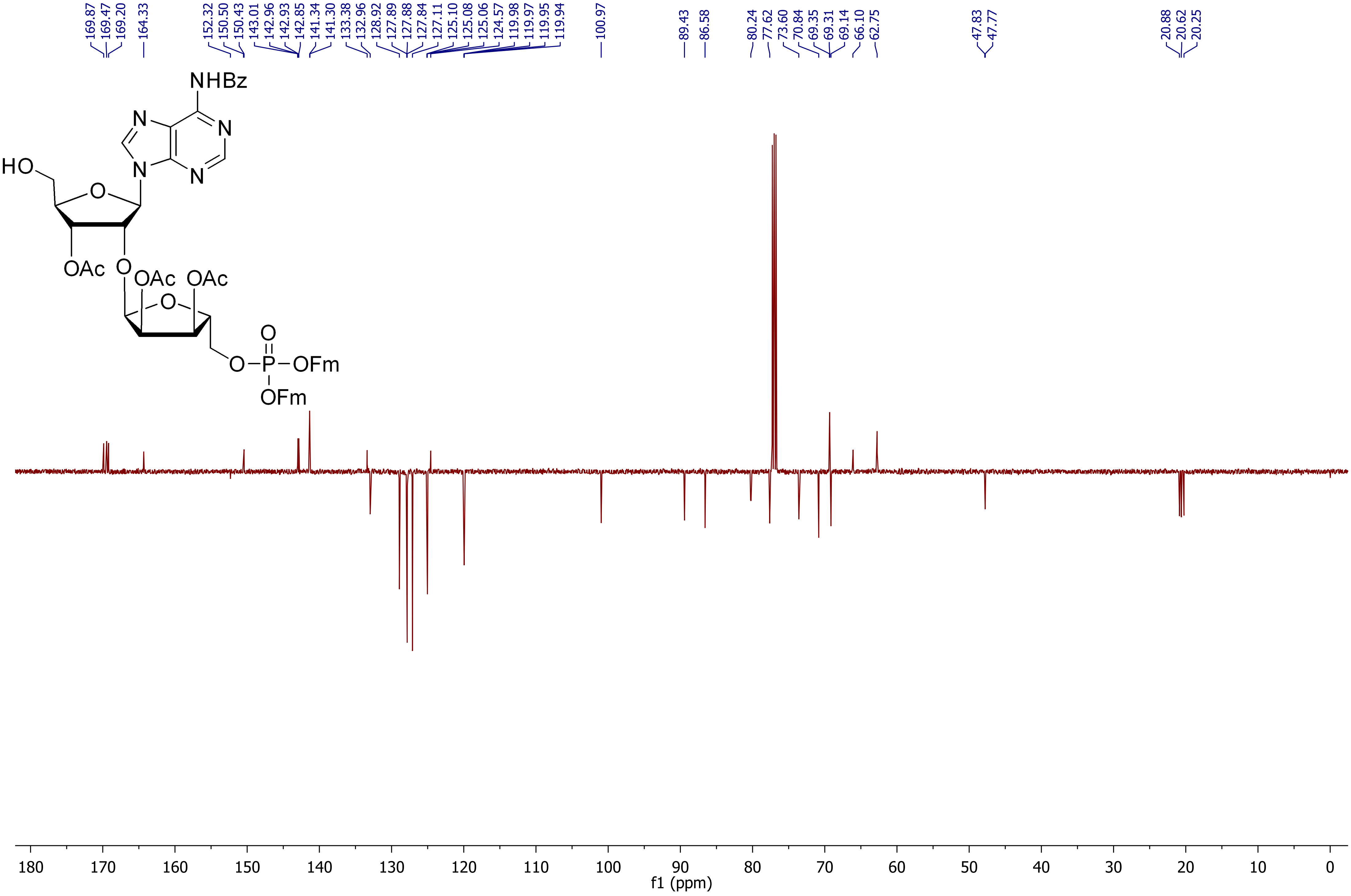

Compound **18**, ^{31}P--NMR, $CDCl_3$, 202 MHz

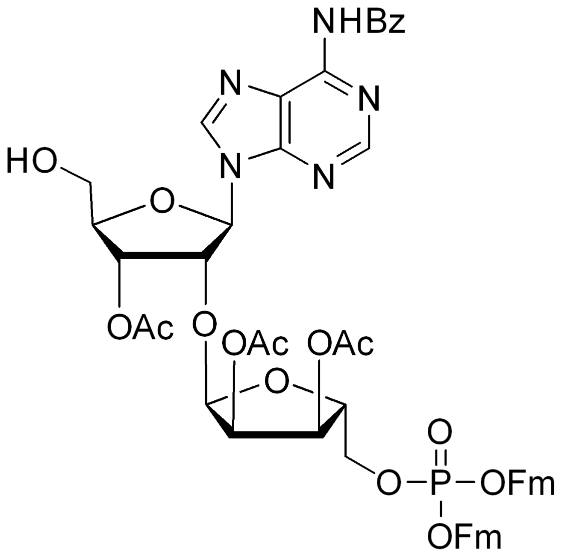

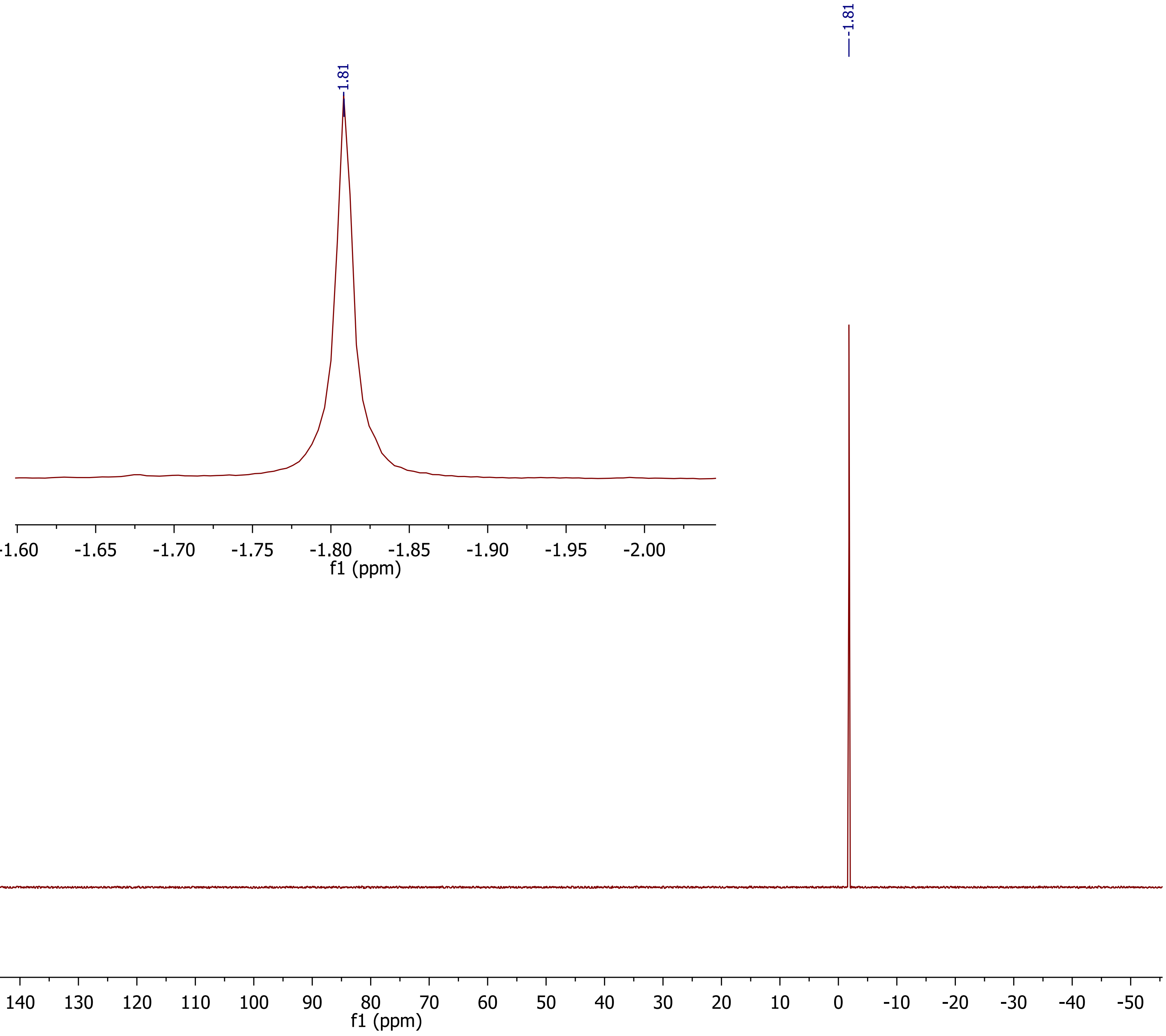

Compound **3**, ^{1}H--NMR, $CDCl_3$, 500 MHz

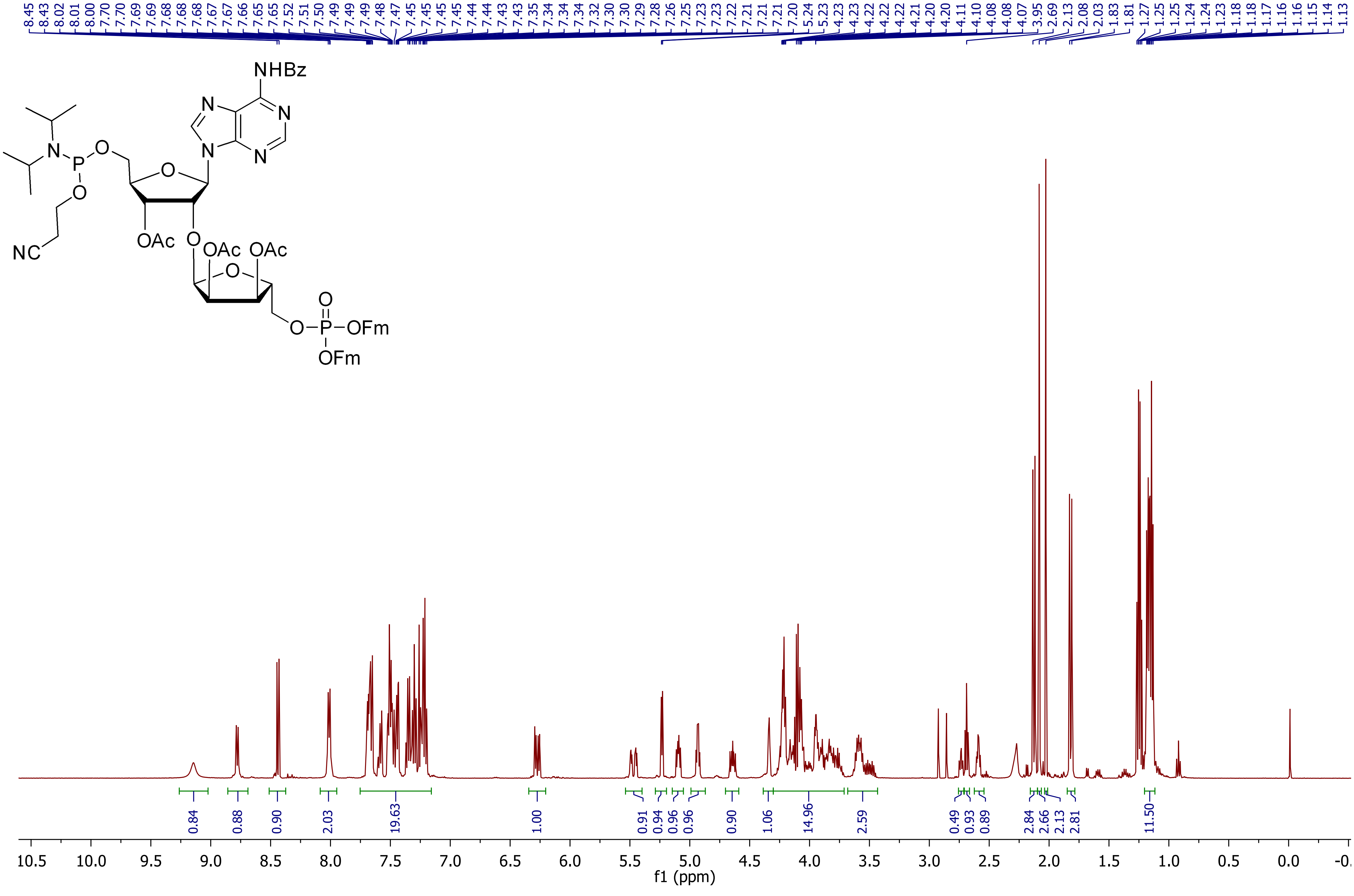

Compound **3**, ^{13}C--NMR, $CDCl_3$, 126 MHz

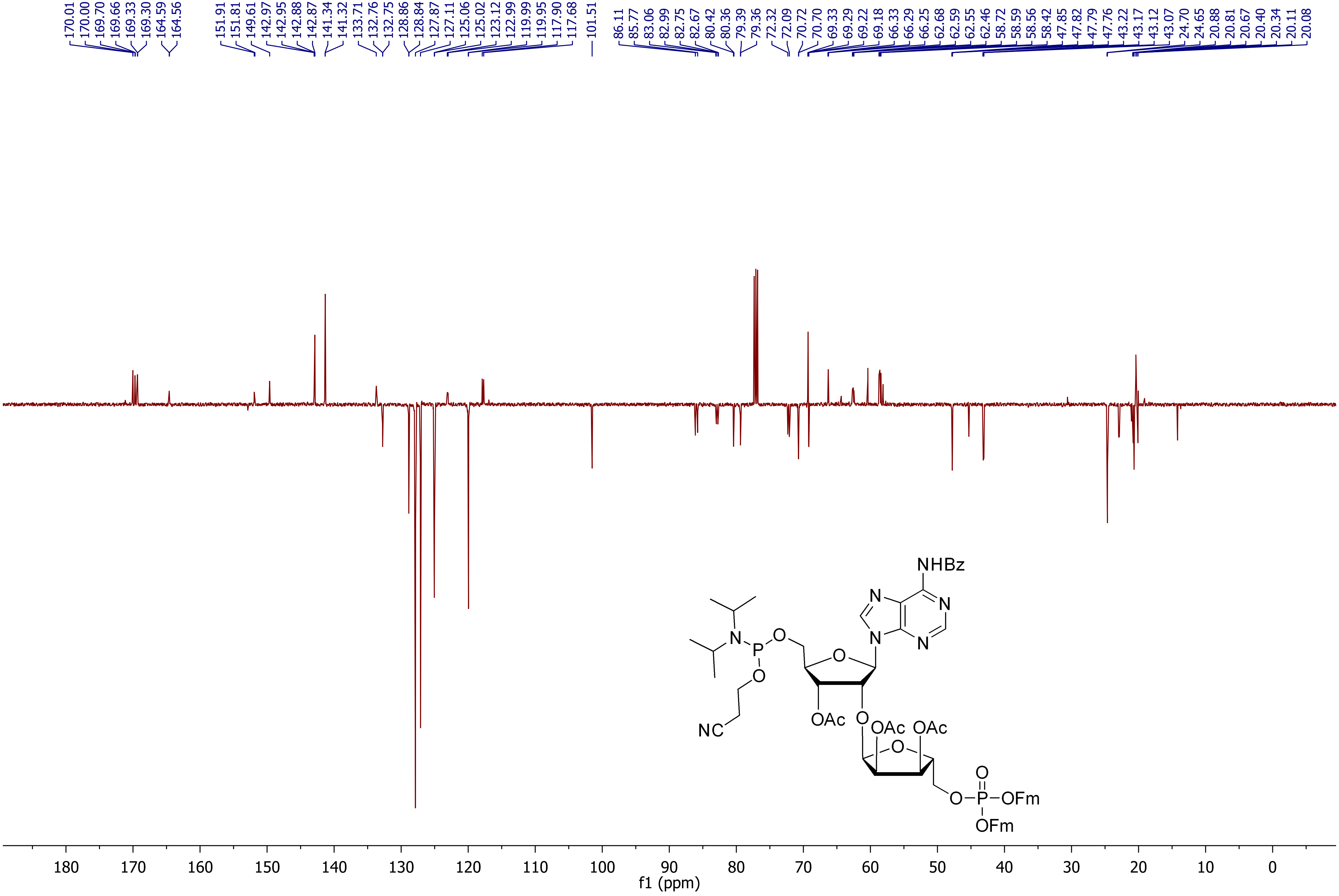

Compound **3**, ^{31}P--NMR, $CDCl_3$, 202 MHz

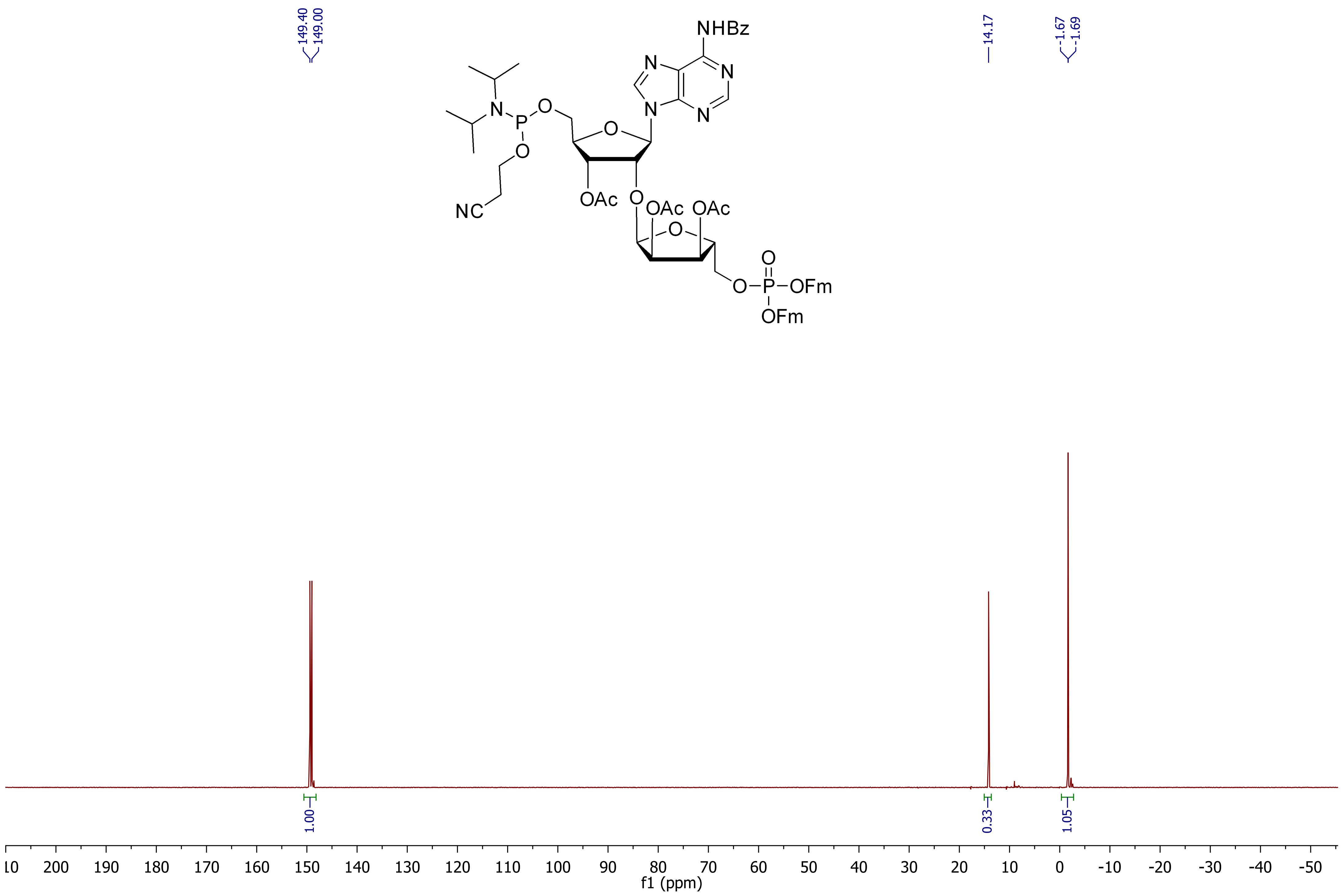

Compound **1a**, ^{1}H--NMR, D_2O, 850 MHz

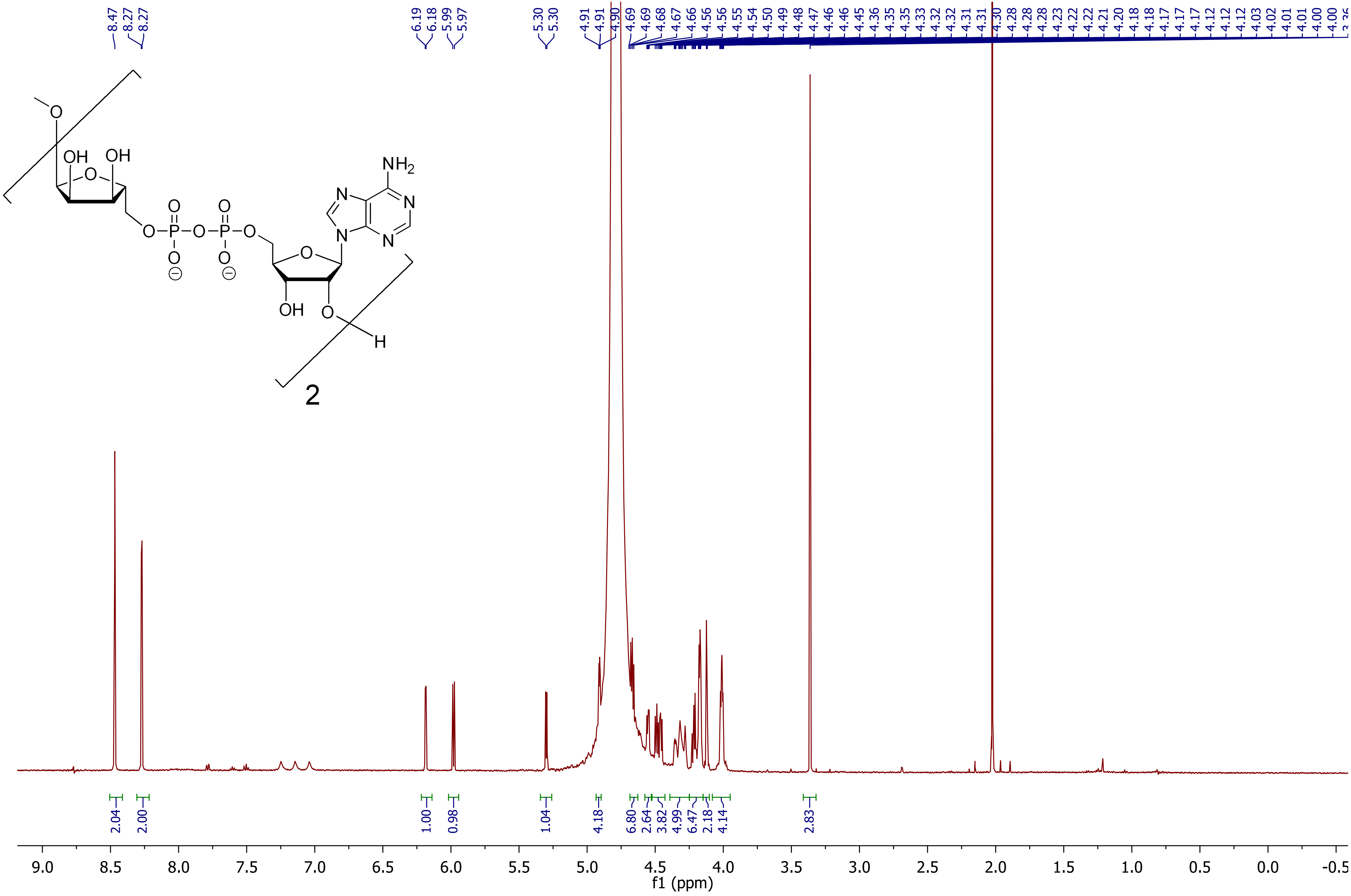

Compound **1a**, ^{1}H--NMR, D_2O, H_2O suppression, 850 MHz

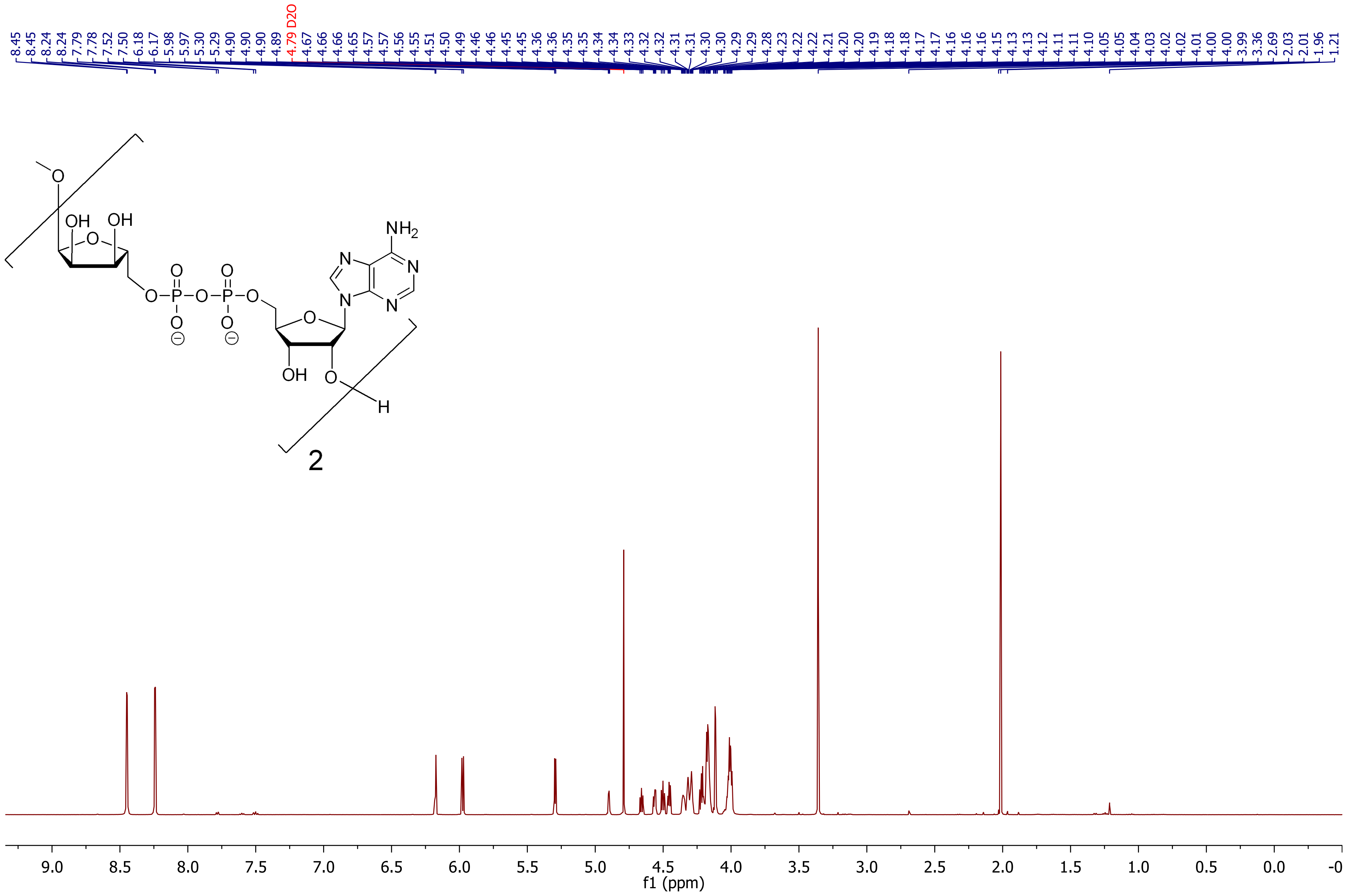

Compound **1a**, ^{13}C--NMR, D_2O, 202 MHz

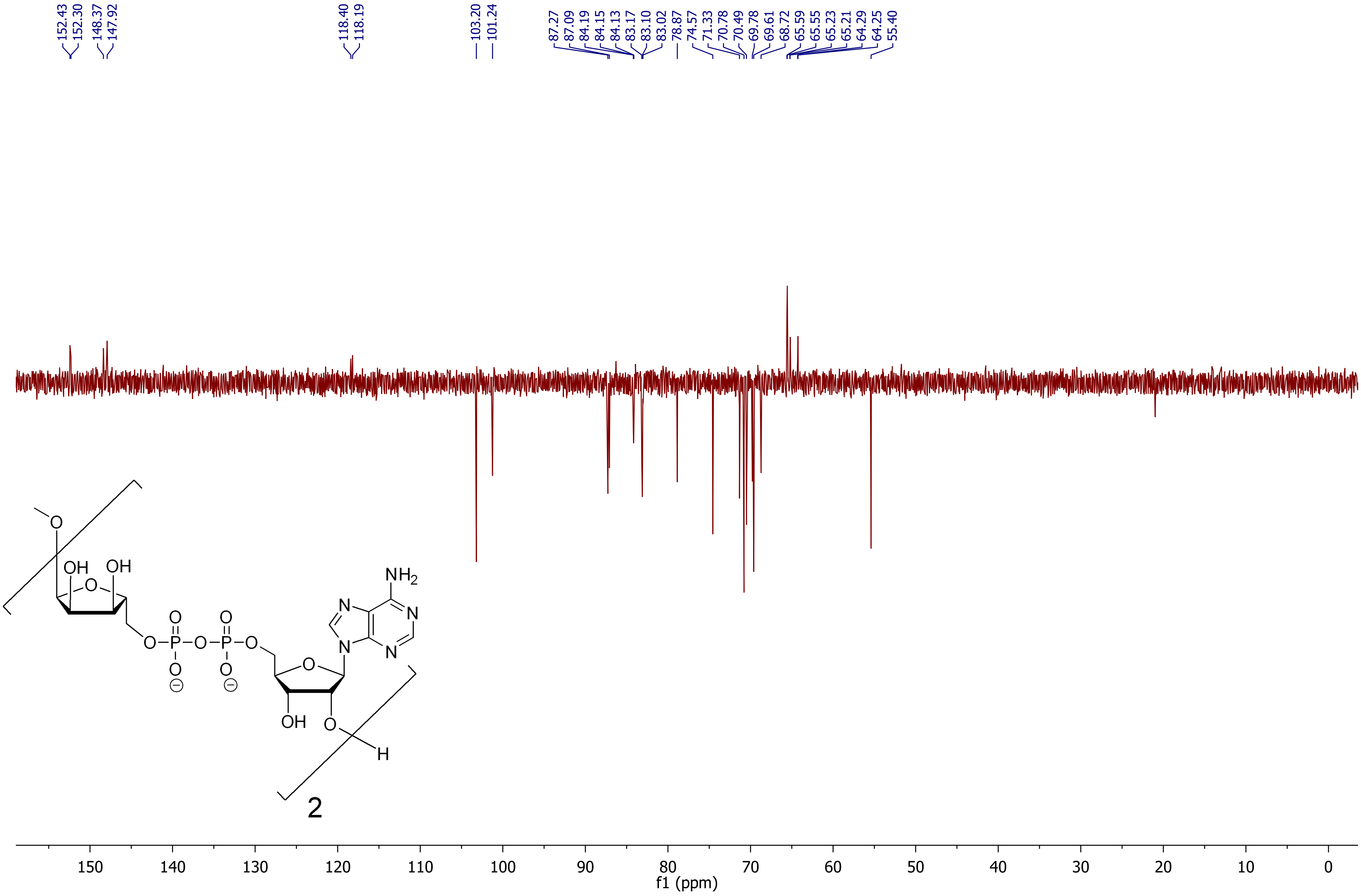

Compound **1a**, ^{31}P--NMR, D_2O, 202 MHz

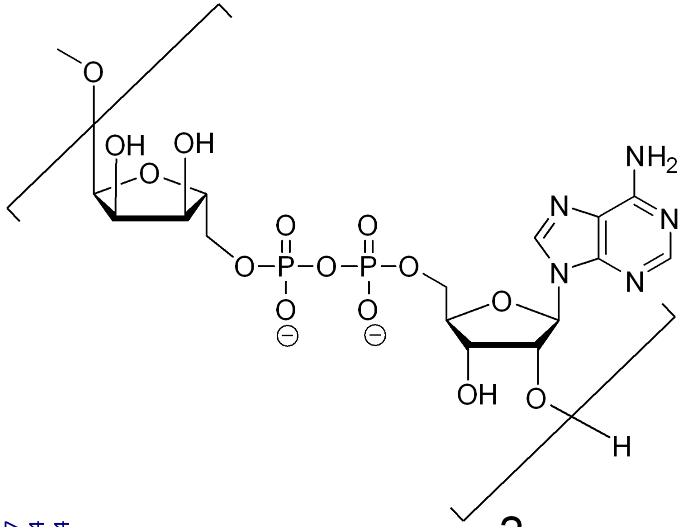

2

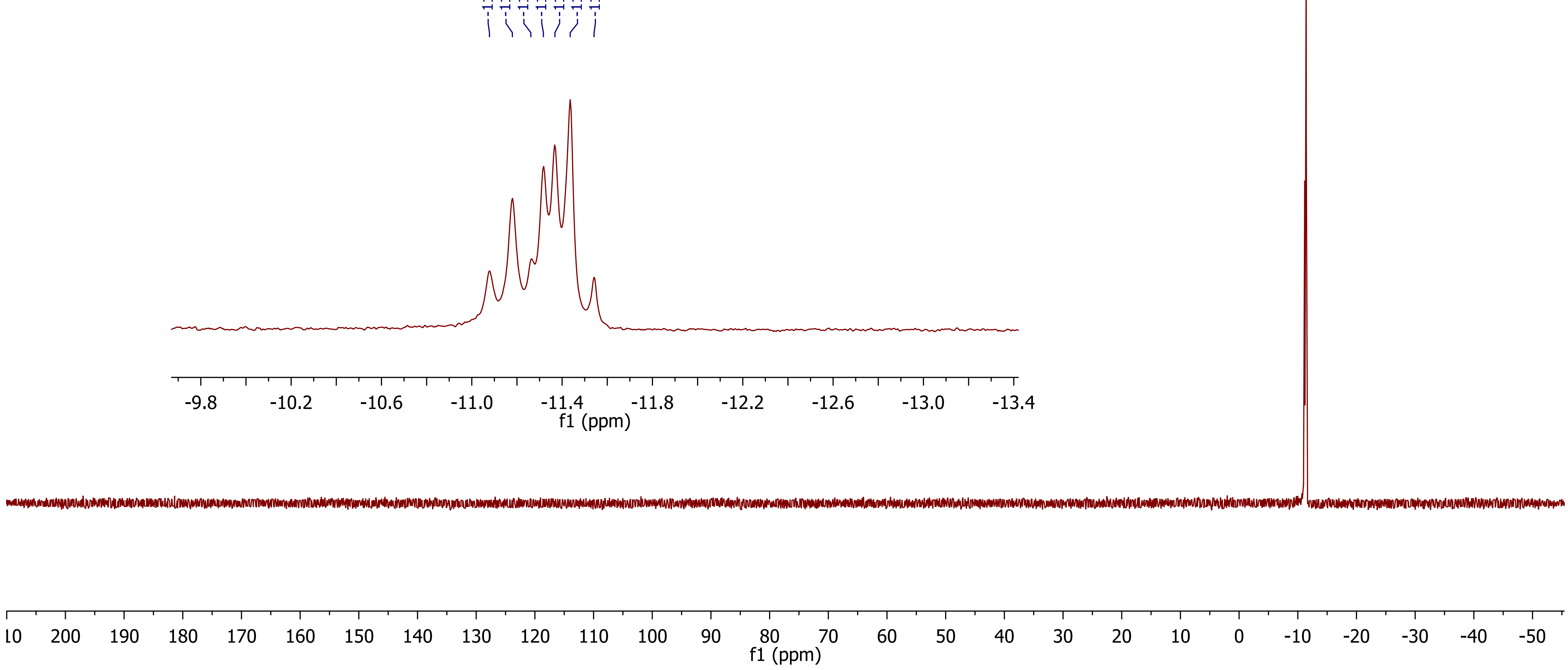

Compound **1a**, COSY, D_2O, 850 MHz

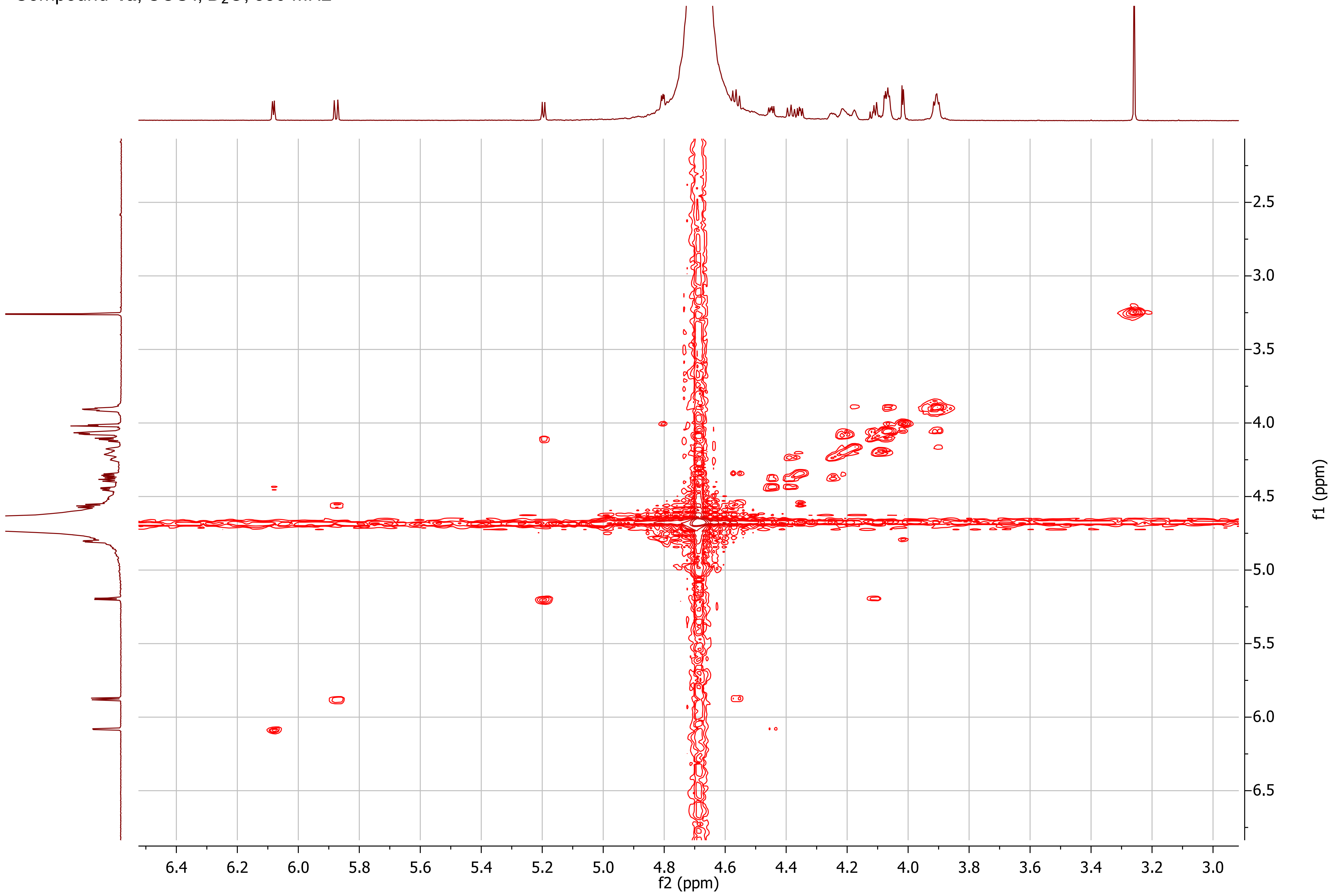

Compound **1a**, HSQC, D_2O, 850 MHz

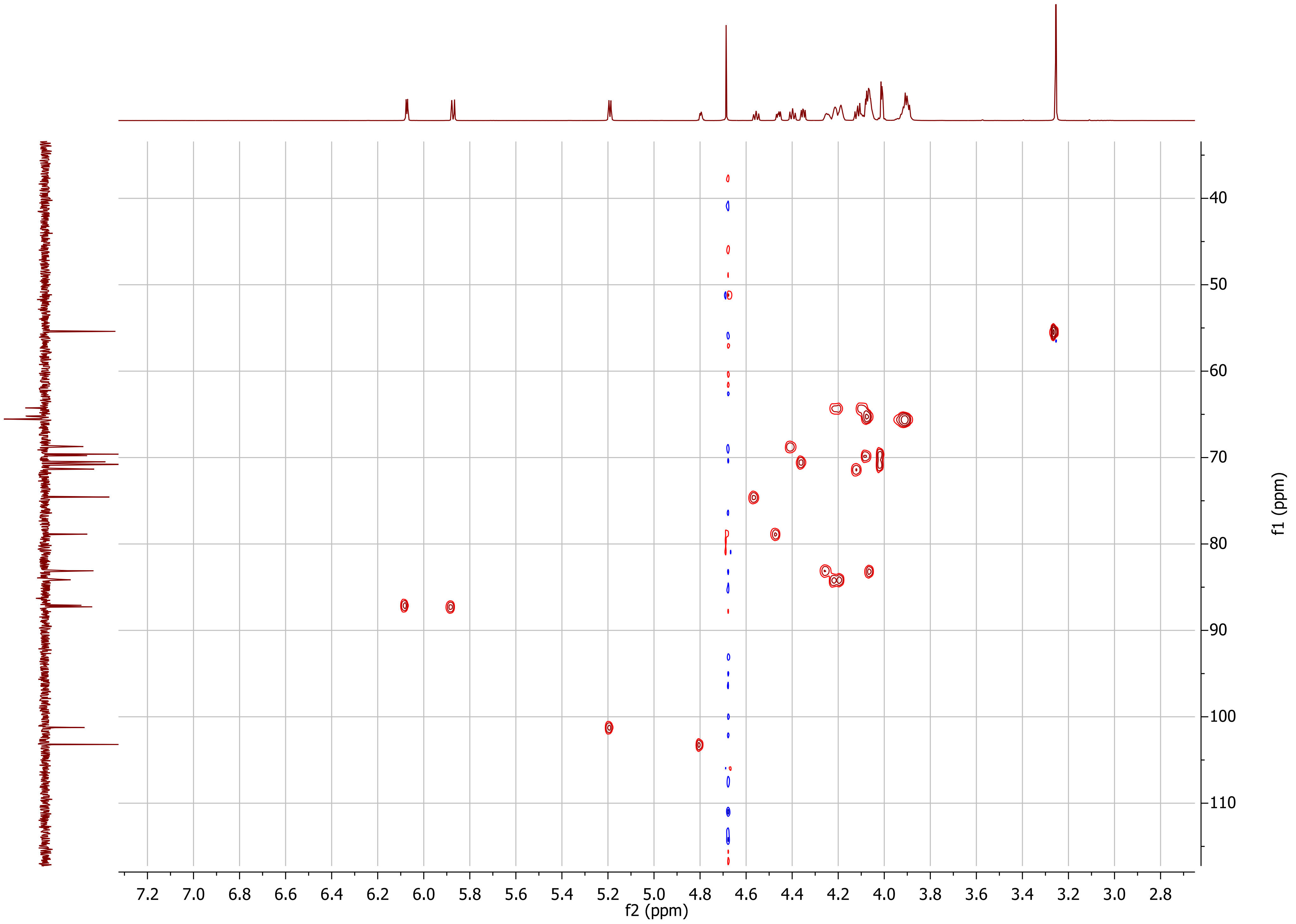

Compound **1b**, ^{1}H--NMR, D_2O, 500 MHz

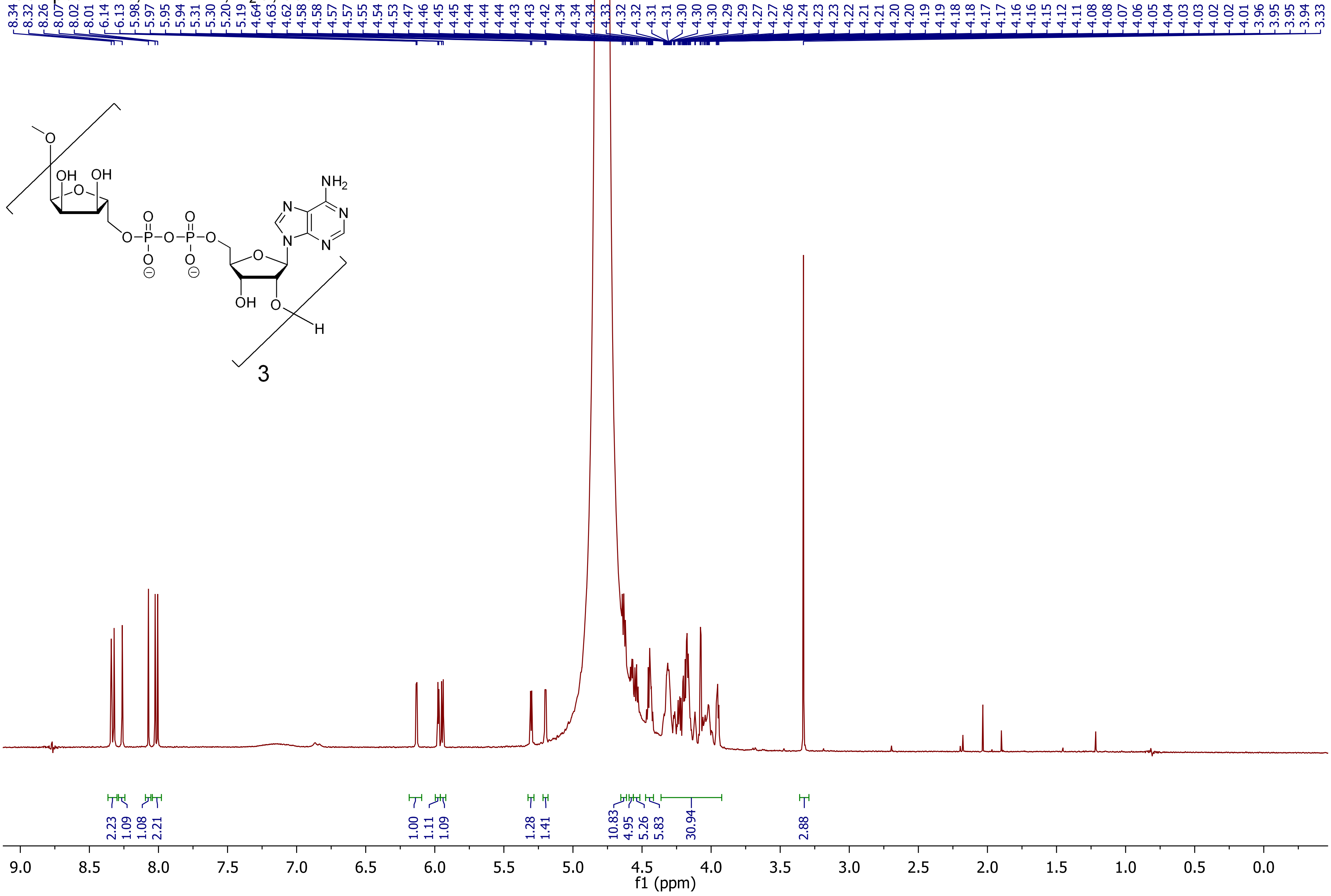

Compound **1b**, ^{1}H--NMR, D_2O, H_2O suppression, 500 MHz

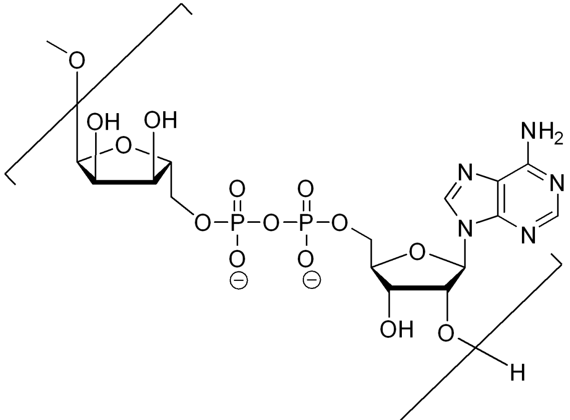

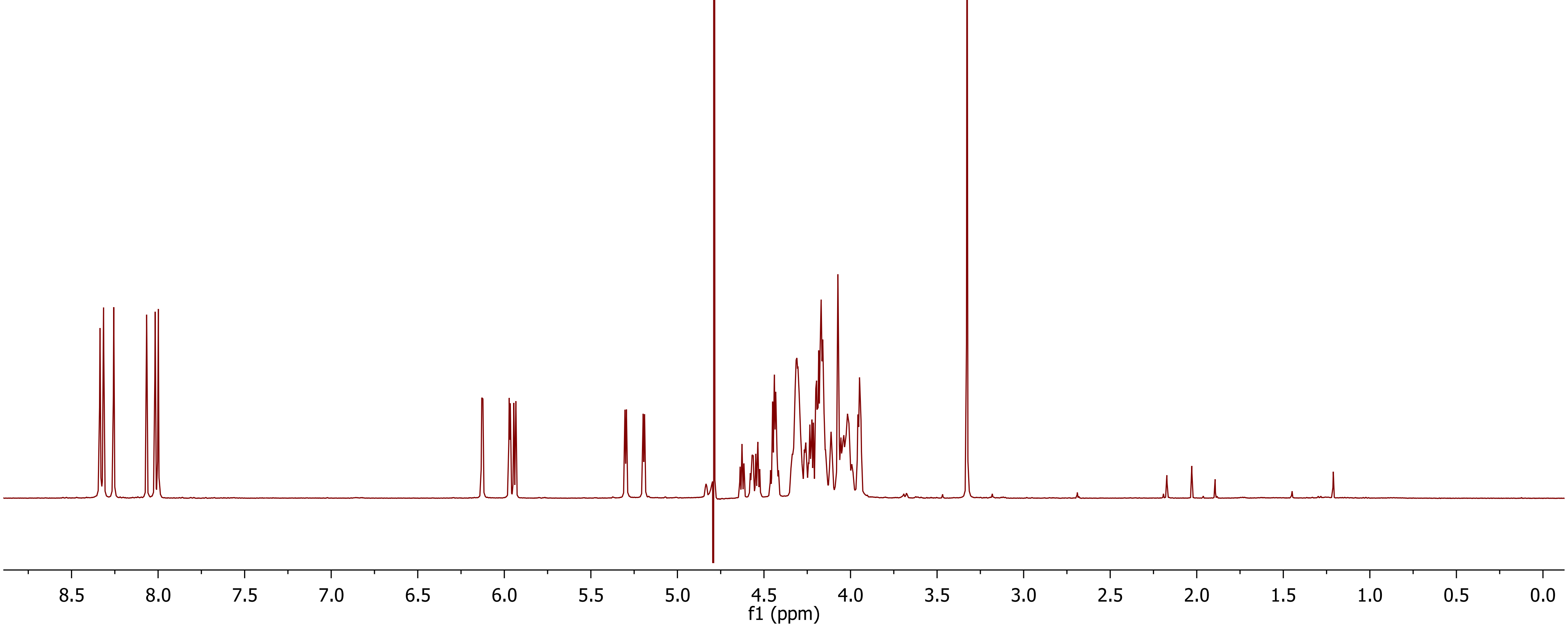

Compound **1b**, ^{13}C--NMR, D_2O, 214 MHz

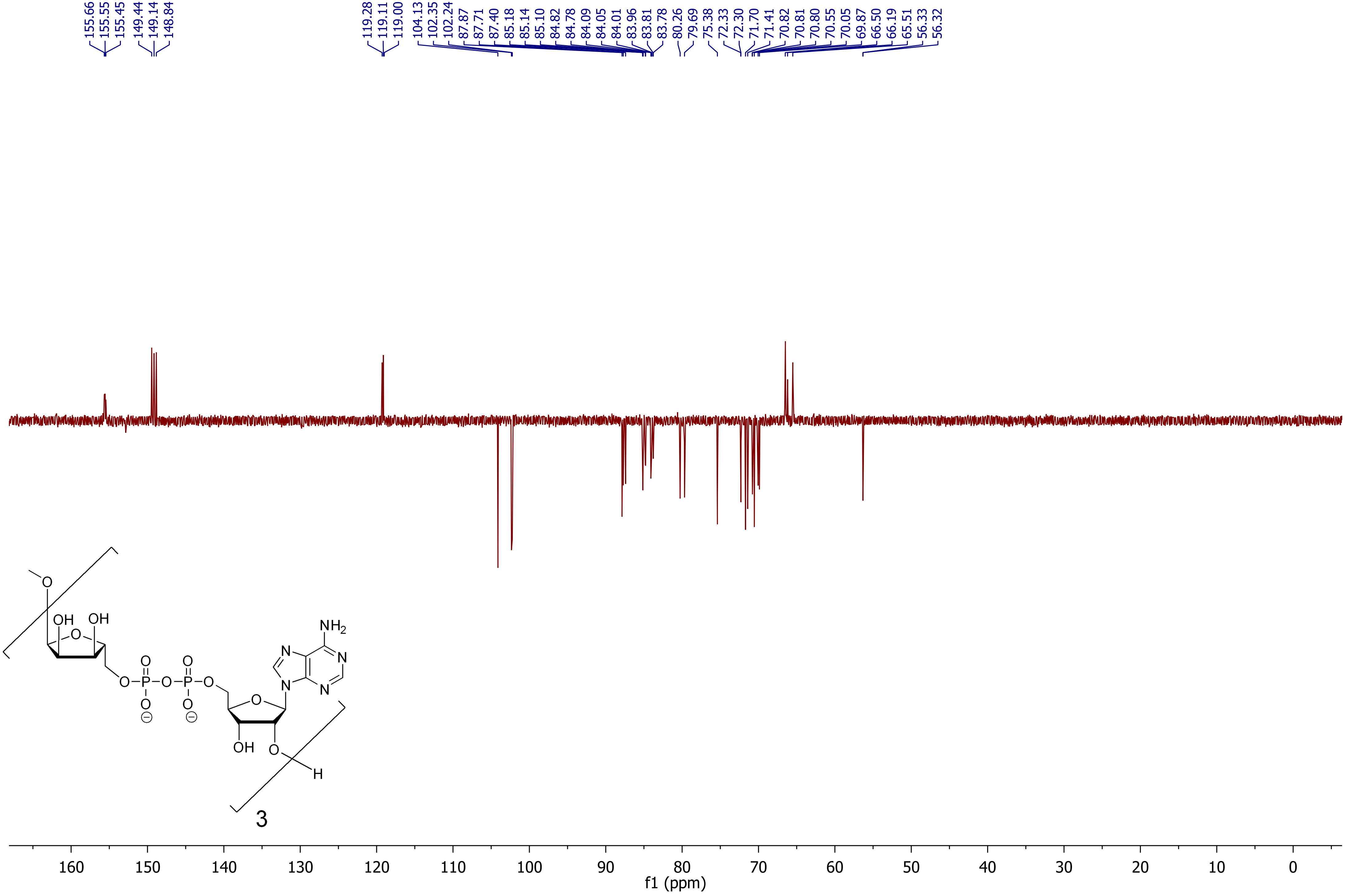

Compound **1b**, ^{31}P--NMR, D_2O, 202 MHz

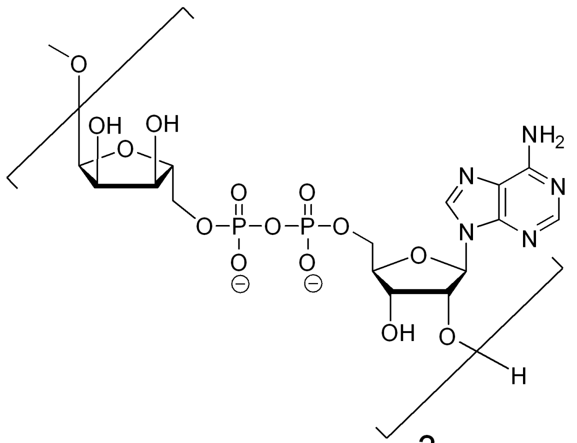

3

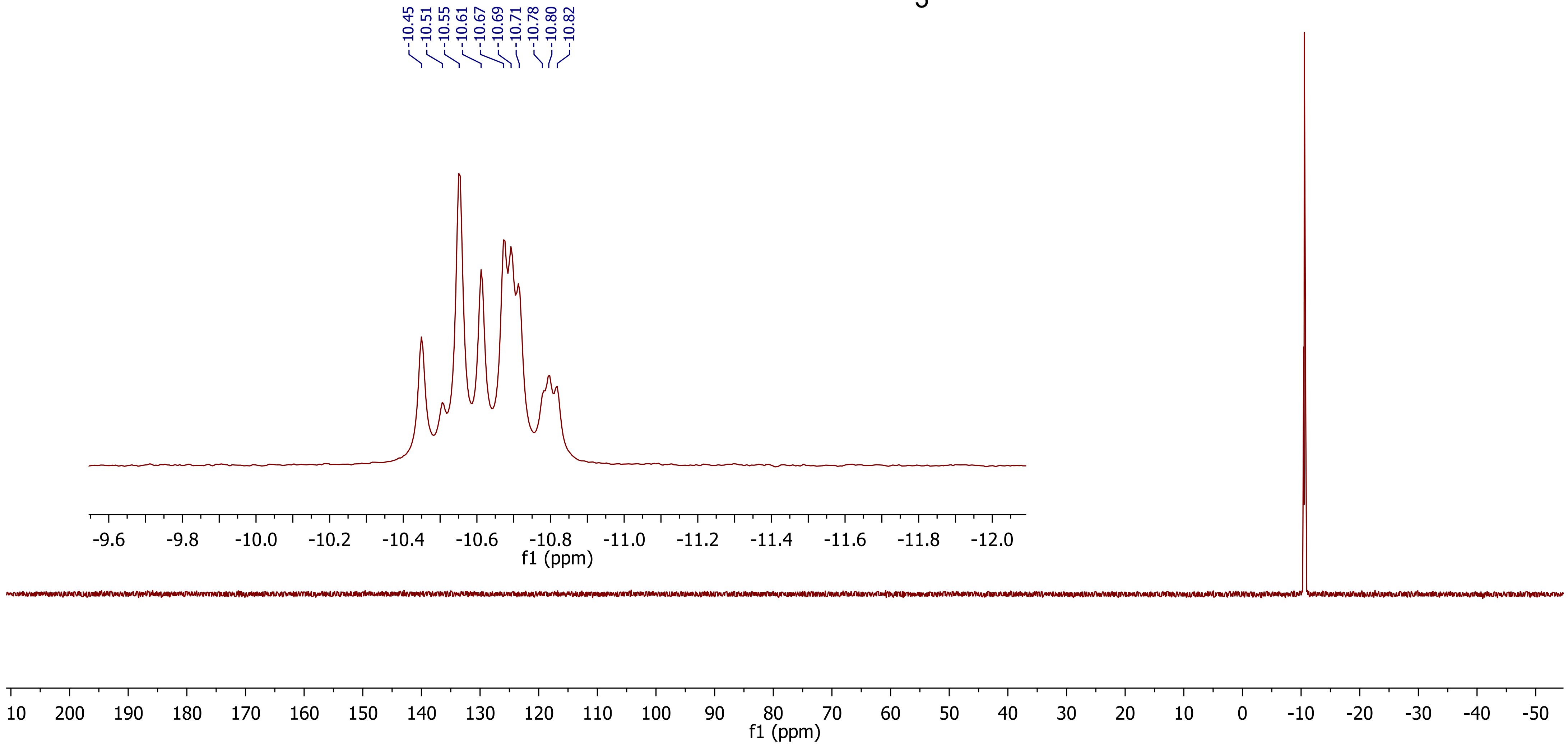

Compound **1b**, COSY, D_2O, 850 MHz

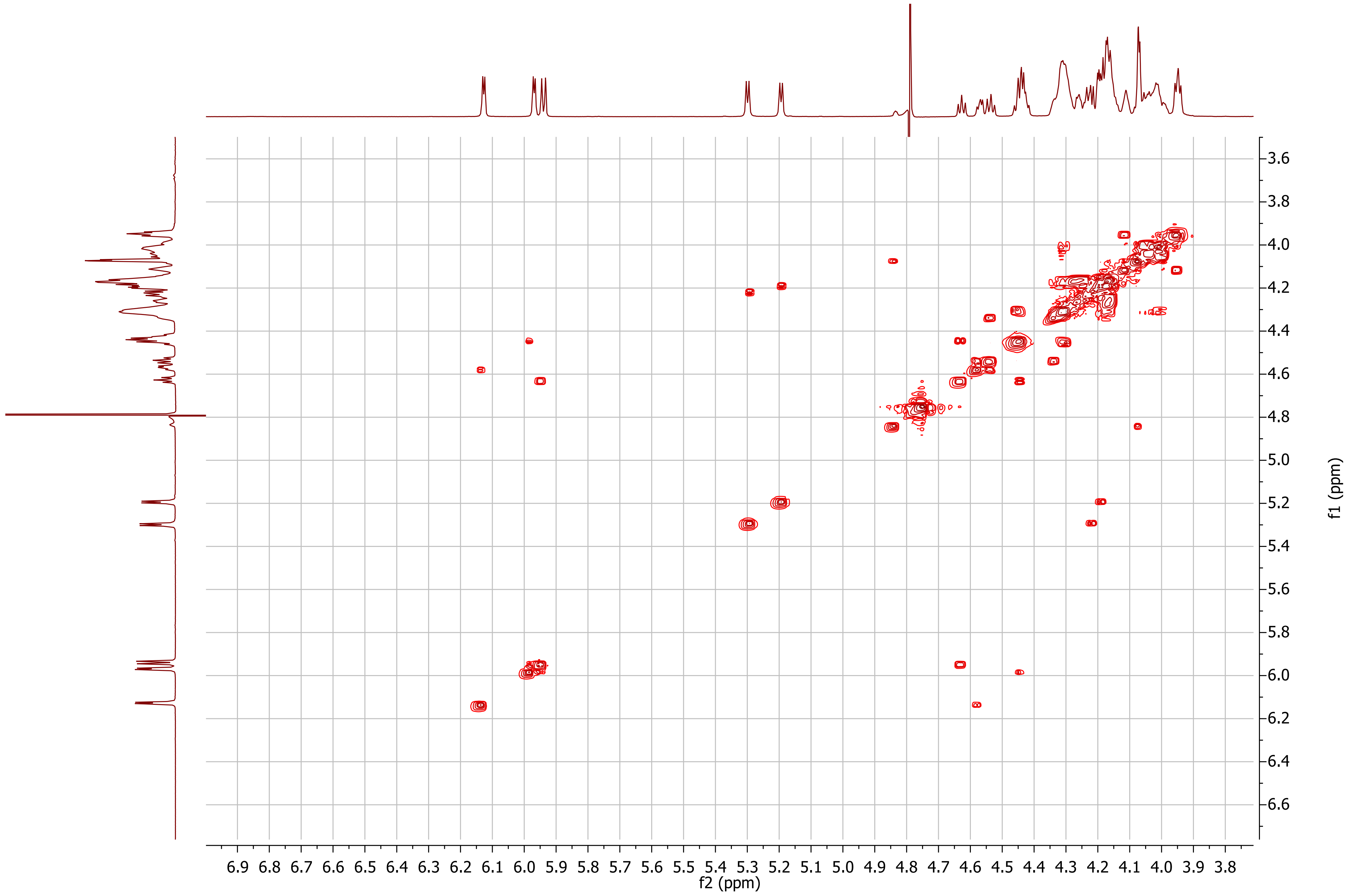

Compound **1b**, HSQC, D_2O, 850 MHz

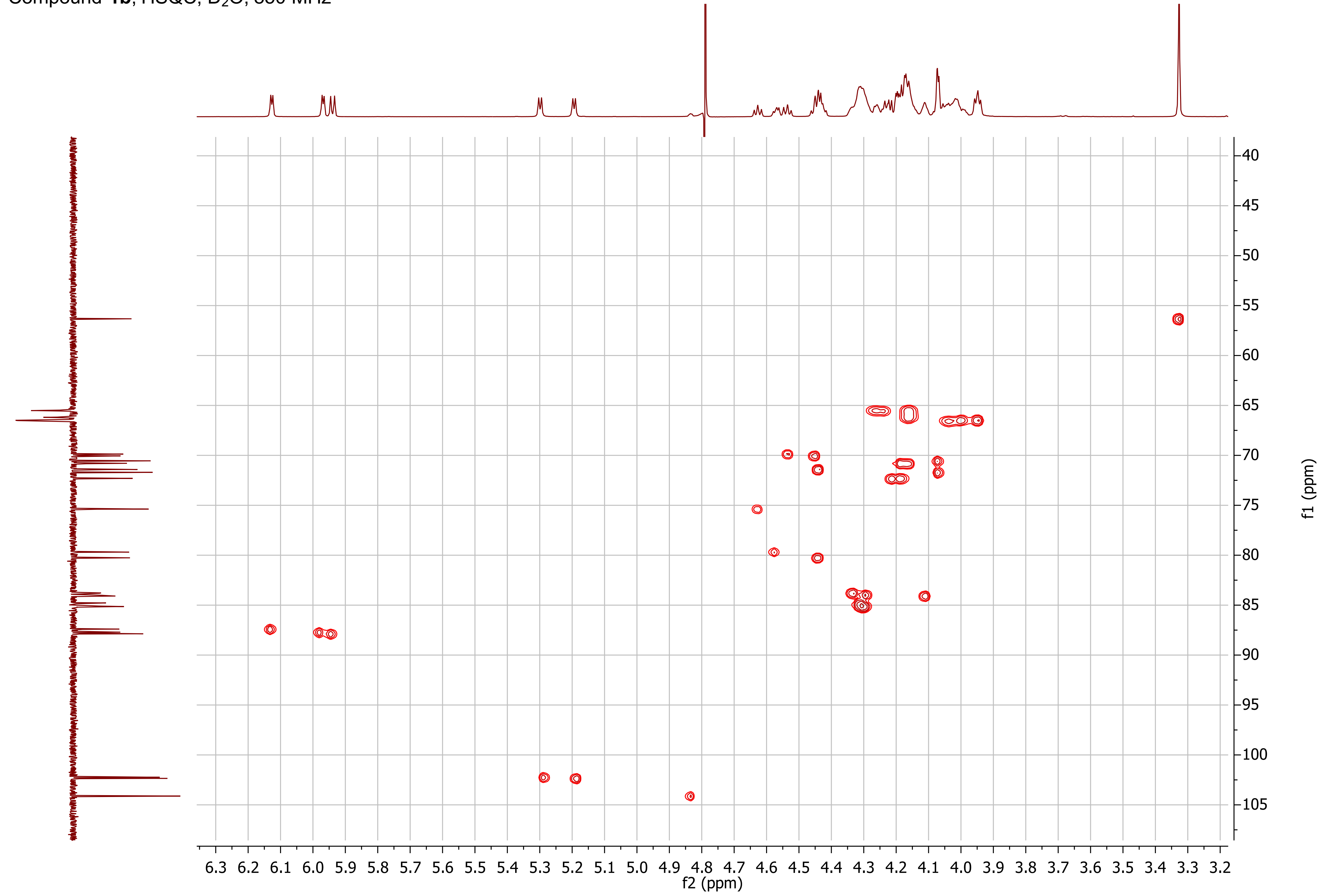

Compound **1c**, ^{1}H--NMR, D_2O, 850 MHz

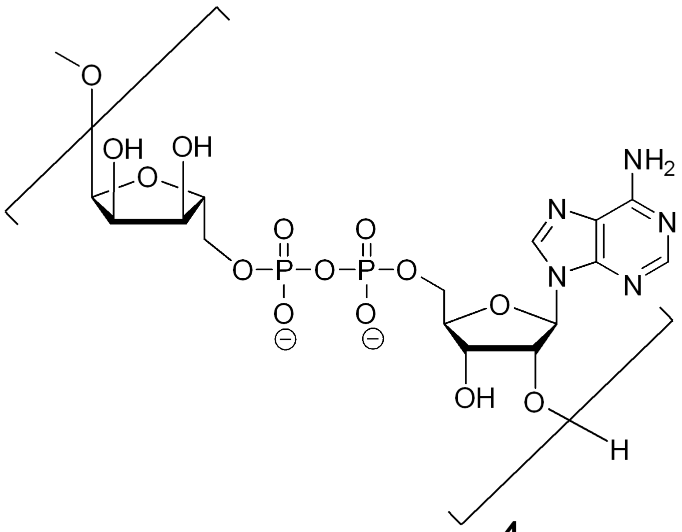

4

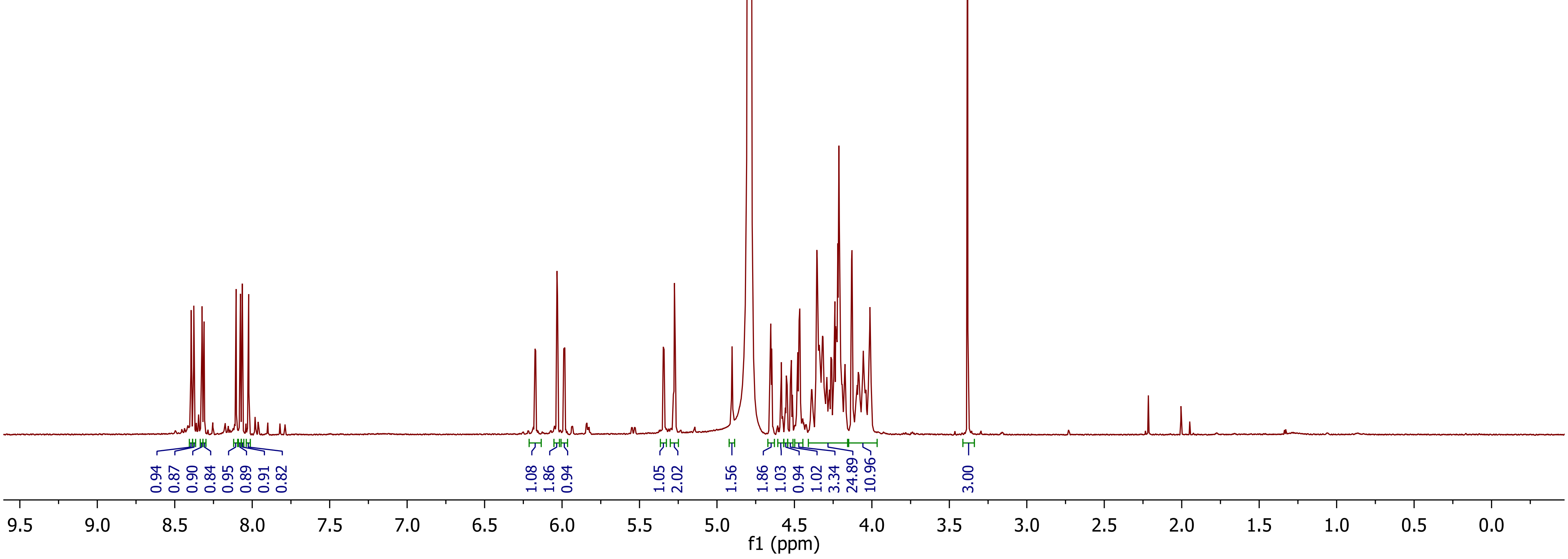

Compound **1c**, ^{1}H--NMR, D_2O, H_2O suppression, 850 MHz

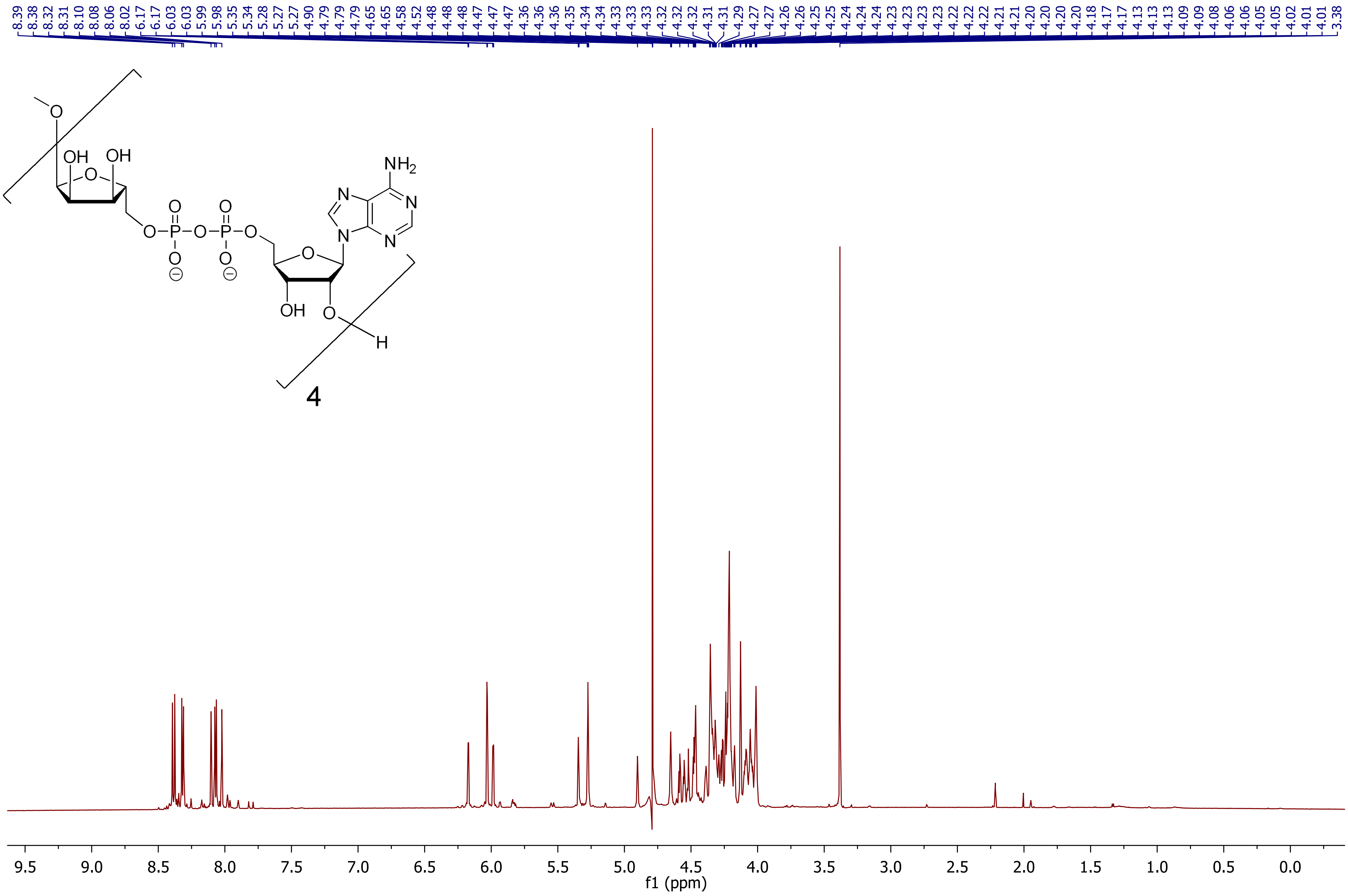

Compound **1c**, ^{31}P--NMR, D_2O, 202 MHz

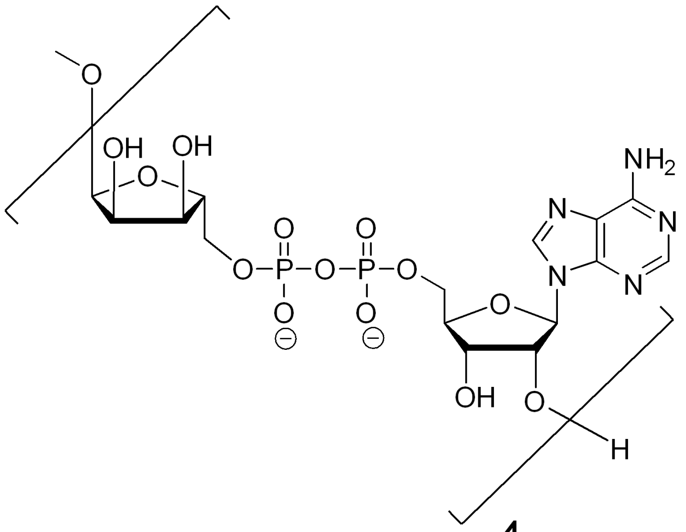

4

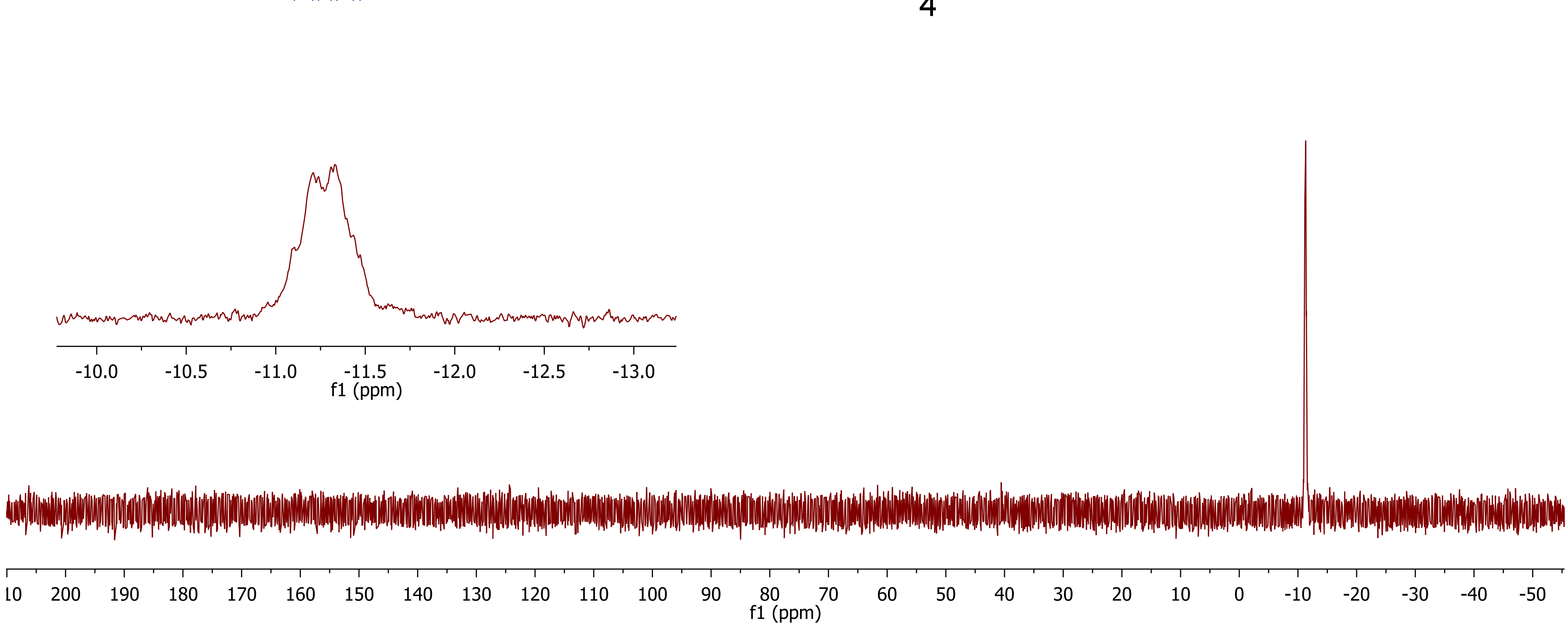

Compound **1c**, COSY, D_2O, 850 MHz

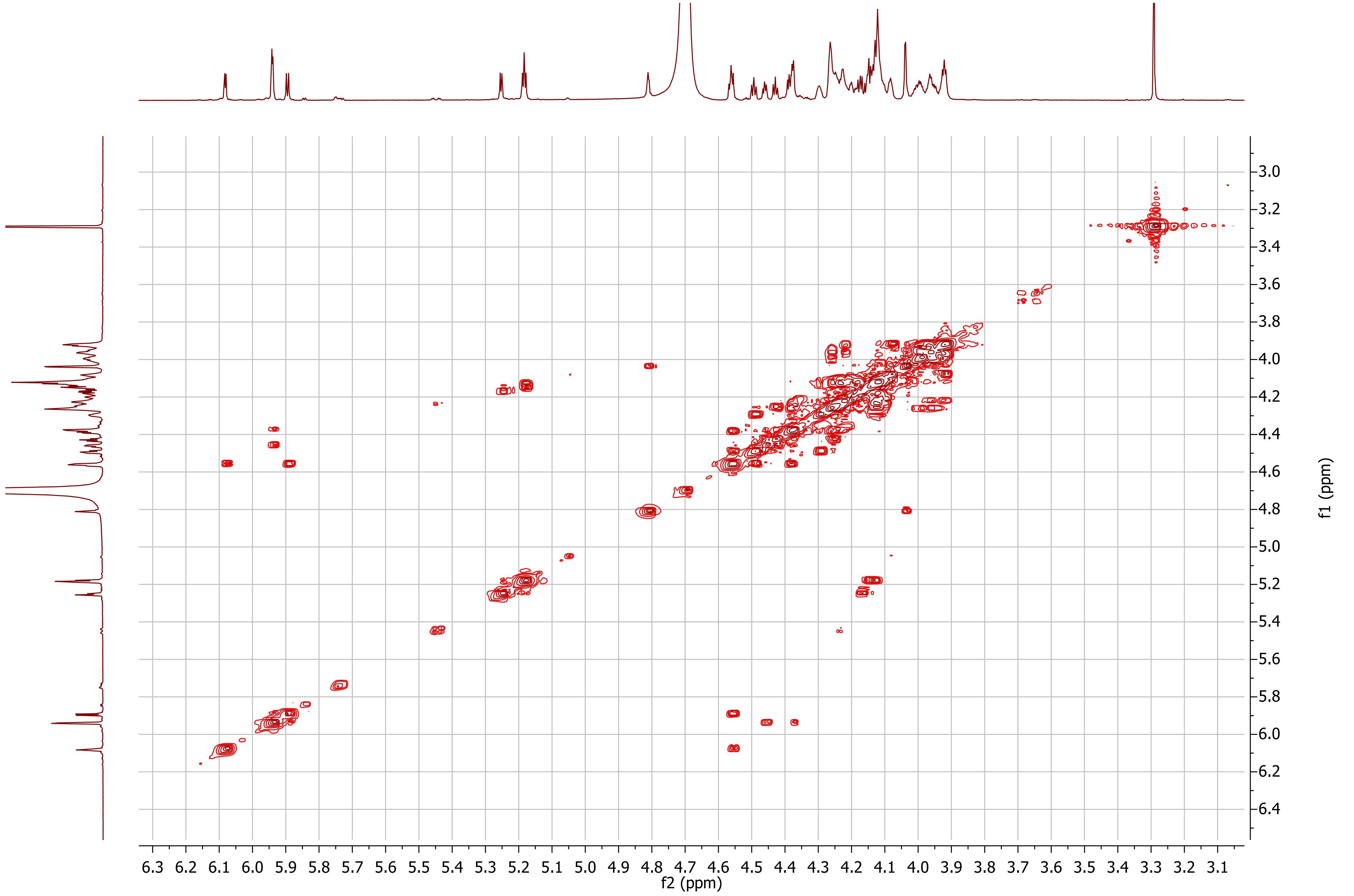

Compound **1d**, ^{1}H--NMR, D_2O, 850 MHz

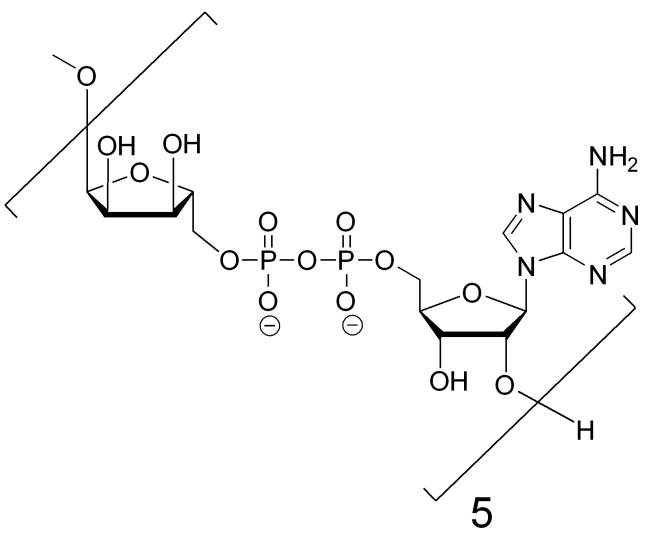

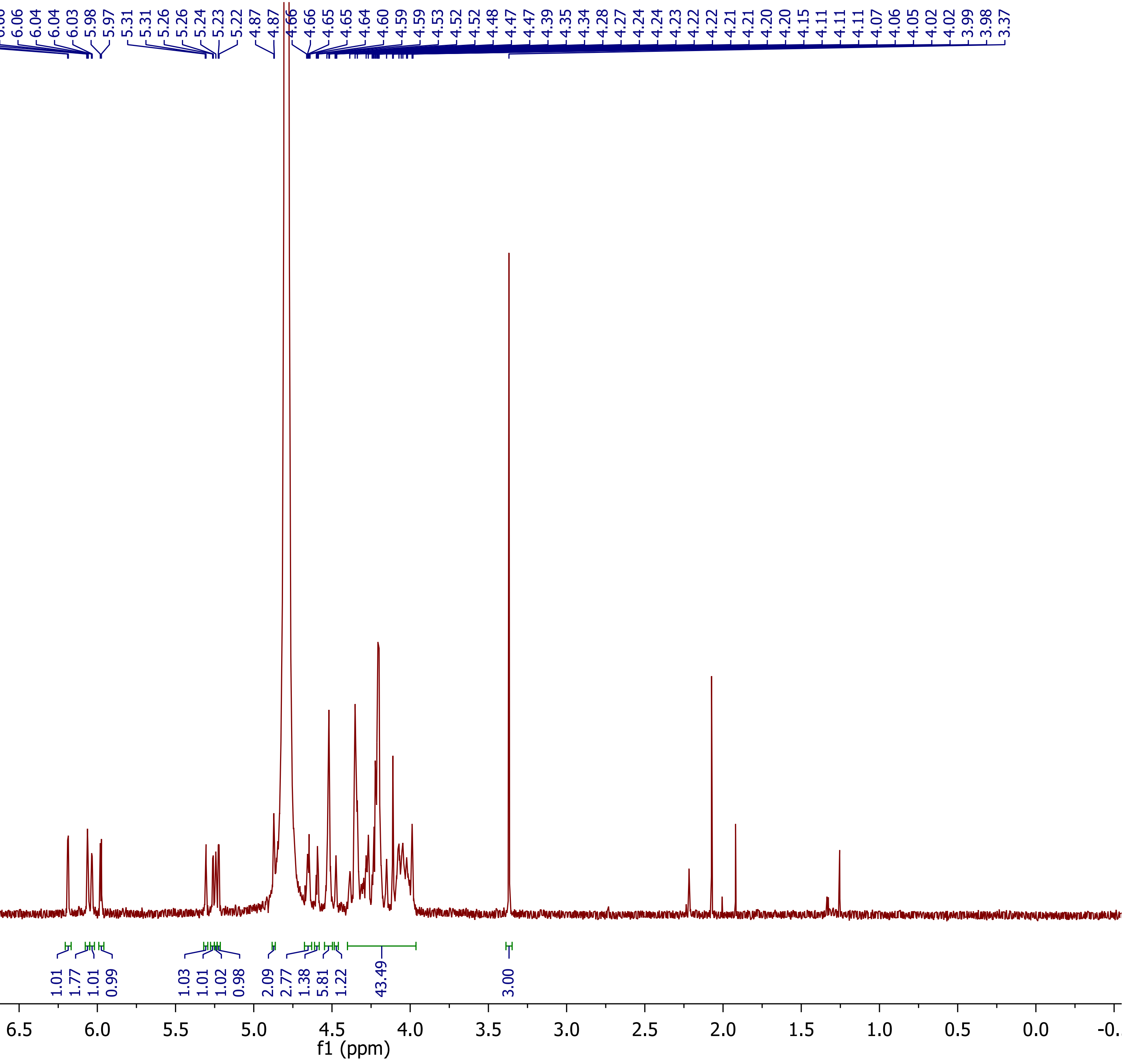

Compound **1d**, ^{1}H--NMR, D_2O, H_2O suppression, 850 MHz

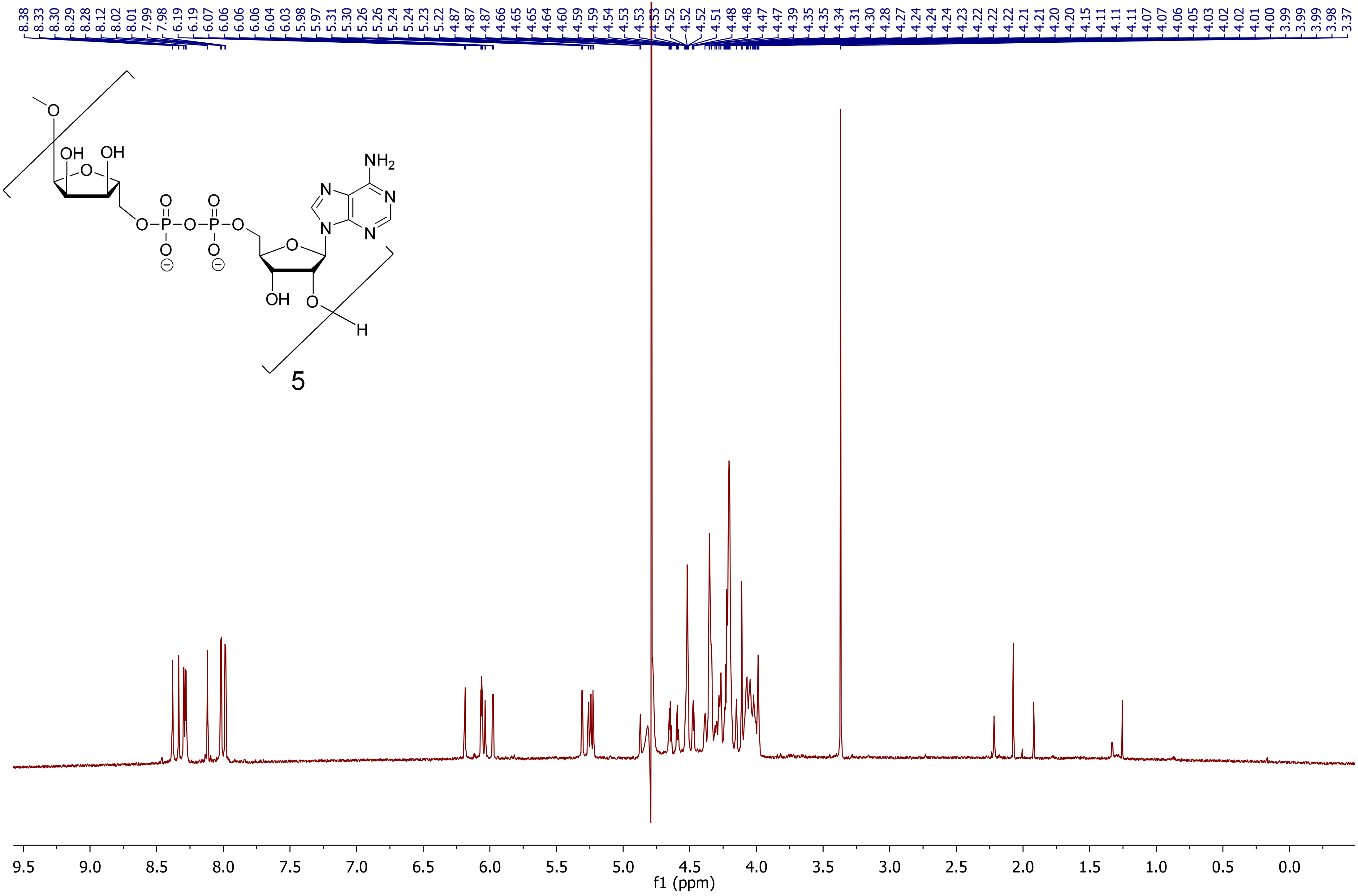

5

Compound **1d**, ^{31}P--NMR, D_2O, 202 MHz

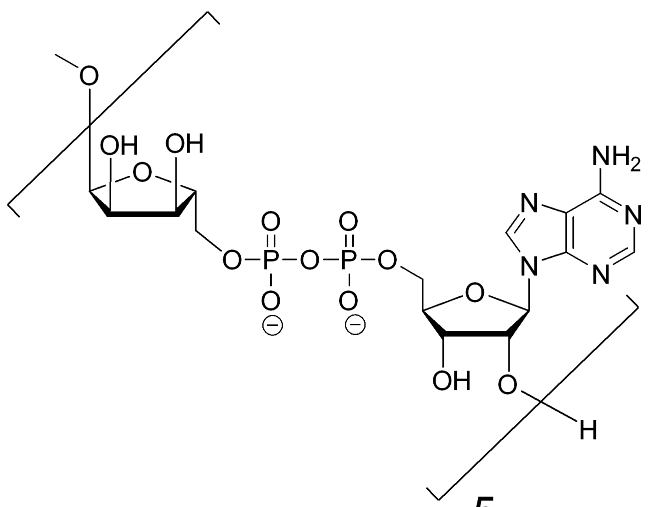

5

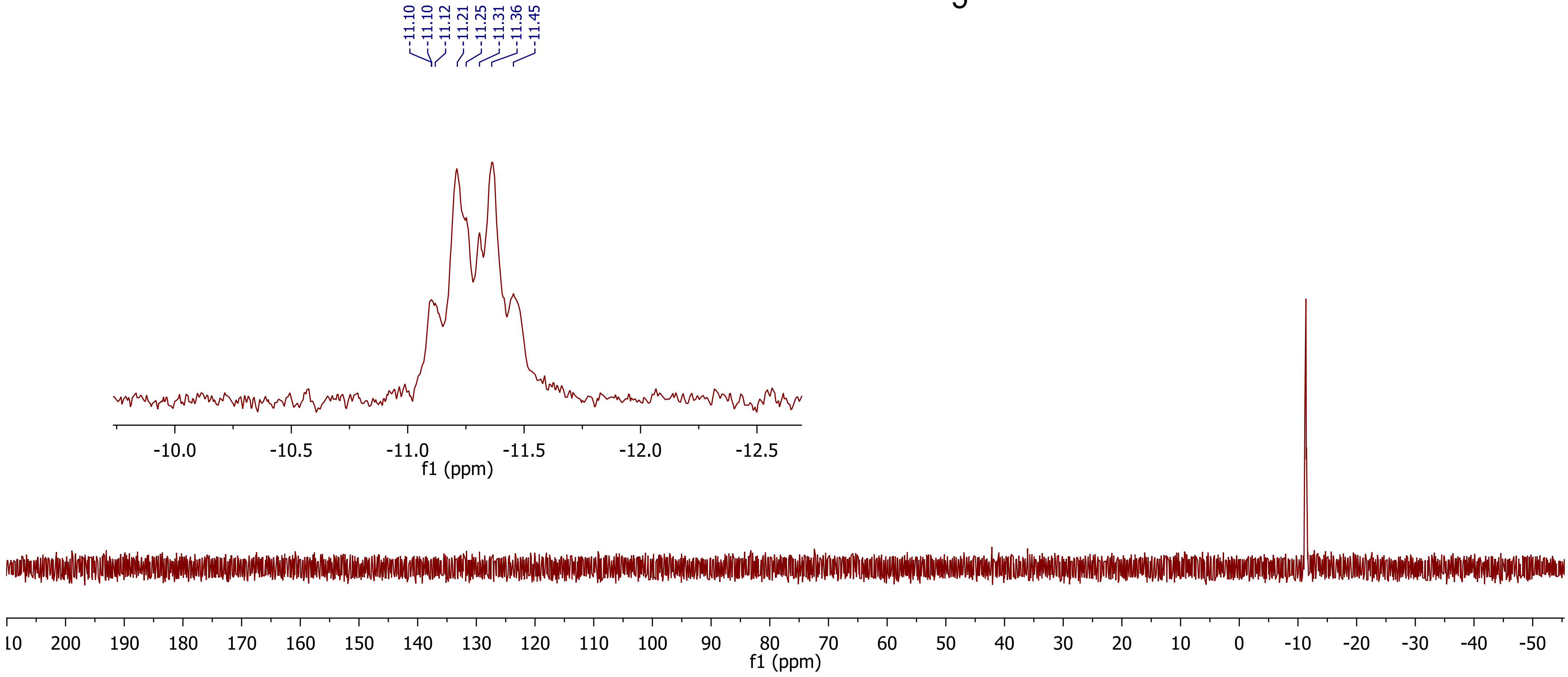

Compound **1d**, COSY, D_2O, 850 MHz

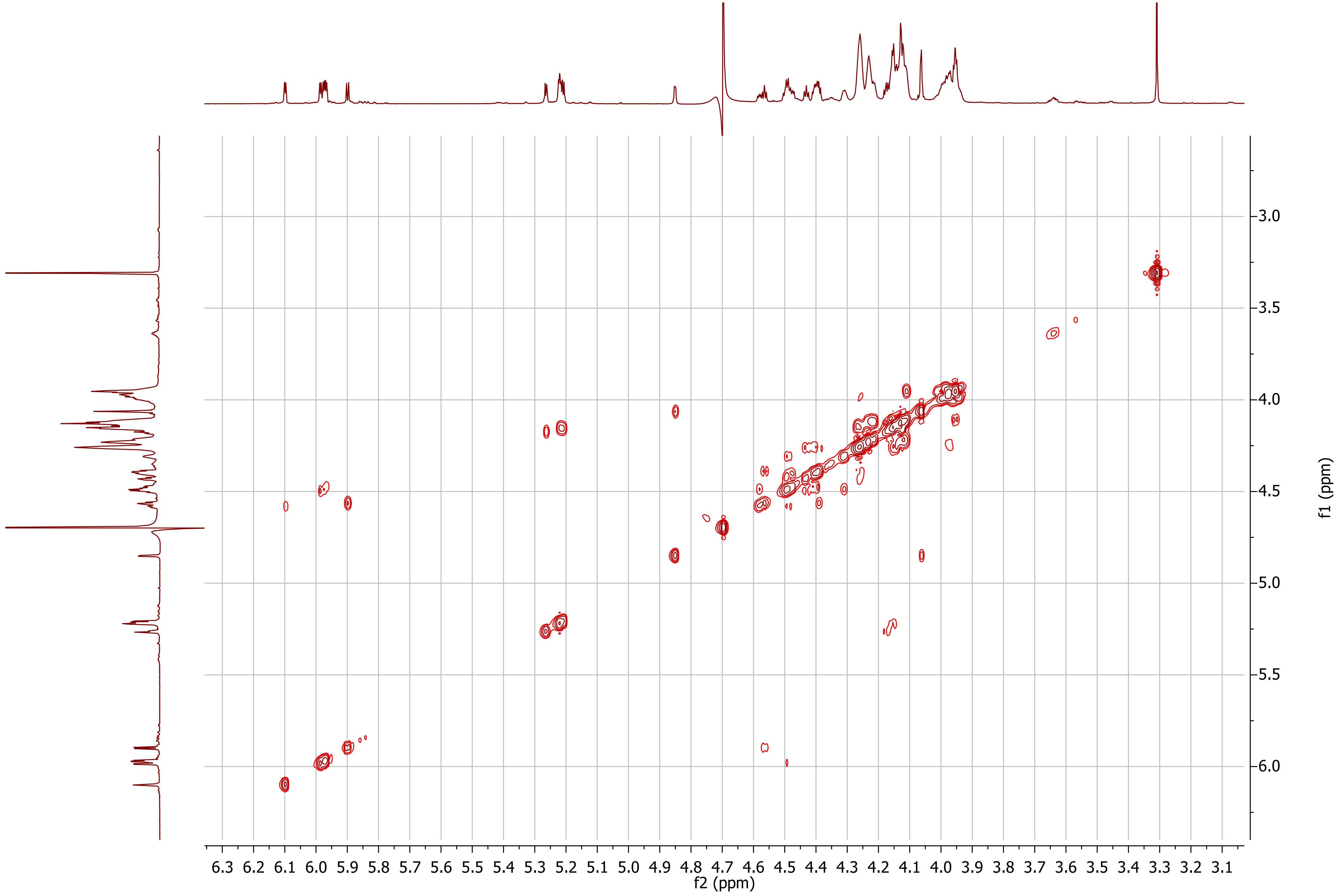

Supplement: SC-012-D1SC02340C-s001 [file SC-012-D1SC02340C-s001.pdf]
