# Supplementary material for: Chemical synthesis of linear ADP-ribose oligomers up to pentamer and their binding to the oncogenic helicase ALC1
Source: Chem Sci. 2021 Aug 18;12(37):12468–75. doi: 10.1039/d1sc02340c (PMC8480336; doi:10.1039/d1sc02340c)

Electronic Supplementary Material (ESI) for Chemical Science.
This journal is © The Royal Society of Chemistry 2021

Compound **5**, ^{1}H--NMR, $CDCl_3$, 500 MHz

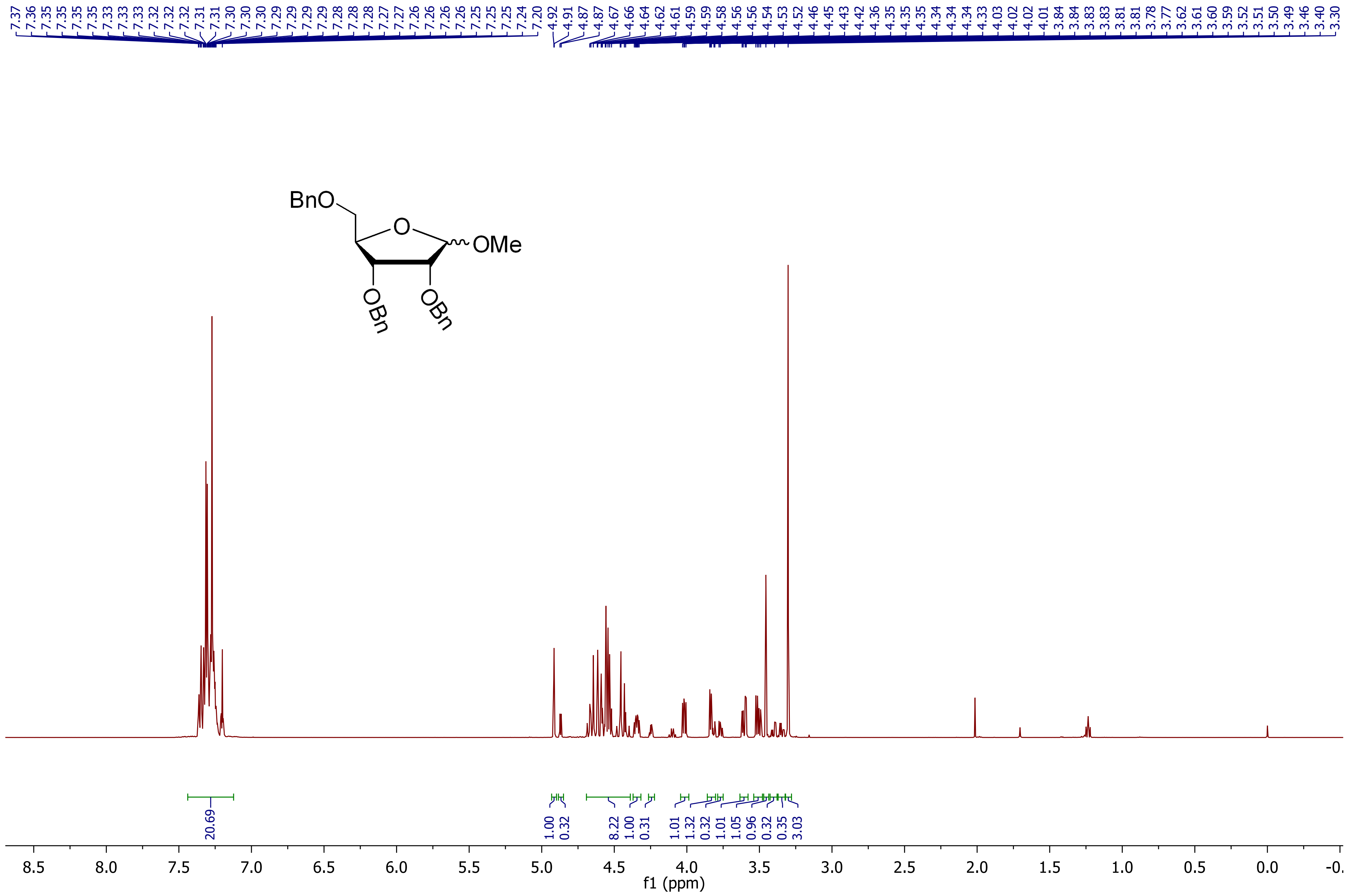

Compound **5**, ^{13}C--NMR, $CDCl_3$, 126 MHz

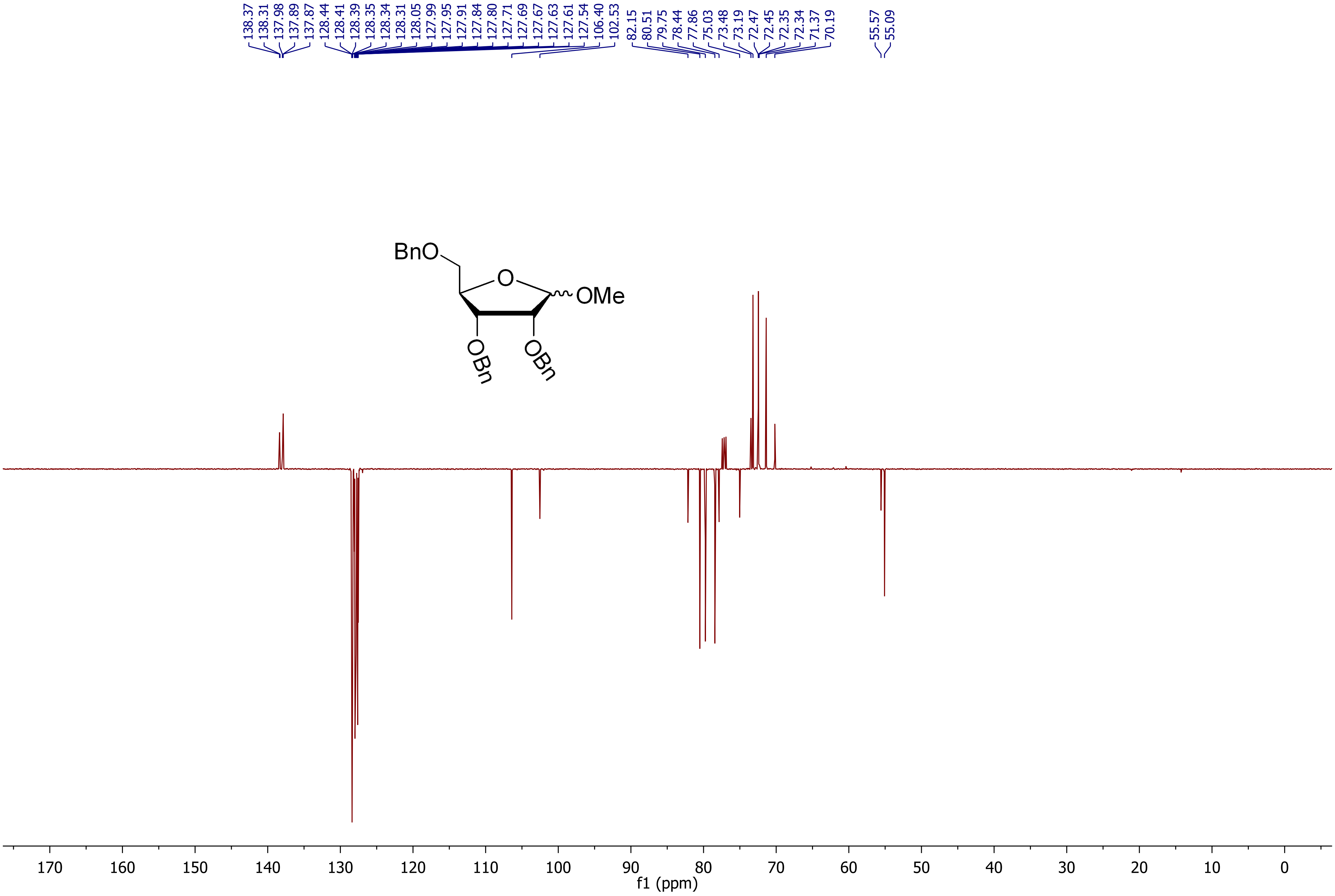

Compound **6**, ^{1}H--NMR, $CDCl_3$, 500 MHz

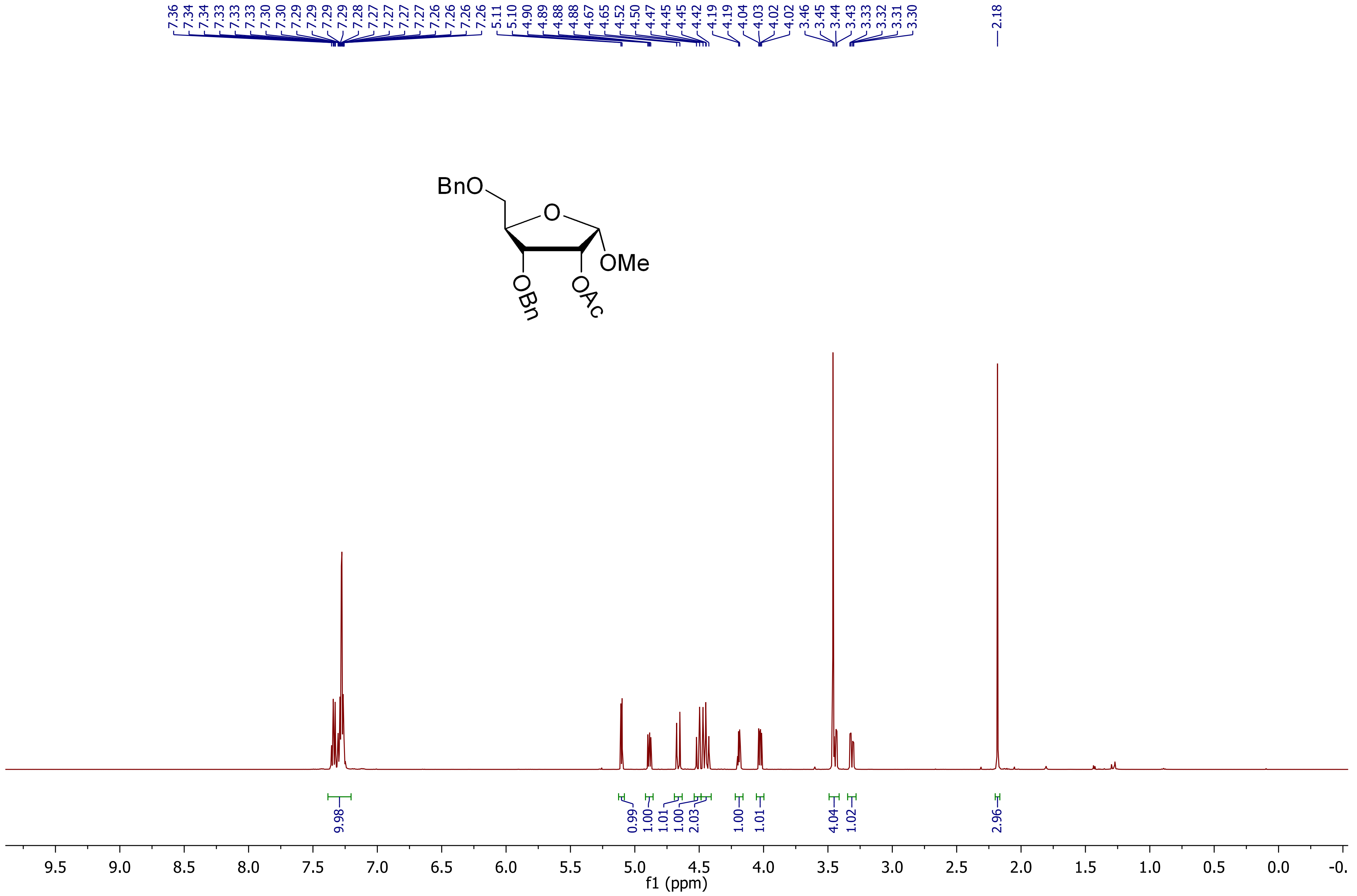

Compound **6,** ^{13}C--NMR, $CDCl_3$, 126 MHz

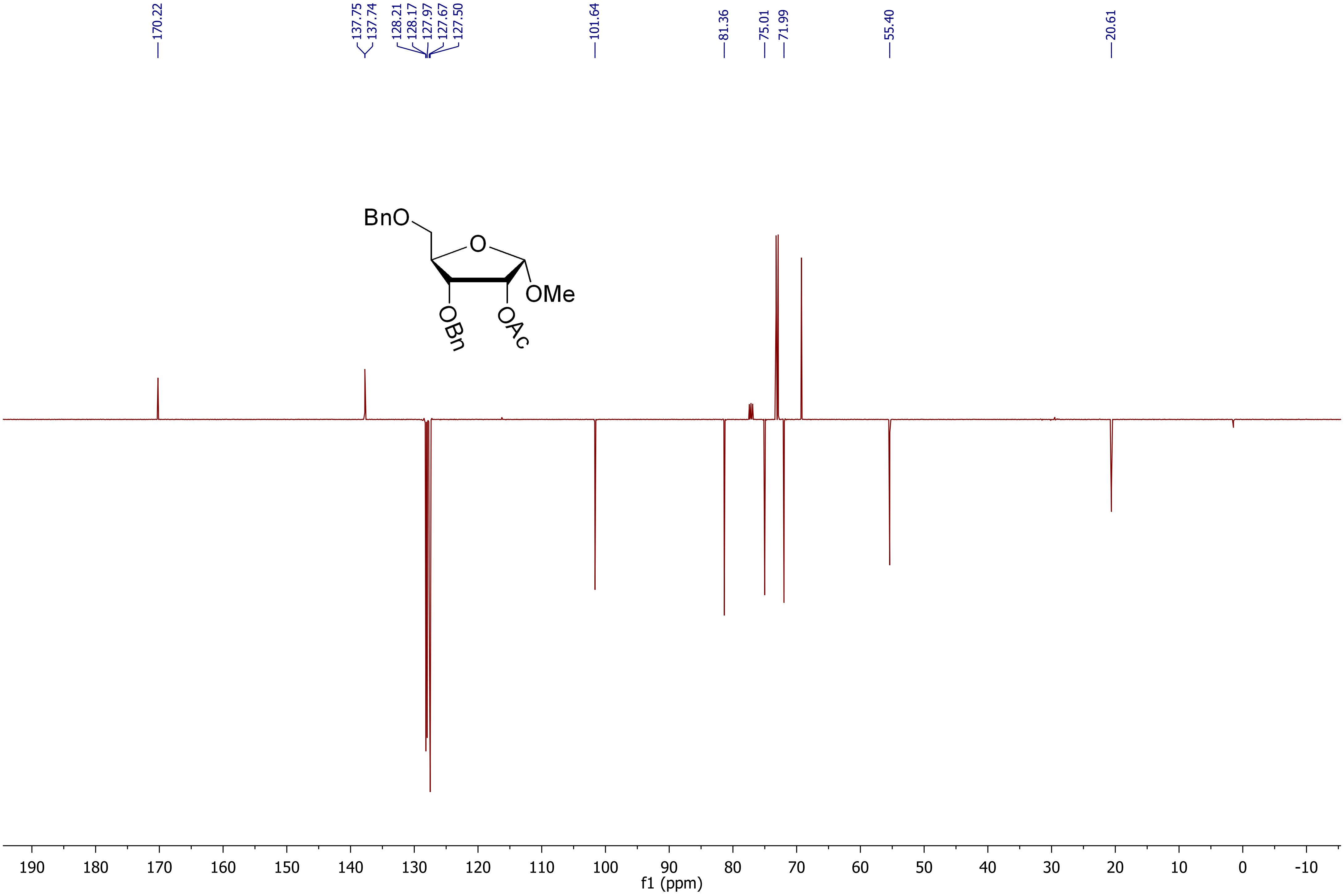

Compound **7**, ^{1}H--NMR, $CDCl_3$, 500 MHz

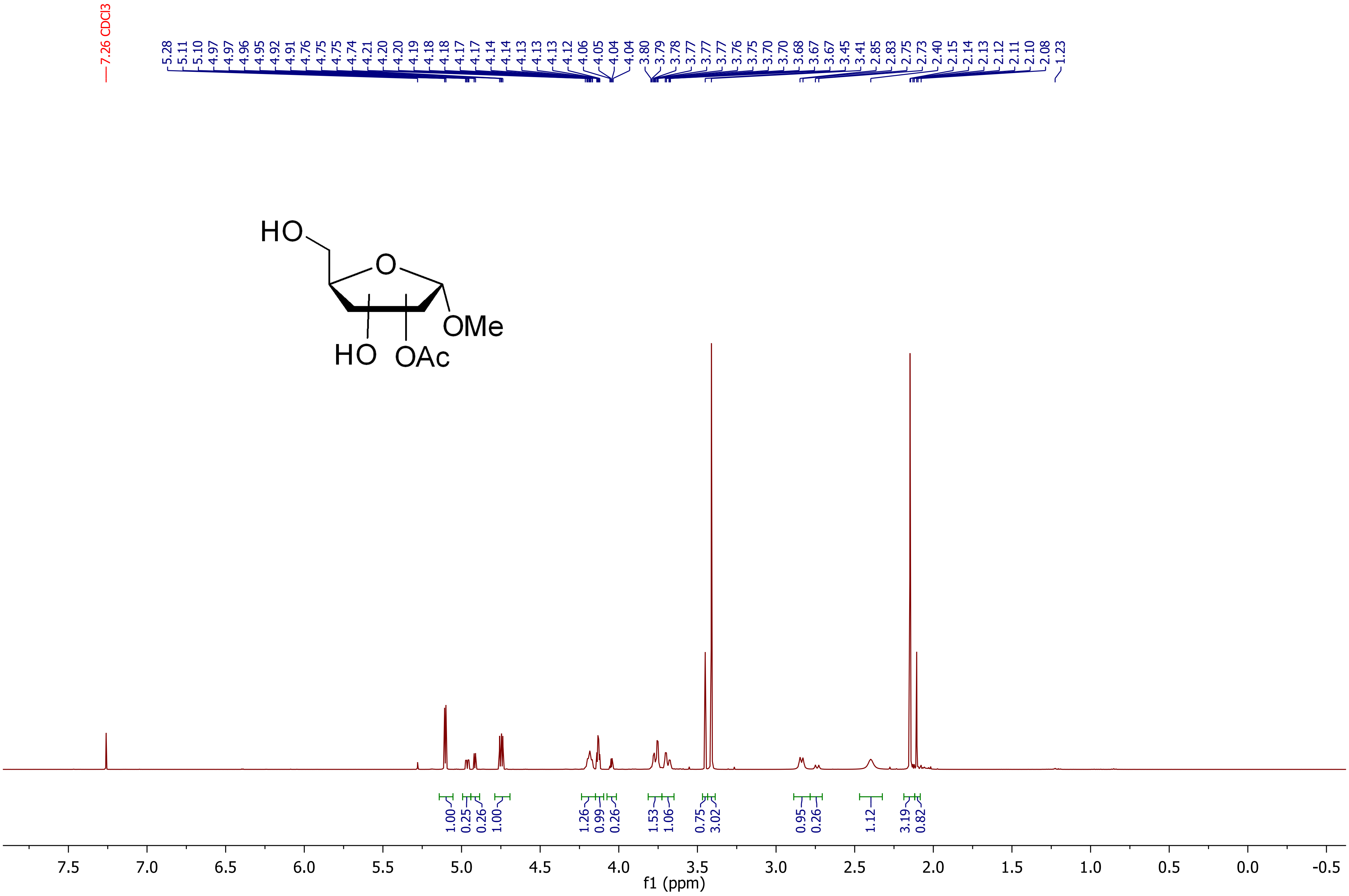

Compound **7**, ^{13}C--NMR, CDCl$_3$, 126 MHz

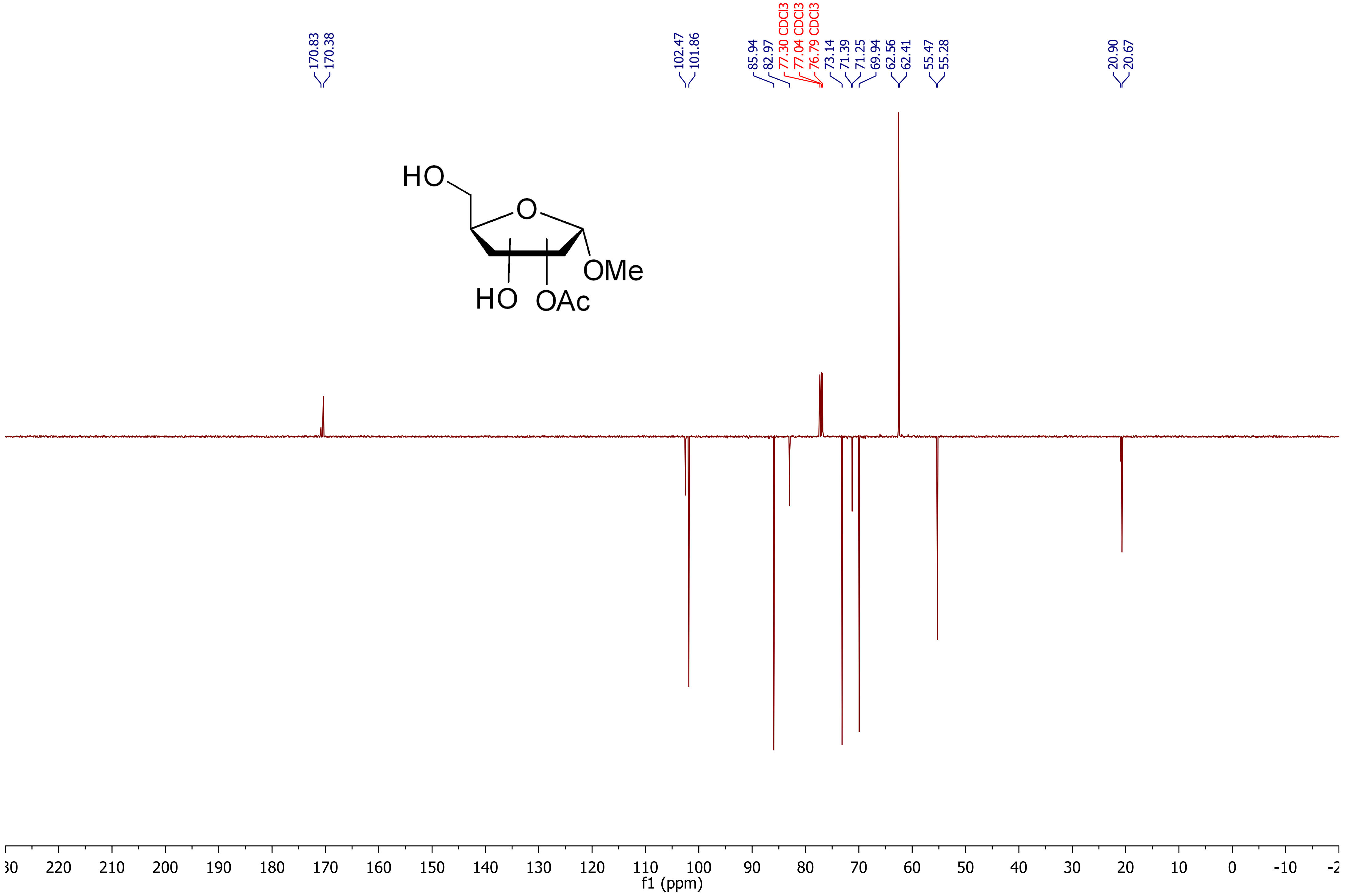

Compound **8**, ^{1}H--NMR, $CDCl_3$, 400 MHz

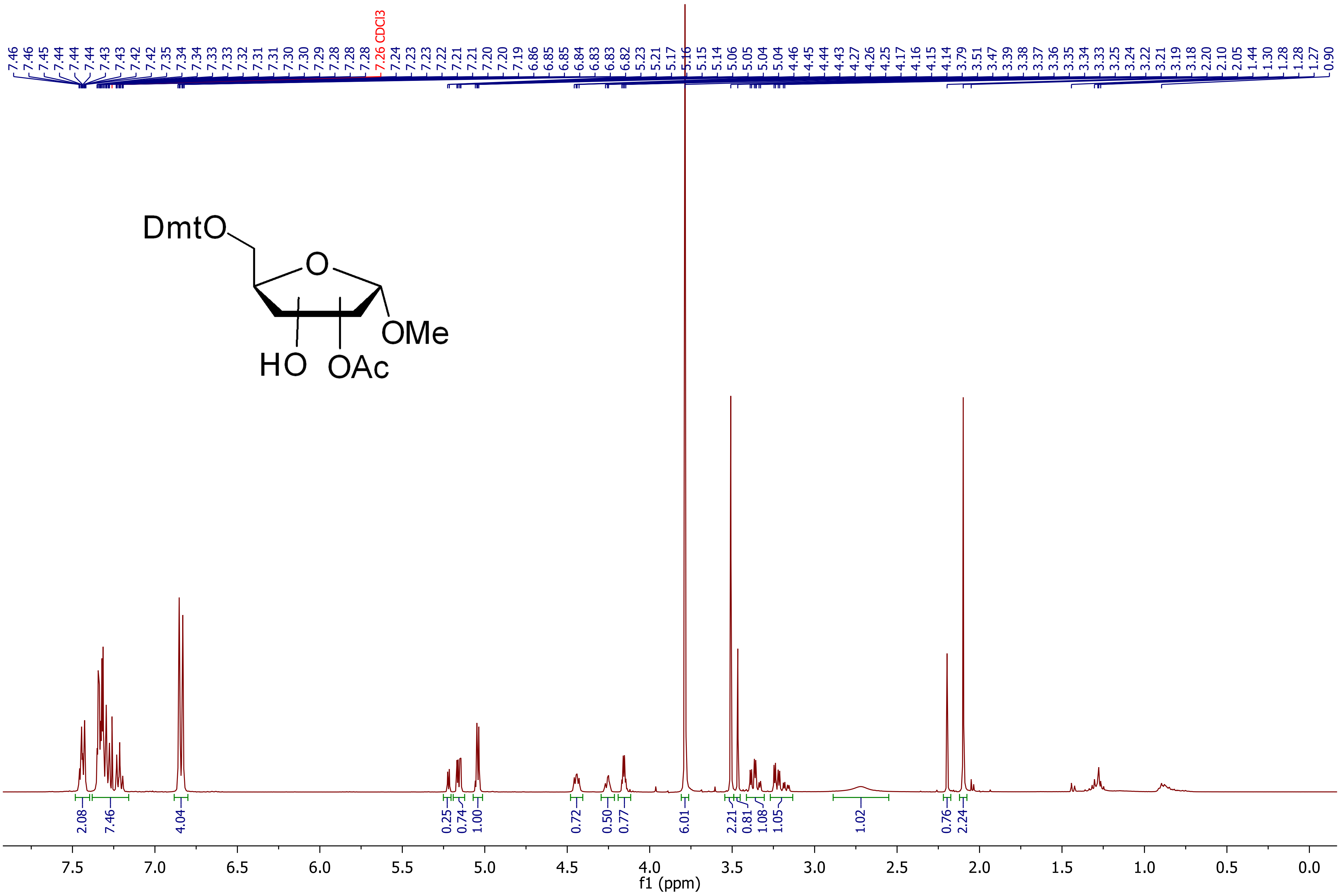

Compound **8**, ^{13}C--NMR, $CDCl_3$, 101 MHz

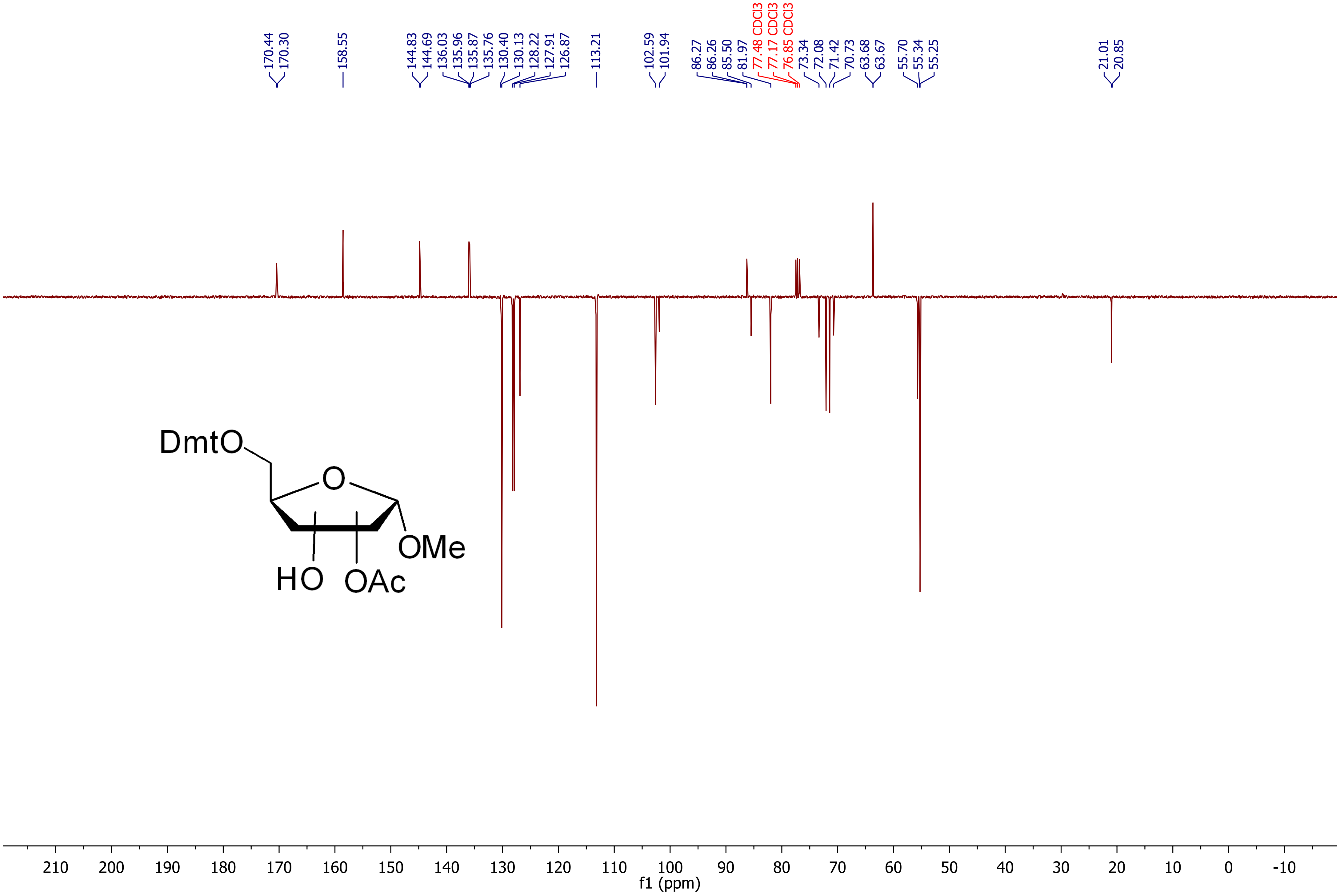

Compound **10**, ^{1}H--NMR, $CDCl_3$, 500 MHz

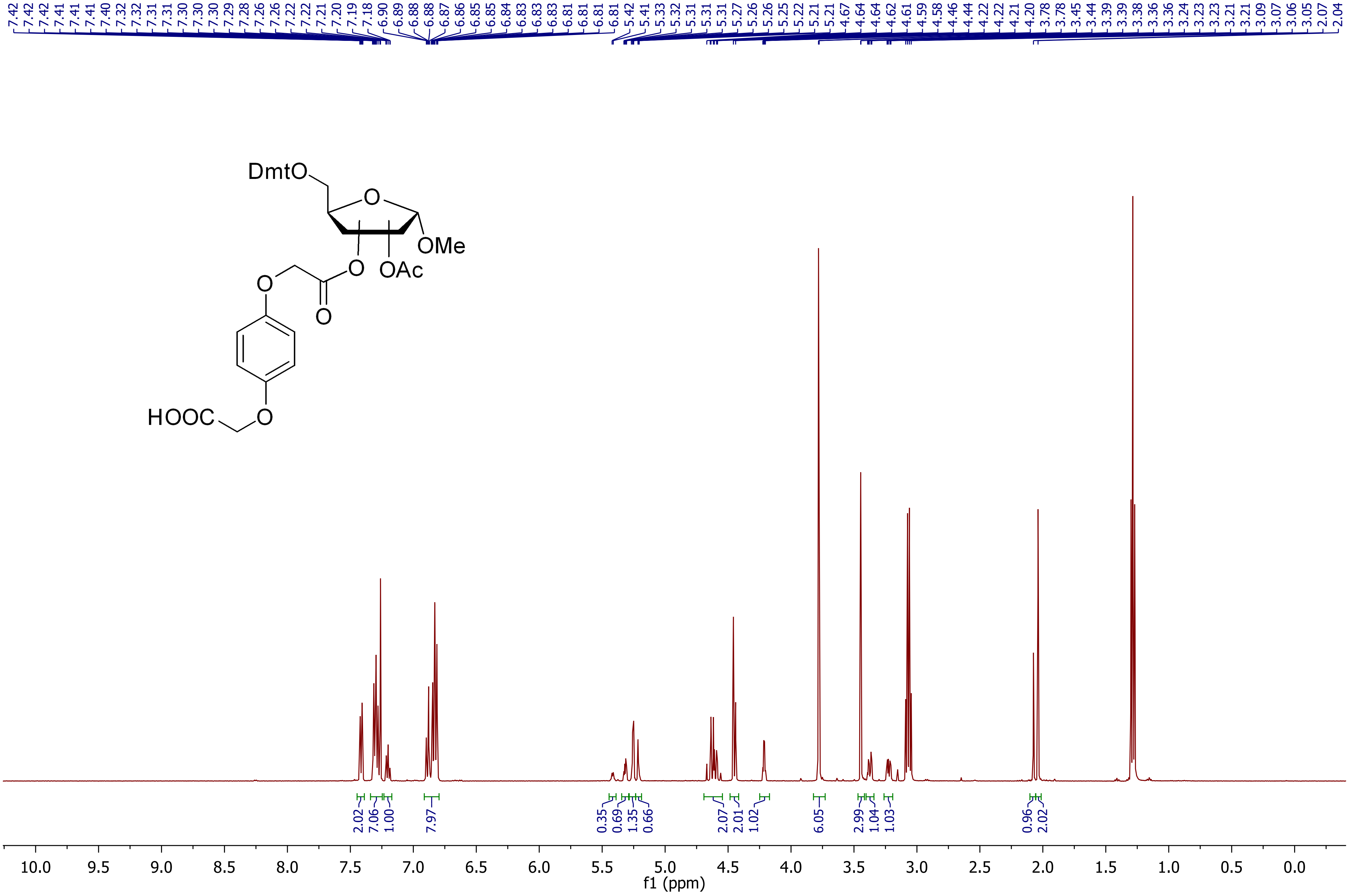

Compound **10**, ^{13}C--NMR, $CDCl_3$, 126 MHz

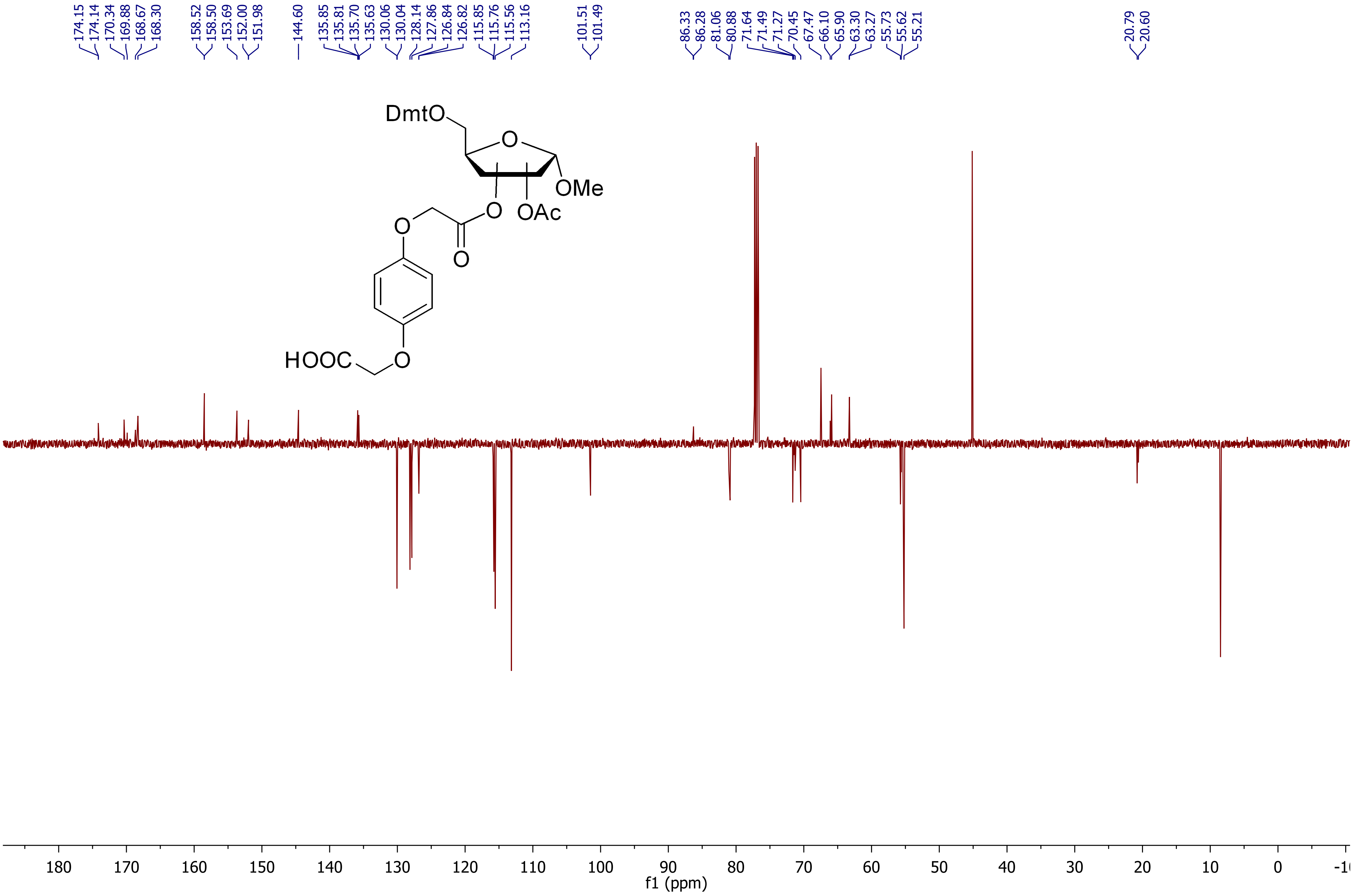

Compound **2m** (deprotected), ^{31}P--NMR, D_2O, 162 MHz

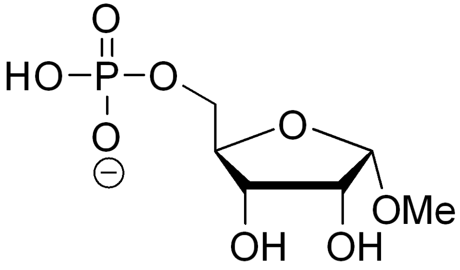

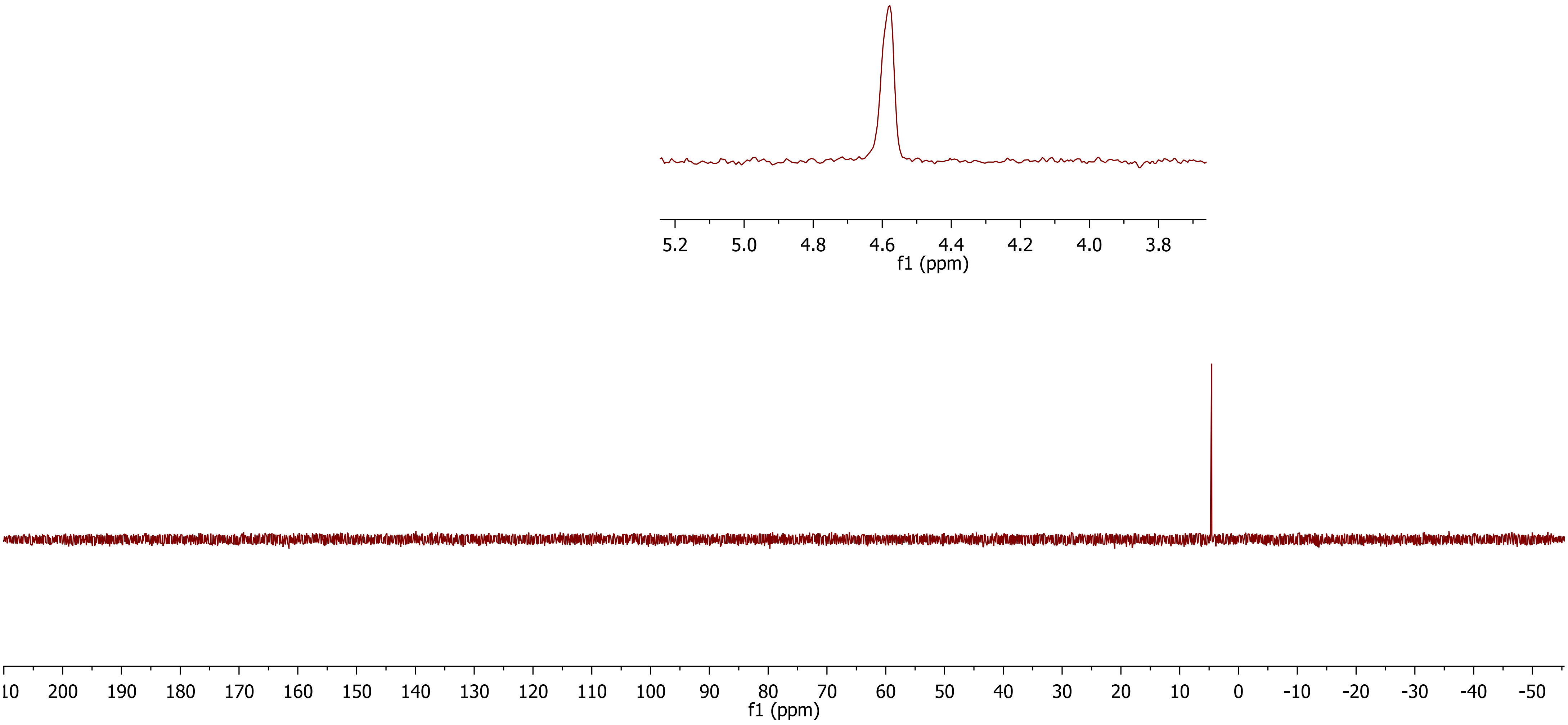

Compound **2k** (deprotected), ^{31}P--NMR, D_2O, 162 MHz

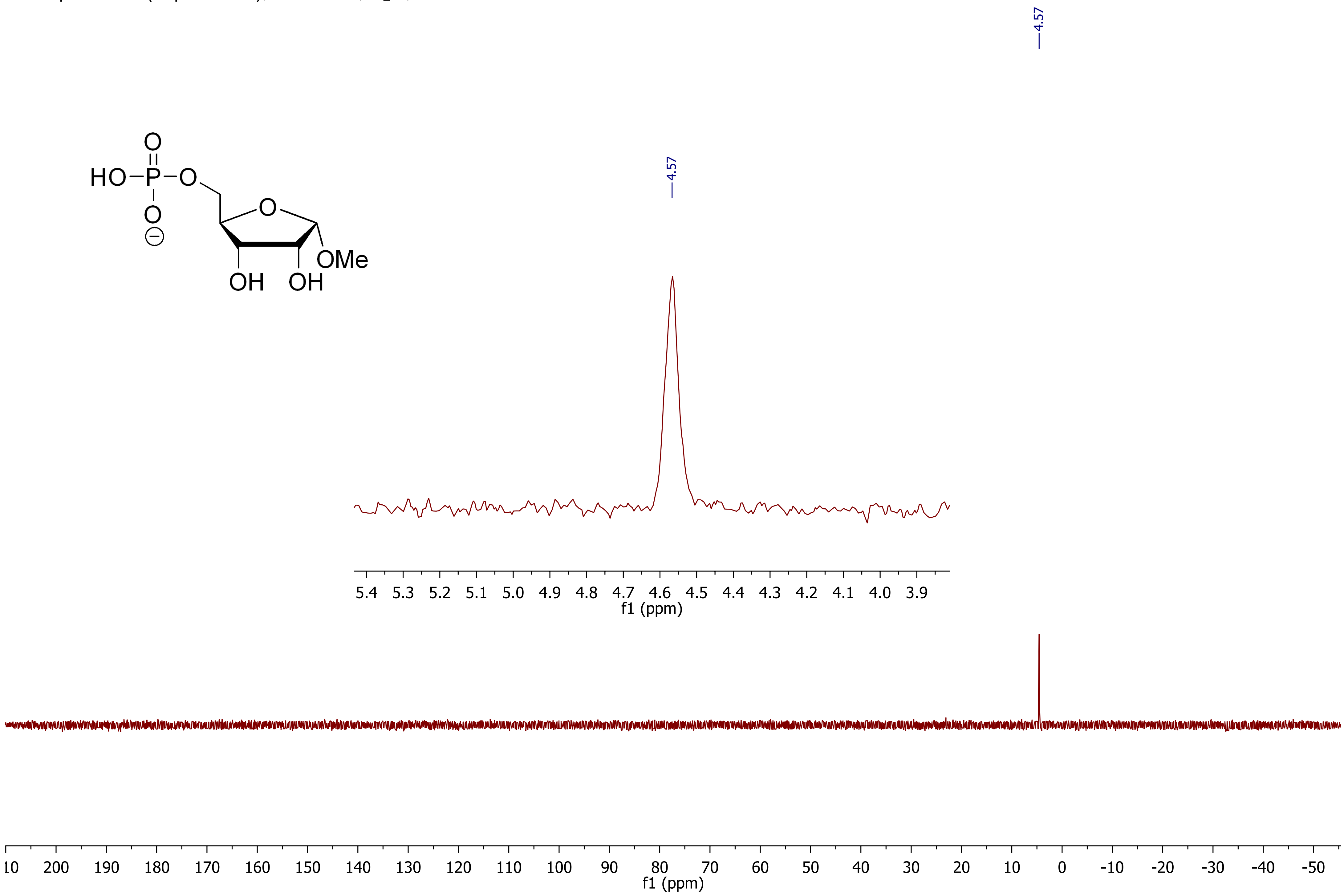

Compound **15**, ^{1}H--NMR, $CDCl_3$, 500 MHz

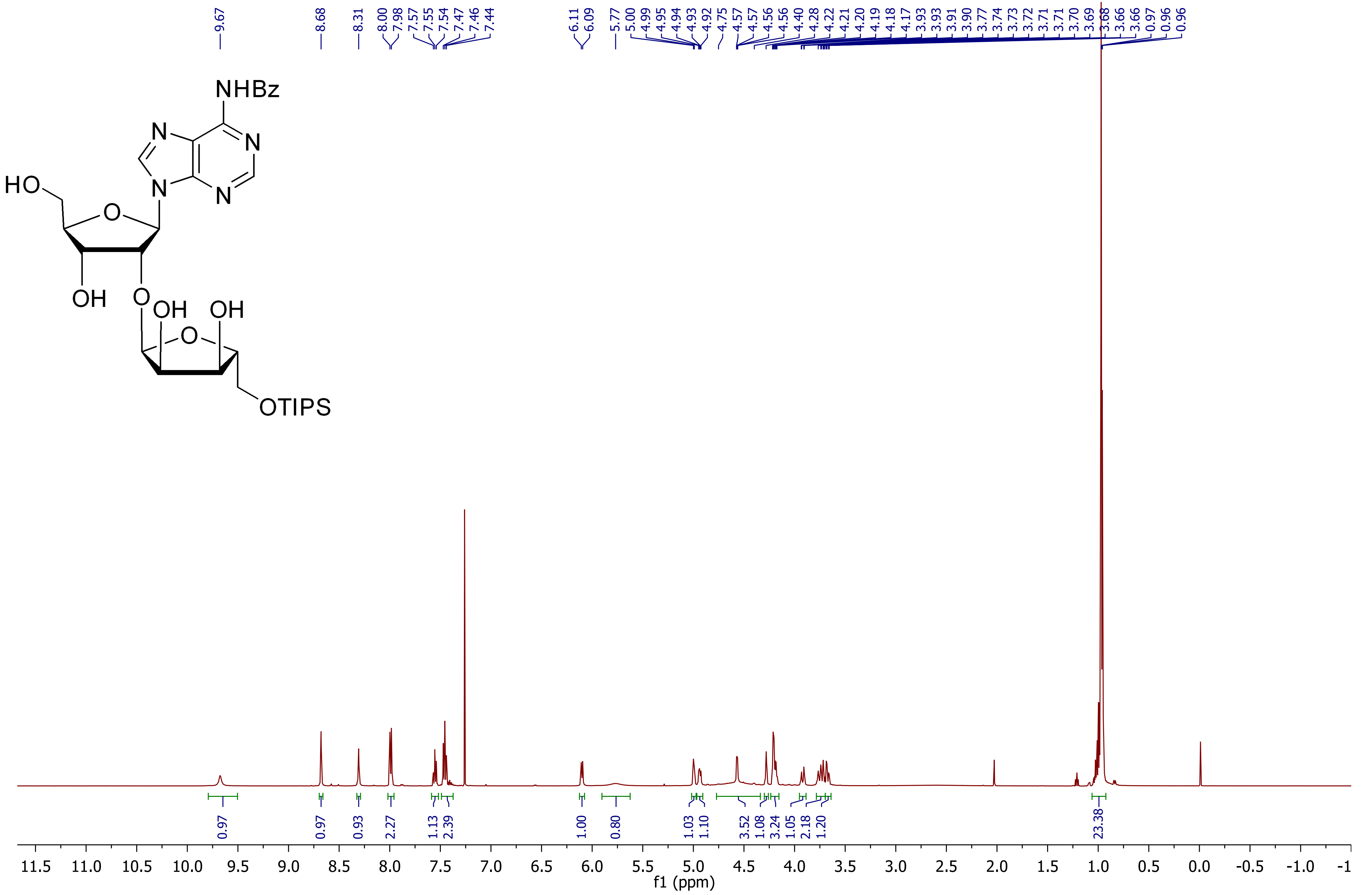

Compound **15**, ^{13}C--NMR, $CDCl_3$, 126 MHz

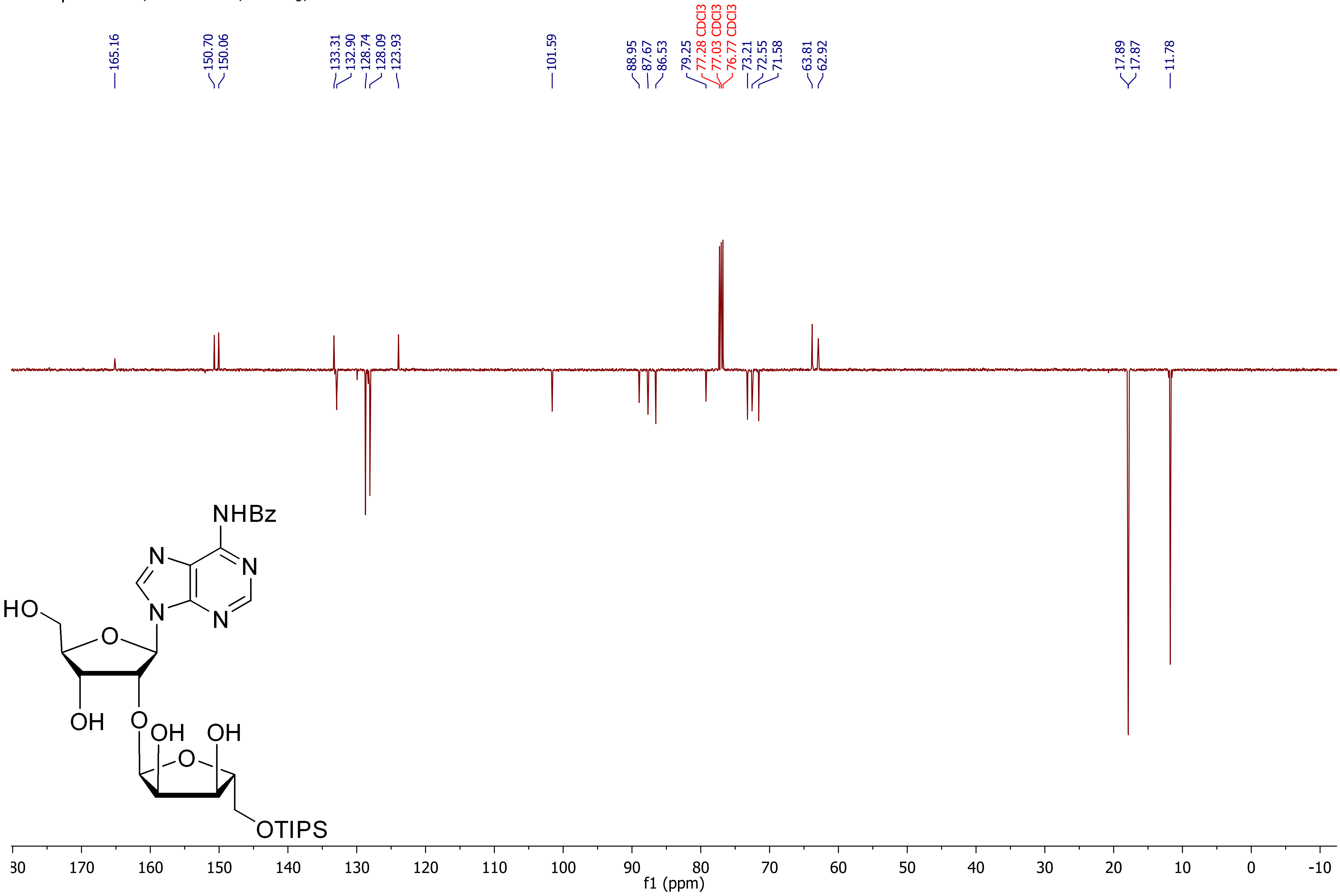

Compound **16**, ^{1}H--NMR, $CDCl_3$, 500 MHz

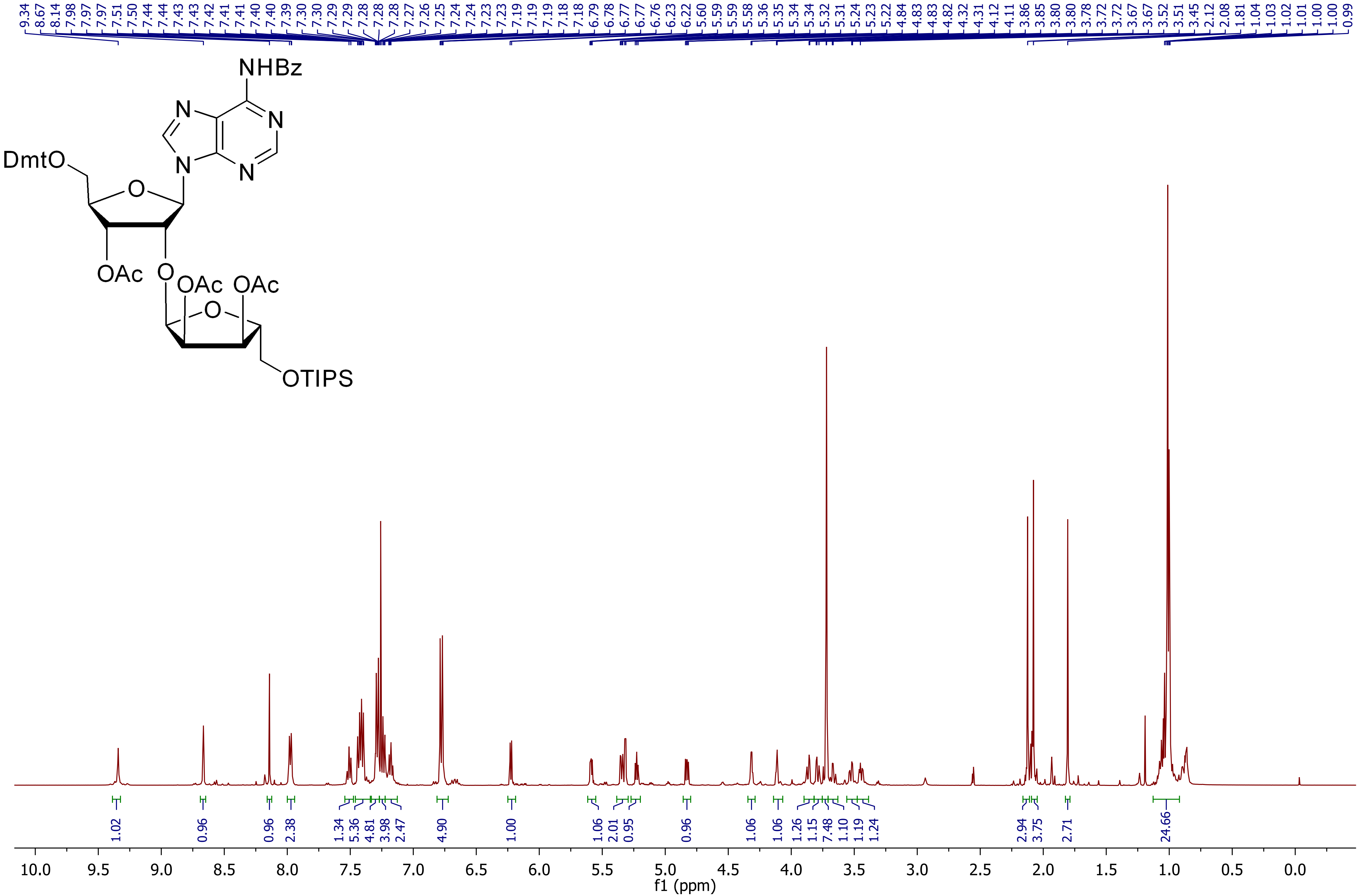

Compound **16**, ^{13}C--NMR, $CDCl_3$, 126 MHz

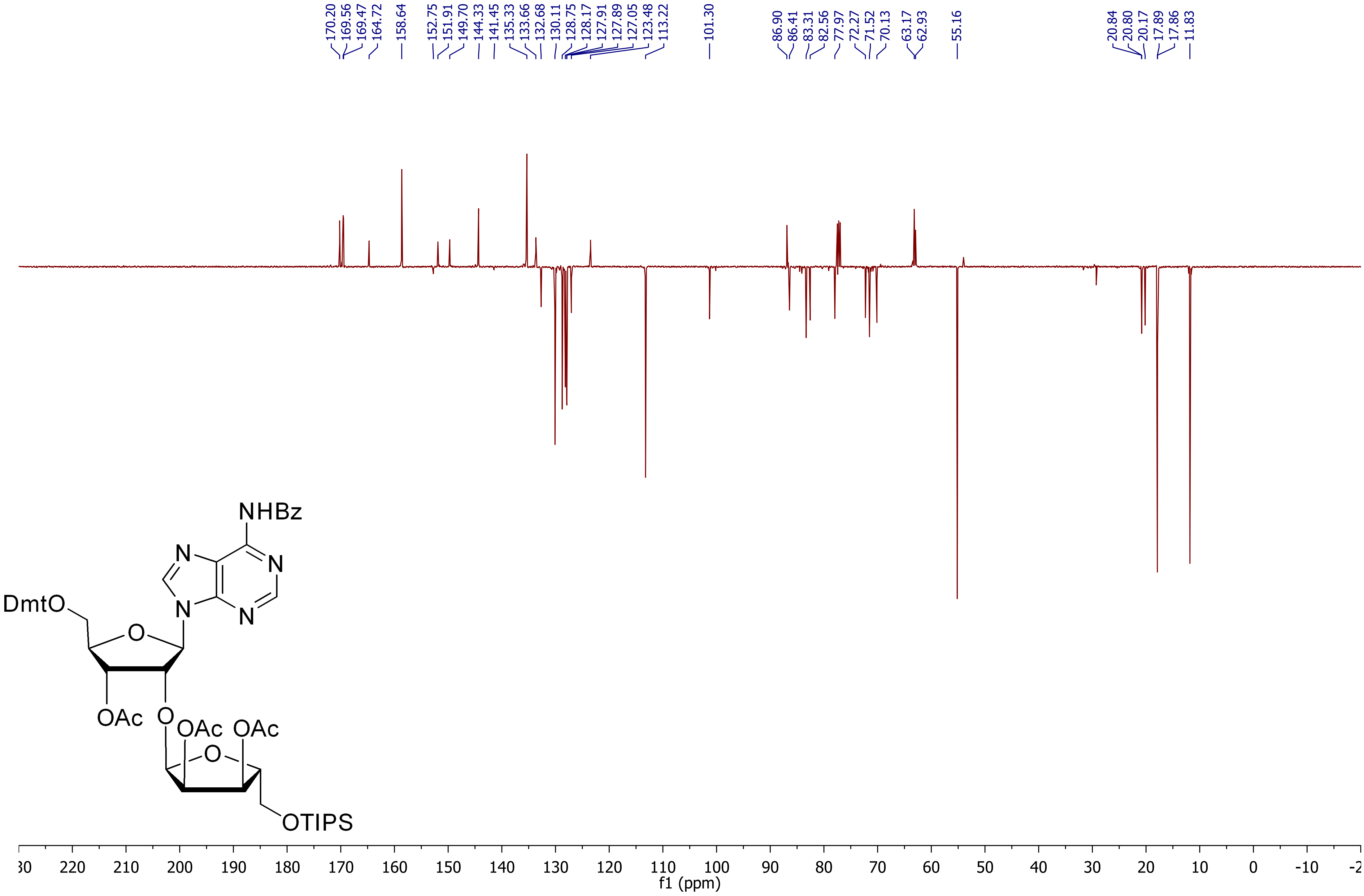

Compound **17**, ^{1}H--NMR, $CDCl_3$, 400 MHz

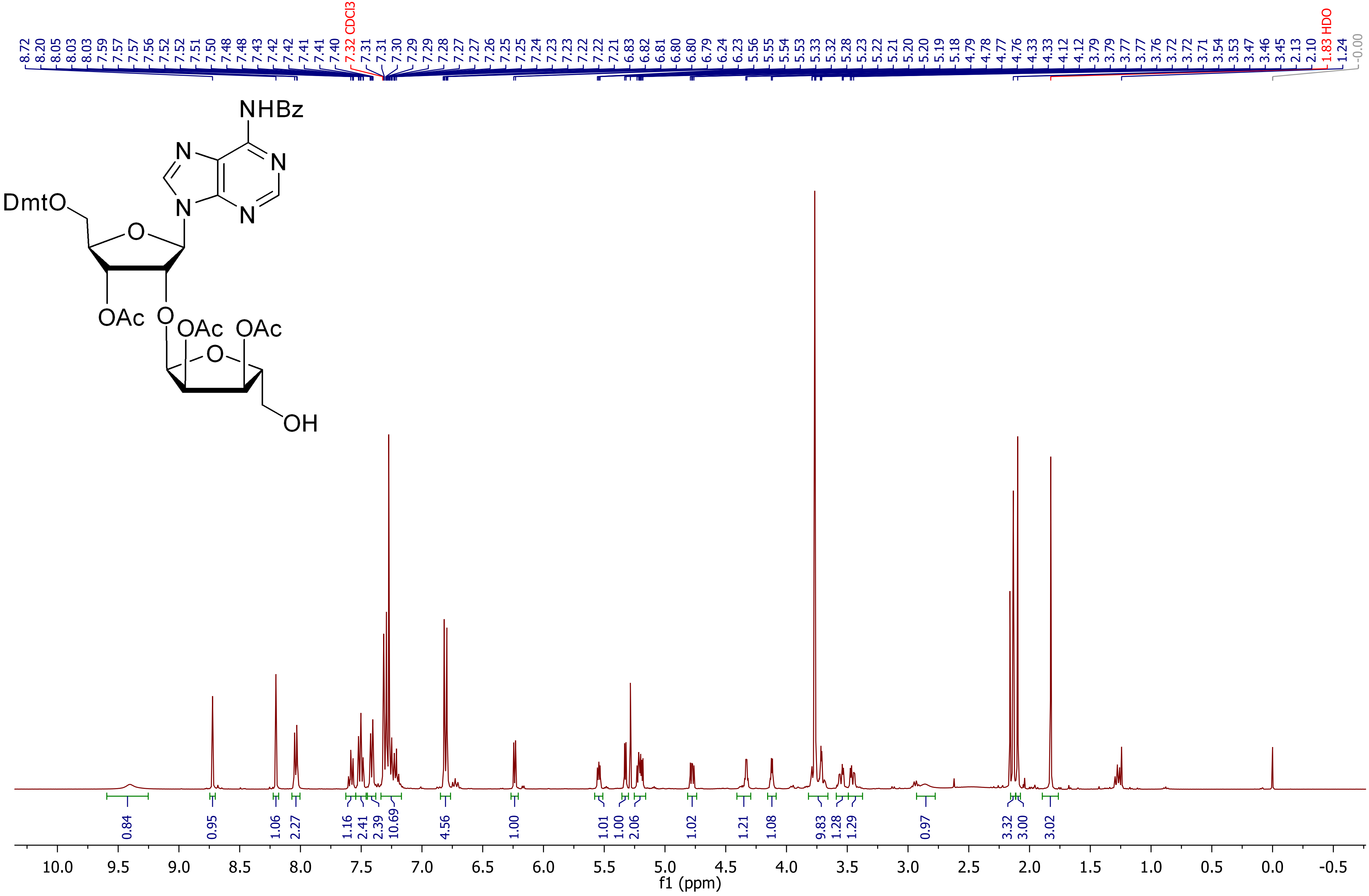

Compound **17**, ^{13}C--NMR, $CDCl_3$, 101 MHz

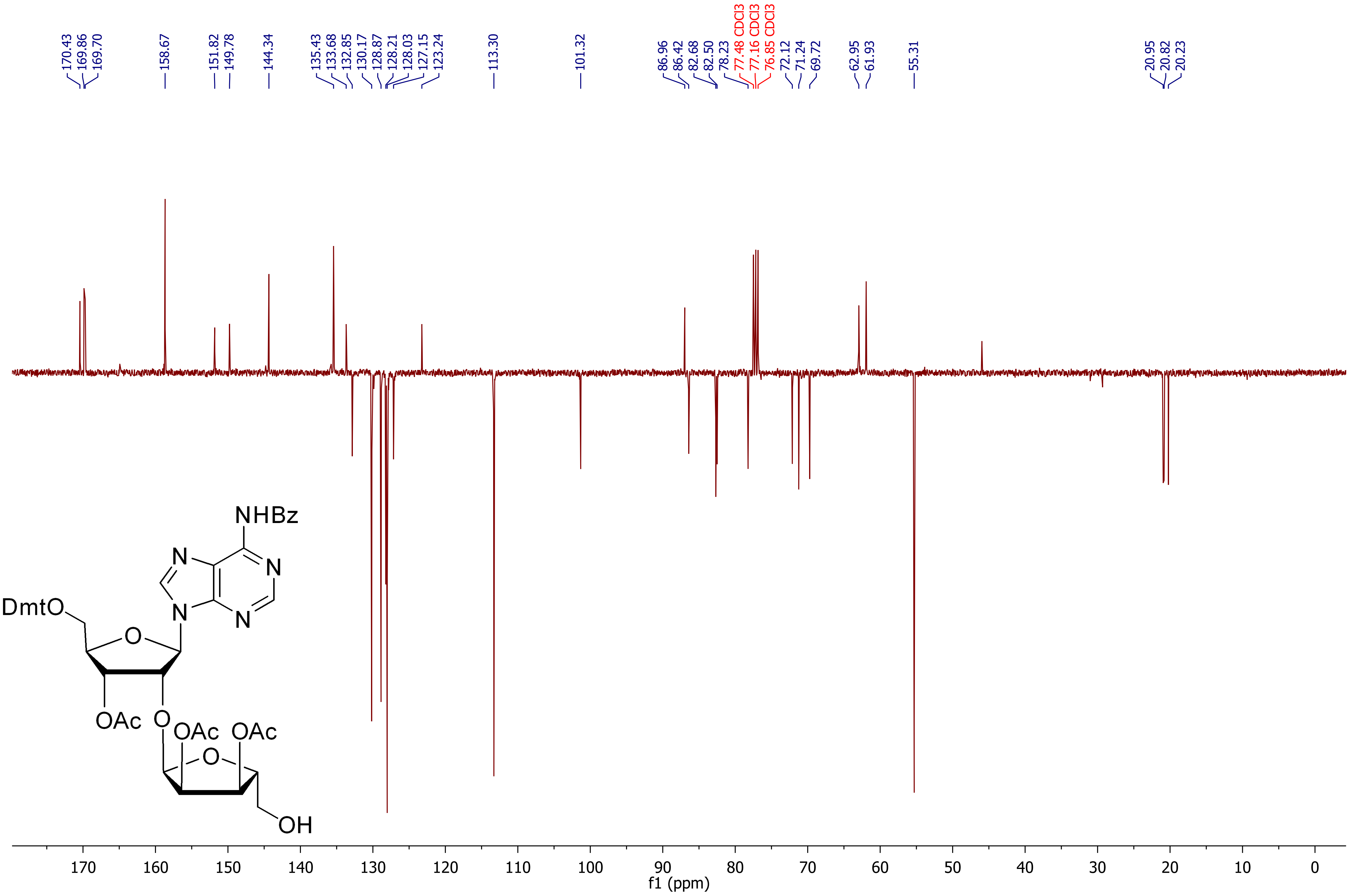

Compound **18**, ^{1}H--NMR, $CDCl_3$, 500 MHz

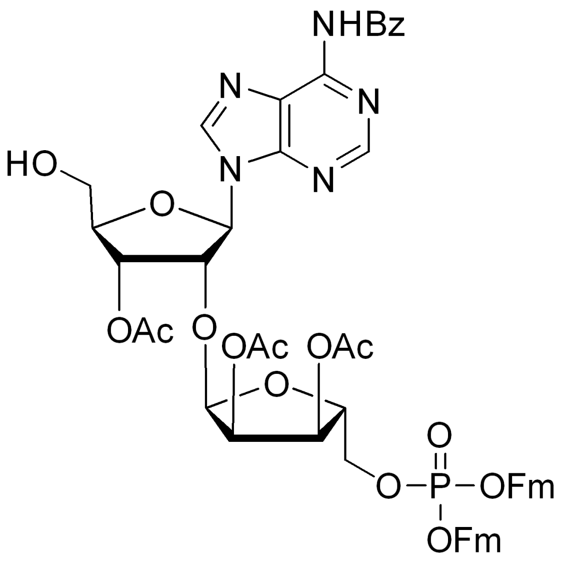

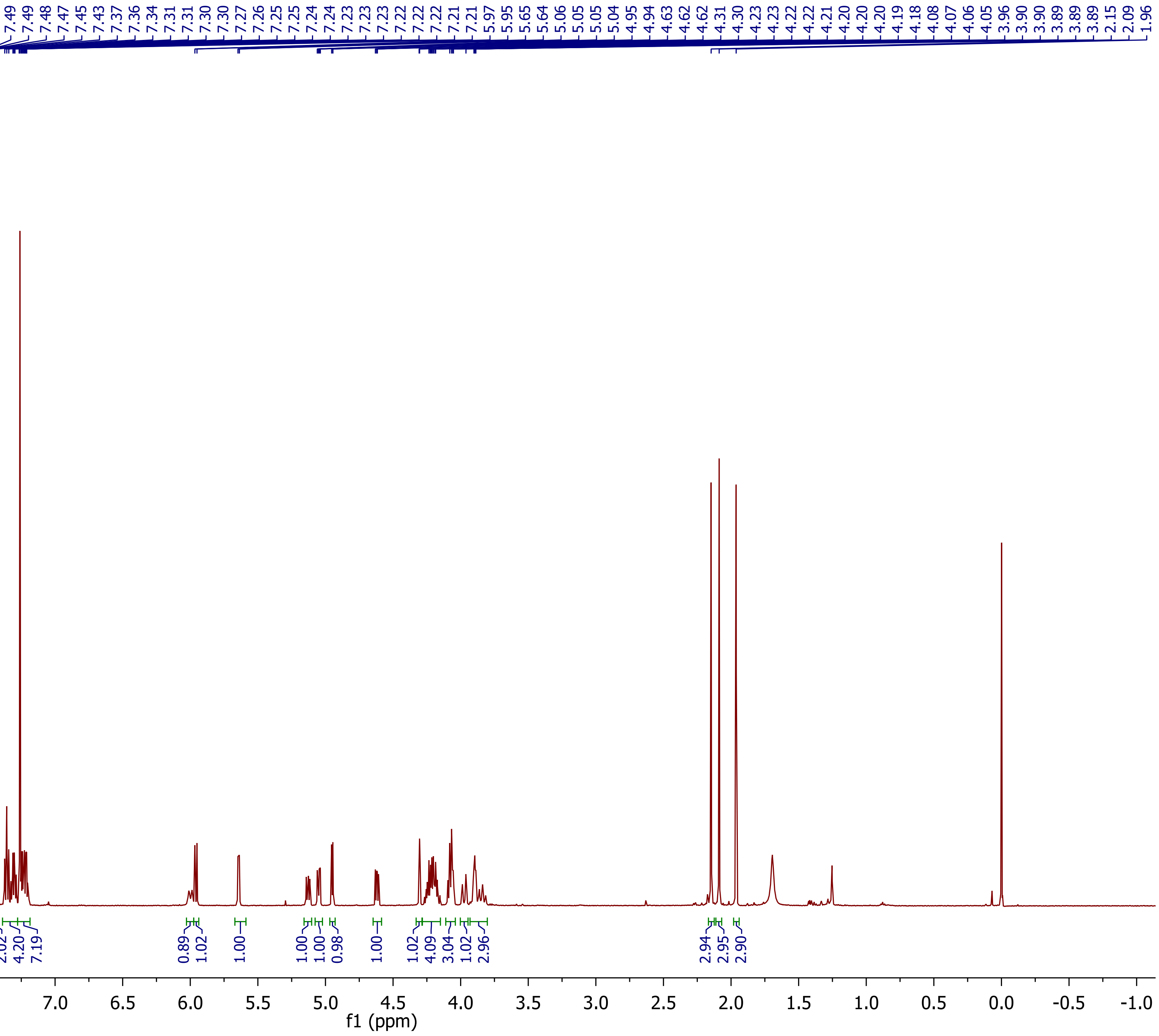

Compound **18**, ^{13}C--NMR, $CDCl_3$, 126 MHz

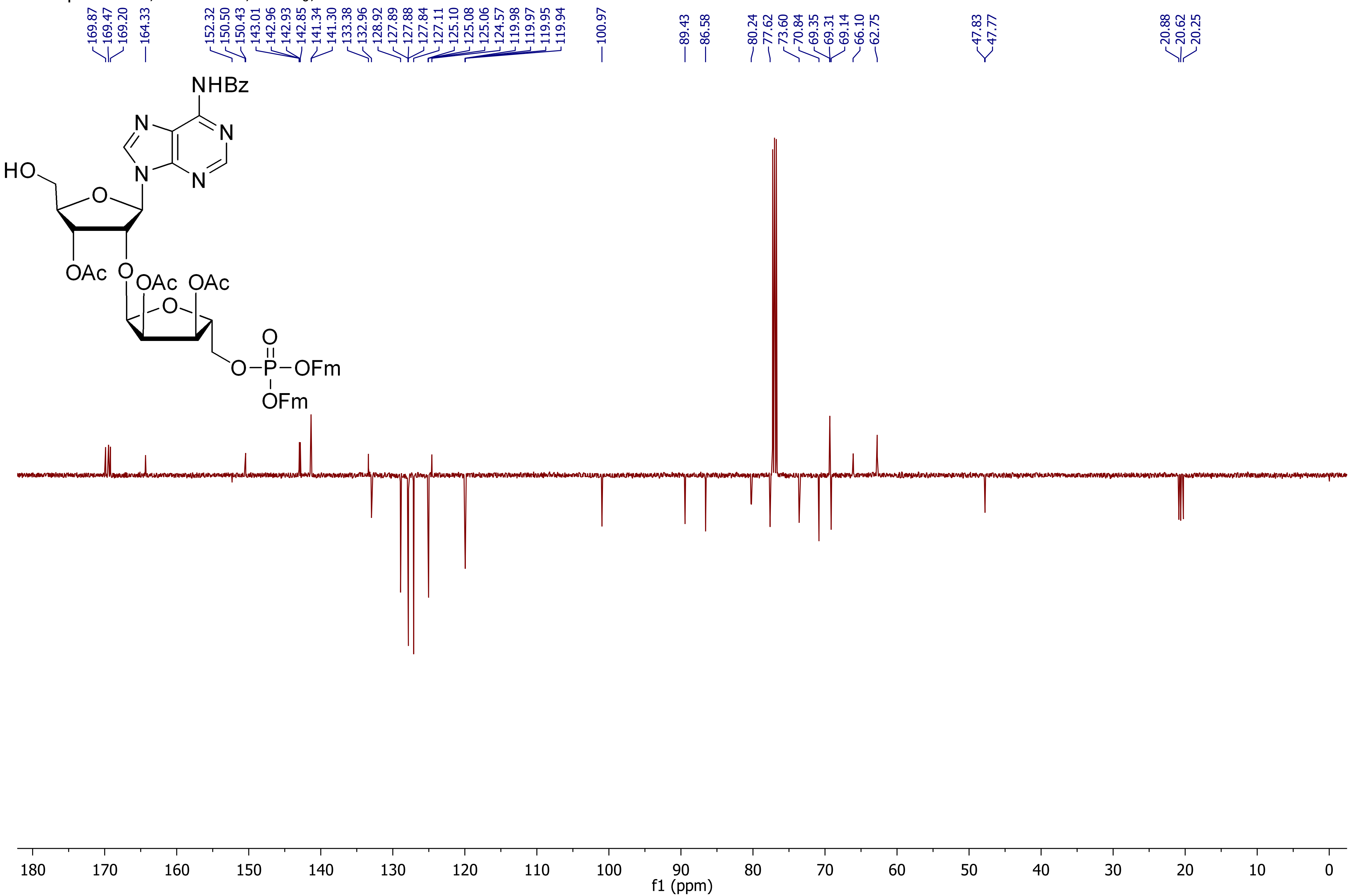

Compound **18**, ^{31}P--NMR, $CDCl_3$, 202 MHz

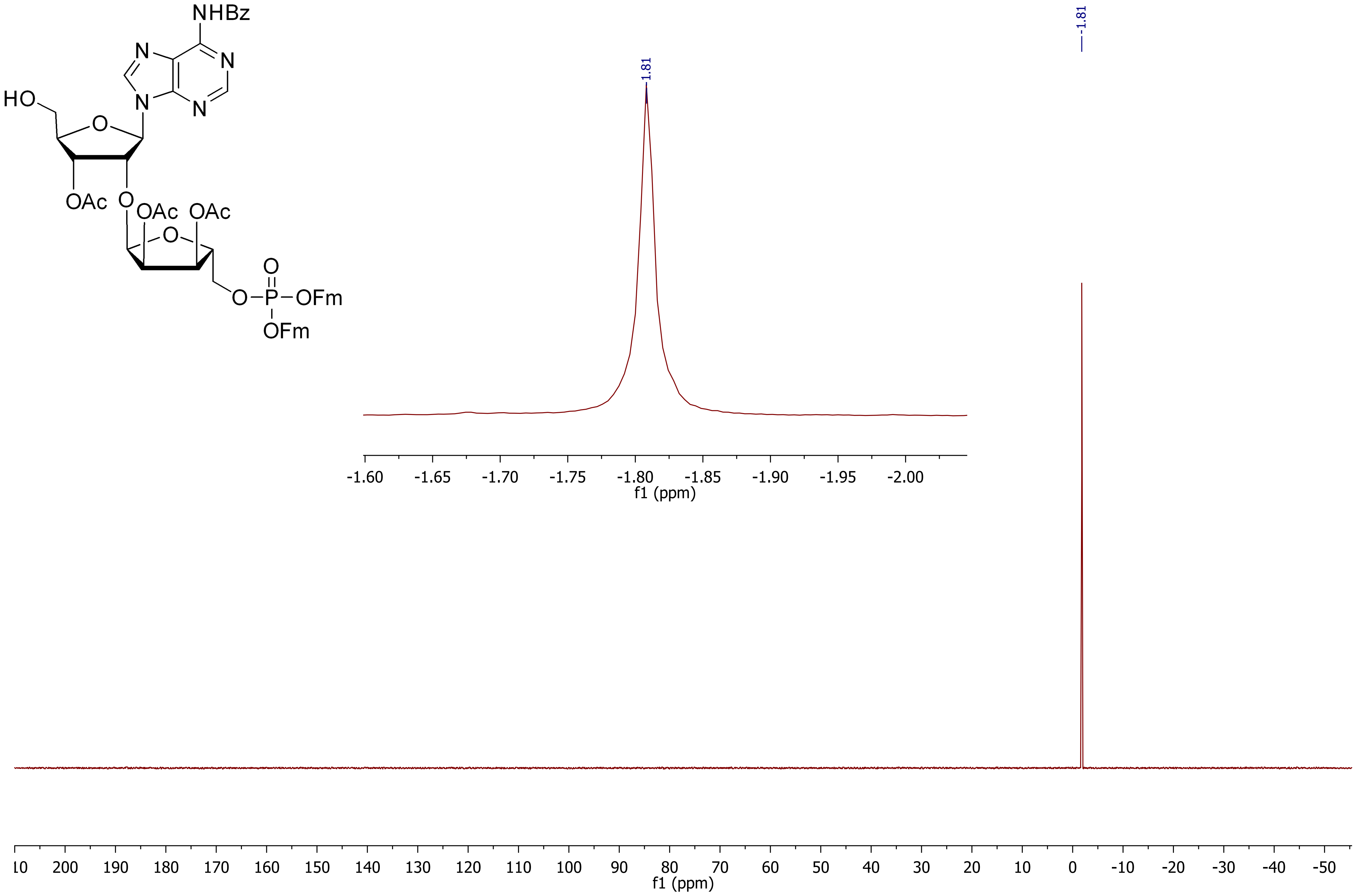

Compound **3**, ^{1}H--NMR, $CDCl_3$, 500 MHz

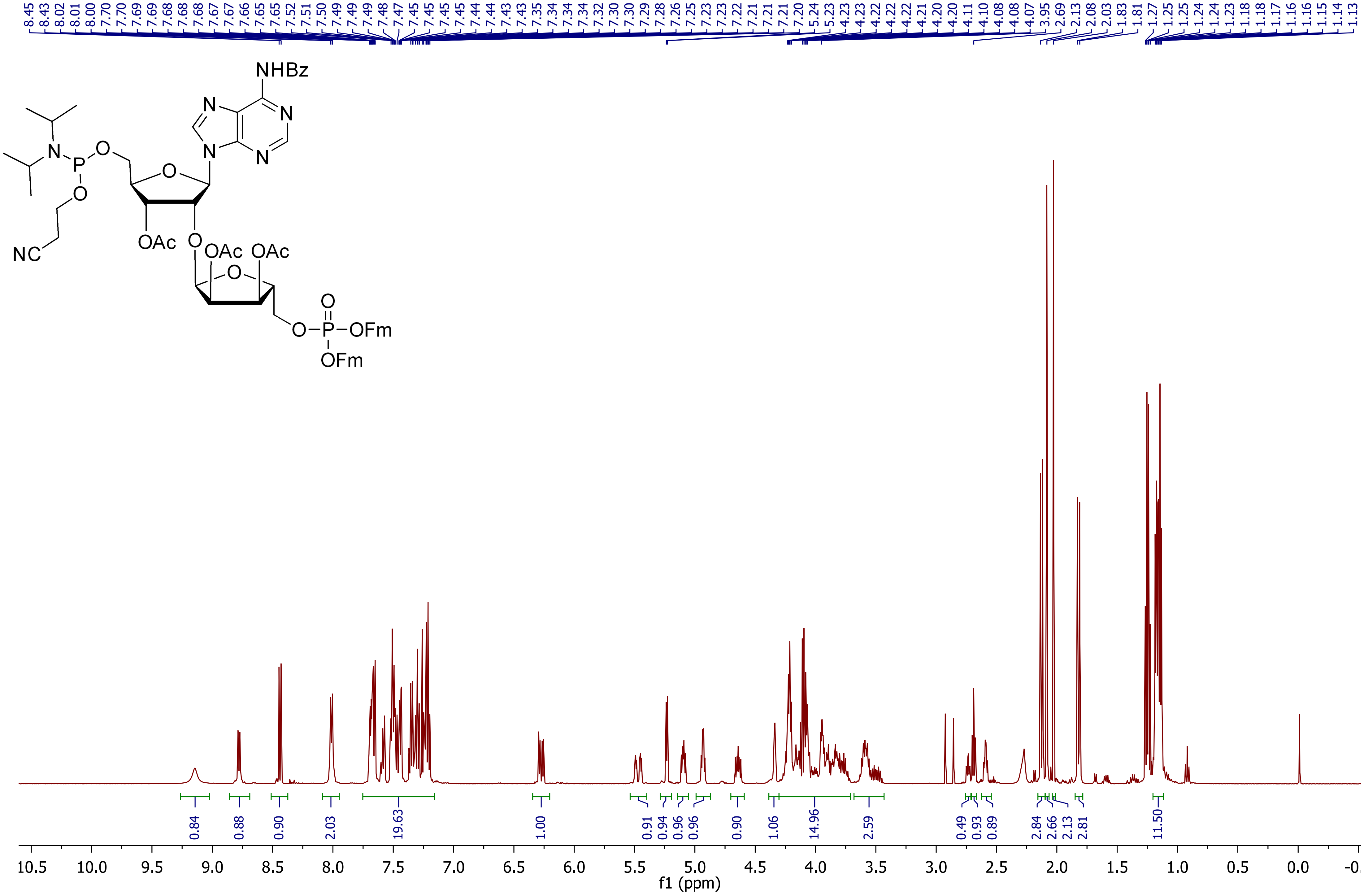

Compound **3**, ^{13}C--NMR, $CDCl_3$, 126 MHz

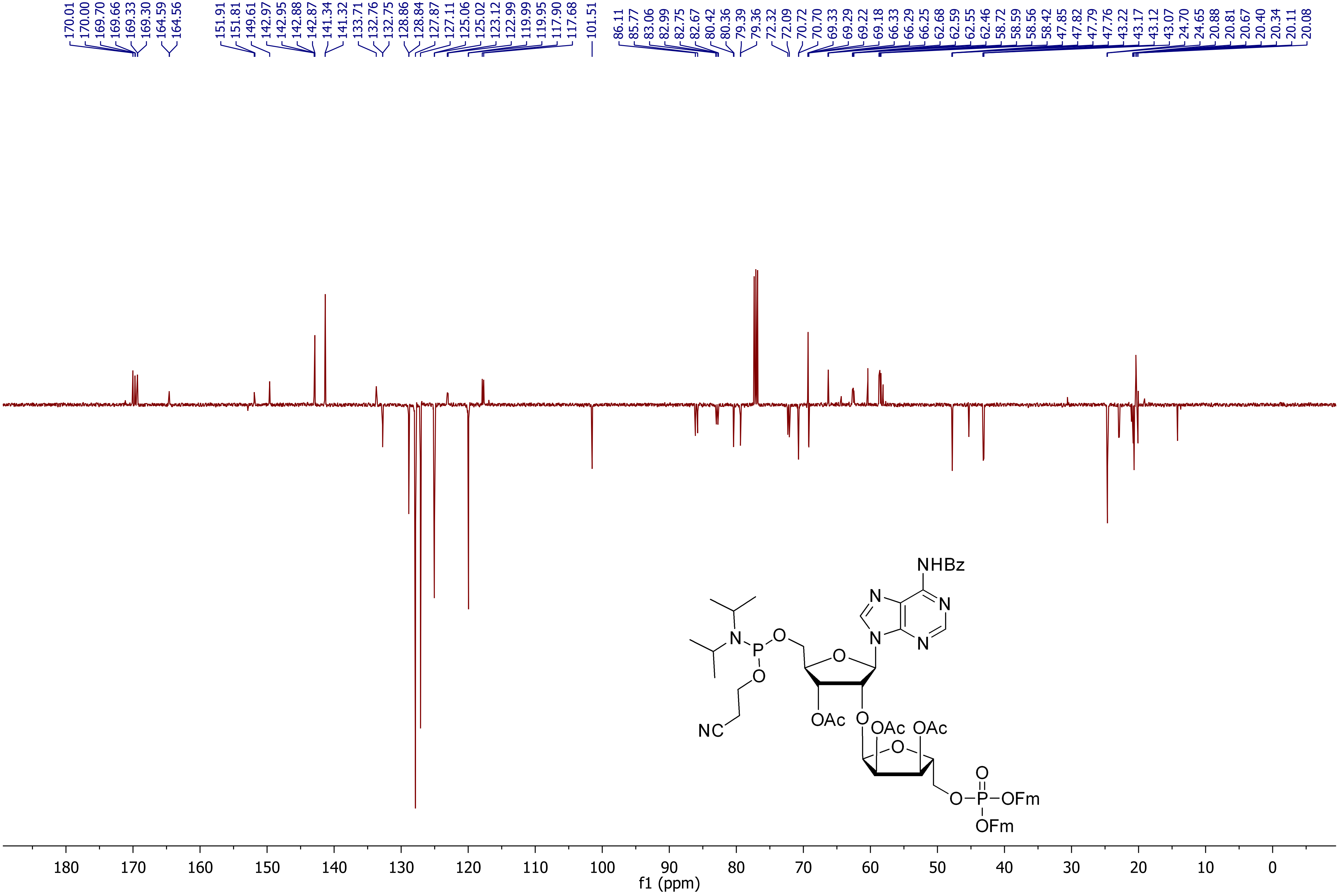

Compound **3**, ^{31}P--NMR, $CDCl_3$, 202 MHz

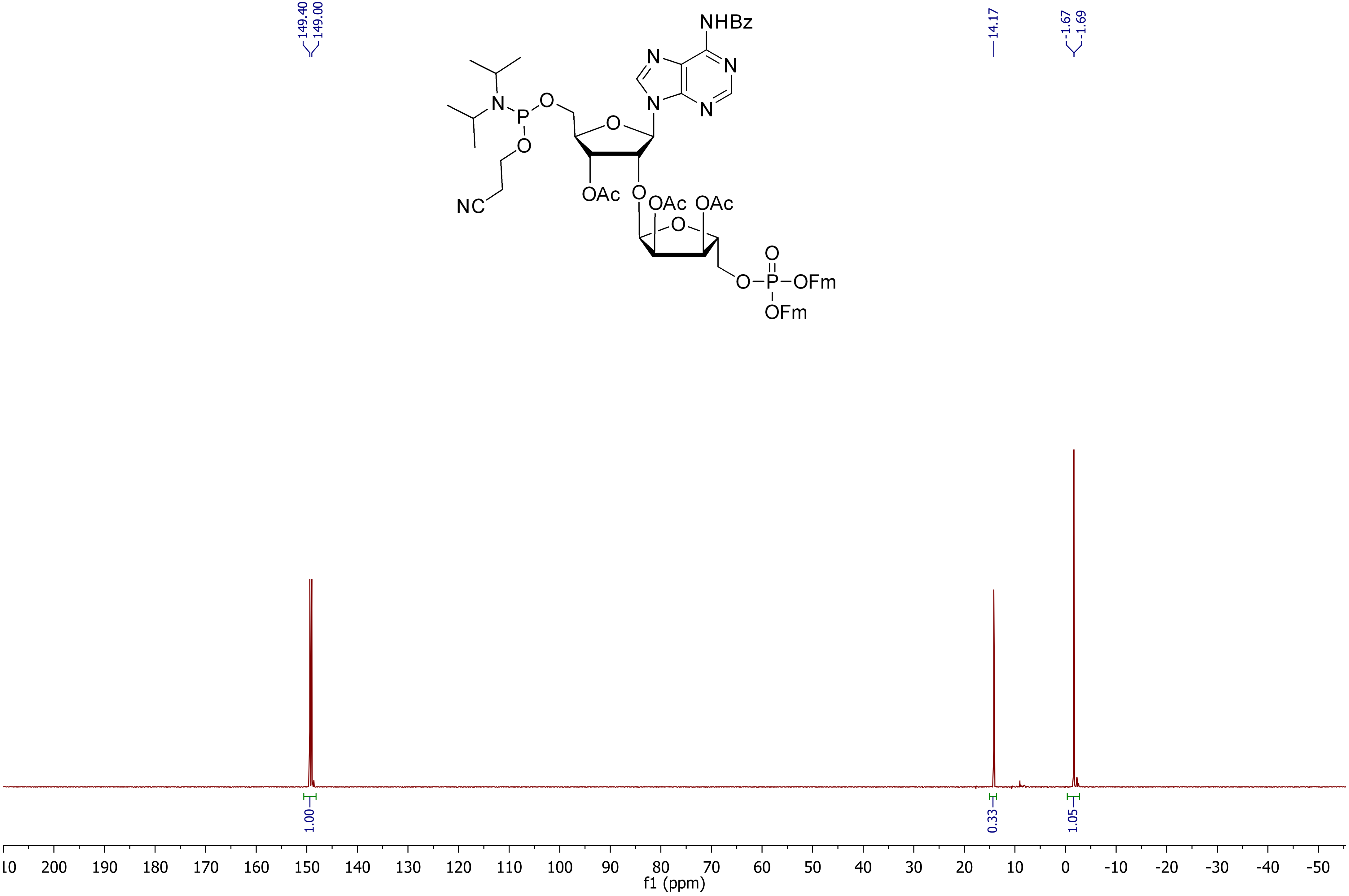

Compound **1a**, ^{1}H--NMR, D_2O, 850 MHz

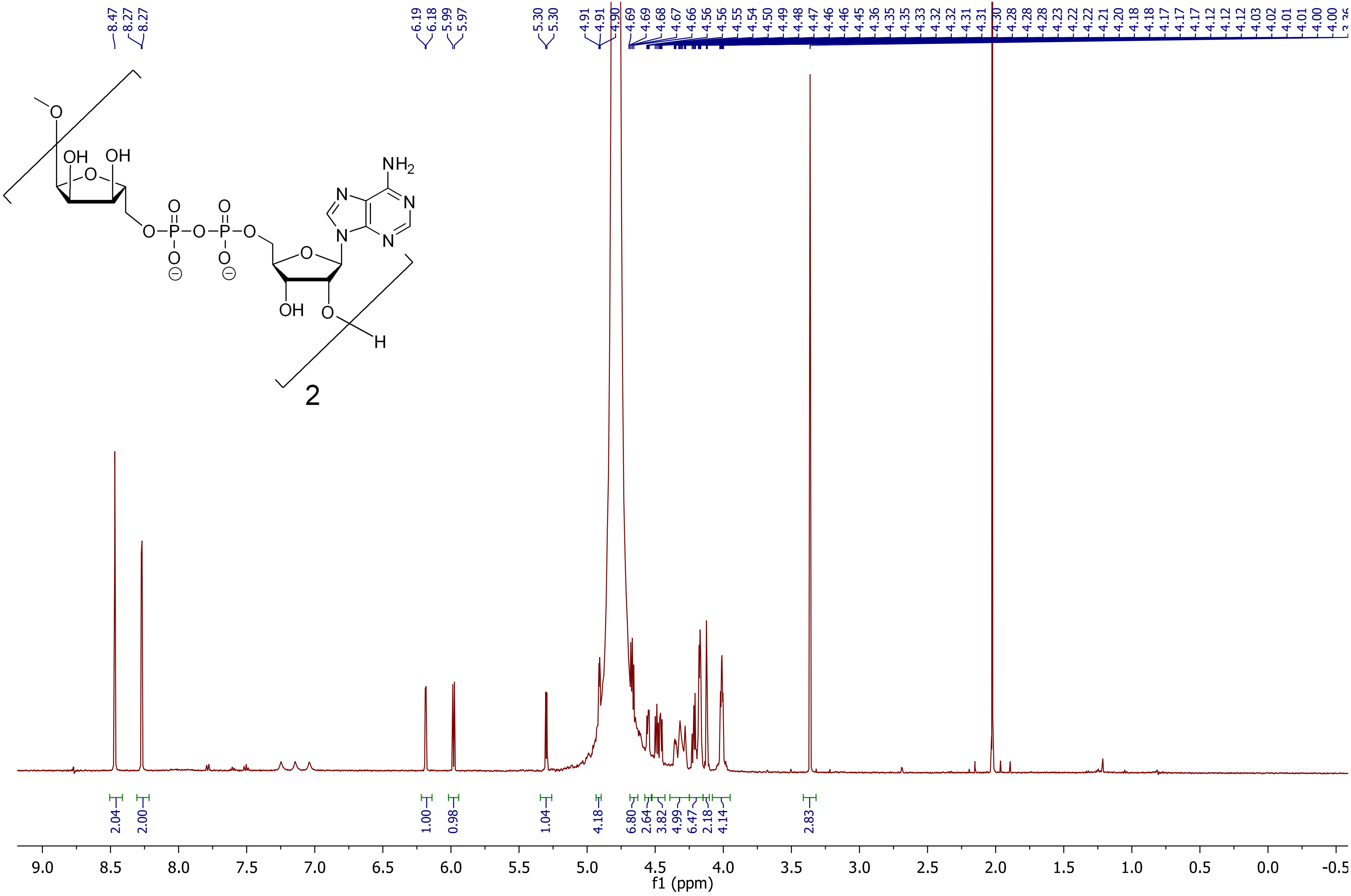

Compound **1a**, ^{1}H--NMR, D_2O, H_2O suppression, 850 MHz

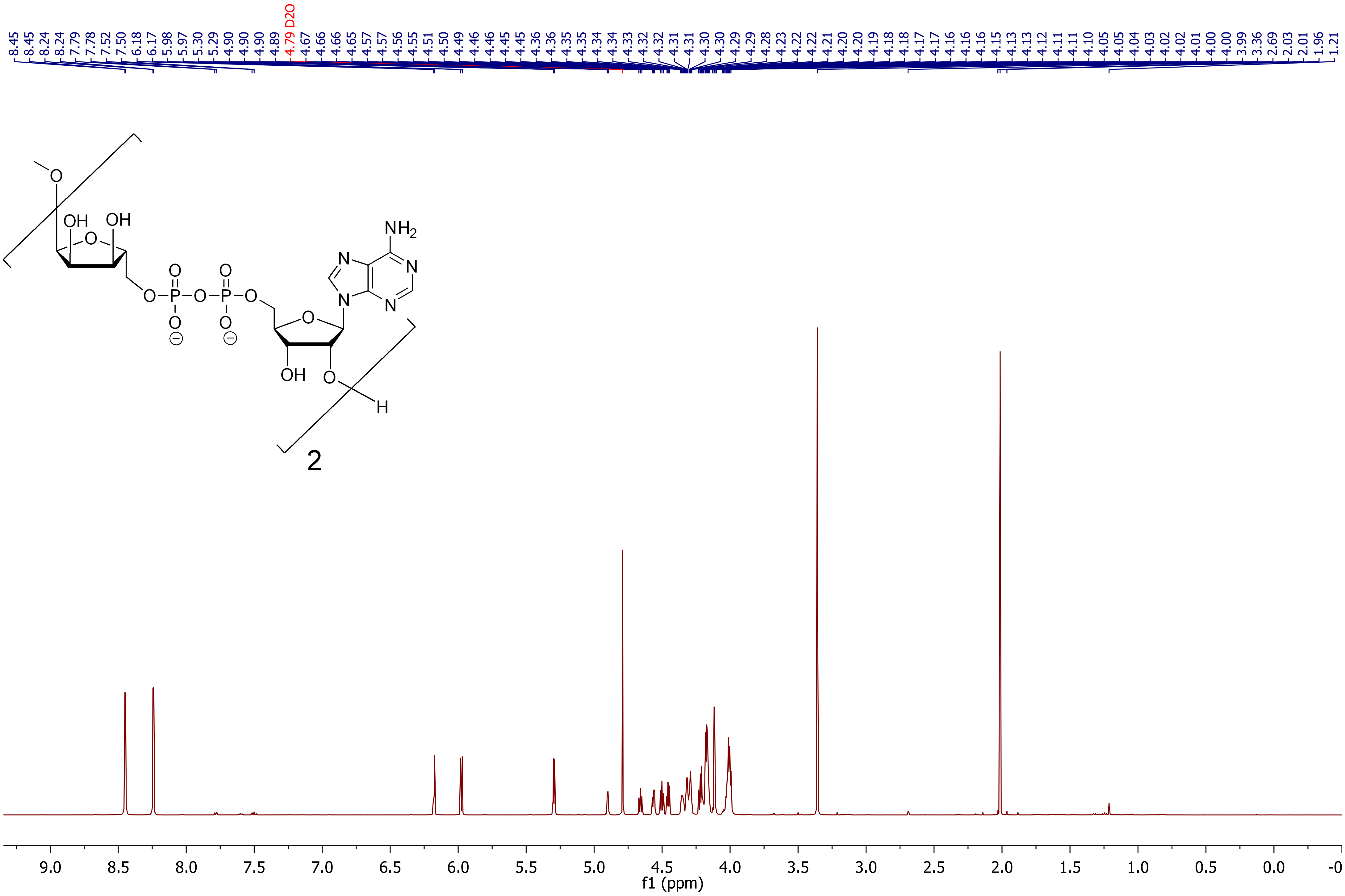

2

Compound **1a**, ^{13}C--NMR, D_2O, 202 MHz

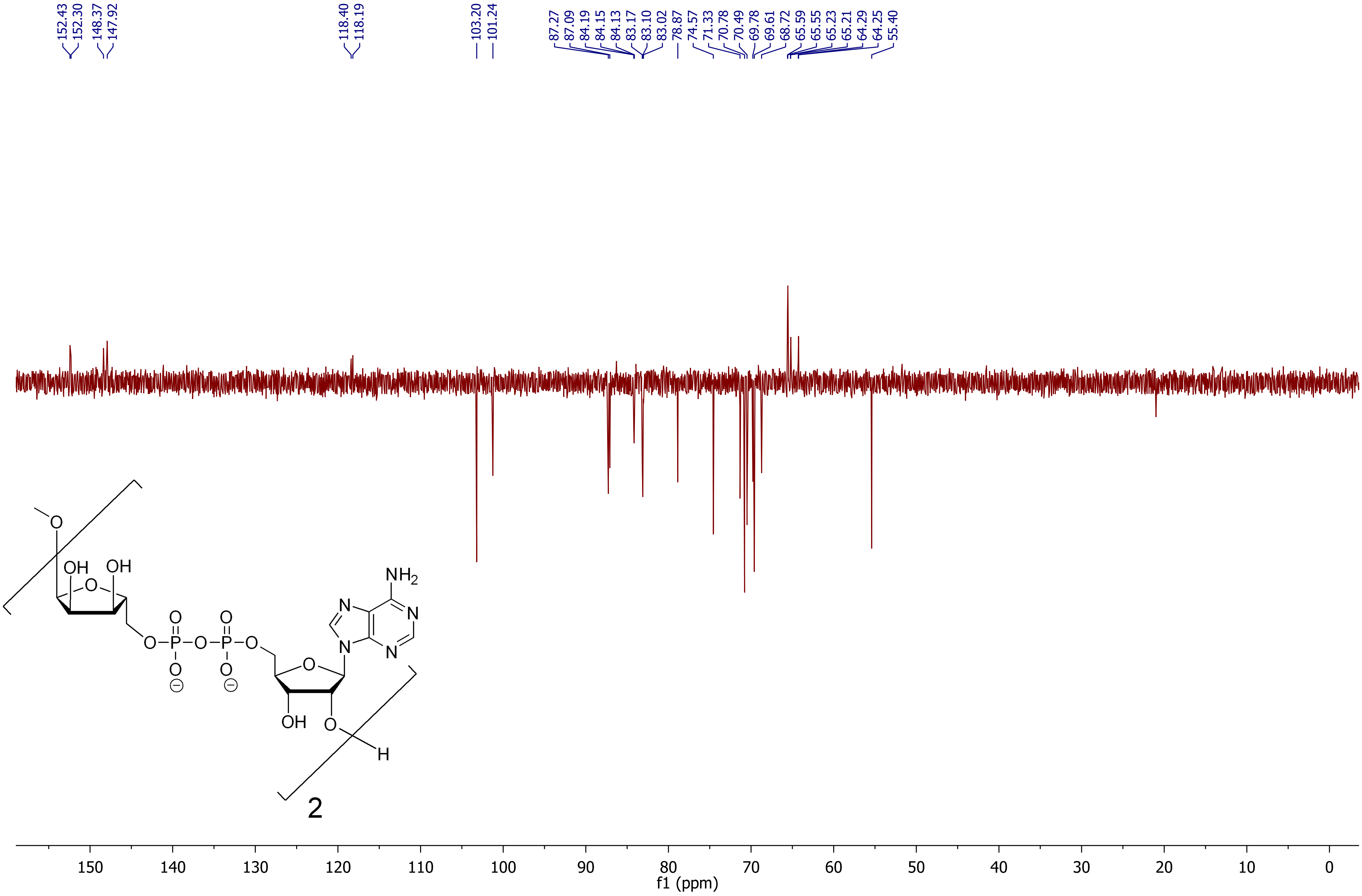

Compound **1a**, ^{31}P--NMR, D_2O, 202 MHz

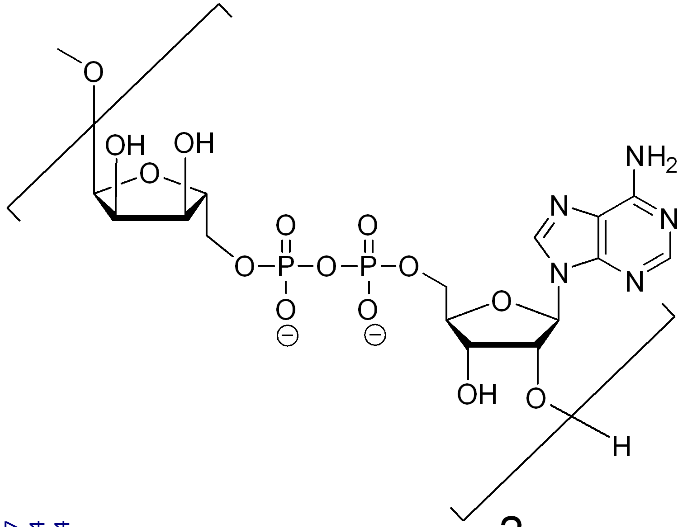

2

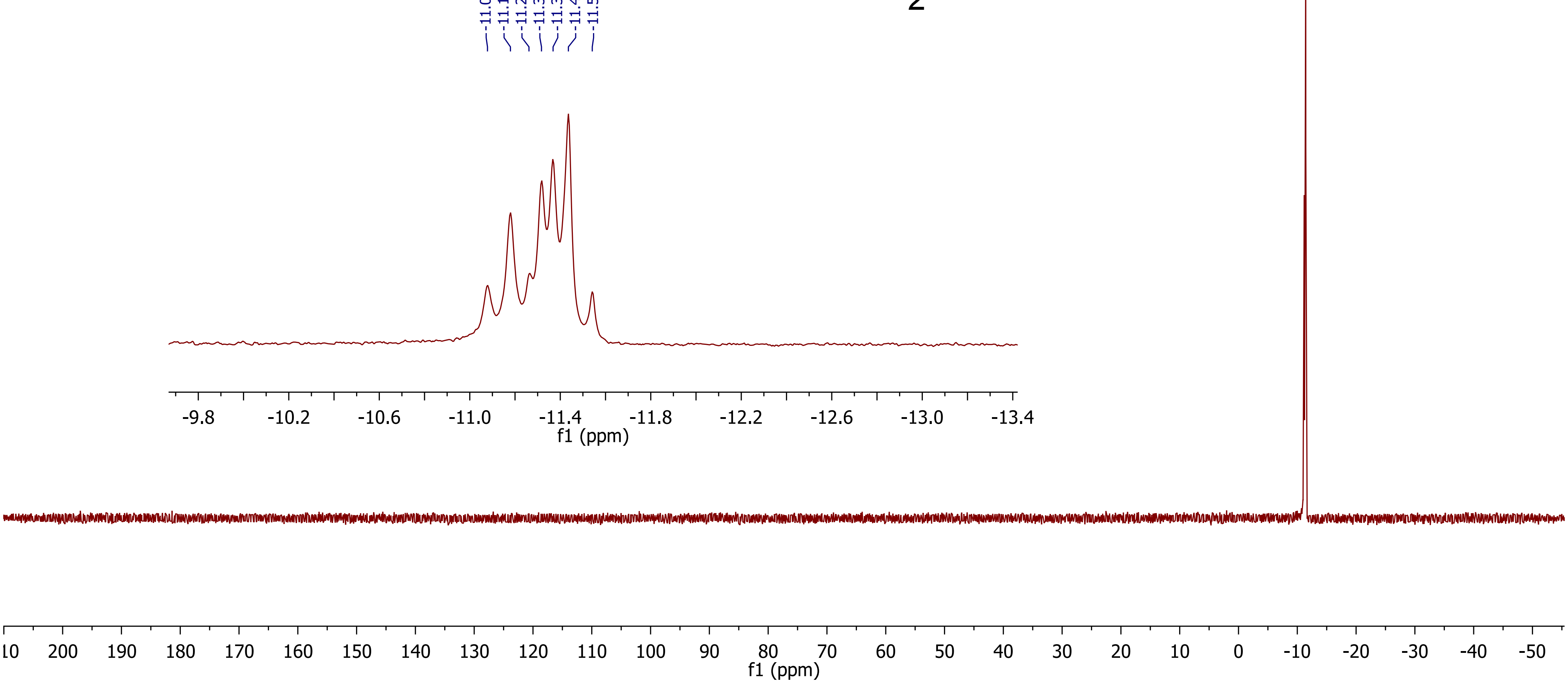

Compound **1a**, COSY, D_2O, 850 MHz

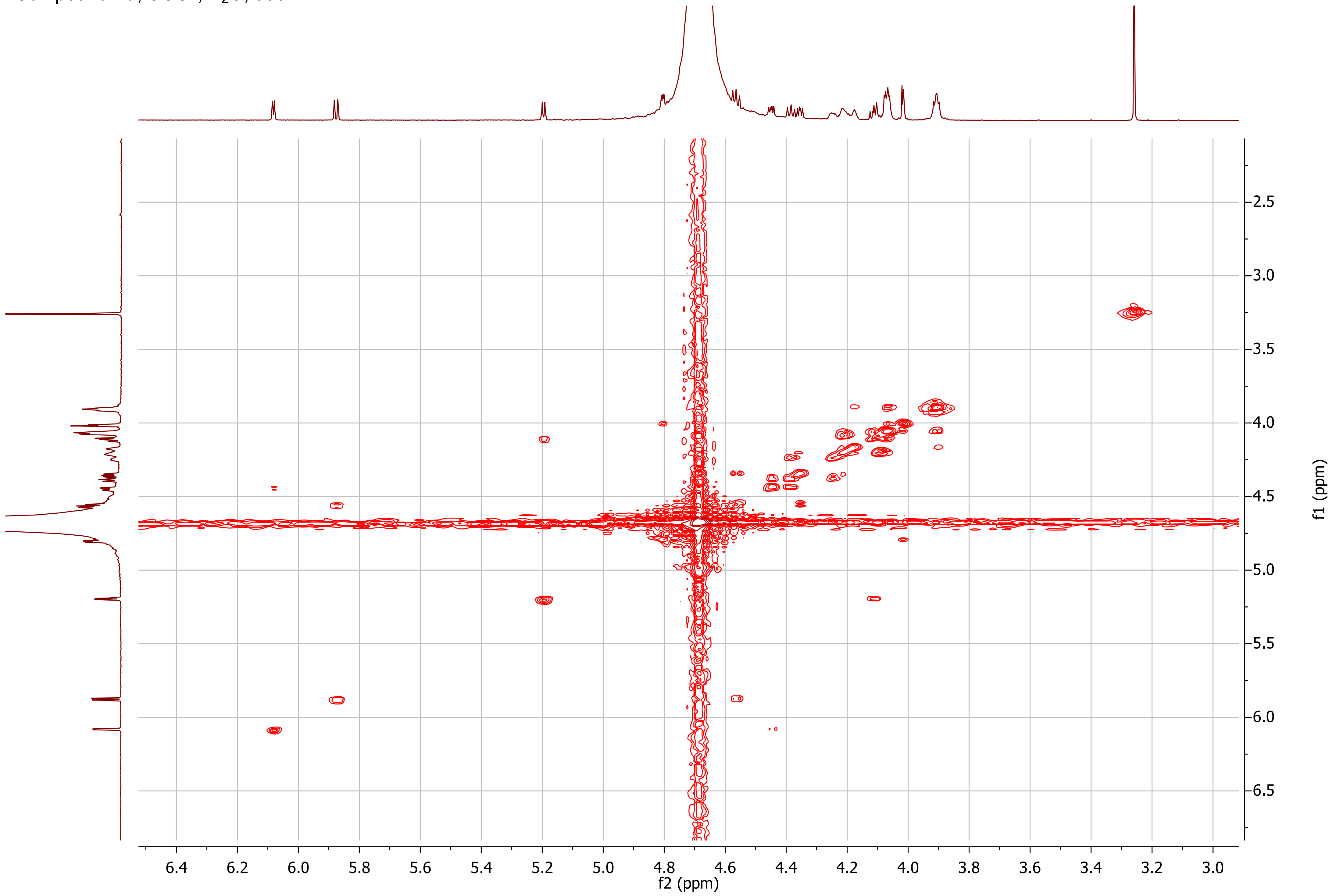

Compound **1a**, HSQC, D_2O, 850 MHz

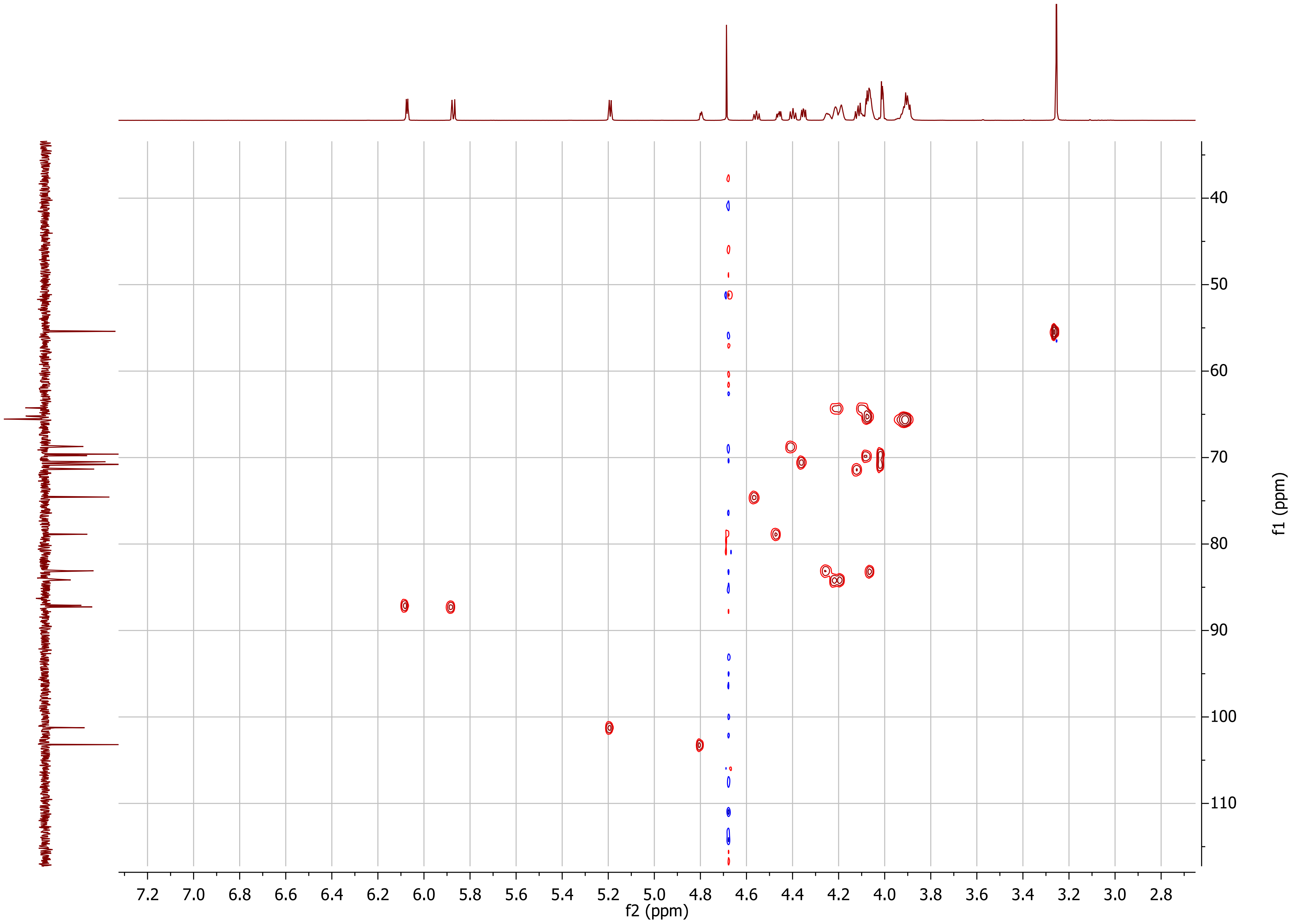

Compound **1b**, ^{1}H--NMR, D_2O, 500 MHz

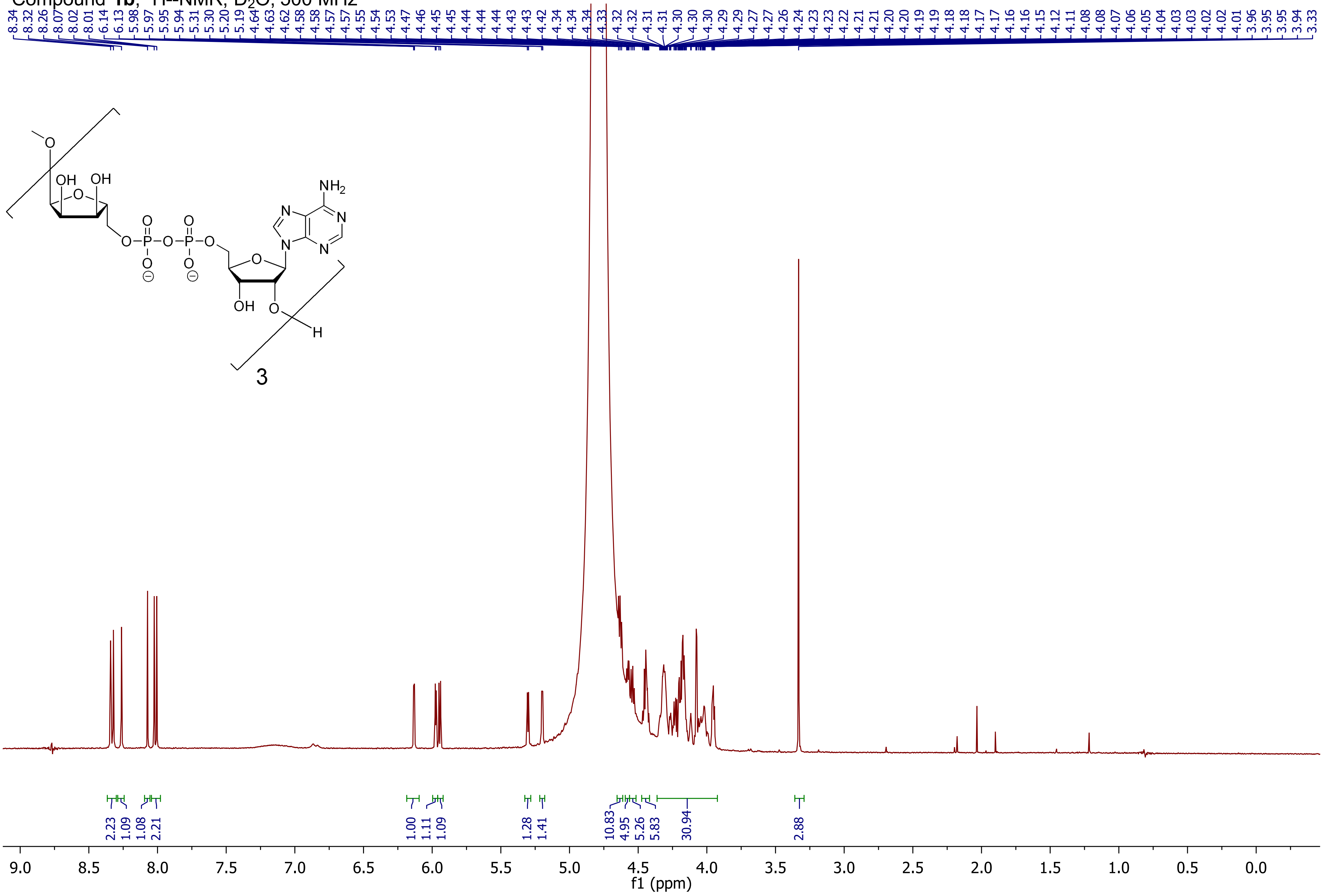

Compound **1b**, ^{1}H--NMR, D_2O, H_2O suppression, 500 MHz

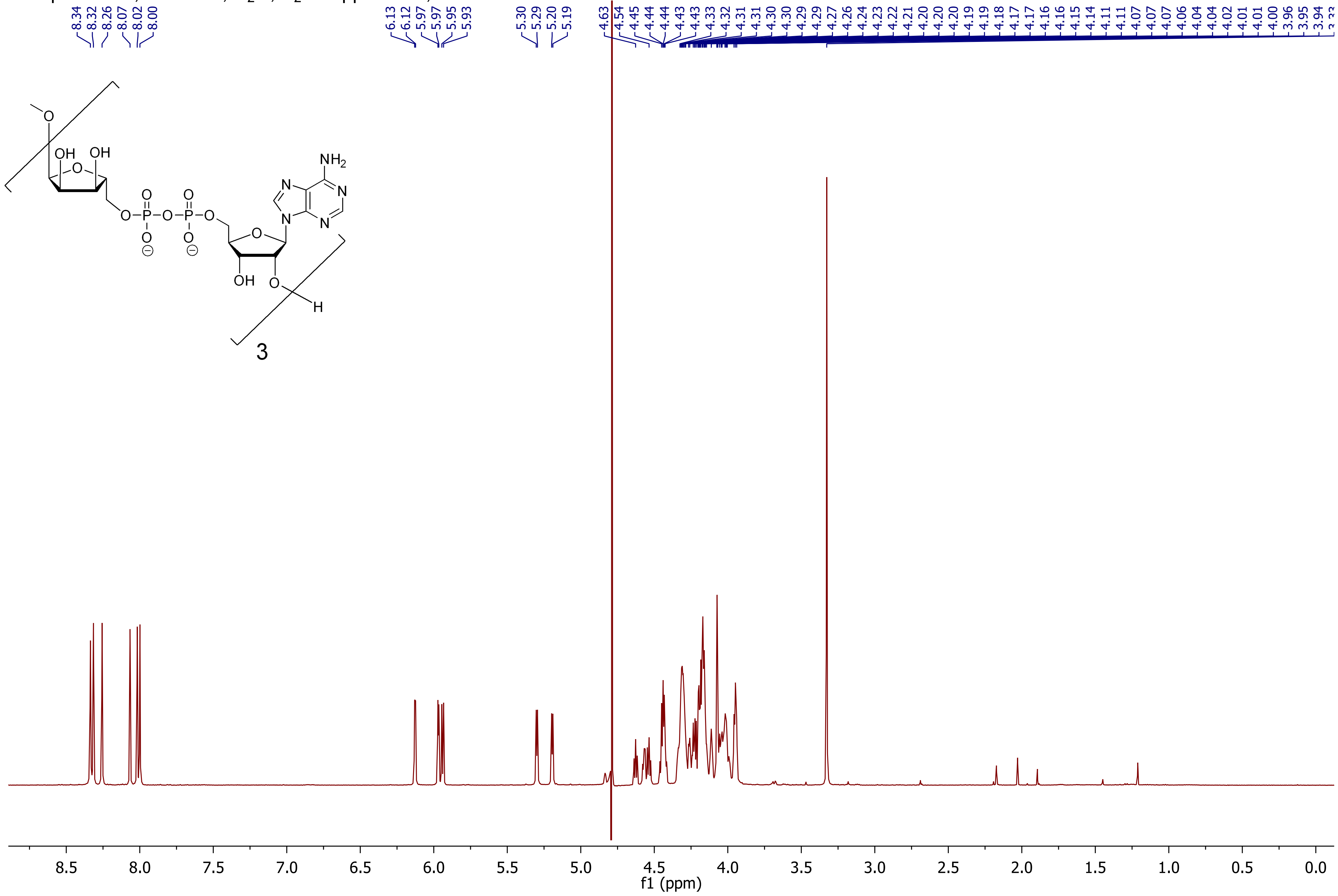

Compound **1b**, ^{13}C--NMR, D_2O, 214 MHz

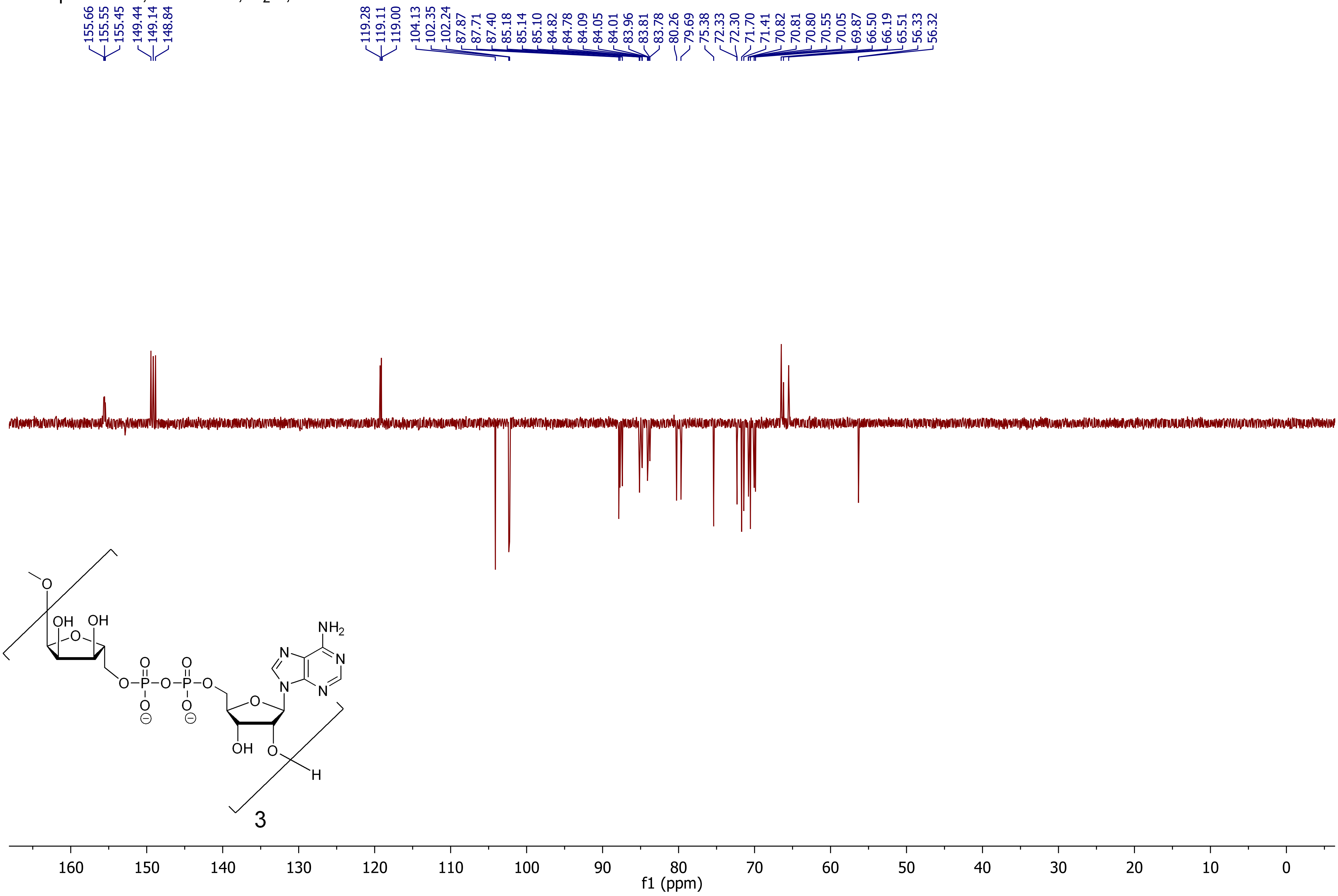

Compound **1b**, ^{31}P--NMR, D_2O, 202 MHz

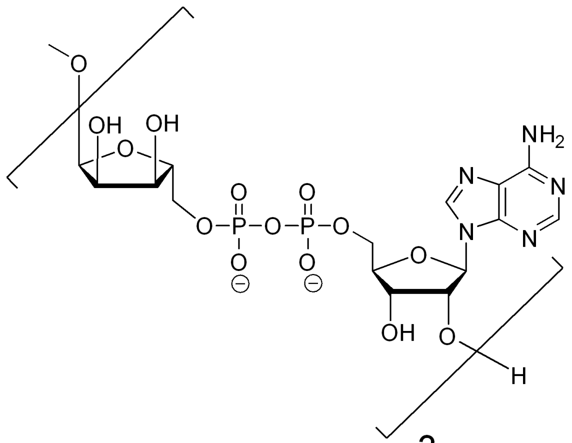

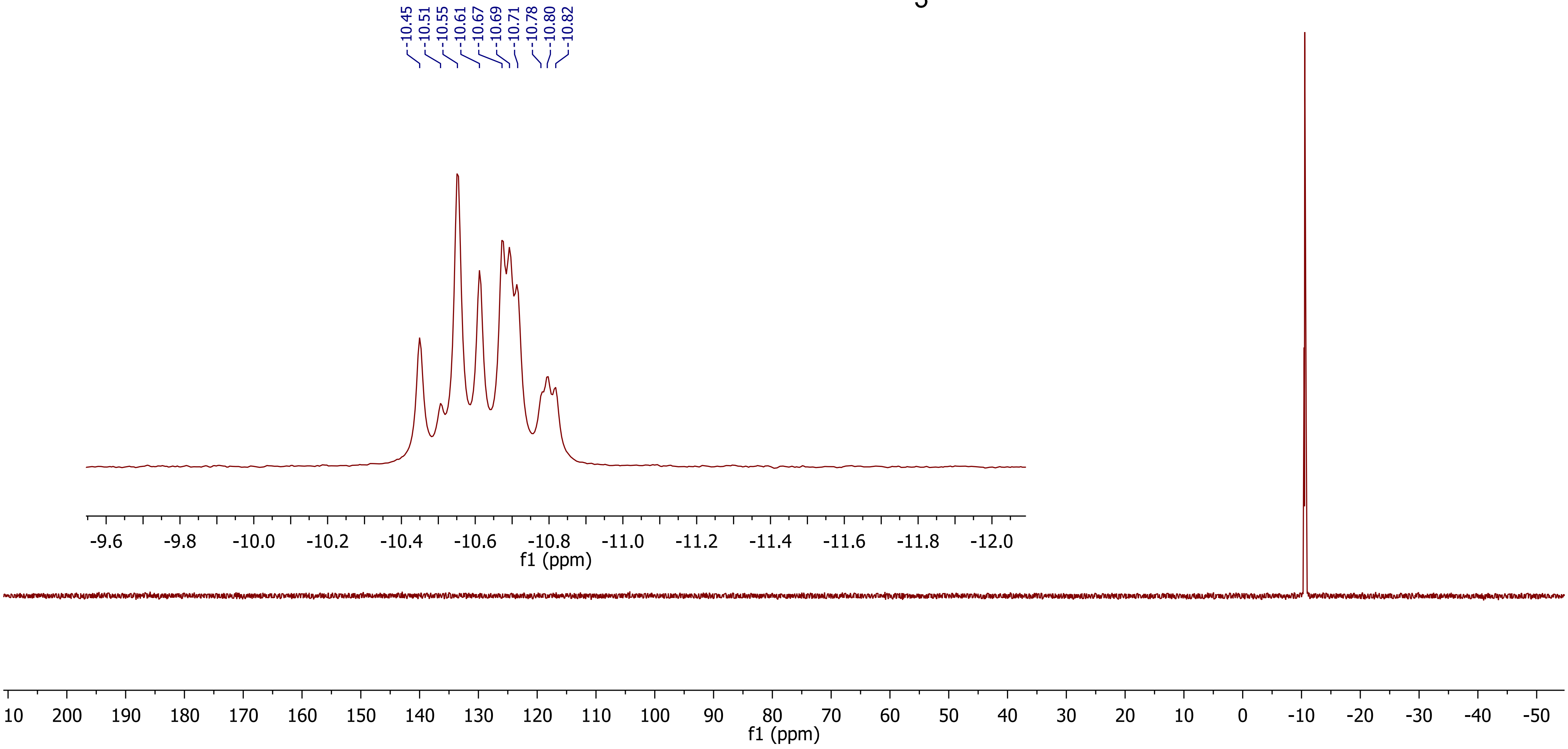

Compound **1b**, COSY, D_2O, 850 MHz

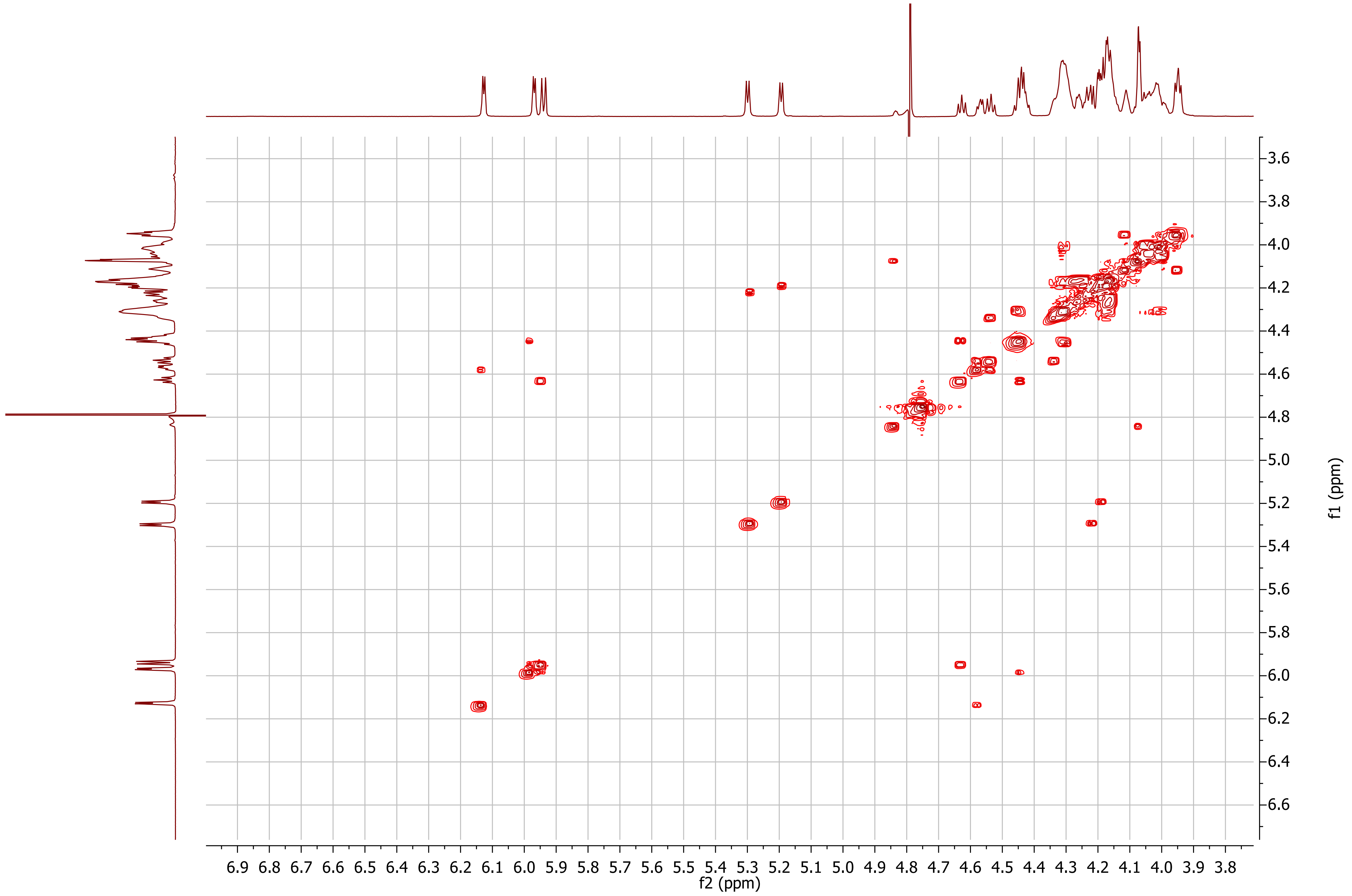

Compound **1b**, HSQC, D_2O, 850 MHz

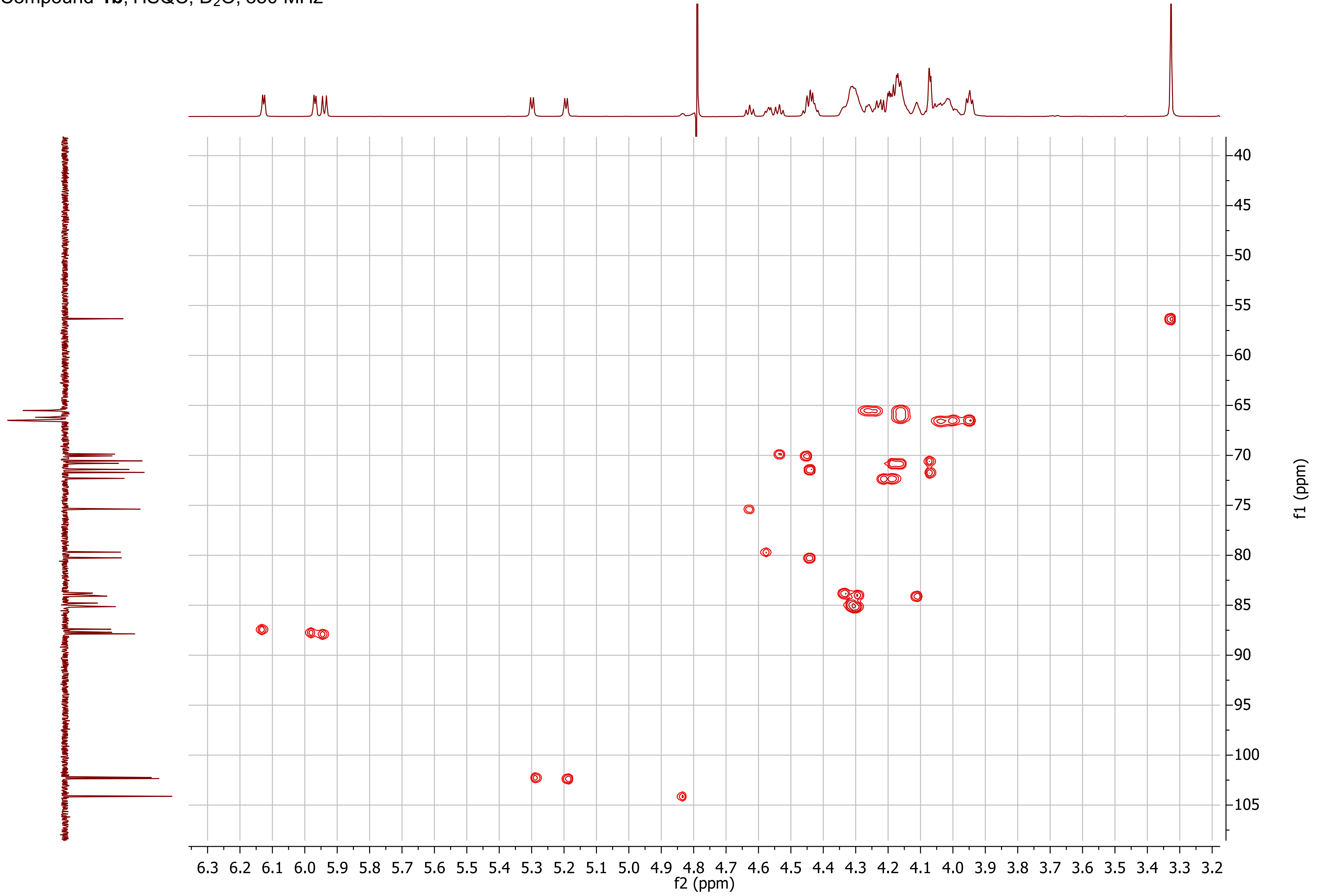

Compound **1c**, ^{1}H--NMR, D_2O, 850 MHz

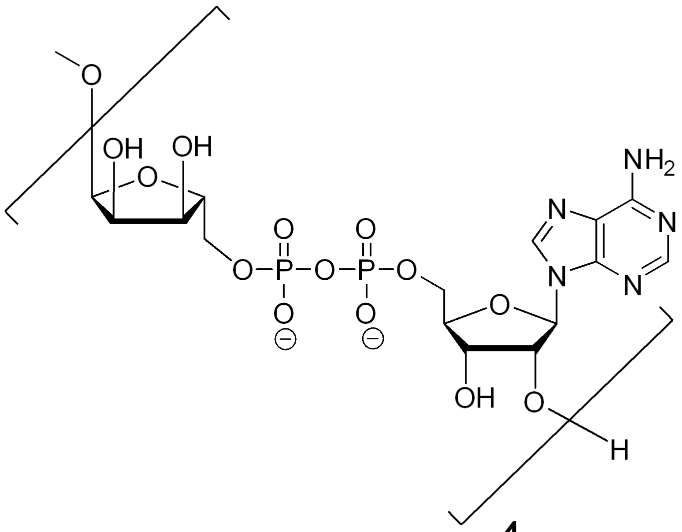

4

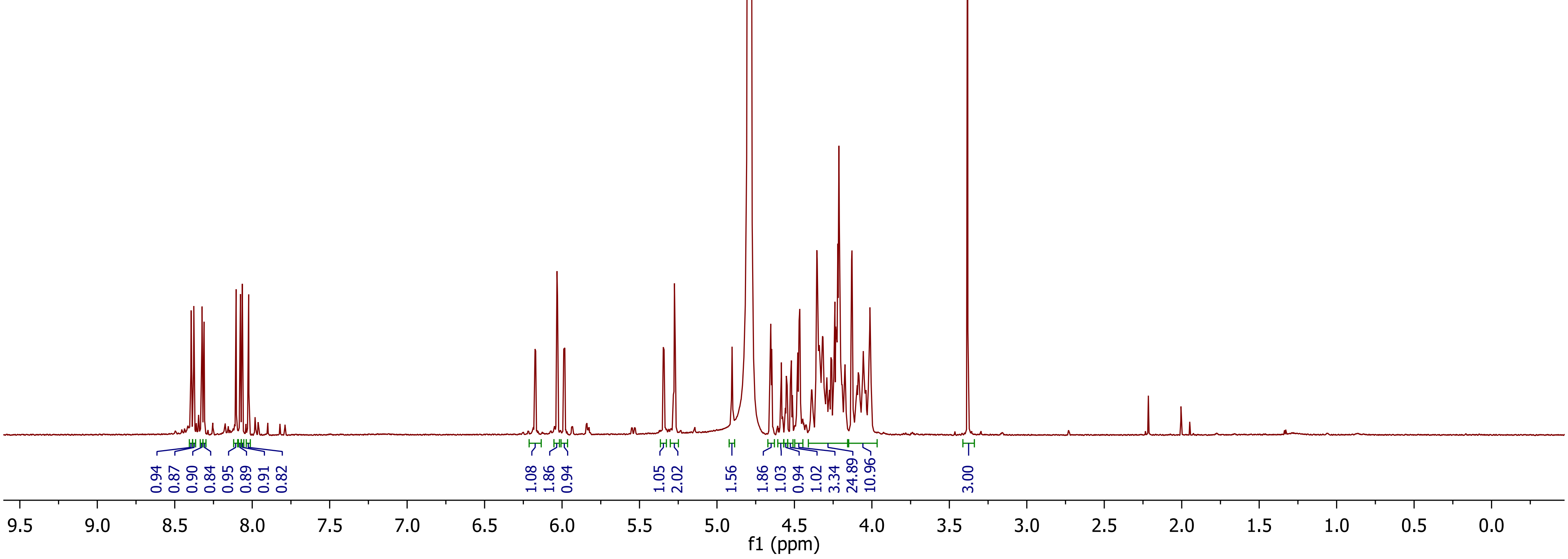

Compound **1c**, ^{1}H--NMR, D_2O, H_2O suppression, 850 MHz

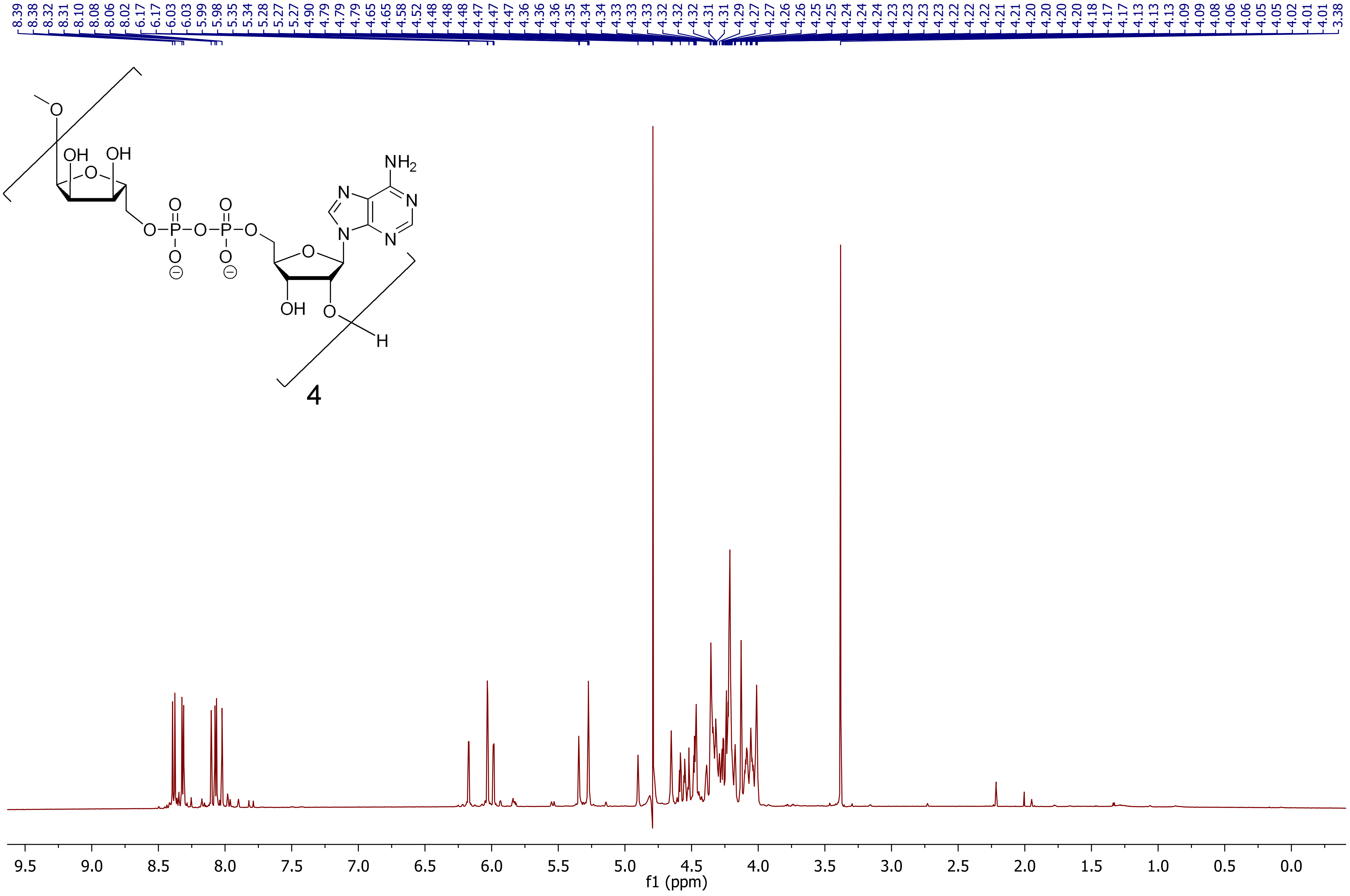

Compound **1c**, ^{31}P--NMR, D_2O, 202 MHz

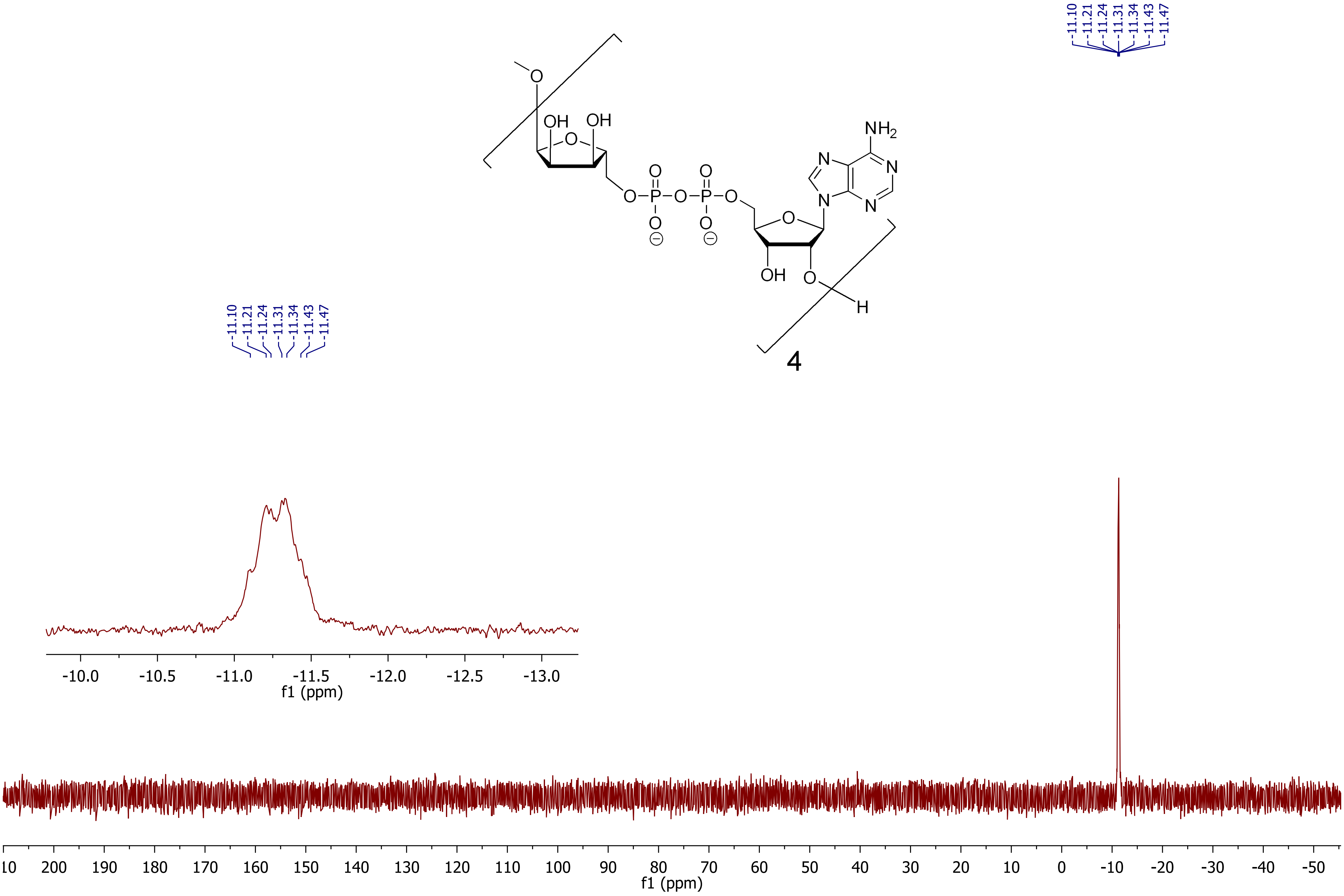

Compound **1c**, COSY, D_2O, 850 MHz

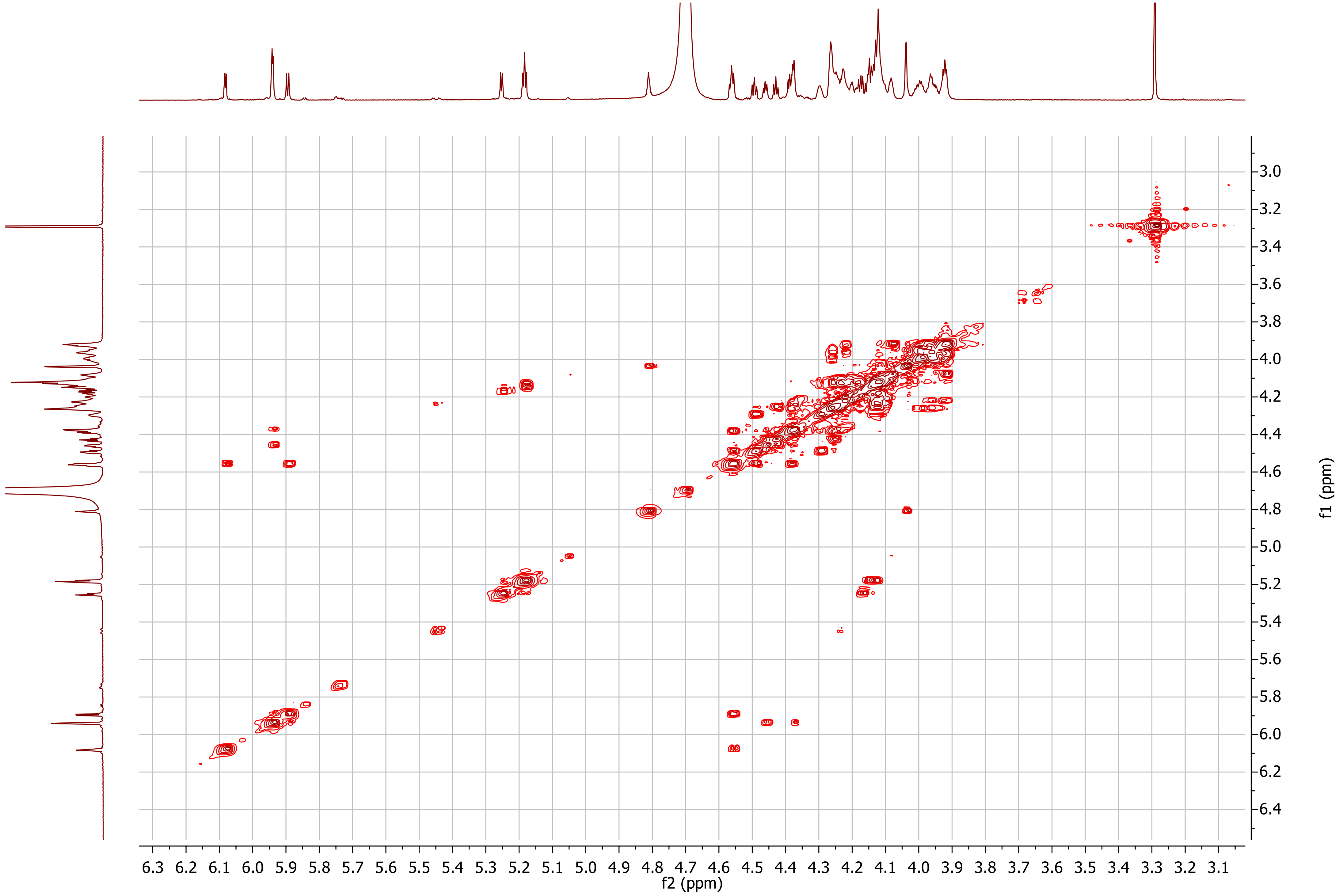

Compound **1d**, ^{1}H--NMR, D_2O, 850 MHz

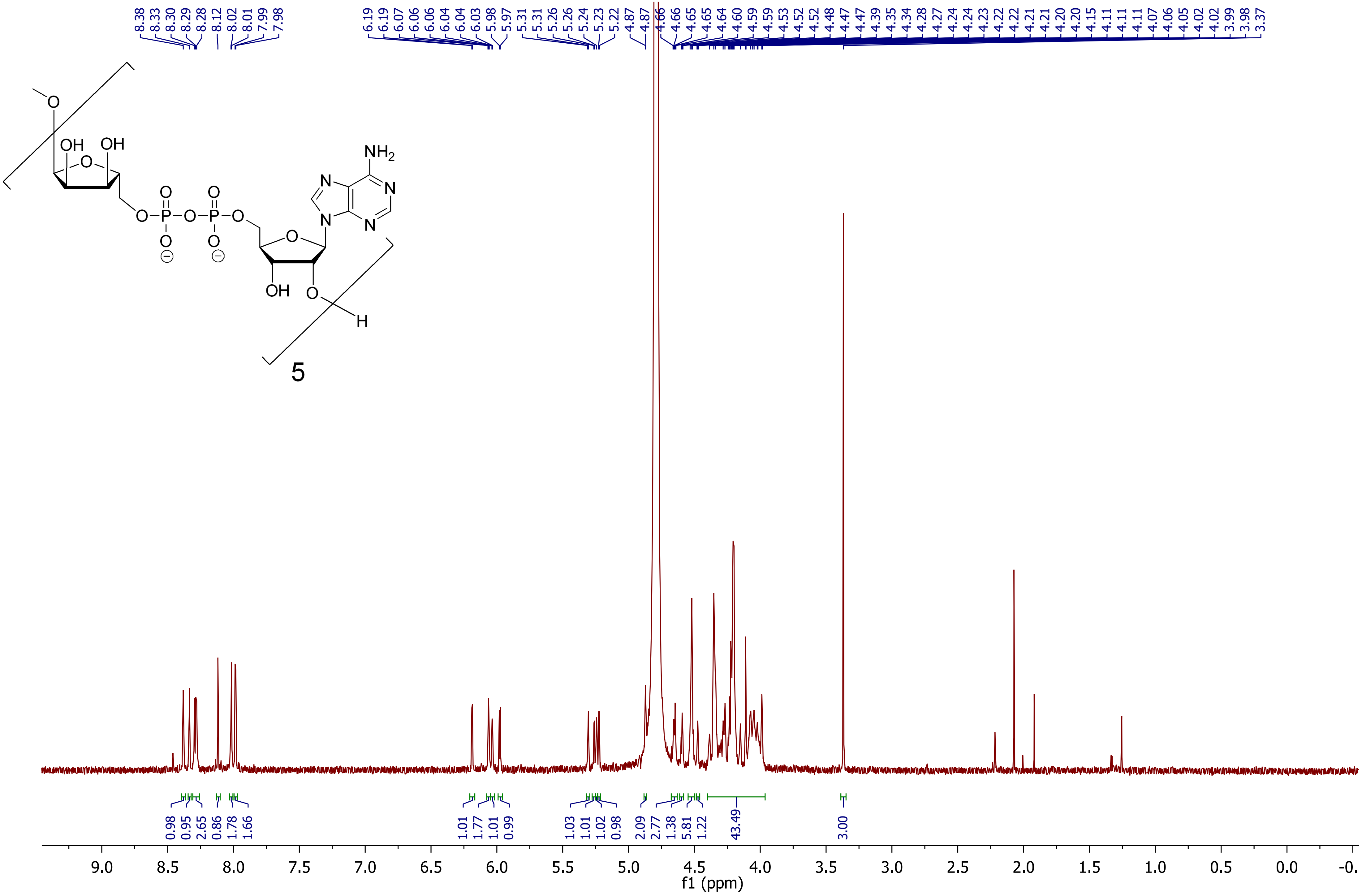

Compound **1d**, ^{1}H--NMR, D_2O, H_2O suppression, 850 MHz

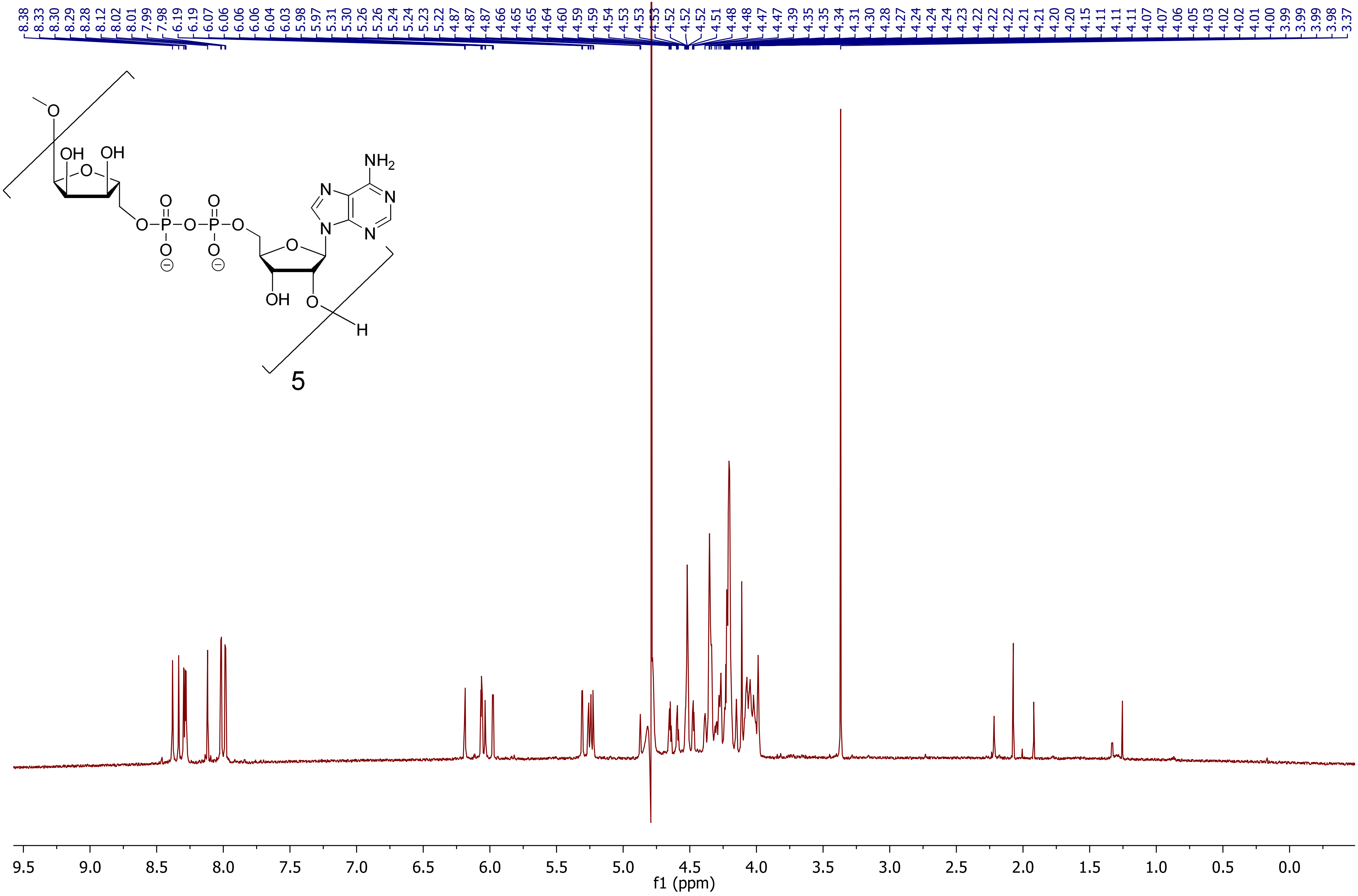

Compound **1d**, ^{31}P--NMR, D_2O, 202 MHz

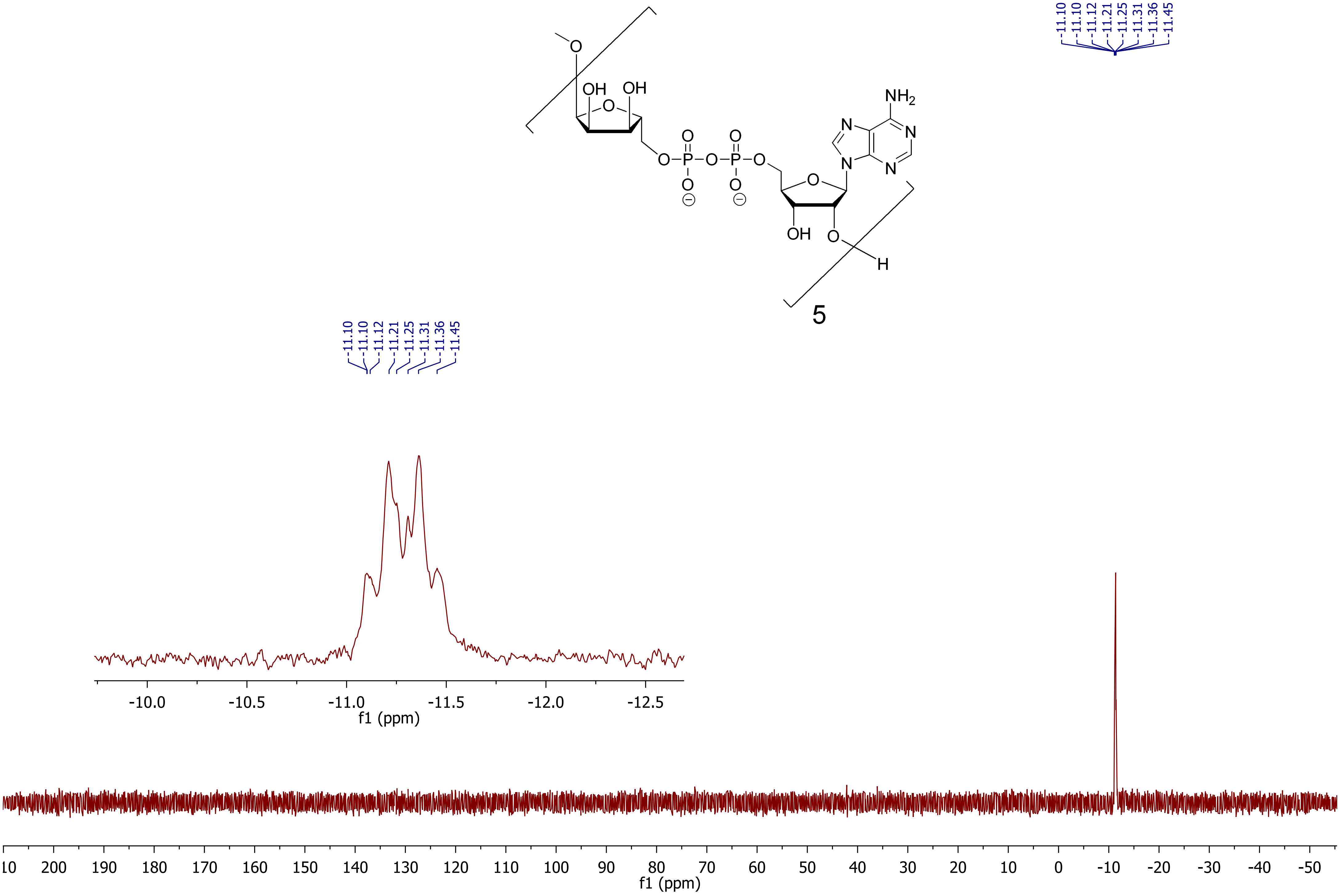

Compound **1d**, COSY, D_2O, 850 MHz

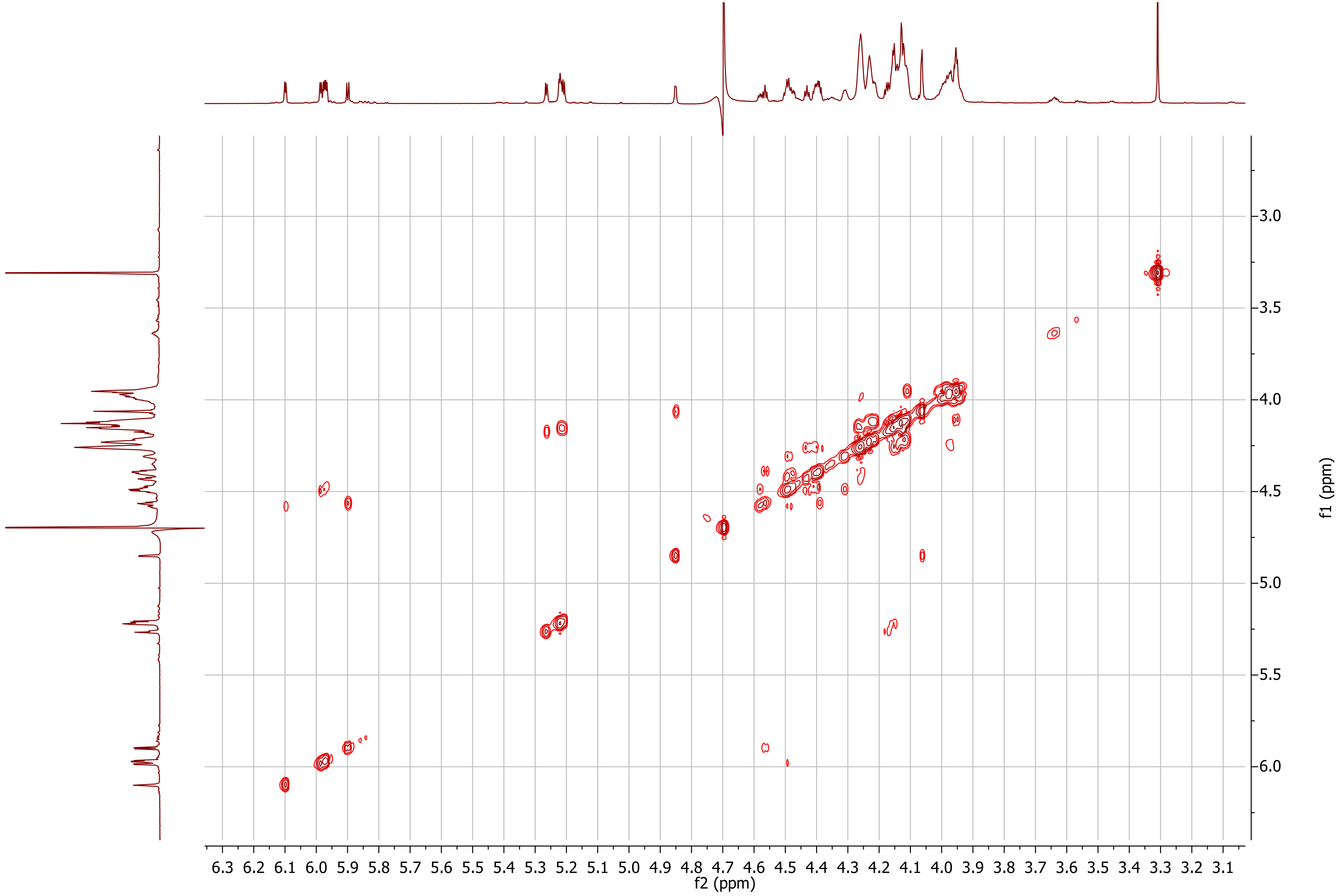

Supplement: SC-012-D1SC02340C-s002 [file SC-012-D1SC02340C-s002.pdf]
